# The Antiproliferative Activity of *Adiantum pedatum* Extract and/or Piceatannol in Phenylhydrazine-Induced Colon Cancer in Male Albino Rats: The miR-145 Expression of the *PI-3K*/*Akt*/*p53* and *Oct4*/*Sox2*/*Nanog* Pathways

**DOI:** 10.3390/molecules28145543

**Published:** 2023-07-20

**Authors:** Tarek Khamis, Abd Al-Aziz Abas Diab, Mansour H. Zahra, Samih Ebrahim El-Dahmy, Basant Ahmed Abd Al-Hameed, Adel Abdelkhalek, Mahmoud A. Said, Hussein Abdellatif, Liana Mihaela Fericean, Ioan Banatean-Dunea, Ahmed Hamed Arisha, Mai S. Attia

**Affiliations:** 1 Department of Pharmacology and Laboratory of Biotechnology, Faculty of Veterinary Medicine, Zagazig University, Zagazig 44519, Egypt; t.khamis@vet.zu.edu.eg; 2 Zoology Department, Faculty of Science, Zagazig University, Zagazig 44519, Egypt; 3 Department of Pharmacology, Faculty of Pharmacy, Zagazig University, Zagazig 44519, Egypt; 4 Faculty of Veterinary Medicine, Badr University in Cairo, Badr City 11829, Egypt; 5 Zagazig University Hospital, Zagazig University, Zagazig 44511, Egypt; 6 Department of Human and Clinical Anatomy, College of Medicine and Health Sciences, Sultan Qaboos University, Muscat 123, Oman; 7 Anatomy and Embryology Department, Faculty of Medicine, Mansoura University, Mansoura 35516, Egypt; 8 Department of Biology, Faculty of Agriculture, University of Life Sciences, King Mihai I” from Timisoara [ULST], Aradului St. 119, 300645 Timisoara, Romania; 9 Department of Animal Physiology and Biochemistry, Faculty of Veterinary Medicine, Badr University in Cairo, Badr City 11829, Egypt; 10 Department of Physiology, Laboratory of Biotechnology, Faculty of Veterinary Medicine, Zagazig University, Zagazig 44519, Egypt

**Keywords:** colon cancer, phenylhydrazine, antioxidant, apoptotic, piceatannol, *Adiantum pedatum* (AP) extract

## Abstract

Colon cancer is one of the most common types of cancer worldwide, and its incidence is increasing. Despite advances in medical science, the treatment of colon cancer still poses a significant challenge. This study aimed to investigate the potential protective effects of Adiantum pedatum (AP) extract and/or piceatannol on colon cancer induced via phenylhydrazine (PHZ) in terms of the antioxidant and apoptotic pathways and histopathologic changes in the colons of male albino rats. The rats were randomly divided into eight groups: control, AP extract, piceatannol (P), PHZ, PHZ and AP treatments, PHZ and P treatments, PHZ and both AP and P, and PHZ and prophylaxis with both AP and P. The results demonstrated that PHZ induced oxidative damage, apoptosis, and histopathological changes compared to the control group. However, the administration of AP or P or AP + P as therapy or prophylaxis significantly ameliorated these changes and upregulated the colonic mir-145 and mRNA expression of P53 and PDCD-4 while downregulating the colonic mRNA expression of PI3K, AKT, c-Myc, CK-20, SOX-2, OCT-4, and NanoG compared to the PHZ group. These findings suggest that the candidate drugs may exert their anti-cancer effects through multiple mechanisms, including antioxidant and apoptotic activities.

## 1. Introduction

Cancer, the uncontrolled growth of abnormal cells, can occur in any part of the body and can be classified based on the type of cell that initially undergoes transformation. The pathophysiology of cancer involves the disruption of normal cellular processes, leading to the uncontrolled growth and spread of abnormal cells to nearby tissues and distant organs as their main cause of death [1]. Colorectal cancer is the third most common cancer worldwide and the second leading cause of cancer-related deaths [2,3]. Oxidative stress, which results from an imbalance between reactive oxygen species (ROS) and antioxidant defenses, has been implicated in cancer development and progression [4]. ROS can cause damage to cellular components such as DNA, proteins, and lipids, leading to mutations and alterations that promote cancer development [5]. Additionally, ROS can activate signaling pathways that promote cell proliferation and survival and inhibit apoptosis. These enzymes, such as superoxide dismutase (SOD), catalase, and glutathione peroxidase, help to neutralize ROS and protect cells from damage [6]. However, in cancer, the expression and activities of antioxidant enzymes are often dysregulated, leading to an imbalance between the production of ROS and antioxidant defenses. Oxidative stress is a critical factor that contributes to the initiation and progression of the disease. Cancer cells often produce higher levels of ROS than normal cells, which can cause DNA damage and mutations that promote tumor growth [7].

Apoptosis, or programmed cell death, plays a critical role in maintaining tissue homeostasis and preventing the development of cancer [8]. The initiation of apoptosis involves two fundamental signaling pathways. The first of these is the extrinsic pathway, which is triggered by external stimuli and involves the activation of death receptors on the surfaces of cells when their pro-apoptotic ligands are bound. The extrinsic pathway typically also activates the intrinsic pathway, and both pathways result in the recruitment and activation of cysteine-aspartic acid proteases (caspases) [9]. Cytotoxic substances, DNA damage, a lack of growth factors, and oxidative stress are some of the intracellular stimuli that can activate the intrinsic (or mitochondrial) route [10], which is regulated by the Bcl-2 family of proteins and mitochondria [11] and characterized by the release of mitochondrial cytochrome c (cyt-c) [12]. Cytochrome c then leads to caspase-3 cleavage, endonuclease activation, and ultimately nuclear DNA fragmentation, which is the hallmark of apoptosis [13]. The Bax protein appears to be necessary for the proper induction of apoptosis, whereas the overexpression of the Bcl-2 protein is thought to reduce the likelihood of apoptosis [14]. The dysregulation of apoptosis can contribute to cancer development and progression by allowing the survival and proliferation of abnormal cells. Apoptosis can be triggered by a variety of signals, including oxidative stress, DNA damage, and the activation of death receptors. Therapeutic strategies that target apoptotic pathways are being developed as potential treatments for cancer.

The tumor suppressor gene TP53, a component of the mitochondrial apoptotic pathway, is a key regulator of cell cycle control and apoptosis. P53 mutations in tumor cells impair the ability to trigger apoptosis. Its expression is limited and downregulated [15]. Several molecular pathways are involved in cancer growth and metastasis; PI3K/AKT pathway activation has been recorded to improve tumorigenesis, metastasis, the epithelium–mesenchymal transition, and the drug resistance of cancer cells [16] via upregulating the expression of c-MYC, which increases the growth, differentiation, invasion, and chemotherapy drug resistance of cancer cells [17]. c-MYC is a key regulator of cell growth and differentiation, and in normal cells, its level of expression is very low. On the contrary, in cancer cells, it displays a sharp upregulation in it is expression subsequent to the activation of the PI3K/AKT pathway [17]. On the other hand, the upstream regulation of c-MYC negatively affects the expression of the tumor suppressor gene P53, promoting cancer cell proliferation and invasion [18]. On the same basis, programmed cell death 4 (PDCD-4) is one of the tumor suppressor genes that halts cancer cells’ growth and metastasis and is often reported to be downregulated in many cancer types that lead to tumor progression, promotion, and proliferation [19]. Thereby, the above-mentioned signaling pathways play critical roles in cancer cell progression or apoptosis. Reasonably, the suppression of the PI3K/AKT pathway could potentially inhibit c-MYC activation and upregulate the expression of the tumor suppressor genes (P53 and PDCD-4) [20].

Several microRNA molecules control the expression of the PI3K/AKT/c-MYC/P53-PDCD-4 signaling pathway; these types of regulation include the activation or suppression of the PI3K/AKT pathway [21]. MicroRNAs (miRNAs) are small, non-coding RNA molecules that play important roles in gene regulation. The dysregulation of miRNA expression has been implicated in the development and progression of many diseases, including cancer [22]. In cancer, altered miRNA expression can contribute to oncogenesis by promoting cell proliferation, inhibiting apoptosis, and enhancing angiogenesis and metastasis [23]. However, miRNAs can also act as tumor suppressors by targeting oncogenes or by regulating cell differentiation and senescence [22,23]. The discovery of the role of miRNAs in cancer has led to growing interest in their potential as therapeutic targets. The ability of miRNAs to modulate multiple genes in a coordinated manner makes them attractive targets for cancer therapy [22,23]. miRNA-based therapeutics can be designed to restore the expression of tumor suppressor miRNAs or inhibit the expression of oncogenic miRNAs. In addition, miRNAs can be used as biomarkers for cancer diagnosis, prognosis, and predicting therapeutic response. Among these microRNAs is mir-145, which is considered a tumor suppressor microRNA that acts via the downstream regulation of the PI3K/AKT/c-MYC pathway, hence improving the expression of PDCD-4 and P53, which halt cancer progression and proliferation [24].

Several stemnesses and proliferating markers have recently been implicated in the scoring of cancer cells’ activity and progression, such as Sox2, Nanog, OCT3/4, and ki67, which reflect the proliferating potency and aggressiveness of cancer cells as higher levels of expression of these markers indicate a bad prognosis and higher degrees of cancer invasion and metastasis [25]. Sox2, Nanog, and OCT3/4 are embryonic transcriptional factors [26]. Also, the Ki-67 protein is a popular proliferation marker for human tumor cells [27]. It exists in each of the cell cycle’s active phases (G1, S, G2, and M) but not in the resting phase (G0) [28]. It participates in the progression of the cell cycle in both interphase and mitotic cells [29]. As an indicator of tumor aggressiveness, the expression of the Ki-67 protein (pKi67) is linked to the proliferative activities of intrinsic cell populations in malignant tumors [30]. Carcinogens seem to play a possible role in causing molecular changes in oncogenes or suppressor genes that will cause the cells to proliferate more than their normal borders.

Despite advances in cancer treatments, the side effects and limited efficacy of conventional therapies have led to increased interest in alternative and complementary approaches. Piceatannol (P), a naturally occurring polyphenol found in various plant sources such as grapes, passion fruit, and white tea, has 11 times the scavenging activity against peroxyl radicals than its parent resveratrol and enhanced the protective effect against DNA damage brought on by •OH radicals [31]. It is effective at scavenging lipid peroxyl radicals [32]. It has a proapoptotic impact on cancer cells. It has been shown to inhibit the growth and proliferation of colon cancer cells by inducing apoptosis (programmed cell death) and cell cycle arrest. Piceatannol has also been shown to suppress the activity of various signaling pathways involved in cancer cell survival and growth, such as the PI3K/Akt and Wnt/β-catenin pathways. Its proapoptotic action might be attributed to mitochondrial potential loss, cytochrome C release, and caspase activation [33]. Recently, its anticancer properties were proven, which operate via targeting tumor-associated macrophages and TGF-β/apoptosis-signaling pathways that limit cancer growth and metastasis, especially in colon and colorectal cancers [34,35,36].

One such approach is the use of plant extracts, which have been used for centuries in traditional medicine to treat a variety of ailments [37]. Plant extracts contain a diverse array of bioactive compounds, including polyphenols, flavonoids, alkaloids, and terpenoids, which have been shown to have anticancer properties [31,32,33]. These compounds can target various molecular pathways involved in cancer development and progression, including cell proliferation, apoptosis, angiogenesis, and metastasis [38,39]. Several plant extracts have shown potential as anticancer agents in preclinical studies and clinical trials. For example, extracts from turmeric (Curcuma longa), green tea (Camellia sinensis), and ginseng (Panax ginseng) have been shown to have anticancer effects in vitro and in vivo and have been the subjects of clinical trials in cancer patients [40]. Other plant extracts, such as those from garlic (Allium sativum), ginger (Zingiber officinale), and milk thistle (Silybum marianum), have also been shown to have anticancer properties [41].

The genus *Adiantum pedatum *L. belongs to the Pteridaceae family and is a delicate and elegant fern species often known as the northern maidenhair fern. Traditional medicine has regularly used *Adiantum pedatum* for its numerous health advantages. It was previously used to treat several afflictions like sore throat, kidney stones, and hepatic insufficiency and to control wound bleeding [42]. Some pteridophytes have been shown to have anticancer properties, but not against a specific kind of cancer. *Asplenium rutamuraria* has phenolic compounds as anticancer agents [43]. Meanwhile, Pteridium aquilinum’s extract is a potent source of anticancer compounds, and Equisetum hyemale induced G2/M arrest and cell apoptosis. However, the anticancer effects of this plant and its bioactive compounds still have not been investigated. Moreover, in-depth studies are needed to completely understand the mechanisms underlying the *Adiantum pedatum* extract’s anti-cancer activities and to evaluate its potential therapeutic uses.

Thus, the present study was designed to investigate the antiproliferative activity of an Adiantum pedatum extract against chemically induced colon cancer in rats and address the implication of the mir-145/PI3K/AKT/c-MYC/PDCD-4—P53 signaling pathway in either the curative or prophylactic effect of the extract in comparison to a recently validated anticancer bioactive compound, piceatannol. This study also investigated the possible therapeutic synergism of combining the AP extract with piceatannol against rat colon cancer.

## 2. Results

### 2.1. Phytochemical Screening of AP Extract

**Figure 1:**
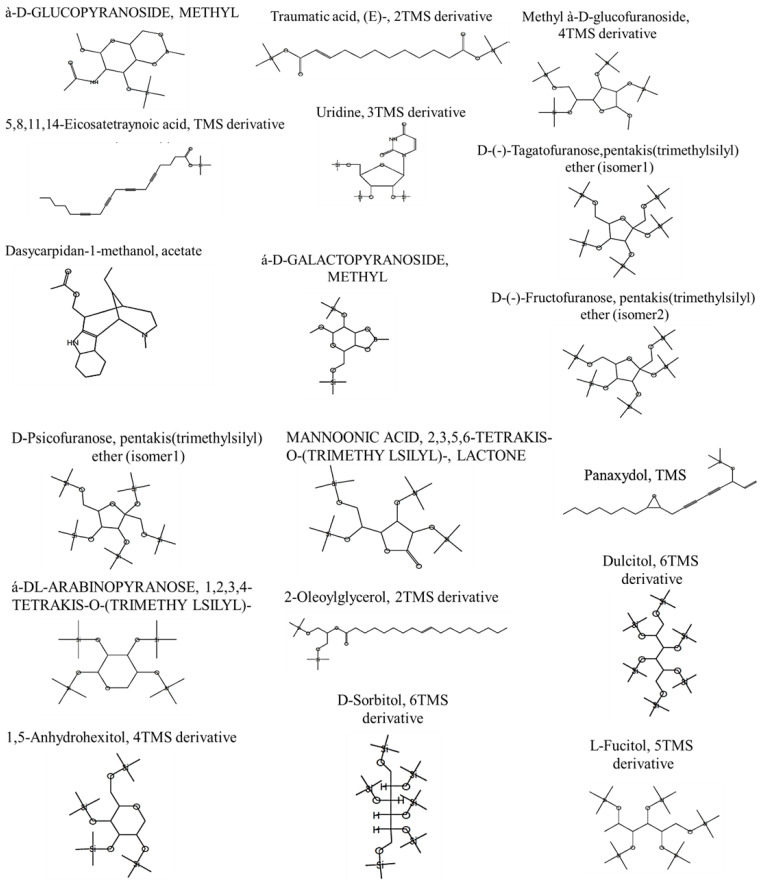
Chemical structure of the GC-MASS-screened Adiantum pedatum (AP) extract’s bioactive compounds. In gas chromatography–mass spectrometry (GC–MS), compound identification is currently achieved by comparing a query mass spectrum with reference mass spectra in a library, the NIST/EPA/NIH Mass Spectral Library (Main EI MS Library(mainlib) and replib), via spectrum matching.

The run time of a GC-Mass screening of the AP extract was 45 min. The GC-Mass phytochemical screening of the AP extract was carried out with the use of the National Institute Standard and Technology (NIST)’s mass spectral library (National Institute of Standards and Technology, Gaithersburg, MD, USA) [44,45]. The screened unknown compounds’ spectra were matched to the NIST-recorded known spectra, revealing 20 bioactive chemicals (Table 1 and Figure 1).

### 2.2. Oxidative Stress Markers

As shown in Table 2, for malondialdehyde (MDA), protein carbonyl (PC), and nitric oxide (NO) levels, the administration of AP or P did not induce any significant change in colon tissue when compared with the control group. On the other hand, the PHZ group showed a significant increase in MDA, PC, and NO levels when compared with the control group, while the PHZ + AP, PHZ + PIC, PHZ + AP + P, and prophylaxis groups showed significantly reduced levels of oxidative stress markers when compared with the PHZ group.

### 2.3. Antioxidant Markers

As shown in Table 3, the administration of AP and P did not induce any significant change in GSH, SOD, CAT, GPx, and TAC levels when compared with the control group, but AP administration induced a significant increase in the GST level when compared with the control group. The PHZ group showed significant decreases in all antioxidant levels when compared with the control group. The PHZ + AP, PHZ + PIC, PHZ + AP + P, and prophylaxis groups showed significantly increased levels of GSH, GST, SOD, CAT, GPx, and TAC when compared with the PHZ group.

### 2.4. mir-145 and mRNA Expression of PI3K, AKT, P53, c-Myc, and PDCD-4

**Figure 2:**
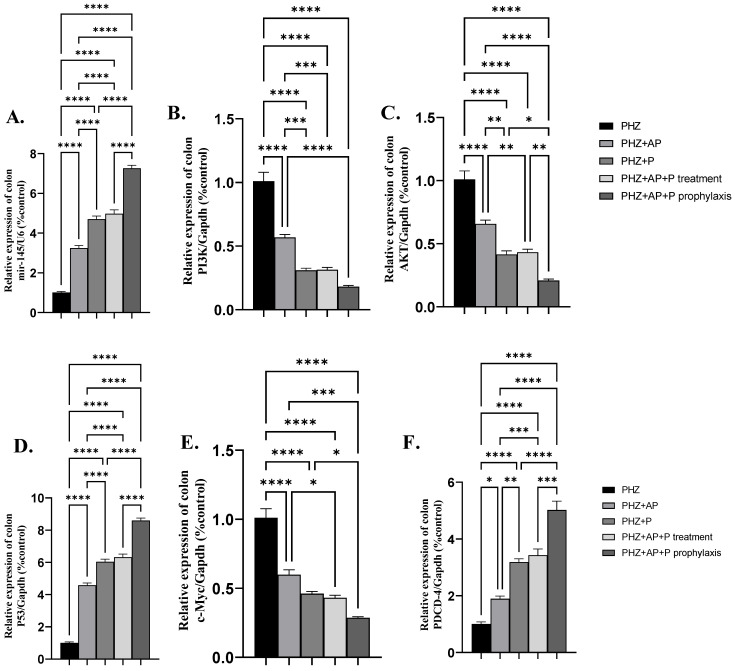
Effects of different treatments on mir-145 and mRNA expression of PI3K, AKT, P53, c-Myc and PDCD-4 (**A**–**F**); (**A**) mir-145, (**B**) mRNA expression of PI3K, (**C**) mRNA expression of AKT, (**D**) mRNA expression of P53, (**E**) mRNA expression of c-Myc, and (**F**) mRNA expression of PDCD-4. Data are expressed as means ± SEMs. *, **, *** and **** indicate significant differences (*p* < 0.05, *p* < 0.01, *p* < 0.001, and *p* < 0.0001).

PHZ + AP, PHZ + P, PHZ + AP + P, and prophylaxis significantly upregulated the colonic mir-145 and mRNA expression of P53 and PDCD-4 compared to the administration of PHZ to rats (Figure 2A,D,F). The administration of AP or P or AP + P as therapy or prophylaxis in PHZ-induced colorectal cancer significantly downregulated the colonic mRNA expression levels of PI3K, AKT, and c-Myc compared to the PHZ group (Figure 2B,C,E).

### 2.5. Apoptotic and Antiapoptotic Proteins

**Figure 3:**
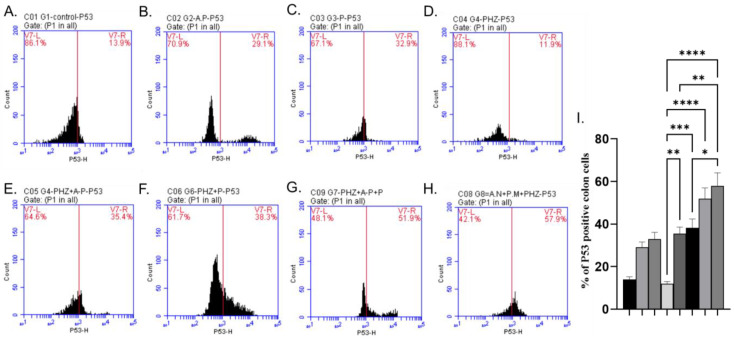
Effects of different treatments on P53% cells, determined using flow cytometric analysis (**A**–**I**). (**A**) Control group, (**B**) AP group, (**C**) P group, (**D**) PHZ group, (**E**) PHZ + AP group, (**F**) PHZ + P group, (**G**) PHZ + AP + P group, (**H**) prophylaxis group, and (**I**) % of P53 positive colon cells. FLI-H: detector of P53 of flurosenceisothiocyanate (FITC) fluorochrome. Count: % of count cells labeled with anti-P53. *, **, *** and **** indicate significant differences (*p* < 0.05, *p* < 0.01, *p* < 0.001, and *p* < 0.0001).

**Figure 4:**
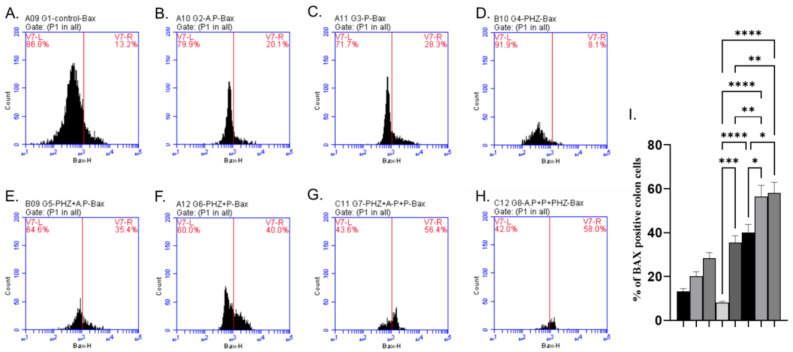
Effects of different treatments on Bax%, determined using flow cytometric analysis (**A**–**I**). (**A**) Control group, (**B**) AP group, (**C**) P group, (**D**) PHZ group, (**E**) PHZ + AP group, (**F**) PHZ + P group, (**G**) PHZ + AP + P group, (**H**) prophylaxis group, and (**I**) % of Bax positive colon cells. FLI-H: detector of Bax of flurosenceisothiocyanate (FITC) fluorochrome. Count: % of count cells labeled with anti-Bax. *, **, *** and **** indicate significant differences (*p* < 0.05, *p* < 0.01, *p* < 0.001, and *p* < 0.0001).

**Figure 5:**
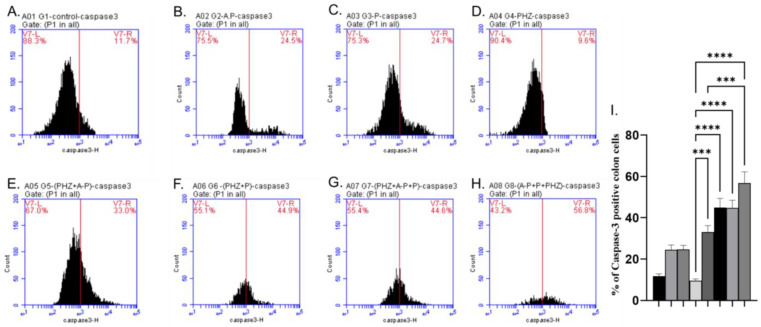
Effects of different treatments on caspase 3%, determined using flow cytometric analysis (**A**–**I**). (**A**) Control group, (**B**) AP group, (**C**) P group, (**D**) PHZ group, (**E**) PHZ + AP group, (**F**) PHZ + P group, (**G**) PHZ + AP + P group, (**H**) prophylaxis group, and (**I**) % of Caspase3 positive colon cells. FLI-H: detector of caspase 3 of flurosenceisothiocyanate (FITC) flourchrome. Count: % of count cells labeled with anti-caspase 3. *** and **** indicate significant differences (*p* < 0.001, and *p* < 0.0001).

**Figure 6:**
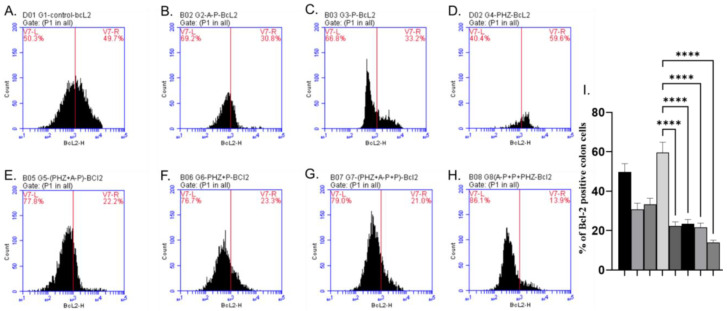
Effects of different treatments on Bcl-2%, determined using flow cytometric analysis (**A**–**I**). (**A**) Control group, (**B**) AP group, (**C**) P group, (**D**) PHZ group, (**E**) PHZ + AP group, (**F**) PHZ + P group, (**G**) PHZ + AP + P group, (**H**) prophylaxis group, and (**I**) % of Bcl2 positive colon cells. FLI-H: detector of Bcl-2 of flurosenceisothiocyanate (FITC) fluorochrome. Count: % of count cells labeled with anti-Bcl-2. **** indicate significant differences (*p* < 0.0001).

The AP and P groups showed significant increases in p53, Bax, and Caspase-3 levels compared to the control group, as shown in Figure 3A–C, Figure 4A–C and Figure 5A–C). The administration of PHZ induced significant decreases in p53, caspase 3, and Bax levels compared to the control group, as shown in Figure 3D, Figure 4D and Figure 5D). The PHZ + AP, PHZ + P, PHZ + AP + P, and prophylaxis groups showed significantly increased p53, caspase 3, and Bax levels compared to the PHZ group, as shown in Figure 3E–H, Figure 4E–H and Figure 5E–H). The AP and P groups showed significant decreases in Bcl-2 compared to the control group, as shown in Figure 6A–C. The administration of PHZ induced a significant increase in Bcl-2 level compared to the control group, as shown in Figure 6D. The PHZ + AP, PHZ + P, PHZ + AP + P, and prophylaxis groups showed significant decreases in Bcl-2 level compared to the PHZ group, as shown in Figure 6E–H.

### 2.6. Immunohistochemical Analysis of P53

**Figure 7:**
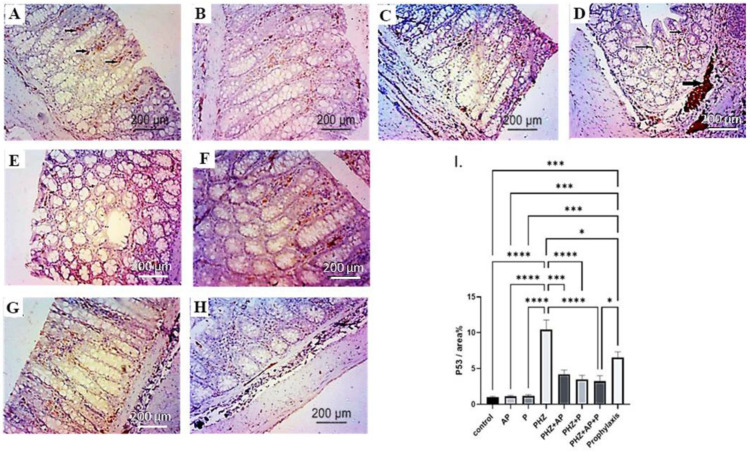
Effects of different treatments on immunohistochemical staining of P53, as shown via positive immunological reactions (arrowheads) of different investigated groups (**A**–**I**). (**A**) Photomicrograph of immunostained colon segment with P53; control group colon shows typical low nuclear P53. Scale bar: 200 μm, ×100. (**B**) Photomicrograph of immunostained colon segment with P53 in the AP group, demonstrating typical low nuclear P53 approximately identical to NC. Scale bar: 200 μm, ×100. (**C**) Photomicrograph of immunostained colon segment with P53 in the P group, revealing typical low nuclear P53 approximately identical to NC. Scale bar: 200 μm, ×100. (**D**) Photomicrograph of PHZ-induced colon cancer; inactive mutant P53 was strongly expressed in nuclear P53 immunopositivity. A photomicrograph of the PHZ + AP group immunostained with P53 showed reduced P53 expression compared to the PHZ group. Scale bar = 200 μm, ×100 (**E**) Scale bar = 50 μm, ×400. (**F**) Compared to PHZ, the PHZ + P group immunostained with P53 showed reduced P53 expression. Scale bar = 50 μm, ×400. (**G**) P53 expression was lower in the combined AP-P and PHZ immunostained photomicrographs than in the PHZ group. Scale bar: 50 μm, ×400. (**H**). Photomicrograph of combined AP-P and PHZ (prophylaxis) showed less P53 expression than the PHZ group. (**I**) P53 immunostaining intensity (% area), scale bar: 50 m, magnification: 400. The data are presented as means ± SEMs. *, *** and **** indicate significant differences (*p* < 0.05, *p* < 0.001, and *p* < 0.0001).

The control, AP, and P groups revealed characteristic low levels of nuclear P53 expression (Figure 7A–C,I). Figure 7D,I depict the robust nuclear expression of P53 in the PHZ-induced colon cancer group compared to the control group. Nevertheless, treatment with AP and P alone or in combination, as well as the prophylactic approach with PHZ, significantly reduced the expression of the P53 protein (Figure 7E–I).

### 2.7. mRNA Expression of CK-20, SOX-2, OCT-4 and NanoG

**Figure 8:**
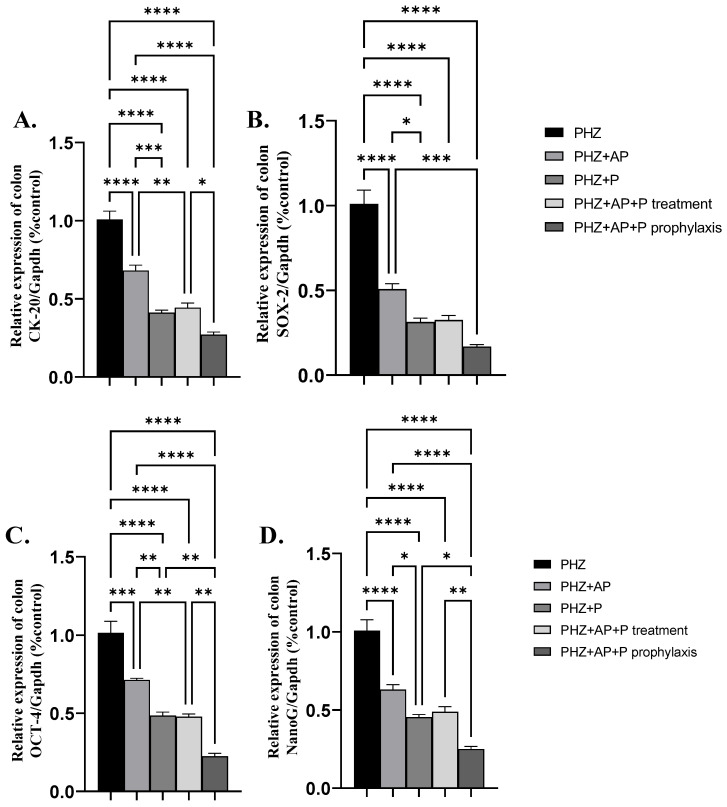
Effects of different treatments on mRNA expression of CK-20, SOX-2, OCT-4, and NanoG (**A**–**D**). (**A**) mRNA expression of CK-20, (**B**) mRNA expression of SOX-2, (**C**) mRNA expression of OCT-4, and (**D**) mRNA expression of NanoG. Data are expressed as means ± SEMs. *, **, *** and **** indicate significant differences (*p* < 0.05, *p* < 0.01, *p* < 0.001, and *p* < 0.0001).

The administration of AP or P or AP + P as therapy or prophylaxis in PHZ-induced colorectal cancer significantly downregulated the colonic mRNA expression of CK-20, SOX-2, OCT-4, and NanoG compared to the PHZ group (Figure 8A–D).

### 2.8. Immunohistochemical Analysis of Ki-67

**Figure 9:**
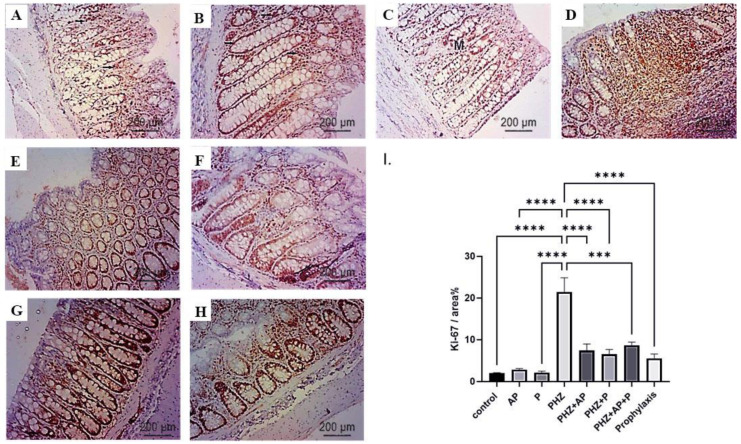
The effects of different treatments on the immunohistochemical staining of Ki-67, as indicated by positive immune reactions (arrowheads), in the various groups studied (**A**–**I**). (**A**) A photomicrograph of a colon section immunostained with Ki-67; the normal colon of the control group demonstrates normal low nuclear Ki-67 expression. Scale bar = 200 μm, ×100. (**B**) A photomicrograph of an immunostained colon section with Ki-67 in the AP group reveals a normal low nuclear level of Ki-67 almost identical to that of the NC group. Scale bar = 200 μm, ×100. (**C**) A photomicrograph of an immunostained colon section with Ki-67 in the P group reveals a normal low nuclear level of Ki-67 that is nearly identical to the NC group. Scale bar = 200 μm, ×100. (**D**) A microphotograph of colon carcinoma induced via PHZ with Ki-67 immunostaining, revealing nuclear Ki-67 immunopositivity with abundant expression of inactive mutants Ki-67. Scale bar = 200 μm, ×100, (**E**) A photomicrograph of the PHZ + AP group immunostained with Ki-67 exhibited a lower level of Ki-67 expression than the PHZ group. Scale bar = 50 μm, ×400. (**F**) A photomicrograph of the PHZ + P group immunostained with Ki-67 revealed a lower level of Ki-67 expression in comparison to the PHZ group. Scale bar = 50 μm, ×400. (**G**) A photomicrograph of the combination of AP-P and PHZ immunostained with Ki-67 revealed a lower level of Ki-67 expression compared to the PHZ group. Scale bar = 50 μm, ×400. (**H**) A photomicrograph of the combined AP-P and PHZ (prophylaxis) simultaneously revealed a lower level of Ki-67 expression compared to the PHZ group. Scale bar = 50 m, ×400. (**I**) Ki-67 immunostaining intensity (percent area). The data are presented as means ± SEMs. *** and **** indicate significant differences (*p* < 0.001, and *p* < 0.0001).

An immunohistochemical analysis of Ki-67 in the colons of various groups revealed distinct Ki-67 immunostaining patterns. The colon sections of the control, AP, and P groups exhibited moderate Ki-67 expression (Figure 9A–C,I). In contrast, a large number of strongly stained positive nuclei were observed in the colon sections of rats treated with PHZ, indicating a robust expression of the Ki-67 protein (Figure 9D,I). Nevertheless, treatment with AP and P alone or in combination, as well as the prophylactic approach, substantially decreased the expression of the Ki-67 protein compared to the PHZ group (Figure 9E–I).

### 2.9. Histopathological Results

**Figure 10:**
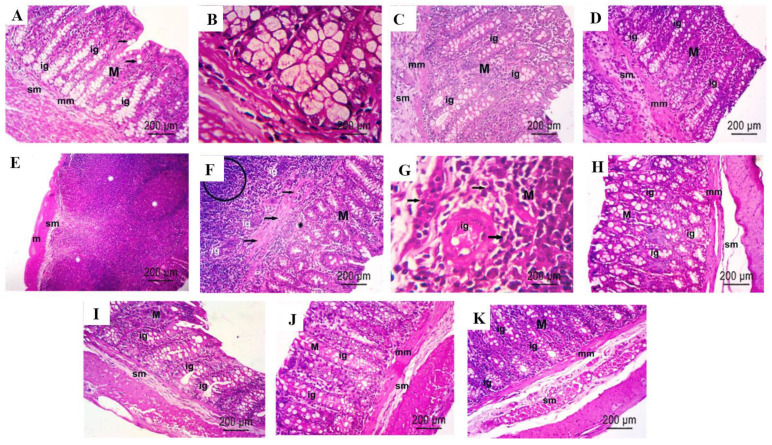
Histological characteristics of rat colons (**A**–**K**) in H&E-stained sections. (**A**,**B**) A photomicrograph of a colon cross-section stained with H&E (×400) shows a normal pattern of colon tissue, including a normal mucosa (M), intestinal glands (igs), goblet cells (arrows), muscularis mucosae (mm), and submucosa (sm). (**C**) A photomicrograph of a colon cross-section of a normal rat treated with AP stained with H&E (×400) shows normal intestinal glands in the mucosa, followed by a musclaris mucosae and submucosa comparable to the NC group. (**D**) A photomicrograph of a cross-section in the colon of a normal rat treated with P stained with H&E (×400) shows normal intestinal glands in the mucosa, followed by a musclaris mucosae and submucosa like the NC group. Notice the slightly disorganized intestinal glands (igs). (**E**) A photomicrograph of a colon specimen from the PHZ group stained with H&E (×200), showing significant dysplasia, with the nodular infiltration (*) of mononuclear cells of large sizes in the submucosa (sm) displacing the mucosa. M: muscularis. (**F**) PHZ colon specimen stained with H&E (×400), showing damaged intestinal glands (igs), nodular leucocytic infiltration (circle), disordered circular muscles (arrows), and mucosa (M) with necrotic zones (*). (**G**) A photomicrograph of a PHZ-treated colon cross-section stained with H&E (×400) showing intestinal gland dysplasia and damaged cells with hyperchromatic nuclei and apoptotic cells (arrows). (**H**) A photomicrograph of a PHZ + AP-treated colon cross-section stained with H&E (×400) shows decreased lymphocytic cell infiltration and intestinal gland dysplasia. (**I**) A photomicrograph of a PHZ + P-treated colon cross-section stained with H&E (×400) showed reduced crypt dysplasia and minimal lymphocytic cell infiltration, as well as an improved mucosa (M), intestinal glands (igs), and submucosa (sm). (**J**) A photomicrograph of a cross-section of a colon of the PHZ + AP + P-treated group stained with H&E (×400,) demonstrating a colonic structure remarkably similar to the normal control group, with a well-organized mucosa (M), intestinal glands (igs), muscularis mucosae (mm), and submucosa (sm). (**K**) A photomicrograph of a cross-section of a colon from the PHZ + AP + P-treated group stained with H&E (×400), showing a structured colonic architecture that is comparable to the normal control group.

The colons of the control rats displayed normal mucosae composed of regular arrangements of intestinal glands (Ig) or colonic crypts rich in goblet cells (arrows), secreting mucin and with normal basal nuclei; these highly glycosylated proteins are the major components of the mucous layer that lubricates and protects the gastrointestinal tract. The mucosa is also lined with columnar epithelia and has lamina propria between the intestinal glands. Figure 10A,B demonstrate that the intestinal crypts are followed by muscularis mucosae (mm) and then submucosae (sm). The rats administered the AP extract exhibited normal mucosae with normally arranged intestinal glands, followed by the mm and sm, Figure 10C. The colon of a normal rat treated with P demonstrated a normal mucosa (M) with normally arranged intestinal glands, followed by a thin muscularis mucosa (mm) and submucosa (sm), and as demonstrated, the intestinal glands descended deeply into the submucosa, Figure 10D. A PHZ-treated rat colon displayed the mucosa, submucosa, and muscle layers. The mucosa exhibited marked dysplasia and an abnormal and irregular arrangement of intestinal glands (Ig) with hyperchromatic nuclei, some of which shared cell walls; the architecture was severely disrupted, and necrotic areas were also expanded. Necrosis could be observed in a number of malignant neoplastic glands. The muscularis mucosae was disrupted and disorganized, and intestinal glands diffused into the submucosa, generating tumor cell clusters associated with leucocytic cell infiltration (Figure 10E–G). The colon of a diseased animal revived with AP demonstrated a decrease in dysplastic changes within the intestinal glands, a marked improvement in the histopathological examination via H&E stains, and an improvement in the architecture of the crypts, which were lined with a columnar epithelium with minor size variations. The muscularis mucosae was restored to a thin layer, and there were fewer leucocytic cells in the submucosa, Figure 10H. The colons of diseased animals cured via P demonstrated decreases in intestinal dysplasia alterations (Figure 10I). Figure 10J demonstrates that the architecture of the crypts in the colon of a diseased animal revived with AP and P was significantly improved. The colons of diseased animals revived with AP and P (prophylaxis) demonstrated mitigations of the deleterious effects of PHZ, successfully suppressed tumor progression, and marked improvements in tissue sections, which may be attributed to the synergistic suppression effect of AP when combined with P (Figure 10K).

## 3. Discussion

Colorectal cancer (CRC) is a major cause of cancer-related deaths worldwide. Moreover, several epidemiological studies have linked the consumption of red meat with an increased risk of CRC [2,66]. For example, a meta-analysis showed that the consumption of red meat was associated with a 12% increase in the risk of CRC per 100 g per day of consumption [66]. Moreover, a study showed that the consumption of red meat was associated with an increased risk of CRC in both men and women [67]. Several potential mechanisms have been proposed to explain the link between red meat consumption and the CRC risk [66]. One such mechanism is the production of carcinogenic compounds during the cooking process. When meat is cooked at high temperatures, such as through grilling or frying, it can produce heterocyclic amines (HCAs) and polycyclic aromatic hydrocarbons (PAHs), which are known carcinogens [68]. These compounds have been shown to induce mutations in key oncogenes and tumor suppressor genes, leading to the development of CRC. Another potential mechanism underlying the link between red meat consumption and CRC risk is meat’s high content of heme iron [69]. It has been suggested that a high intake of heme iron may promote the development and progression of CRC through a variety of mechanisms, including the production of reactive oxygen species and the upregulation of pro-inflammatory cytokines [70]. Further research is needed to better understand the complex molecular mechanisms underlying the development and progression of CRC, as well as the potential link between red meat consumption and the risk of CRC. This knowledge can be used to develop more effective prevention and treatment strategies for CRC. In the meantime, individuals can reduce their risk of developing CRC by adopting a healthy lifestyle, including a balanced diet that is rich in fruits and vegetables and low in red and processed meats, exercising regularly, and avoiding smoking and excessive alcohol consumption.

A variety of plants and their bioactive substances have anti-carcinogenic and anti-proliferative effects on colon cancer cells [71]. Studies have also shown a positive correlation between plants’ antioxidant activities and their anti-proliferative properties, indicating that antioxidants may act to slow the growth of cancer cells. For instance, flavonoids exhibit a variety of biological properties, such as cytoprotective properties, and several of them are known to have anti-cancer properties [72]. The present study evaluates the protective roles of both AP and P on colon cancer induced via PHZ. In this study, using PHZ caused elevations in MDA, NO, and PC levels as oxidative stress markers, while MDA, NO, and PC levels significantly decreased in the AP and P with PHZ groups. Also, PHZ caused a decline in all antioxidant levels (SOD, CAT, GST, GSH, and TAC); on the other hand, groups treated with AP and P and PHZ showed significant increases in all antioxidant levels. PHZ intoxication in rats can lead to DNA fragmentation, oxidative DNA damage, and the development of tumors [73]. PHZ causes oxidative stress, which raises ROS such as MDA, which is a lipid peroxidation metabolite; it also lowers antioxidant status [74]. It increased PC and reduced GSH [75]. Plasma PC levels have been associated with the etiology of colorectal cancer [76]. The administration of PHZ led equally to significant reductions in the activities of the enzymes SOD, CAT, and GPx [77]. In the present study, PHZ caused a significant increase in reactive radical NO levels, and these results are in line with Aloke et al. [78], who showed that PHZ caused significant increases in MDA and NO levels as oxidative markers and significant decreases in SOD, CAT, and GSH as antioxidant markers. The excess production of NO causes DNA damage and inhibits DNA repair proteins [79]. Similarly, the levels of NO have been shown to be higher in CRC patients compared to healthy controls [80]. P is a resveratrol metabolite that is regarded as a powerful antioxidant and cytoprotectant due to its significant ability to suppress ROS [81]. P significantly increased the levels of the antioxidants SOD and CAT, it reduced the amounts of the lipid peroxides LPO and NO, which are indicators of mitochondrial oxidative stress [82], and it caused a significant induction of the total antioxidant capacity TAC [81]. Thus, the anti-oxidative property of P may be involved in its anti-invasive action [83]. P inhibits the production of ROS and reduces oxidative stress, exhibiting anti-oxidant and anti-inflammatory effects [84]. P significantly increased GSH levels and decreased lipid peroxidation, according to Wahdan et al. [85]. Interestingly, the antioxidant and anti-cancer activities of the AP extract could be attributed to the bioactive compounds which were identified via GC-MASS spectra and investigated previously for their antioxidant and anticancer activities, such as Methyl α-D-glucopyranoside [46,47], 5,8,11,14-Eicosatetraynoic acid TMS derivative [48], Dasycarpidan-1-methanol, acetate [49], Traumatic acid, (E)-, 2TMS derivative [50], Panaxydol, TMS [51], 2-Oleoylglycerol, 2TMS derivative [52], alpha-D-Mannopyranoside, methyl 2,3,5,6-tetrakis-O-(trimethylsilyl) [53], Uridine, 3TMS derivative [54], Methyl alpha-D-galactopyranoside [55], Methyl à-D-glucofuranoside, 4TMS derivative [56], D-(-)-Tagatofuranose,pentakis (trimethylsilyl) ether [57], D-(-)-Fructofuranose, pentakis(trimethylsilyl) ether [58], D-Psicofuranose, pentakis(trimethylsilyl) ether [58], 1,5-Anhydrohexitol, 4TMS derivative [59], α- DL- Arabinopyranose, 1,2,3,4- tetrakis-O-(trimethylsilyl) [60], Mannoonic acid, 2,3,5,6-tetrakis-O-(trimethylsilyl)-, lactone [61], Dulcitol, 6TMS derivative [62], D-Sorbitol, 6TMS derivative [63], Butanal, 2,3,4-tris[(trimethylsilyl)oxy]-3-[[(trimethylsilyl)oxy]methyl]-[64], L-Fucitol, and 5TMS derivative [65].

The molecular mechanisms underlying CRC development and progression are numerous and complex [86]. CRC is a complex disease that involves the accumulation of genetic and epigenetic alterations in key oncogenes and tumor suppressor genes [86,87]. These alterations lead to the dysregulation of several signaling pathways, including the Wnt/β-catenin, PI3K/Akt/mTOR, and MAPK/ERK pathways, among others [88]. These pathways regulate a wide range of cellular processes, including cell proliferation, differentiation, and apoptosis. The dysregulation of these pathways can lead to uncontrolled cell growth and the development of CRC. One such mechanism is the Nrf2 antioxidant response, which plays a key role in protecting cells from oxidative stress and other forms of cellular damage [89]. Nrf2 is a transcription factor that plays a key role in protecting cells from oxidative stress and other forms of cellular damage. Under normal conditions, Nrf2 is sequestered in the cytoplasm by the protein Keap1 [90]. However, when cells are exposed to oxidative stress or other forms of cellular damage, Nrf2 is released from Keap1 and translocates to the nucleus, where it activates the expression of antioxidant enzymes and other cytoprotective proteins. The Nrf2 antioxidant response has been shown to play a key role in protecting cells from oxidative damage and promoting cell survival. However, recent studies have suggested that the Nrf2 antioxidant response may also play a role in CRC development and progression [91,92]. Several studies have suggested that the Nrf2 antioxidant response may be involved in CRC development and progression. For example, a study showed that Nrf2 activation promotes the growth and survival of CRC cells [93]. Another study showed that Nrf2 activation may promote CRC metastasis [91]. MicroRNAs (miRNAs) are a large family of short (19–22 nucleotide), endogenous RNAs that adversely affect the expression of target genes by cleaving mRNA or via translation inhibition [94]; depending on the target, they can operate as oncogenes or tumor suppressors. miR-145 is a p53-regulated tumor suppressor that has been discovered to be downregulated in colorectal cancer and other malignancies [95,96]. It modulates cancer stem cell stemness and pluripotency by targeting many embryonic transcriptional factors that enhance the oncogenic activity of metastatic cancers, including OCT4, SOX2, NanoG, and KLF4 [95]. miR-145 is a p53-controlled gene. In response to DNA damage, p53 can stimulate transcription and improve the post-transcriptional maturation of the miR-143/miR-145 cluster [96,97] via interacting with the Drosha processing complex [98]. The Ras-responsive element-binding protein (RREB1), which represses the miR-143/145 promoter, can inhibit miR-143/145 cluster transcription [99]. In our study, PHZ + AP, PHZ + P, PHZ + AP + P, and prophylaxis significantly upregulated the colonic mir-145 and mRNA expression levels of P53 and PDCD-4 compared to what was observed in the PHZ-administered rats. The administration of AP or P or AP + P as therapy or prophylaxis in PHZ-induced colorectal cancer significantly downregulated the colonic mRNA expression of PI3K, AKT, c-Myc, CK-20, SOX-2, OCT-4, and NanoG compared to the PHZ group. Hatley and colleagues found that miR-21 promotes RAS signaling activity and thereby suppresses the miR-143/145 cluster [100]. MiR-145 was found to be significantly downregulated in a variety of tumors, including pancreatic, breast, colon, and prostatic cancers [95,96]. Indeed, miR-145 has been well established as a tumor suppressor gene due to its negative regulation of various oncogenes such as Myc, K-Ras, IRS-1, and ERK5 [96,101]. Furthermore, miR-145 inhibits breast cancer cell motility and invasiveness by negatively regulating the junctional cell adhesion molecule (JAM-A), fascin and MUC1 [102,103]. By targeting the oncogenic FLI1, miR-145 suppresses the growth of colon cancer cells and sensitizes them to 5-fluorouracil [104].

Plants produce polyphenols as secondary metabolites to defend themselves from stressful conditions like excessive ultraviolet (UV) irradiation, heat exposure, insect attacks, and bacterial or fungal infections. AP’s high concentration of polyphenolic compounds like terpenoids, cardiac glycosides, steroids, and phenols may be the cause of its antioxidant activity. These findings show promising potential for the development of antimicrobial and antioxidant drugs from the AP plant and provide scientific evidence to support its traditional uses [105]. Acetone extracts and ethanol extracts have high tannin contents [105]. Tannins are antioxidants often characterized by their reducing power and scavenging activities [106]. In the present study, we investigated the expression levels of of p53, caspase3, Bax, and Bcl-2 using the flow cytometric technique to understand the signaling mechanism related to the apoptotic effects of AP and P against colorectal cancer induced via PHZ. In this study, the use of PHZ caused decreases in p53, caspase3, and Bax, and elevations in Bcl2 levels. On the other hand, using AP and P with PHZ increased the levels of p53, caspase3, and Bax and decreased Bcl2 levels. The induction of apoptosis is a major cytotoxic mechanism of anticancer therapies, including radiation, chemotherapy, and targeted therapies [107]. Cancer cells can develop various mechanisms to evade apoptosis. Deficiency in p53, Bax, or caspase activation can cause resistance to radiation and chemotherapy, while the overexpression of anti-apoptotic proteins such as Bcl-XL, c-FLIP, and IAPs are frequently associated with therapeutic resistance [108]. Bcl-2 is a protein best known for its roles in inhibiting apoptosis and promoting oncogenesis [109], and it is widely believed to be an apoptosis suppressor gene. The overexpression of the protein in cancer cells may block or delay the onset of apoptosis by selecting and maintaining long-living cells and arresting cells in the G0 phase of the cell cycle [110]. According to NaveenKumar et al. [111], the use of PHZ decreased p53, Bax, and Caspase-3 levels and increased Bcl-2 in hyperbilirubinemia rats.

Many anticancer drugs act along the physiological pathways of apoptosis, leading to tumor cell destruction [71]. The pro-apoptotic effects of p are mediated through the induction of apoptosis via increases in p53 levels, the upregulation of Bax, the activation of caspases -3, the downregulation of Bcl-2, the loss of mitochondrial potential, and the release of cytochrome c. P has been shown to induce apoptosis in cancer cells [112]. The regulation of apoptotic responses and decreases in oxidative stress, and inflammation by P were linked to a significant amelioration of mitochondrial function [82]. The tumor necrosis factor-related apoptosis-inducing ligand (TRAIL) is a pro-apoptotic ligand that activates the extrinsic apoptosis pathway of cell death receptors; TRAIL could inhibit metastasis and colon cancer cell invasion by promoting platelet apoptosis [113]. P substantially enhances TRAIL-induced cell death (apoptosis), including DNA fragmentation, in human leukemia [114]. Also, in this study, AP succeeded in increasing the apoptotic proteins and decreasing the Bcl-2 level, so it can be concluded that it can inhibit cell proliferation and cause the induction of apoptosis.

The histopathological features may be observed in the biochemical, immunohistochemical, genetic, and epigenetic elements found in the colonic mucosa [115]. For immunohistochemical studies, in the present study, PHZ showed high positivity for ki67 and p53 in colon cancer tissue. According to Darwish et al. [116]’s results, high immunohistochemical nuclear positivities for ki67 and p53 in a Wilms tumor were shown. According to Nussrat, et al. [117], the high-grade dysplasia of colorectal cancer showed significant positive immunohistochemical markers of Ki-67 and P53. The positively stained P53 in the mucosal cells may have appeared as a result of inactive mutants of P53. This result agrees with Hahn and Weinberg [118], who reported that the P53 gene is mutated and deleted in approximately one-half of colorectal tumors, leading to the inactivation of the P53 protein. On the other hand, using P and the AP extract caused lower levels of expression of the Ki-67 and P53 proteins compared with the PHZ group. According to Lee et al. [119], immunohistochemistry staining showed a reduction in the expression of Ki-67 in human oral cancer cells in a P-treated group, and this effect was due to its growth inhibitory effects and the induction of apoptosis.

## 4. Materials and Methods

### 4.1. Chemicals

Piceatannol ((E)-4-[2-(3,5Dihydroxyphenyl)ethenyl]1,2-benzenediol, 3,3′,4,5′-Stilbenetetrol, 3,3′,4,5′-Tetrahydroxy-trans-stilbene, Astringenin, and phenylhydrazine were purchased from Sigma–Aldrich Chemical Co. (St. Louis, MO, USA).

### 4.2. Preparation, Phytochemical Screening, and Physicochemical Standardization of the Adiantum pedatum (AP) Extract

An *Adiantum pedatum* plant was collected on 15 June 2021 at the Faculty of Pharmacy of Zagazig University and was authenticated under a voucher number (DBCU06) of the herbarium at the Department of Botany, Cairo University. The plant was cleaned with deionized water, and the plant sample was dried at room temperature for 7 days; then, it was ground into a powder and stored in air-tight containers at 4 °C. For the extraction of the plant material, 1 kg of dried plant powder was taken in a conical flask and subjected to a maceration extraction at room temperature, using absolute ethanol three times (3 L). The extract was collected in a separate conical flask to evaporate the solvent via natural evaporation for 2 days. The remaining extract weighed around 230 g. The plant phytochemical screening was carried out using gas chromatography–mass spectrometry (GC-Mass) [120], using a direct capillary column TG-5MS (30 m × 0.25 mm × 0.25 μm thickness). Approximately 3 L of the AP extract was automatically injected into the equipment using an Auto sampler AS3000 combined with GC in split mode and the report attached in the Supplementary Materials. The instrumental analysis was then performed as previously reported [121]. The components were identified by comparing their retention times and mass spectra to the databases Wiley 09 and NIST 11 [121]. Assays for the AP moisture content%, pH, cold extract%, hot extract%, ash (total, acid insoluble, and water-soluble ash%), and total phenolic compound% and tests for saponins, tannin, flavonoids, and proteins were performed according to the method described in [122], as shown in Table 4.

### 4.3. Animals Care

The animal house of the college of Veterinary Medicine at Zagazig University provided 48 healthy adult male albino rats, each weighing between 180 and 200 g. Throughout the experiment, the animals were housed in standard laboratory settings. All animal procedures were carried out in compliance with the accepted standards for handling and using lab animals. All rats received humane care, and the experimental methods were approved by the Institutional Animal Care and Use Committee of Badr University in Cairo (No. BUC-IACUC/VET/131/A/2023).

### 4.4. Induction of Colon Cancer

The animals were each given a weekly dose of PHZ that was dissolved in 0.9% NaCl and injected intraperitoneally at a dose of (90 mg/kg bw) twice a week for four consecutive weeks to induce colon cancer [123,124], and the PHZ injections continued for the next four weeks.

### 4.5. Experimental Animals Design

The rats were randomly divided into eight groups (15 in each group), and they were administered their doses as follows: group 1 (control group) contained rats fed on a balanced diet without any treatments; the group 2 (AP) rats were administered a dose of 200 mg/kg b.w of AP orally for each alternate day [125]; the group 3 (P) rats were administered a dose of 40 mg/kg b.w. of p orally for each alternate day [126], the group 4 (PHZ) rats were administered a dose of 90 mg/kg b.w. of a colon cancer drug injected twice a week ip [124]; the group 5 (PHZ + AP) rats were administered PHZ and AP treatments in the same previous doses; the group 6 (PHZ + P) rats were administered PHZ and P treatments with the same previous doses; the group 7 (PHZ + AP + P) rats were injected with PHZ and co-administered with both AP and P in the same previous doses; and the group 8 (prophylaxis) (PHZ + AP + P) rats were injected with PHZ and co-administered with both AP and P at the same previous doses all at once from the initiation of the experiment. All treatments were applied regularly under the same conditions for a period of 8 weeks.

### 4.6. Preparation of Colon Tissue Homogenate for Biochemistry Analysis

Before autopsy, the tissues were washed with a PBS (phosphate-buffered saline) solution at a pH of 7.4 which contained 0.16 mg/mL heparin. The tissues were then homogenized in 5–10 mL of cold buffer to remove any red blood cells and centrifuged at 4000 rpm for 15 min at 4 °C. The supernatant was removed for an assay and stored on ice, frozen at −80 °C until use. 

### 4.7. Determination of Oxidative Stress and Antioxidant Markers

All markers we measured were assayed using clear supernatants of colon tissue homogenate. These assays included oxidative stress products. Lipid peroxidation (LPO) was measured by assaying the end product of a peroxidation reaction of MDA, and the result was expressed as (nmol/g wet tissue) [127]. The measurement of protein carbonyl (PC) was carried out according to [128] and expressed as (nmol/g). The measurement of nitric oxide (NO) was carried out according to [129] and expressed as (µmol/g wet tissue). Regarding antioxidant enzymes, the total antioxidant capacity (TAC) was determined according to [130] and expressed as mM/g. CAT was expressed as a unit per gram tissue (U/g) [131]. SOD was expressed as (U/g) [132]. GSH was expressed as (mmol/g) [133]. GST was expressed as (U/g tissue) [134]. The GPx level was expressed as (U/gHb) according to Weinhold et al., 1990. All analyses were performed using a semi–Auto Biochemistry Analyzer (Robonik, Maharashtra, India).

### 4.8. Preparation of Colon Tissue Suspension for Flow Cytometry

Fresh colon tissue specimens were used in isotonic saline 0.9% and prepared using isotone tris EDTA buffer to wash the materials. The prepared materials were dissolved in 250 mL of distilled water, and then 1N HCL was used to adjust the pH to 7.5. The supernatant was separated from the cell suspension after centrifugation at 1800 rpm for 10 min and examined with a microscope for blood contamination. The cells were fixed in ice-cold 96–100% ethanol (BDH) for 1 min. The fixed cells were stored in a refrigerator until use [135].

### 4.9. Flow Cytometry Determination of Apoptotic Markers

The determinations of B cell lymphoma 2 (Bcl-2), the apoptosis regulator (BCL2-associated X protein) (BAX), Caspase 3, and the tumor protein (P53) in the colon tissues were carried out using a BD Accuri C6 flow cytometer.

### 4.10. Histopathological Investigation and Immunohistochemical Detection of P53 and Ki67

Histopathology was carried out according to Bancroft, using hematoxylin and eosin staining techniques [136]. Paraffin-embedded blocks of resected specimens were cut into 5 μm sections. The expression levels of P53 and Ki67 were analyzed using a standard avidin–biotin technique. The procedures were performed according to the manufacturer’s instructions [137].

### 4.11. RT-qPCR

Total RNA was extracted from the colorectal tissue using Qiazol (Qiagen; Hilden, Germany), according to the manufacturer’s guidelines. To estimate the total RNA concentration, we used a NanoDrop^®^ ND–1000 Spectrophotometer (NanoDrop Technologies; Wilmington, DE, USA). Using the cDNA Reverse Transcription Kit with High-Capacity (Applied Biosystems™, Waltham, MA, USA), the reverse transcription of the total RNA to cDNA was performed. The reverse transcription of the miRNA was carried out using 50 ng of the total extracted RNA, which was reverse-transcribed in a final volume of 20 µL (50 ng dissolved in 5µL of nuclease-free water, 4 µL of 5× miRCURY RT reaction buffer, 2.5 µL of 10× miRCURY RT Enzyme Mix, 1.2 µL of a predesigned stem-loop primer (Table 1) and 10 µL of RNase-free water), with a cycling condition of 42 °C for 60 min for the reverse transcription stage and 95 °C for the inactivation of the enzyme, according to the instructions of the manufacturer (Qiagen, Germany). The cDNA was then aliquoted and stored at −20 °C until use. The amplification of the cDNA was conducted in a real-time thermal cycler Rotor-Gene Q [138,139] with a SYBER Green master mix and a TOPreal™ qPCR 2×PreMIX (Enzynomics, Daejeon, Republic of Korea) with oligo-NTPs primers (Sangon Biotech, Beijing, China), as noted in Table 5. The relative gene expression over the normalizer gene Gapdh was finally calculated and represented as a percentage from the control, and the fold change was estimated as 2^−ΔΔct^ [140].

### 4.12. Statistical Analysis

The results are expressed as means ± standard error means (SEMs). A one-way analysis of variance (ANOVA) was used to analyze the data, followed by a test of the least significant difference (LSD). The analyses were conducted using the SPSS statistical package, version 19.00, software. A *p* value ≤ 0.05 was considered statistically significant.

## 5. Conclusions

Based on the available information, it appears that both AP and P demonstrated some potential in terms of antioxidant and apoptotic activities in male rats with PHZ-induced colon cancer. However, intensive studies are required to be able to draw any definitive conclusions about the effectiveness of either drug. It is important to note that animal studies are often used as a preliminary step in evaluating the potential effectiveness and safety of new drugs, but the results of these studies may not always translate directly to humans. Further research, including clinical trials in humans, will be necessary to fully evaluate the safety and efficacy of these candidate drugs.

## Figures and Tables

**Table 1:** GC-Mass analysis report for the AP extract.

Bioactive Compounds	RT	Area%	MF	MW	Formula	Activity—Based on the Previous Literature	References
à-D-GLUCOPYRANOSIDE, METHYL	21.36	1.68	739	331	C_13_H_26_BNO_6_Si	Anticancer Antioxidant	[46,47]
5,8,11,14-Eicosatetraynoic acid, TMS derivative	21.36	9.68	707	368	C_23_H_32_O_2_Si	Anticancer	[48]
Dasycarpidan-1-methanol, acetate	21.36	12.68	713	326	C_20_H_26_N_2_O_2_	Anticancer	[49]
Traumatic acid, (*E*)-, 2TMS derivative	22.74	0.69	710	372	C_18_H_36_O_4_Si_2_	Anticancer Antioxidant	[50]
Panaxydol, TMS	22.6	11.25	694	332	C_20_H_32_O_2_Si	Anticancer	[51]
2-Oleoylglycerol, 2TMS derivative	22.88	0.36	731	500	C_27_H_56_O_4_Si_2_	Antioxidant	[52]
MANNOFURANOSIDE, METHYL 2,3,5,6-TETRAKIS-O-(TRIMETHY LSILYL)-, à-D-	23.31	8.56	772	482	C_19_H_46_O_6_Si_4_	Anticancer Antimicrobial Antioxidant	[53]
Uridine, 3TMS derivative	23.31	7.56	717	460	C_18_H_36_N_2_O_6_Si_3_	Anticancer	[54]
á-D-GALACTOPYRANOSIDE, METHYL	23.07	15.38	734	362	C_14_H_31_BO_6_Si_2_	Anticancer	[55]
Methyl à-D-glucofuranoside, 4TMS derivative	24.43	2.5	836	482	C_19_H_46_O_6_Si_4_	Anticancer	[56]
D-(-)-Tagatofuranose,pentakis(trimethylsilyl) ether (isomer1)	24.77	8.77	765	540	C_21_H_52_O_6_Si_5_	Antioxidant	[57]
D-(-)-Fructofuranose, pentakis(trimethylsilyl) ether (isomer2)	24.82	8.91	782	540	C_21_H_52_O_6_Si_5_	Antioxidant Anti-inflammatory	[58]
D-Psicofuranose, pentakis(trimethylsilyl) ether (isomer1)	24.82	10.91	777	540	C_21_H_52_O_6_Si_5_	Antioxidant anticancer	[58]
1,5-Anhydrohexitol, 4TMS derivative	25.44	9.97	797	452	C_18_H_44_O_5_Si_4_	Antioxidant	[59]
á-DL-ARABINOPYRANOSE, 1,2,3,4-TETRAKIS-O-(TRIMETHY LSILYL)-	25.44	8.97	807	438	C_17_H_42_O_5_Si_4_	Antioxidant anticancer	[60]
MANNOONIC ACID, 2,3,5,6-TETRAKIS-O-(TRIMETHY LSILYL)-, LACTONE	26.2	6.2	728	466	C_18_H_42_O_6_Si_4_	Antioxidant anticancer	[61]
Dulcitol, 6TMS derivative	27.72	6.27	759	614	C_24_H_62_O_6_Si_6_	Anti-inflammatory Anticancer	[62]
D-Sorbitol, 6TMS derivative	27.72	6.27	752	614	C_24_H_62_O_6_Si_6_	Antioxidant	[63]
BUTANAL, 2,3,4-TRIS[(TRIMETHYLSILYL)O XY]-, (R*,R*)-	27.72	6.27	870	336	C_13_H_32_O_4_Si_3_	Antioxidant anticancer	[64]
L-Fucitol, 5TMS derivative	27.72	6.27	752	526	C_21_H_54_O_5_Si_5_	Antioxidant anticancer	[65]

MF; a matching factor or a direct match. It is a comparison of the unknown mass spectrum’s peaks to those of the peaks in the library’s spectra. This number, therefore, is an indication of how similar the unknown spectrum is to the library’s known spectra. The suggested general guidelines of the National Institute of Standards and Technology (NIST) for match factor scores are as follows: >900 is an excellent match, 800–900 is a good match, 700–800 is a fair match, and <600 is a poor match. RT; retention time, MW; molecular weight.

**Table 2:** Effects of different treatments on oxidative stress markers (MDA, PC, and NO) in the colons of male albino rats.

Groups	MDA	PC	NO
Control	12.12 ± 0.351 ^d^	0.837 ± 0.035 ^cd^	0.625 ± 0.027 ^c^
AP	11.38 ± 0.756 ^d^	0.773 ± 0.026 ^d^	0.644 ± 0.626 ^c^
P	12.00 ± 0.425 ^d^	0.787 ± 0.223 ^d^	0.740 ± 0.040 ^c^
PHZ	33.183 ± 1.359 ^a^	3.152 ± 0.078 ^a^	5.183 ± 0.252 ^a^
PHZ + AP	18.88 ± 1.339 ^bc^	1.830 ± 0.044 ^b^	0.756 ± 0.110 ^c^
PHZ + P	21.37 ± 1.644 ^b^	1.732 ± 0.026 ^b^	0.752 ± 0.045 ^c^
PHZ + AP + P	19.45 ± 1.981 ^b^	1.728 ± 0.030 ^b^	1.120 ± 0.102 ^b^
PHZ + AP + P (Prophylaxis)	16.37 ± 1.620 ^c^	1.163 ± 0.032 ^c^	0.820 ± 0.028 ^c^

Data are presented as means ± SEMs. ^a,b,c,d^ Different superscript letters indicate a significant difference (*p* < 0.05).

**Table 3:** Effects of different treatments on antioxidant enzymes (SOD, CAT, GST, GSH, GPx, and TAC) in the colons of male albino rats.

Groups	SOD	CAT	GST	GSH	GPx	TAC
Control	459.17 ± 12.21 ^a^	11.80 ± 0.52 ^a^	12.40 ± 0.57 ^a^	11.17 ± 0.32 ^a^	31.50 ± 0.52 ^a^	5.13 ± 0.39 ^a^
AP	456.00 ± 12.37 ^a^	10.28 ± 1.07 ^a^	14.14 ± 0.59 ^a^	10.82 ± 0.58 ^a^	32.36 ± 2.71 ^a^	4.24 ± 0.94 ^ab^
P	468.17 ± 14.71 ^a^	11.42 ± 0.31 ^a^	12.97 ± 0.17 ^a^	10.94 ± 0.19 ^a^	27.67 ± 0.88 ^b^	5.23 ± 0.39 ^a^
PHZ	224.83 ± 9.46 ^c^	3.29 ± 0.77 ^c^	4.88 ± 0.42 ^c^	4.11 ± 0.63 ^b^	3.88 ± 0.56 ^d^	0.52 ± 0.12 ^c^
PHZ + AP	401.00 ± 24.75 ^ab^	6.86 ± 0.71 ^b^	10.00 ± 0.91 ^ab^	8.48 ± 0.59 ^a^	16.34 ± 2.17 ^c^	3.78 ± 0.30 ^b^
PHZ + P	419.83 ± 15.56 ^ab^	9.12 ± 0.52 ^a^	8.66 ± 0.64 ^b^	8.50 ± 0.46 ^a^	27.82 ± 1.65 ^b^	3.58 ± 0.24 ^b^
PHZ + AP + P	386.00 ± 17.89 ^b^	9.90 ± 0.33 ^a^	9.39 ± 0.43 ^ab^	8.28 ± 0.88 ^a^	29.68 ± 1.70 ^ab^	4.83 ± 0.34 ^a^
PHZ + AP + P (Prophylaxis)	429.17 ± 20.27 ^ab^	10.47 ± 0.47 ^a^	11.48 ± 0.41 ^ab^	9.65 ± 0.67 ^a^	32.20 ± 1.72 ^a^	4.30 ± 0.30 ^ab^

Data are presented as means ± SEMs. ^a,b,c,d^ Different superscript letters indicate a significant difference (*p* < 0.05).

**Table 4:** Physicochemical standardization of the *Adiantum pedatum* (AP) extract.

Items	Results
Moisture content%	9.57 ± 0.33
Cold extraction	
Petroleum ether extracts (Cold)	10.97 ± 0.49
Chloroform extracts (Cold)	14.75 ± 0.54
Methanol extracts (Cold)	8.25 ± 0.19
Aqueous extract (cold)	2.85 ± 0.29
Hot extraction	
Chloroform extract (Hot)	0.33 ± 0.02
Alcoholic extract (Hot)	17.66 ± 0.46
Aqueous extract (hot)	12.76 ± 0.82
Total Ash	7.75 ± 0.29
Acid insoluble ash	3.45 ± 0.17
Water soluble ash	8.8 ± 0.316
Test for phenolic compounds	
Total Phenolic %	4.6 ± 0.18
pH	
5% 10%	5.62 5.48
TEST FOR SAPONINS	
Foam Test	+
TEST FOR TANNINS	
(a) Ferric chloride reagent (b) Lead acetate test (c) Potassium dichromate test	+ + +
TEST FOR FLAVANOIDS	
Shinoda Test	+
TEST FOR PROTEINS	
(a) Biuret Test (b) Xanthoproteic test	+ +

+ means positive reaction.

**Table 5:** Primers and stem-loop sequences of targeted genes.

Gene	Forward Primer (5′ to 3′)	Reverse Primer (5′ to 3′)	Product Size	Accession No.
Gapdh	GCATCTTCTTGTGCAGTGCC	GGTAACCAGGCGTCCGATAC	91	NM_017008.4
Myc	CAACAACCGCAAATGCTCCA	AGCTACGCTTCAGCTCGTTT	110	NM_012603.2
P53	CCCCTGAAGACTGGATAACTGT	TCTCCTGACTCAGAGGGAGC	75	NM_030989.3
PDCD4	CGGCCCGAGGGGATTCTAAA	GGGTCAGTGGGGTTCACATT	123	NM_022265.3
CK-20	CGCATCAATACTGTGCGGTG	AGCTCCCCAGAGTGAAAACG	91	NM_173128.2
AKT-1	GAAGGAGAAGGCCACAGGTC	TTCTGCAGGACACGGTTCTC	111	NM_033230.3
PI3K	CCCTGCCCCATTTCATCCTT	TGTTGTTGCCCCAGACATGA	162	NM_053481.2
SOX2	ACAGAGAAAACCTGAGGGCG	CATCGCCCGGAGTCTAGTTC	173	NM_001109181.2
Nanog	TGCATTTGTCTGAGCTGGGT	ATGGAGTAGGGTGGGTGTGT	115	NM_001100781.1
OCT4	AAGTTGGCGTGGAGACTCTG	GGACTCCTCGGGACTAGGTT	143	NM_001009178.2
mir-145-5P	AACCGGGTCCAGTTTTCCC	GTCGTATCCAGTGCAGGGT		
U6	GCTCGCTTCGGCAGCACA	GAGGTATTCGCACCAGAGGA		
mir-145-5P stem-loop	GTCGTATCCAGTGCAGGGTCCGAGGTATTCGCACTGGATACGACAGGGAT			
U6 stem-loop	AACGCTTCACGAATTTGCGTG			

## Data Availability

The data presented in this study are available upon request from the corresponding author.

## Supplementary Materials

The following supporting information can be downloaded at: https://www.mdpi.com/article/10.3390/molecules28145543/s1.

## Sample Availability

Samples of the compounds used in this study are available from the authors upon reasonable request.

## References

[1] Anand P., Kunnumakara A.B., Sundaram C., Harikumar K.B., Tharakan S.T., Lai O.S., Sung B., Aggarwal B.B. (2008). Cancer is a Preventable Disease that Requires Major Lifestyle Changes. Pharm. Res..

[2] Rawla P., Sunkara T., Barsouk A. (2019). Epidemiology of colorectal cancer: Incidence, mortality, survival, and risk factors. Prz. Gastroenterol..

[3] Hämälistö S., Jäättelä M. (2016). Lysosomes in cancer—Living on the edge (of the cell). Curr. Opin. Cell Biol..

[4] Yilmazer A. (2018). Cancer cell lines involving cancer stem cell populations respond to oxidative stress. Biotechnol. Rep..

[5] Alcaraz R., Muñiz P., Cavia M., Palacios Ó., Samper K.G., Gil-García R., Jiménez-Pérez A., García-Tojal J., García-Girón C. (2020). Thiosemicarbazone-metal complexes exhibiting cytotoxicity in colon cancer cell lines through oxidative stress. J. Inorg. Biochem..

[6] Valko M., Rhodes C.J., Moncol J., Izakovic M., Mazur M. (2006). Free radicals, metals and antioxidants in oxidative stress-induced cancer. Chem.-Biol. Interact..

[7] Obtułowicz T., Winczura A., Speina E., Swoboda M., Janik J., Janowska B., Cieśla J.M., Kowalczyk P., Jawien A., Gackowski D. (2010). Aberrant repair of etheno–DNA adducts in leukocytes and colon tissue of colon cancer patients. Free Radic. Biol. Med..

[8] Erekat N.S. (2022). Programmed cell death in cerebellar Purkinje neurons. J. Integr. Neurosci..

[9] Taylor R.C., Cullen S.P., Martin S.J. (2008). Apoptosis: Controlled demolition at the cellular level. Nat. Rev. Mol. Cell Biol..

[10] Hassan M., Feyen O., Grinstein E. (2009). Fas-Induced Apoptosis of Renal Cell Carcinoma is Mediated by Apoptosis Signal-Regulating Kinase 1 via Mitochondrial Damage-Dependent Caspase-8 Activation. Anal. Cell. Pathol..

[11] Adams J.M., Cory S. (2007). The Bcl-2 apoptotic switch in cancer development and therapy. Oncogene.

[12] Su Z., Yang Z., Xu Y., Chen Y., Yu Q. (2015). Apoptosis, autophagy, necroptosis, and cancer metastasis. Mol. Cancer.

[13] Giansanti V., Torriglia A., Scovassi A.I. (2011). Conversation between apoptosis and autophagy: “Is it your turn or mine?”. Apoptosis.

[14] Bukholm I.K., Nesland J.M. (2000). Protein expression of p53, p21 (WAF1/CIP1), bcl-2, Bax, cyclin D1 and pRb in human colon carcinomas. Virchows Arch..

[15] Huerta S., Goulet E.J., Livingston E.H. (2006). Colon cancer and apoptosis. Am. J. Surg..

[16] Jiang N., Dai Q., Su X., Fu J., Feng X., Peng J. (2020). Role of PI3K/AKT pathway in cancer: The framework of malignant behavior. Mol. Biol. Rep..

[17] Elbadawy M., Usui T., Yamawaki H., Sasaki K. (2019). Emerging Roles of C-Myc in Cancer Stem Cell-Related Signaling and Resistance to Cancer Chemotherapy: A Potential Therapeutic Target Against Colorectal Cancer. Int. J. Mol. Sci..

[18] Feng Y.C., Liu X.Y., Teng L., Ji Q., Wu Y., Li J.M., Gao W., Zhang Y.Y., La T., Tabatabaee H. (2020). c-Myc inactivation of p53 through the pan-cancer lncRNA MILIP drives cancer pathogenesis. Nat. Commun..

[19] Wang Q., Yang H.S. (2018). The role of Pdcd4 in tumour suppression and protein translation. Biol. Cell.

[20] Karim S., Burzangi A.S., Ahmad A., Siddiqui N.A., Ibrahim I.M., Sharma P., Abualsunun W.A., Gabr G.A. (2022). PI3K-AKT Pathway Modulation by Thymoquinone Limits Tumor Growth and Glycolytic Metabolism in Colorectal Cancer. Int. J. Mol. Sci..

[21] Ahadi A. (2020). The significance of microRNA deregulation in colorectal cancer development and the clinical uses as a diagnostic and prognostic biomarker and therapeutic agent. Non-Coding RNA Res..

[22] Peng Y., Croce C.M. (2016). The role of MicroRNAs in human cancer. Signal Transduct. Target. Ther..

[23] Davis-Dusenbery B.N., Hata A. (2010). MicroRNA in Cancer: The Involvement of Aberrant MicroRNA Biogenesis Regulatory Pathways. Genes Cancer.

[24] Imedio L., Cristóbal I., Rubio J., Santos A., Rojo F., García-Foncillas J. (2020). MicroRNAs in Rectal Cancer: Functional Significance and Promising Therapeutic Value. Cancers.

[25] Ge N., Lin H.X., Xiao X.S., Guo L., Xu H.M., Wang X., Jin T., Cai X.Y., Liang Y., Hu W.H. (2010). Prognostic significance of Oct4 and Sox2 expression in hypopharyngeal squamous cell carcinoma. J. Transl. Med..

[26] Wang Z.X., Teh C.H., Kueh J.L., Lufkin T., Robson P., Stanton L.W. (2007). Oct4 and Sox2 directly regulate expression of another pluripotency transcription factor, Zfp206, in embryonic stem cells. J. Biol. Chem..

[27] Giagulli V.A., Carbone M.D., Ramunni M.I., Licchelli B., De Pergola G., Sabbà C., Guastamacchia E., Triggiani V. (2015). Adding liraglutide to lifestyle changes, metformin and testosterone therapy boosts erectile function in diabetic obese men with overt hypogonadism. Andrology.

[28] Shirendeb U., Hishikawa Y., Moriyama S., Win N., Minn Myint Thu M., Swe Mar K., Khatanbaatar G., Masuzaki H., Koji T. (2009). Human Papillomavirus Infection and Its Possible Correlation with p63 Expression in Cervical Cancer in Japan, Mongolia, and Myanmar. Acta Histochem. Cytochem..

[29] Sun X., Kaufman P.D. (2018). Ki-67: More than a proliferation marker. Chromosoma.

[30] KlÖPpel G., Perren A., Heitz P.U. (2004). The Gastroenteropancreatic Neuroendocrine Cell System and Its Tumors: The WHO Classification. Ann. N. Y. Acad. Sci..

[31] Sueishi Y., Nii R., Kakizaki N. (2017). Resveratrol analogues like piceatannol are potent antioxidants as quantitatively demonstrated through the high scavenging ability against reactive oxygen species and methyl radical. Bioorg. Med. Chem. Lett..

[32] Wittgen H.G.M., van Kempen L.C.L.T. (2007). Reactive oxygen species in melanoma and its therapeutic implications. Melanoma Res..

[33] Piotrowska H., Kucinska M., Murias M. (2012). Biological activity of piceatannol: Leaving the shadow of resveratrol. Mutat. Res./Rev. Mutat. Res..

[34] Huang X.M., Yang Z.J., Xie Q., Zhang Z.K., Zhang H., Ma J.Y. (2019). Natural products for treating colorectal cancer: A mechanistic review. Biomed. Pharmacother..

[35] Alhakamy N.A., Badr-Eldin S.M., Ahmed O.A.A., Asfour H.Z., Aldawsari H.M., Algandaby M.M., Eid B.G., Abdel-Naim A.B., Awan Z.A., Alghaith A.F. (2020). Piceatannol-Loaded Emulsomes Exhibit Enhanced Cytostatic and Apoptotic Activities in Colon Cancer Cells. Antioxidants.

[36] Chiou Y.S., Lan Y.M., Lee P.S., Lin Q., Nagabhushanam K., Ho C.T., Pan M.H. (2022). Piceatannol Prevents Colon Cancer Progression via Dual-Targeting to M2-Polarized Tumor-Associated Macrophages and the TGF-β1 Positive Feedback Signaling Pathway. Mol. Nutr. Food Res..

[37] Greenwell M., Rahman P.K. (2015). Medicinal Plants: Their Use in Anticancer Treatment. Int. J. Pharm. Sci. Res..

[38] Nosrati N., Bakovic M., Paliyath G. (2017). Molecular Mechanisms and Pathways as Targets for Cancer Prevention and Progression with Dietary Compounds. Int. J. Mol. Sci..

[39] Savage P.B., Li C., Taotafa U., Ding B., Guan Q. (2002). Antibacterial properties of cationic steroid antibiotics. FEMS Microbiol. Lett..

[40] Talib W.H., Awajan D., Hamed R.A., Azzam A.O., Mahmod A.I., Al-Yasari I.H. (2022). Combination Anticancer Therapies Using Selected Phytochemicals. Molecules.

[41] Kooti W., Servatyari K., Behzadifar M., Asadi-Samani M., Sadeghi F., Nouri B., Zare Marzouni H. (2017). Effective Medicinal Plant in Cancer Treatment, Part 2: Review Study. J. Evid.-Based Complement. Altern. Med..

[42] Chandrappa C.P., Shilpashree C.B., Karthik M.R., Govindappa M., Sadananda T.S. (2011). Antibacterial and Antioxidant Activities of *Adiantum pedatum* L.. J. Phytol..

[43] Fan P., Zhao L., Hostettmann K., Lou H. (2012). Chemical constituents of *Asplenium rutamuraria* L.. Nat. Prod. Res..

[44] Stein S.E., Ausloos P., Lias S.G. (1991). Comparative evaluations of mass spectral databases. J. Am. Soc. Mass Spectrom..

[45] Stein S. (2012). Mass spectral reference libraries: An ever-expanding resource for chemical identification. Anal. Chem..

[46] Islam S., Hosen M.A., Ahmad S., ul Qamar M.T., Dey S., Hasan I., Fujii Y., Ozeki Y., Kawsar S.M.A. (2022). Synthesis, antimicrobial, anticancer activities, PASS prediction, molecular docking, molecular dynamics and pharmacokinetic studies of designed methyl α-D-glucopyranoside esters. J. Mol. Struct..

[47] Abdullah N.A., Zain W.Z.W.M., Ramli N.W., Hamzah F., Hamid N.A. (2022). Antioxidant and GC-MS Analysis of Cyperus iria, Fimbristyis miliacea, and Fimbristylis globulosa. IOP Conf. Ser. Earth Environ. Sci..

[48] Shahin A., Nabil-Adam A., Elnagar K., Osman H., Shreadah M.A. (2022). Bioactivity and metabolomics fingerprinting characterization of different organic solvents extracts of Padina pavonica collected from Abu Qir Bay, Egypt. Egypt. J. Chem..

[49] Nasr M., Naeem S.A., El-Shenbaby I., Mohamed F.M.A., Mahmoud S.M., Abuamara T.M.M., Abd-Elhay W.M., Elbayoumy F., Elkot A., Shikhon T. (2023). Pomegranate Seeds and Peel Ethanolic Extracts Anticancer Potentials and Related Genetic, Histological, Immunohistochemical, Apoptotic and Oxidative Stress Profiles: In vitro Study. J. Exp. Pharmacol..

[50] Jabłońska-Trypuć A., Wydro U., Wołejko E., Rodziewicz J., Butarewicz A. (2020). Possible Protective Effects of TA on the Cancerous Effect of Mesotrione. Nutrients.

[51] Kim H.S., Lim J.M., Kim J.Y., Kim Y., Park S., Sohn J. (2016). Panaxydol, a component of Panax ginseng, induces apoptosis in cancer cells through EGFR activation and ER stress and inhibits tumor growth in mouse models. Int. J. Cancer.

[52] Masek A., Latos-Brozio M., Kałużna-Czaplińska J., Rosiak A., Chrzescijanska E. (2020). Antioxidant Properties of Green Coffee Extract. Forests.

[53] Al-Abdallah B., Al-Faiyz Y.S., Shaaban S. (2022). Anticancer, Antimicrobial, and Antioxidant Activities of Organodiselenide-Tethered Methyl Anthranilates. Biomolecules.

[54] Khwaza V., Oyedeji O.O., Aderibigbe B.A. (2020). Ursolic Acid-Based Derivatives as Potential Anti-Cancer Agents: An Update. Int. J. Mol. Sci..

[55] Lyantagaye S.L. (2013). Methyl-α-D-glucopyranoside from Tulbaghia violacea extract induces apoptosis in vitro in cancer cells. Bangladesh J. Pharmacol..

[56] Abotaleb M., Samuel S.M., Varghese E., Varghese S., Kubatka P., Liskova A., Büsselberg D. (2019). Flavonoids in Cancer and Apoptosis. Cancers.

[57] Fatani A., Baothman O., Shash L., Abuaraki H., Zeyadi M., Hosawi S., Altayb H., Abo-Golayel M. (2022). Hepatoprotective effect of date palm fruit extract against doxorubicin intoxication in Wistar rats: In vivo and in silico studies. Asian Pac. J. Trop. Biomed..

[58] Ragab T.I.M., Ali N.A., El Gendy A.N.G., Mohamed S.H., Shalby A.B., Farrag A.H., Shalaby A.S.G. (2021). Renoprotective and therapeutic effects of newly water, ethanol, and butanol ginseng fractions in hypertensive and chronic kidney disease with L-NAME. Biomed. Pharmacother..

[59] Spanou C., Manta S., Komiotis D., Dervishi A., Kouretas D. (2007). Antioxidant Activity of a Series of Fluorinated Pyrano-nucleoside Analogues of N(4)-benzoyl Cytosine and N(6)-benzoyl Adenine. Int. J. Mol. Sci..

[60] Chen M., Meng H., Zhao Y., Chen F., Yu S. (2015). Antioxidant and in vitro anticancer activities of phenolics isolated from sugar beet molasses. BMC Complement. Altern. Med..

[61] Youssef A.M.M., Maaty D.A.M., Al-Saraireh Y.M. (2023). Phytochemical Analysis and Profiling of Antioxidants and Anticancer Compounds from *Tephrosia purpurea* (L.) subsp. apollinea Family Fabaceae. Molecules.

[62] Suresh V., Sruthi V., Padmaja B., Asha V.V. (2011). In vitro anti-inflammatory and anti-cancer activities of Cuscuta reflexa Roxb. J. Ethnopharmacol..

[63] El-Far M., Taie H. (2009). Antioxidant activities, total anthocyanins, phenolics and flavonoids contents of some sweetpotato genotypes under stress of different concentrations of sucrose and sorbitol. Aust. J. Basic Appl. Sci..

[64] Bekhouche K., Özen T., Boussaha S., Koldaş S., Yenigün S., Lassed S., Demirtas I., Benayache F., Samir B., Zama D. (2018). Anti-oxidant, DNA-damage protection and anti-cancer properties of n-butanol extract of the endemic Perralderia coronopifolia. Bangladesh J. Pharmacol..

[65] Pal L.C., Gautam A., Pande V., Rao C.V. (2022). Anticancer property of *Selaginella bryopteris* (L.) Bak. against hepatocellular carcinoma in vitro and in vivo. Phytomed. Plus.

[66] Aykan N.F. (2015). Red Meat and Colorectal Cancer. Oncol. Rev..

[67] Zhao Z., Feng Q., Yin Z., Shuang J., Bai B., Yu P., Guo M., Zhao Q. (2017). Red and processed meat consumption and colorectal cancer risk: A systematic review and meta-analysis. Oncotarget.

[68] Lu F., Kuhnle G.K., Cheng Q. (2017). Heterocyclic amines and polycyclic aromatic hydrocarbons in commercial ready-to-eat meat products on UK market. Food Control.

[69] Cross A.J., Ferrucci L.M., Risch A., Graubard B.I., Ward M.H., Park Y., Hollenbeck A.R., Schatzkin A., Sinha R. (2010). A large prospective study of meat consumption and colorectal cancer risk: An investigation of potential mechanisms underlying this association. Cancer Res..

[70] Huang L., Li W., Lu Y., Ju Q., Ouyang M. (2023). Iron metabolism in colorectal cancer. Front. Oncol..

[71] Bostan M., Mihaila M., Hotnog C., Bleotu C., Anton G., Roman V., Brasoveanu L.I. (2016). Modulation of Apoptosis in Colon Cancer Cells by Bioactive Compounds. Color. Cancer–Pathog. Treat..

[72] Russo M., Spagnuolo C., Tedesco I., Russo G.L. (2010). Phytochemicals in Cancer Prevention and Therapy: Truth or Dare?. Toxins.

[73] Ferrali M., Signorini C., Sugherini L., Pompella A., Lodovici M., Caciotti B., Ciccoli L., Comporti M. (1997). Release of free, redox-active iron in the liver and DNA oxidative damage following phenylhydrazine intoxication. Biochem. Pharmacol..

[74] Luangaram S., Kukongviriyapan U., Pakdeechote P., Kukongviriyapan V., Pannangpetch P. (2007). Protective effects of quercetin against phenylhydrazine-induced vascular dysfunction and oxidative stress in rats. Food Chem. Toxicol..

[75] Paul S., Ghosh A.K., Ghosh D., Dutta M., Mitra E., Dey M., Bhowmick D., Das T., Firdaus S.B., Mishra S. (2014). Aqueous bark extract of Terminalia arjuna protects against phenylhydrazine induced oxidative damage in goat red blood cell membrane protein, phospholipid asymmetry and structural morphology: A flow cytometric and biochemical analysis. J. Pharm. Res.

[76] Di Giacomo C., Acquaviva R., Lanteri R., Licata F., Licata A., Vanella A. (2003). Nonproteic Antioxidant Status in Plasma of Subjects with Colon Cancer. Exp. Biol. Med..

[77] Ofeimun J.O., Enwerem J.C., Benjamin G. (2020). Haematological And In-Vivo Antioxidant Modulatory Activities of Justicia Secunda Vahl [Acanthaceae] Leaf Extract In Phenylhydrazine-Induced Anemic Rats. Niger. J. Pharm..

[78] Aloke C., Uche Emelike C., Ajuka Obasi N., Nkemjika Ogbu P., Oswald Edeogu C., Godwin Uzomba C., Ekakitie O., Adewale Iyaniwura A., Okoro C.C., Peter Okey B. (2021). HPLC profiling and studies on Copaifera salikounda methanol leaf extract on phenylhydrazine-induced hematotoxicity and oxidative stress in rats. Arab. J. Chem..

[79] Jaiswal M., LaRusso N.F., Burgart L.J., Gores G.J. (2000). Inflammatory cytokines induce DNA damage and inhibit DNA repair in cholangiocarcinoma cells by a nitric oxide-dependent mechanism. Cancer Res..

[80] Benkhelifa S., Rafa H., Belhadef S., Ait-kaci H., Medjeber O., Belkhelfa M., Hetit S., Ait-Younes S., De Launoit Y., Moralès O. (2019). Aberrant up-regulation of iNOS/NO system is correlated with an increased abundance of Foxp3+ cells and reduced effector/memory cell markers expression during colorectal cancer: Immunomodulatory effects of cetuximab combined with chemotherapy. Inflammopharmacology.

[81] Costa F.P.d., Puty B., Nogueira L.S., Mitre G.P., Santos S.M.d., Teixeira B.J.B., Kataoka M.S.d.S., Martins M.D., Barboza C.A.G., Monteiro M.C. (2019). Piceatannol Increases Antioxidant Defense and Reduces Cell Death in Human Periodontal Ligament Fibroblast under Oxidative Stress. Antioxidants.

[82] Mahmoud Moustafa E., Rashed E.R., Rashed R.R., Omar N.N. (2021). Piceatannol promotes hepatic and renal AMPK/SIRT1/PGC-1α mitochondrial pathway in rats exposed to reserpine or gamma-radiation. Int. J. Immunopathol. Pharmacol..

[83] Kita Y., Miura Y., Yagasaki K. (2012). Antiproliferative and Anti-Invasive Effect of Piceatannol, a Polyphenol Present in Grapes and Wine, against Hepatoma AH109A Cells. J. Biomed. Biotechnol..

[84] Yang J.S., Tongson J., Kim K.-H., Park Y. (2020). Piceatannol attenuates fat accumulation and oxidative stress in steatosis-induced HepG2 cells. Curr. Res. Food Sci..

[85] Wahdan S.A., Azab S.S., Elsherbİny D.A., El-demerdash E. (2017). Piceatannol ameliorates cisplatin-induced histological and biochemical alterations in rats kidney. Int. J. Pharm. Pharm. Sci..

[86] Malki A., ElRuz R.A., Gupta I., Allouch A., Vranic S., Al Moustafa A.E. (2020). Molecular Mechanisms of Colon Cancer Progression and Metastasis: Recent Insights and Advancements. Int. J. Mol. Sci..

[87] Bardhan K., Liu K. (2013). Epigenetics and colorectal cancer pathogenesis. Cancers.

[88] Stefani C., Miricescu D., Stanescu S., Nica R.I., Greabu M., Totan A.R., Jinga M. (2021). Growth Factors, PI3K/AKT/mTOR and MAPK Signaling Pathways in Colorectal Cancer Pathogenesis: Where Are We Now?. Int. J. Mol. Sci..

[89] Ma Q. (2013). Role of nrf2 in oxidative stress and toxicity. Annu. Rev. Pharmacol. Toxicol..

[90] He F., Ru X., Wen T. (2020). NRF2, a Transcription Factor for Stress Response and Beyond. Int. J. Mol. Sci..

[91] Lee Y.J., Kim W.I., Bae J.H., Cho M.K., Lee S.H., Nam H.S., Choi I.H., Cho S.W. (2020). Overexpression of Nrf2 promotes colon cancer progression via ERK and AKT signaling pathways. Ann. Surg. Treat. Res..

[92] Li J., Wang D., Liu Y., Zhou Y. (2022). Role of NRF2 in Colorectal Cancer Prevention and Treatment. Technol. Cancer Res. Treat..

[93] Garufi A., Pistritto G., D’Orazi V., Cirone M., D’Orazi G. (2022). The Impact of NRF2 Inhibition on Drug-Induced Colon Cancer Cell Death and p53 Activity: A Pilot Study. Biomolecules.

[94] Hutvagner G., Zamore P.D.J.S. (2002). A microRNA in a multiple-turnover RNAi enzyme complex. Science.

[95] Bandrés E., Cubedo E., Agirre X., Malumbres R., Zarate R., Ramirez N., Abajo A., Navarro A., Moreno I., Monzo M. (2006). Identification by Real-time PCR of 13 mature microRNAs differentially expressed in colorectal cancer and non-tumoral tissues. Mol. Cancer.

[96] Sachdeva M., Zhu S., Wu F., Wu H., Walia V., Kumar S., Elble R., Watabe K., Mo Y.-Y. (2009). p53 represses c-Myc through induction of the tumor suppressor miR-145. Proc. Natl. Acad. Sci. USA.

[97] Spizzo R., Nicoloso M., Lupini L., Lu Y., Fogarty J., Rossi S., Zagatti B., Fabbri M., Veronese A., Liu X. (2010). miR-145 participates with TP53 in a death-promoting regulatory loop and targets estrogen receptor-α in human breast cancer cells. Cell Death Differ..

[98] Suzuki H.I., Yamagata K., Sugimoto K., Iwamoto T., Kato S., Miyazono K. (2009). Modulation of microRNA processing by p53. Nature.

[99] Kent O.A., Chivukula R.R., Mullendore M., Wentzel E.A., Feldmann G., Lee K.H., Liu S., Leach S.D., Maitra A., Mendell J.T. (2010). Repression of the miR-143/145 cluster by oncogenic Ras initiates a tumor-promoting feed-forward pathway. Genes Dev..

[100] Hatley M.E., Patrick D.M., Garcia M.R., Richardson J.A., Bassel-Duby R., Van Rooij E., Olson E.N. (2010). Modulation of K-Ras-dependent lung tumorigenesis by MicroRNA-21. Cancer Cell.

[101] Ibrahim A.F., Weirauch U., Thomas M., Grünweller A., Hartmann R.K., Aigner A. (2011). MicroRNA replacement therapy for miR-145 and miR-33a is efficacious in a model of colon carcinoma. Cancer Res..

[102] Götte M., Mohr C., Koo C., Stock C., Vaske A., Viola M., Ibrahim S., Peddibhotla S., Teng Y.H., Low J. (2010). miR-145-dependent targeting of junctional adhesion molecule A and modulation of fascin expression are associated with reduced breast cancer cell motility and invasiveness. Oncogene.

[103] Sachdeva M., Mo Y.-Y. (2010). MicroRNA-145 Suppresses Cell Invasion and Metastasis by Directly Targeting Mucin 1Suppression of Cell Invasion and Metastasis by miR-145. Cancer Res..

[104] Zhang J., Guo H., Zhang H., Wang H., Qian G., Fan X., Hoffman A.R., Hu J.F., Ge S. (2011). Putative tumor suppressor miR—145 inhibits colon cancer cell growth by targeting oncogene friend leukemia virus integration 1 gene. Cancer.

[105] Sung S.H., Kim K.H., Jeon B.T., Cheong S.H., Park J.H., Kim D.H., Kweon H.J., Moon S.H. (2012). Antibacterial and antioxidant activities of tannins extracted from agricultural by-products. J. Med. Plants Res..

[106] Minussi R.C., Rossi M., Bologna L., Cordi L.v., Rotilio D., Pastore G.M., Durán N. (2003). Phenolic compounds and total antioxidant potential of commercial wines. Food Chem..

[107] Yu J., Zhang L. (2004). Apoptosis in human cancer cells. Curr. Opin. Oncol..

[108] Zhang L., Yu J. (2013). Role of Apoptosis in Colon Cancer Biology, Therapy, and Prevention. Curr. Color. Cancer Rep..

[109] Coultas L., Strasser A. (2003). The role of the Bcl-2 protein family in cancer. Semin. Cancer Biol..

[110] Nik Salleh N.N.H., Othman F.A., Kamarudin N.A., Tan S.C. (2020). The Biological Activities and Therapeutic Potentials of Baicalein Extracted from Oroxylum indicum: A Systematic Review. Molecules.

[111] NaveenKumar S.K., Thushara R.M., Sundaram M.S., Hemshekhar M., Paul M., Thirunavukkarasu C., Basappa, Nagaraju G., Raghavan S.C., Girish K.S. (2015). Unconjugated Bilirubin exerts Pro-Apoptotic Effect on Platelets via p38-MAPK activation. Sci. Rep..

[112] Song H., Jung J.I., Cho H.J., Her S., Kwon S.-H., Yu R., Kang Y.-H., Lee K.W., Park J.H.Y. (2015). Inhibition of tumor progression by oral piceatannol in mouse 4T1 mammary cancer is associated with decreased angiogenesis and macrophage infiltration. J. Nutr. Biochem..

[113] Wu L.-S., Wang X.-W., He W., Ma X.-T., Wang H.-Y., Han M., Li B.-H. (2019). TRAIL inhibits platelet-induced colorectal cancer cell invasion. J. Int. Med. Res..

[114] Kang C.-H., Moon D.-O., Choi Y.H., Choi I.-W., Moon S.-K., Kim W.-J., Kim G.-Y. (2011). Piceatannol enhances TRAIL-induced apoptosis in human leukemia THP-1 cells through Sp1- and ERK-dependent DR5 up-regulation. Toxicol. In Vitro.

[115] Alrawi S.J., Stoler D., Carroll R.E., Gibbs J.F., Schiff M., Tan D., Dayton M., Anderson G.R. (2006). New parameters in aberrant crypt foci evaluation in colon carcinogenesis (glutathione S-transferases, B-RAF mutation, genomic instability, single nucleotide polymorphism (SNP) arrays). J. Surg. Res..

[116] Darwish A., Alemam D., Sheta H. (2021). Role of expression of p53 and Ki67 in the progression of Wilms tumor; correlation with survival. Int. J. Cancer Biomed. Res..

[117] Nussrat F.L., Ali H.H., Hussein H.G., Al-Ukashi R.J. (2011). Immunohistochemical Expression of ki-67 and p53 in Colorectal Adenomas: A Clinicopathological Study. Oman Med. J..

[118] Hahn W.C., Weinberg R.A. (2002). Modelling the molecular circuitry of cancer. Nat. Rev. Cancer.

[119] Lee H.-N., Jang H.-Y., Kim H.-J., Shin S.-A., Choo G.-S., Park B.-K., Kim B.-S., Jung J.-Y. (2015). Induction of Apoptosis by Piceatannol in YD-15 Human Oral Cancer Cells. J. Korean Soc. Food Sci. Nutr..

[120] Rodríguez J.D.W., Peyron S., Rigou P., Chalier P. (2018). Rapid quantification of clove (*Syzygium aromaticum*) and spearmint (*Mentha spicata*) essential oils encapsulated in a complex organic matrix using an ATR-FTIR spectroscopic method. PLoS ONE.

[121] Enan G., Al-Mohammadi A.-R., Mahgoub S., Abdel-Shafi S., Askar E., Ghaly M.F., Taha M.A., El-Gazzar N.J.M. (2020). Inhibition of Staphylococcus aureus LC 554891 by Moringa oleifera seed extract either singly or in combination with antibiotics. Molecules.

[122] Nazim M., Aslam D., Khatoon R., Asif D., Chaudhary S. (2018). Physico-chemical standardization of Hansraj (*Adiantum capillus-Veneris*). J. Drug Deliv. Ther..

[123] Berger J. (2007). Phenylhydrazine haematotoxicity. J. Appl. Biomed..

[124] Awaad A., Adly M.A., Ellatef M.A.A., Foad M.M. (2021). Comparative Expression of P53 and Survivin Proteins in Phenylhydrazine-Induced Colon Cancer of Rats and the Role of Electromagnetic Field and Broccoli Extract. Ph.D. Thesis.

[125] Gupta R.K., Patel A.K., Shah N., Choudhary A.K., Jha U.K., Yadav U.C., Gupta P.K., Pakuwal U. (2014). Oxidative Stress and Antioxidants in Disease and Cancer: A Review. Asian Pac. J. Cancer Prev..

[126] Aljabali A.A.A., Bakshi H.A., Hakkim F.L., Haggag Y.A., Al-Batanyeh K.M., Zoubi M.S.A., Al-Trad B., Nasef M.M., Satija S., Mehta M. (2020). Albumin Nano-Encapsulation of Piceatannol Enhances Its Anticancer Potential in Colon Cancer via down Regulation of Nuclear p65 and HIF-1α. Cancers.

[127] Ohkawa H., Ohishi N., Yagi K. (1979). Assay for lipid peroxides in animal tissues by thiobarbituric acid reaction. Anal. Biochem..

[128] Buss H., Chan T.P., Sluis K.B., Domigan N.M., Winterbourn C.C. (1997). Protein Carbonyl Measurement by a Sensitive ELISA Method. Free Radic. Biol. Med..

[129] Ingram G., Montgomery H.A.C., Dymock J.F., Henneberry G.O., Baker B.E., Forbes J.S., Dalladay D.B., Bloxam T.W. (1961). Notes. Analyst.

[130] Koracevic D. (2001). Method for the measurement of antioxidant activity in human fluids. J. Clin. Pathol..

[131] Aebi H. (1984). [13] Catalase in vitro. Methods Enzymol..

[132] Nishikimi M., Appaji Rao N., Yagi K. (1972). The occurrence of superoxide anion in the reaction of reduced phenazine methosulfate and molecular oxygen. Biochem. Biophys. Res. Commun..

[133] Beutler E., Yeh M.K.Y. (1963). Erythrocyte Glutathione Reductase. Blood.

[134] Habig W.H., Pabst M.J., Jakoby W.B. (1974). Glutathione S-Transferases. J. Biol. Chem..

[135] Tribukait B., Esposti P.L. (1978). Quantitative flow-microfluorometric analysis of the DNA in cells from neoplasms of the urinary bladder: Correlation of aneuploidy with histological grading and the cytological findings. Urol. Res..

[136] Bancroft J.D., Layton C. (2013). The hematoxylins and eosin. Bancroft’s Theory Pract. Histol. Tech..

[137] Peng Y., Wang L., Gu J. (2013). Elevated preoperative carcinoembryonic antigen (CEA) and Ki67 is predictor of decreased survival in IIA stage colon cancer. World J. Surg..

[138] Khamis T., Abdelalim A.F., Abdallah S.H., Saeed A.A., Edress N.M., Arisha A.H. (2020). Early intervention with breast milk mesenchymal stem cells attenuates the development of diabetic-induced testicular dysfunction via hypothalamic Kisspeptin/Kiss1r-GnRH/GnIH system in male rats. Biochim. Biophys. Acta (BBA)-Mol. Basis Dis..

[139] Khamis T., Abdelalim A.F., Saeed A.A., Edress N.M., Nafea A., Ebian H.F., Algendy R., Hendawy D.M., Arisha A.H., Abdallah S.H. (2021). Breast milk MSCs upregulated β-cells PDX1, Ngn3, and PCNA expression via remodeling ER stress/inflammatory/apoptotic signaling pathways in type 1 diabetic rats. Eur. J. Pharmacol..

[140] Schmittgen T.D., Livak K.J. (2008). Analyzing real-time PCR data by the comparative C(T) method. Nat. Protoc..

