# Supplementary material for: The Antiproliferative Activity of Adiantum pedatum Extract and/or Piceatannol in Phenylhydrazine-Induced Colon Cancer in Male Albino Rats: The miR-145 Expression of the PI-3K/Akt/p53 and Oct4/Sox2/Nanog Pathways
Source: Molecules. 2023 Jul 20;28(14):5543. doi: 10.3390/molecules28145543 (PMC10383735; doi:10.3390/molecules28145543)

# *My GC-MS Report*

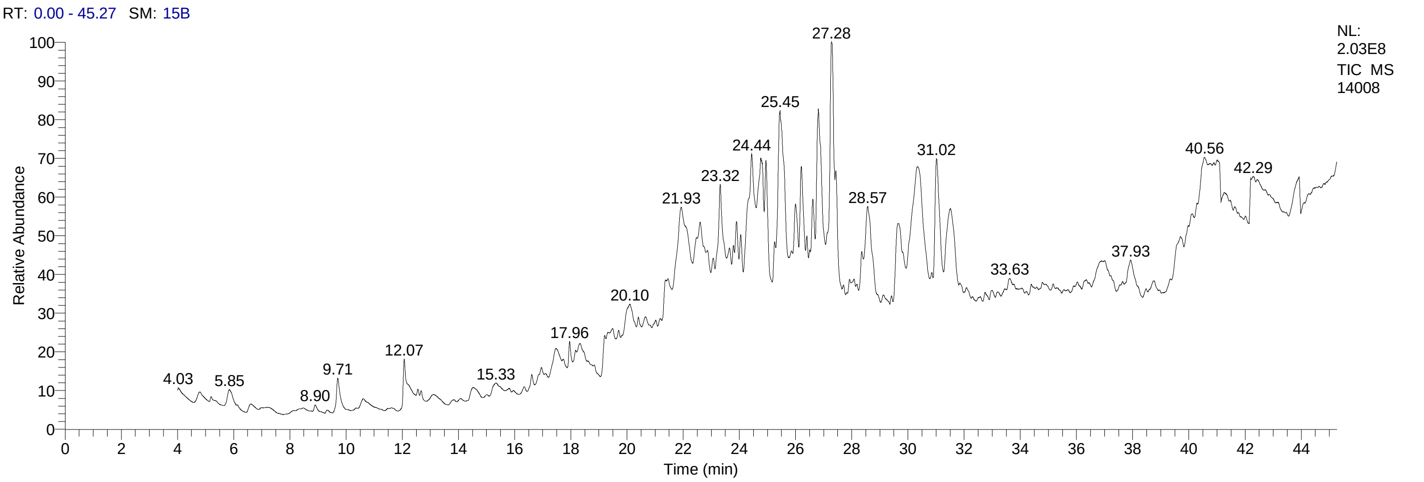


| RT | Area % | Peak Area | Peak Height |
|---:|---:|---:|---:|
| 5.84 | 0.71 | 63705883.66 | 6598600.64 |
| 9.70 | 0.86 | 77173509.68 | 13635953.81 |
| 12.07 | 2.27 | 202957060.93 | 25070442.31 |
| 16.61 | 0.41 | 36501124.01 | 7019474.56 |
| 17.46 | 1.34 | 119742414.79 | 9558040.67 |
| 17.96 | 0.64 | 57129492.35 | 13667943.59 |
| 18.17 | 0.32 | 28301911.30 | 6352589.84 |
| 18.33 | 1.31 | 116985804.68 | 9417661.22 |
| 19.20 | 0.98 | 87553744.02 | 15972746.22 |
| 19.30 | 1.93 | 171849306.80 | 13059370.36 |
| 20.02 | 0.69 | 61692392.57 | 9920707.55 |
| 20.10 | 0.93 | 83125116.69 | 10650713.87 |
| 21.36 | 1.68 | 149713892.98 | 15244142.55 |
| 21.92 | 1.34 | 119672317.64 | 14925900.20 |
| 22.47 | 0.69 | 61839381.49 | 10548396.56 |
| 22.60 | 1.25 | 111125117.77 | 15137116.25 |
| 22.88 | 0.36 | 31887394.58 | 6503154.24 |
| 23.07 | 0.38 | 33806191.86 | 7257448.27 |
| 23.31 | 2.56 | 228137259.66 | 36548472.46 |
| 23.66 | 0.47 | 41877983.03 | 8191584.06 |
| 23.79 | 0.44 | 39402592.13 | 11154183.82 |
| 23.90 | 1.27 | 113329520.06 | 22602758.70 |
| 24.05 | 0.85 | 75420017.61 | 17872770.16 |
| 24.31 | 1.80 | 160443498.69 | 21174138.40 |
| 24.43 | 2.50 | 223072776.44 | 34862051.19 |
| 24.77 | 1.77 | 158052998.77 | 30284999.39 |
| 24.82 | 0.91 | 81307492.60 | 28254286.05 |
| 24.95 | 2.37 | 211572268.31 | 40689001.82 |
| 25.25 | 0.47 | 42242749.05 | 12335142.04 |
| 25.44 | 8.97 | 800301350.23 | 67108837.40 |
| 25.99 | 1.81 | 161617585.33 | 25335443.50 |
| 26.20 | 3.20 | 285570462.16 | 43912979.25 |
| 26.40 | 0.39 | 34724151.70 | 11765079.48 |
| 26.61 | 1.47 | 131139801.29 | 27003687.08 |
| 26.81 | 6.38 | 569115396.02 | 65820489.74 |
| 27.27 | 6.27 | 559040319.79 | 85827775.67 |
| 27.45 | 0.99 | 88355993.17 | 23070932.75 |
| 27.92 | 0.37 | 32978118.72 | 8055922.87 |
| 28.08 | 0.62 | 54934768.94 | 7381560.91 |
| 28.35 | 0.56 | 49718505.97 | 12277147.83 |
| 28.57 | 2.45 | 218163345.18 | 24309427.27 |
| 29.62 | 2.98 | 265570783.98 | 26501969.85 |

# My GC-MS Report

| RT | Area % | Peak Area | Peak Height |
|---|---|---|---|
| 30.33 | 6.55 | 584067704.98 | 34058095.58 |
| 31.02 | 5.25 | 468555343.40 | 54891177.90 |
| 31.50 | 5.61 | 500910266.69 | 31232160.66 |
| 36.91 | 1.40 | 125312088.17 | 8754720.60 |
| 37.01 | 0.72 | 64326592.51 | 8891984.85 |
| 37.92 | 1.06 | 94994526.59 | 10136166.31 |
| 39.57 | 1.05 | 93929956.13 | 10644957.61 |
| 39.69 | 0.68 | 60390158.40 | 9814241.73 |
| 40.08 | 0.52 | 46777010.70 | 6596041.33 |
| 40.46 | 0.63 | 55930215.01 | 11298453.64 |
| 40.54 | 0.87 | 77345236.71 | 11622705.05 |
| 41.09 | 1.18 | 105590743.78 | 15598963.81 |
| 41.27 | 0.44 | 39395259.98 | 6342001.02 |
| 41.33 | 0.43 | 38026269.76 | 6196843.74 |
| 42.19 | 1.49 | 132655279.81 | 18676194.18 |
| 43.93 | 2.17 | 193266188.79 | 18933808.14 |

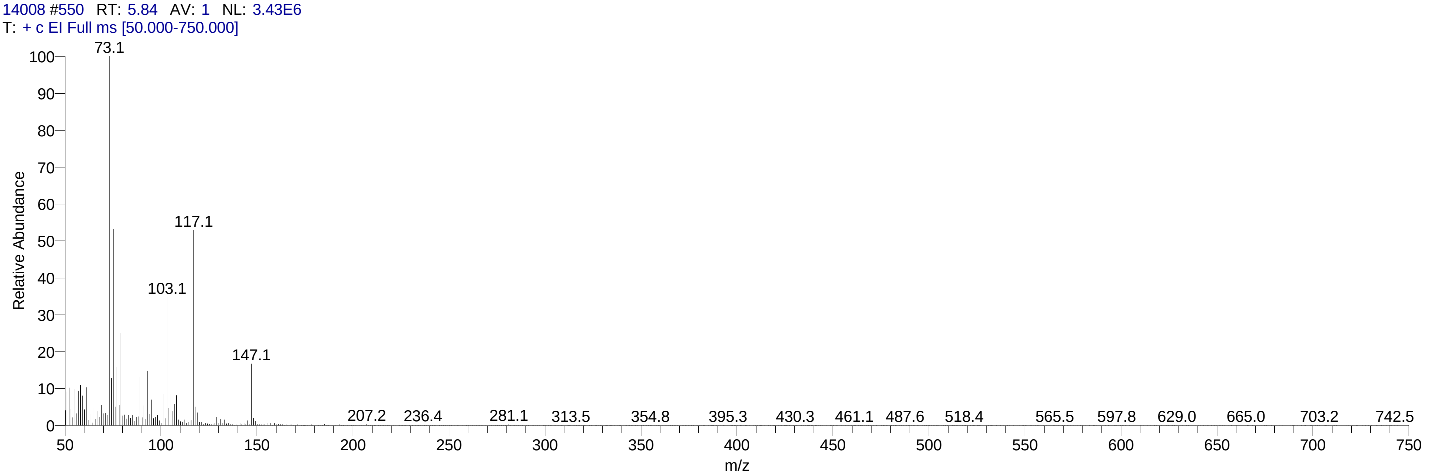


| RT | Compound Name | Area % | MF | Molecular Formula | Molecular Weight | Cas # | Library |
|---|---|---|---|---|---|---|---|
| 5.84 | Pregnane-3,20-diol, (3à,5á,20S)-, 2TMS derivative | 0.71 | 729 | C27H52O2Si2 | 464 | 16134-56-8 | replib |
| 5.84 | Ethyltriethylene glycol, TBDMS derivative | 0.71 | 764 | C14H32O4Si | 292 | NA | mainlib |
| 5.84 | Dimethyl(trimethylsilyl)ethoxysilane | 0.71 | 723 | C7H20OSi2 | 176 | 18297-47-7 | mainlib |
| 5.84 | 5,8,11-Eicosatrienoic acid, (Z)-, TMS derivative | 0.71 | 650 | C23H42O2Si | 378 | NA | mainlib |
| 5.84 | BUTANOIC ACID, 4-[(TRIMETHYLSILYL)OXY]-, TRIMETHYLSILYL ESTER | 0.71 | 716 | C10H24O3Si2 | 248 | 55133-95-4 | WileyRegistry8e |

| Compound Structure | Hit Spectrum |
|---|---|

Pregnane-3,20-diol, (3à,5á,20S)-, 2TMS derivative
Formula C27H52O2Si2, MW 464, CAS# 16134-56-8, Entry# 18229
Silane, [[(3à,5á,20S)-pregnane-3,20-diyl]bis(oxy)]bis[trimethyl-

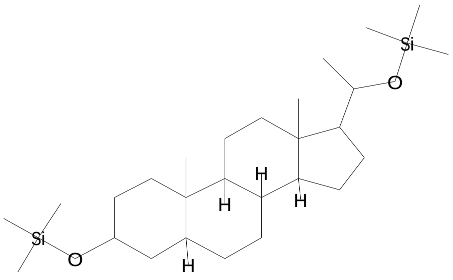


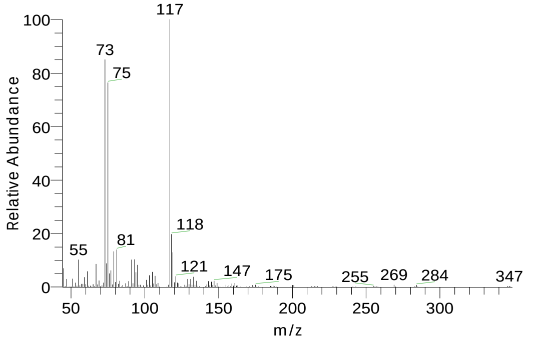

# *My GC-MS Report*

Compound Structure | Hit Spectrum

Ethyltriethylene glycol, TBDMS derivative
Formula C14H32O4Si, MW 292, CAS# NA, Entry# 40852
tert-Butyl-[2-[2-(2-ethoxyethoxy)ethoxy]ethoxy]dimethylsilane

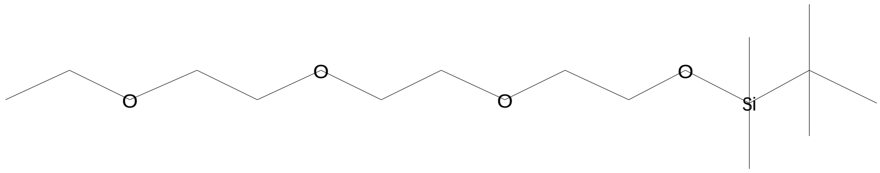

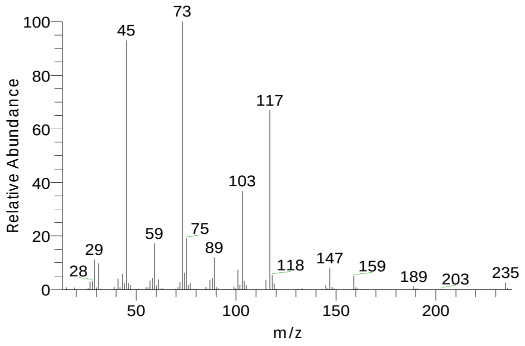


Dimethyl(trimethylsilyl)ethoxysilane
Formula C7H20OSi2, MW 176, CAS# 18297-47-7, Entry# 134908
1-Ethoxy-1,1,2,2,2-pentamethyldisilane #

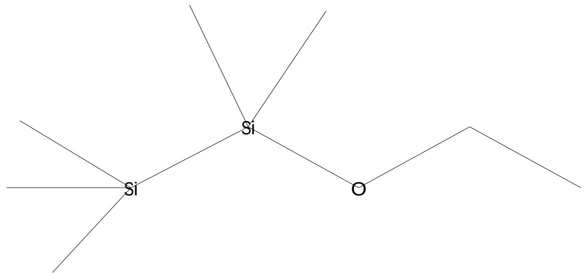

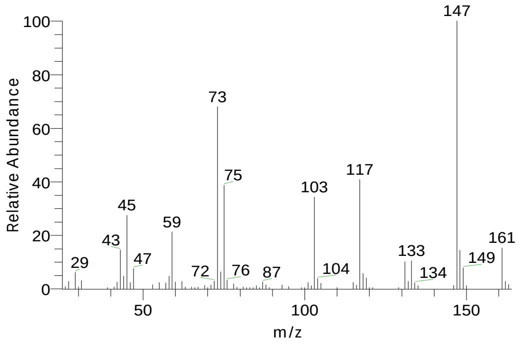


5,8,11-Eicosatrienoic acid, (Z)-, TMS derivative
Formula C23H42O2Si, MW 378, CAS# NA, Entry# 41326
cis-5,8,11-Eicosatrienoic acid, trimethylsilyl ester

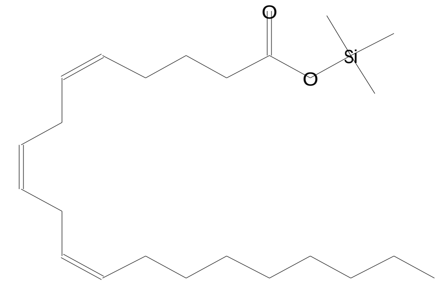

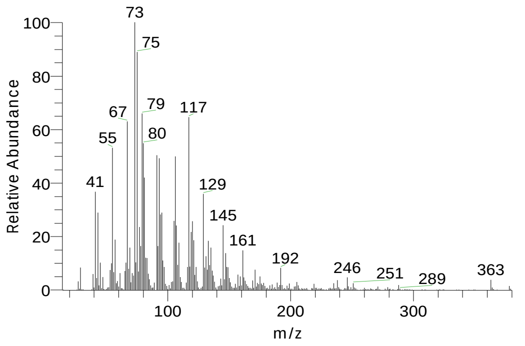


BUTANOIC ACID, 4-[(TRIMETHYLSILYL)OXY]-, TRIMETHYLSILYL ESTER
Formula C10H24O3Si2, MW 248, CAS# 55133-95-4, Entry# 137122
TRIMETHYLSILYL 4-[(TRIMETHYLSILYL)OXY]BUTANOATE #

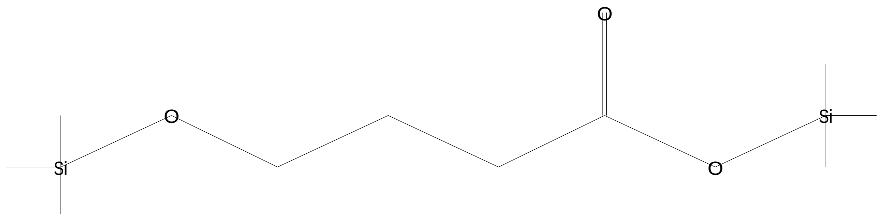

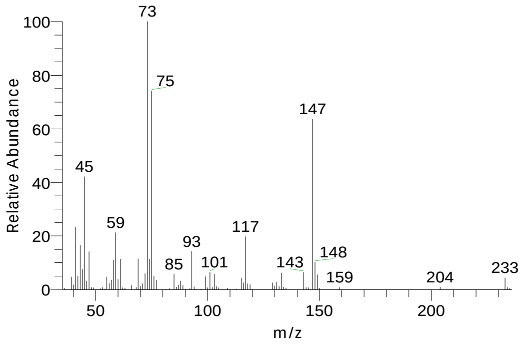

# My GC-MS Report

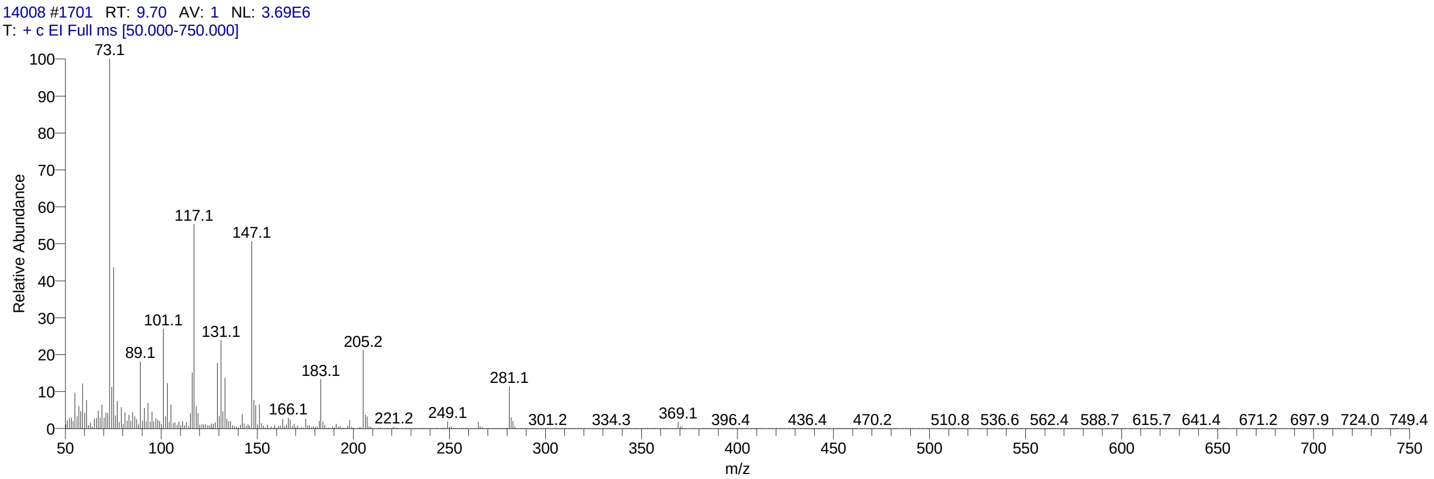


| RT | Compound Name | Area % | MF | Molecular Formula | Molecular Weight | Cas # | Library |
|---|---|---|---|---|---|---|---|
| 9.70 | 3,6-DIOXA-2,7-DISILAOCTANE, 2,2,4,7,7-PENTAMETHYL- | 0.86 | 780 | C9H24O2Si2 | 220 | 17887-27-3 | WileyRegistry8e |
| 9.70 | Propylene glycol, 2TMS derivative | 0.86 | 779 | C9H24O2Si2 | 220 | 17887-27-3 | replib |
| 9.70 | Silane, [(1-methoxy-1,3-propanediyl)bis(oxy)]bis[trimethyl- | 0.86 | 752 | C10H26O3Si2 | 250 | 62185-57-3 | mainlib |
| 9.70 | SILANE, [(2-METHOXY-1,3-PROPANEDIYL)BIS(OXY)]BIS[TRIMETHYL- | 0.86 | 752 | C10H26O3Si2 | 250 | 73639-55-1 | WileyRegistry8e |
| 9.70 | 3,6-DIOXA-2,7-DISILAOCTANE, 4-(METHOXYMETHYL)-2,2,7,7-TETRAMETHYL- | 0.86 | 752 | C10H26O3Si2 | 250 | NA | WileyRegistry8e |

| Compound Structure | Hit Spectrum |
|---|---|

3,6-DIOXA-2,7-DISILAOCTANE, 2,2,4,7,7-PENTAMETHYL-
Formula C9H24O2Si2, MW 220, CAS# 17887-27-3, Entry# 105960
2,2,4,7,7-PENTAMETHYL-3,6-DIOXA-2,7-DISILAOCTANE #

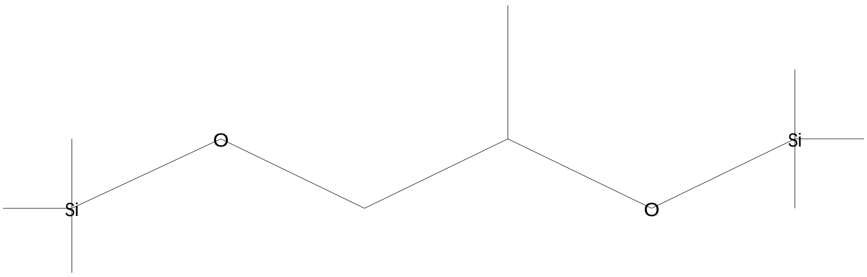


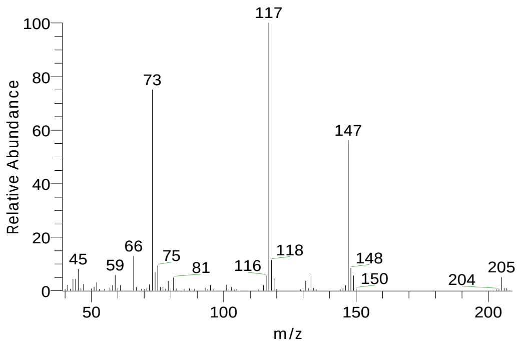


Propylene glycol, 2TMS derivative
Formula C9H24O2Si2, MW 220, CAS# 17887-27-3, Entry# 18232
3,6-Dioxa-2,7-disilaoctane, 2,2,4,7,7-pentamethyl-

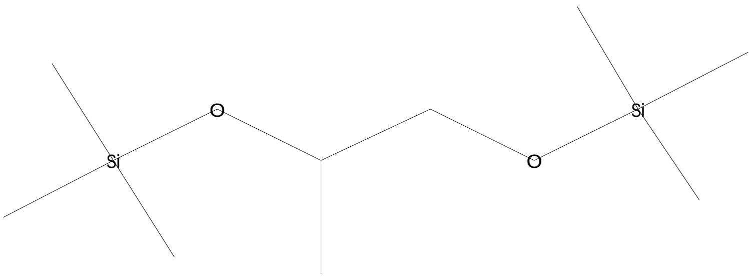


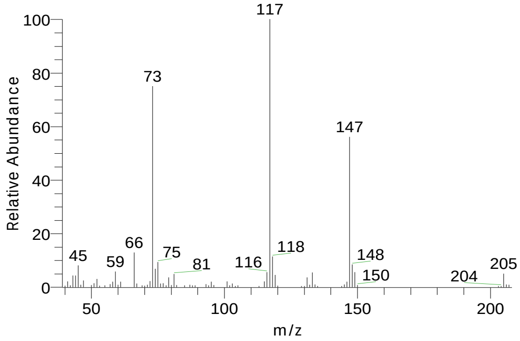

# *My GC-MS Report*

Compound Structure | Hit Spectrum

Silane, [(1-methoxy-1,3-propanediyl)bis(oxy)]bis[trimethyl-
Formula C10H26O3Si2, MW 250, CAS# 62185-57-3, Entry# 42666
Silane, [(1-methoxy-1,3-propanediyl)bis(oxy)]bis*trimethyl-

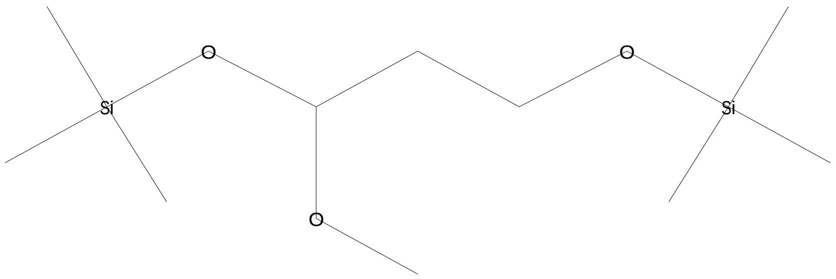


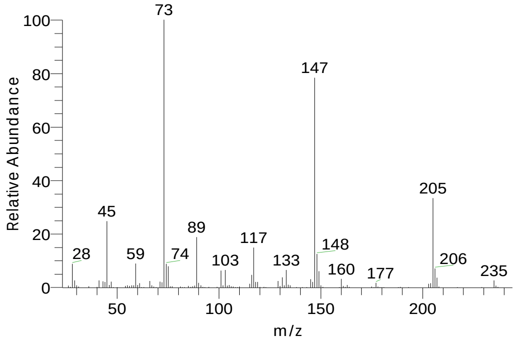


SILANE, [(2-METHOXY-1,3-PROPANEDIYL)BIS(OXY)]BIS[TRIMETHYL-
Formula C10H26O3Si2, MW 250, CAS# 73639-55-1, Entry# 139336
1,3-PROPANDIOL, 2-METHOXY-BIS-O-(TRIMETHYLSILYL)-

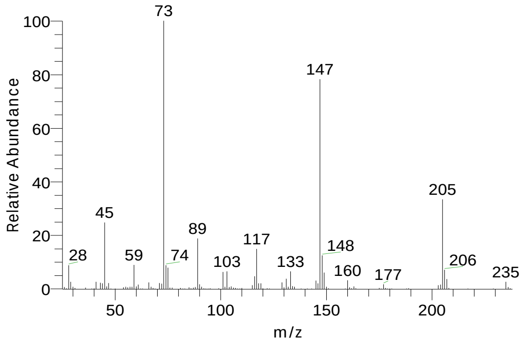


3,6-DIOXA-2,7-DISILAOCTANE, 4-(METHOXYMETHYL)-2,2,7,7-TETRAMETHYL-
Formula C10H26O3Si2, MW 250, CAS# NA, Entry# 393912
4-(METHOXYMETHYL)-2,2,7,7-TETRAMETHYL-3,6-DIOXA-2,7-DISILAOCTANE

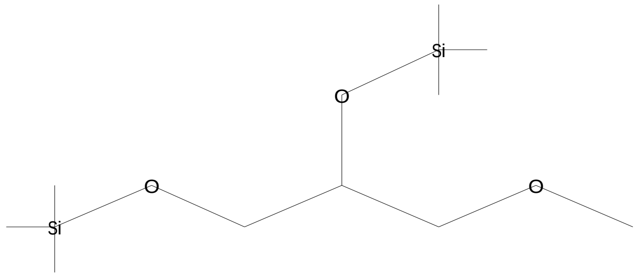


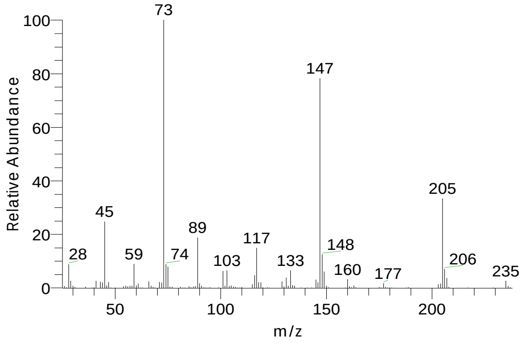


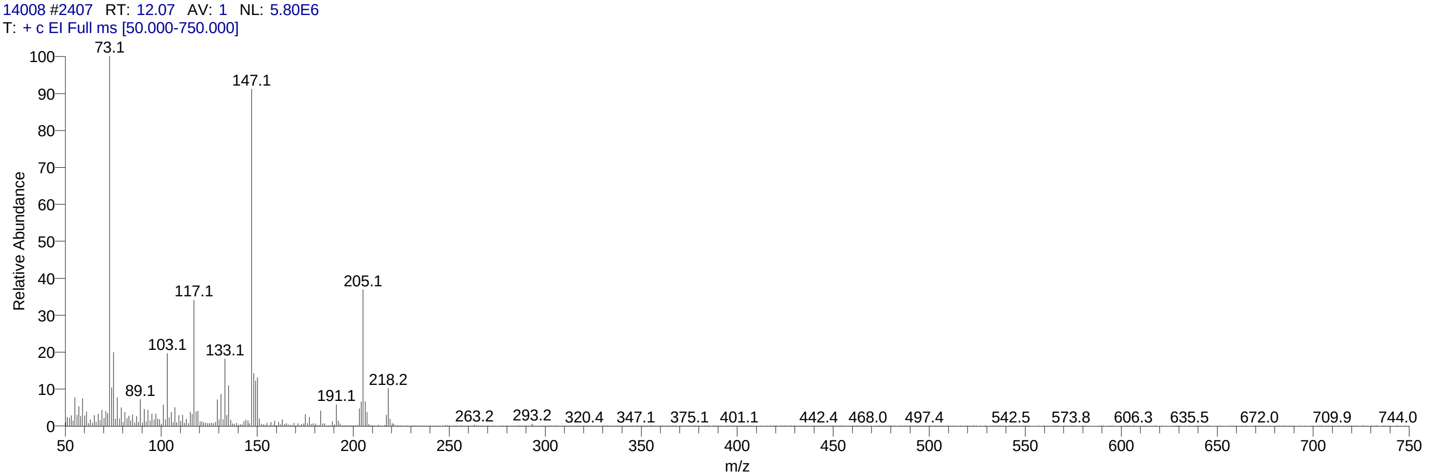


| RT | Compound Name | Area % | MF | Molecular Formula | Molecular Weight | Cas # | Library |
|---|---|---|---|---|---|---|---|
| 12.07 | Glycerol, 3TMS derivative | 2.27 | 845 | C12H32O3Si3 | 308 | 6787-10-6 | mainlib |
| 12.07 | Glycerol, 3TMS derivative | 2.27 | 834 | C12H32O3Si3 | 308 | 6787-10-6 | replib |

# *My GC-MS Report*

| RT | Compound Name | Area % | MF | Molecular Formula | Molecular Weight | Cas # | Library |
|---|---|---|---|---|---|---|---|
| 12.07 | 3,7-DIOXA-2,8-DISILANONANE, 2,2,8,8-TETRAMETHYL-5-[(TRIMETHYLSILYL)OXY]- | 2.27 | 847 | C12H32O3Si3 | 308 | 6787-10-6 | WileyRegistry8e |
| 12.07 | 3,7-DIOXA-2,8-DISILANONANE, 2,2,8,8-TETRAMETHYL-5-[(TRIMETHYLSILYL)OXY]- | 2.27 | 828 | C12H32O3Si3 | 308 | 6787-10-6 | WileyRegistry8e |
| 12.07 | 3,7-DIOXA-2,8-DISILANONANE, 2,2,8,8-TETRAMETHYL-5-[(TRIMETHYLSILYL)OXY]- | 2.27 | 806 | C12H32O3Si3 | 308 | 6787-10-6 | WileyRegistry8e |

Compound Structure | Hit Spectrum

Glycerol, 3TMS derivative
Formula C12H32O3Si3, MW 308, CAS# 6787-10-6, Entry# 42664
Glycerol, tris(trimethylsilyl) ether

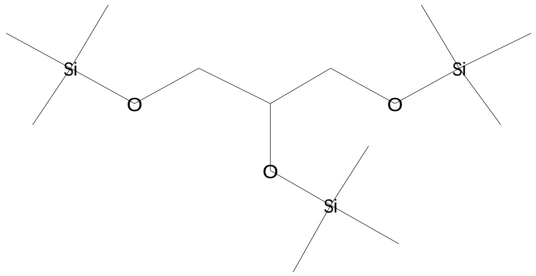


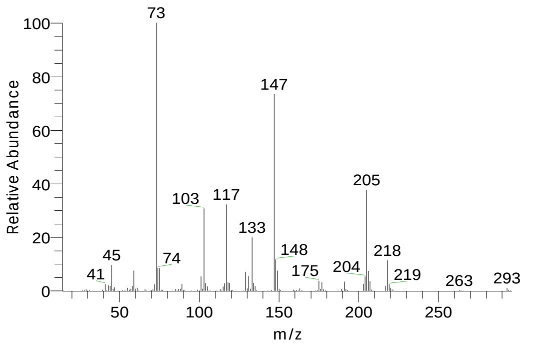


Glycerol, 3TMS derivative
Formula C12H32O3Si3, MW 308, CAS# 6787-10-6, Entry# 9974
Glycerol, tris(trimethylsilyl) ether

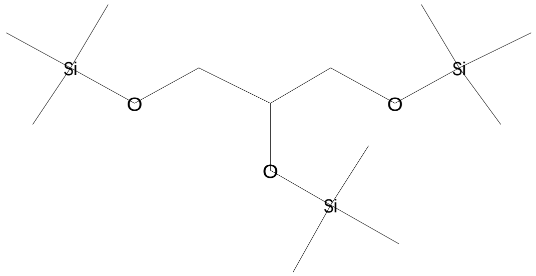


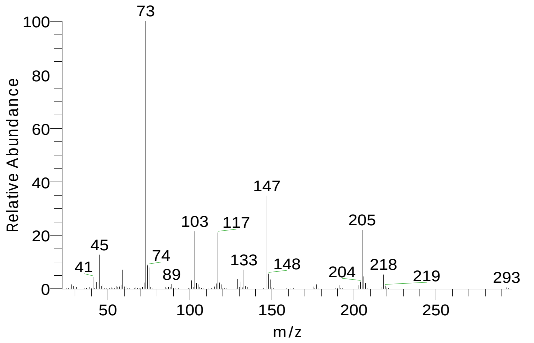


3,7-DIOXA-2,8-DISILANONANE, 2,2,8,8-TETRAMETHYL-5-[(TRIMETHYLSILYL)OXY]-
Formula C12H32O3Si3, MW 308, CAS# 6787-10-6, Entry# 382778
2,2,8,8-TETRAMETHYL-5-[(TRIMETHYLSILYL)OXY]-3,7-DIOXA-2,8-DISILANONANE #

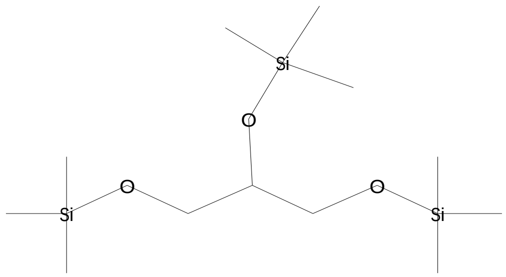


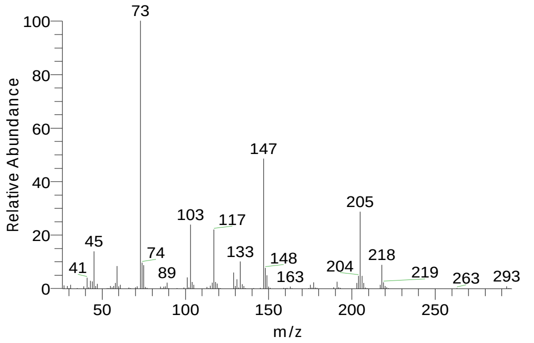


3,7-DIOXA-2,8-DISILANONANE, 2,2,8,8-TETRAMETHYL-5-[(TRIMETHYLSILYL)OXY]-
Formula C12H32O3Si3, MW 308, CAS# 6787-10-6, Entry# 195957
2,2,8,8-TETRAMETHYL-5-[(TRIMETHYLSILYL)OXY]-3,7-DIOXA-2,8-DISILANONANE #

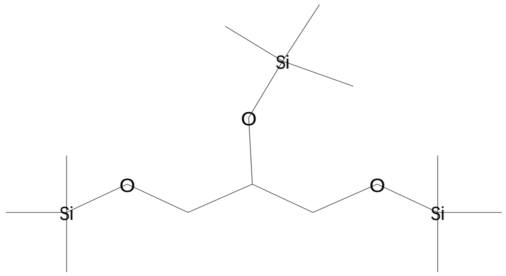


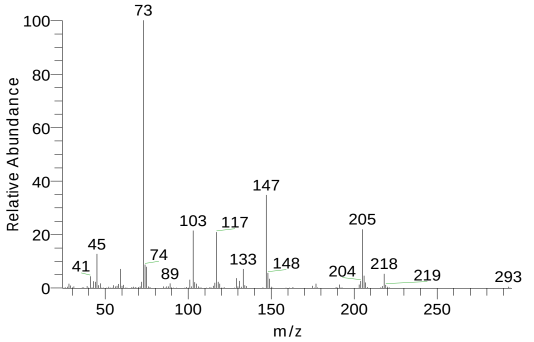

# My GC-MS Report

Compound Structure | Hit Spectrum

3,7-DIOXA-2,8-DISILANONANE, 2,2,8,8-TETRAMETHYL-5-[(TRIMETHYLSILYL)OXY]-
Formula C12H32O3Si3, MW 308, CAS# 6787-10-6, Entry# 195958
2,2,8,8-TETRAMETHYL-5-[(TRIMETHYLSILYL)OXY]-3,7-DIOXA-2,8-DISILANONANE #

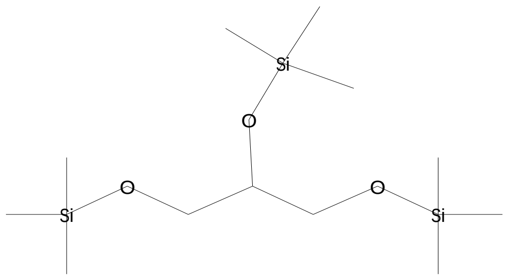


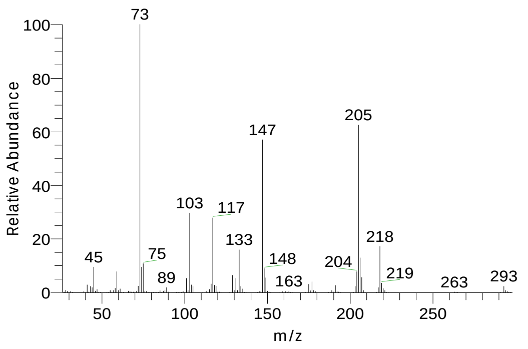


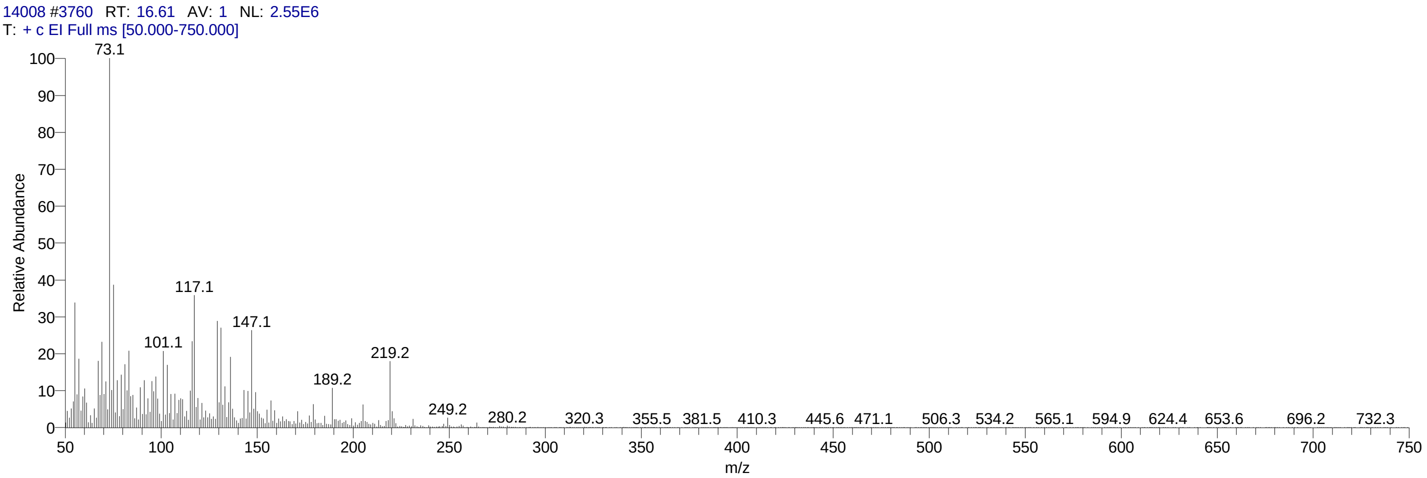


| RT | Compound Name | Area % | MF | Molecular Formula | Molecular Weight | Cas # | Library |
|---|---|---|---|---|---|---|---|
| 16.61 | TRISTRIMETHYLSILYL ETHER DERIVATIVE OF 1,25-DIHYDROXYVITAMIN D2 | 0.41 | 727 | C37H68O3Si3 | 644 | NA | WileyRegistry8e |
| 16.61 | 5,8,11-Eicosatrienoic acid, (Z)-, TMS derivative | 0.41 | 718 | C23H42O2Si | 378 | NA | mainlib |
| 16.61 | á-D-Galactopyranoside, methyl 2,6-bis-O-(trimethylsilyl)-, cyclic methylboronate | 0.41 | 711 | C14H31BO6Si2 | 362 | 56211-06-4 | mainlib |
| 16.61 | á-D-GALACTOPYRANOSIDE, METHYL 2,6-BIS-O-(TRIMETHYLSILYL)-, CYCLIC METHYLBORONATE | 0.41 | 711 | C14H31BO6Si2 | 362 | 56211-06-4 | WileyRegistry8e |
| 16.61 | à-D-GLUCOPYRANOSIDE, METHYL 2,3-BIS-O-(TRIMETHYLSILYL)-, CYCLIC METHYLBORONATE | 0.41 | 709 | C14H31BO6Si2 | 362 | 54400-90-7 | WileyRegistry8e |

Compound Structure | Hit Spectrum

TRISTRIMETHYLSILYL ETHER DERIVATIVE OF 1,25-DIHYDROXYVITAMIN D2
Formula C37H68O3Si3, MW 644, CAS# NA, Entry# 299431

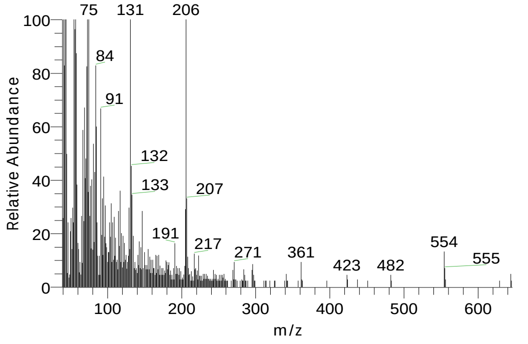

# *My GC-MS Report*

Compound Structure | Hit Spectrum

5,8,11-Eicosatrienoic acid, (Z)-, TMS derivative
Formula C23H42O2Si, MW 378, CAS# NA, Entry# 41326
cis-5,8,11-Eicosatrienoic acid, trimethylsilyl ester

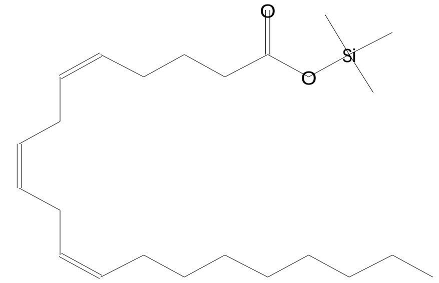

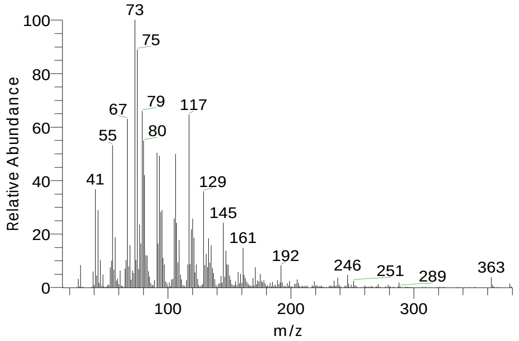


á-D-Galactopyranoside, methyl 2,6-bis-O-(trimethylsilyl)-, cyclic methylboronate
Formula C14H31BO6Si2, MW 362, CAS# 56211-06-4, Entry# 41379

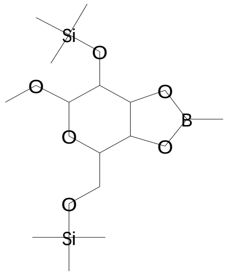

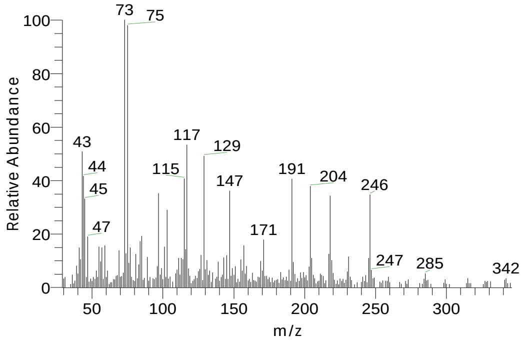


á-D-GALACTOPYRANOSIDE, METHYL 2,6-BIS-O-(TRIMETHYLSILYL)-, CYCLIC METHYLBORONATE
Formula C14H31BO6Si2, MW 362, CAS# 56211-06-4, Entry# 235850

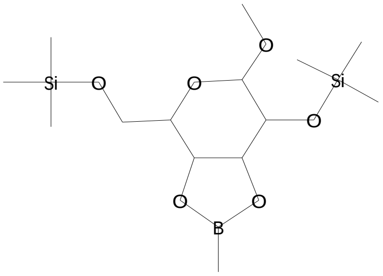

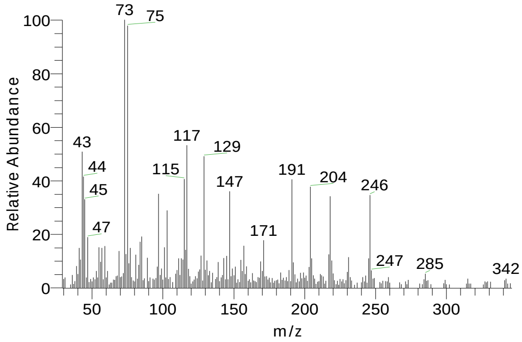


à-D-GLUCOPYRANOSIDE, METHYL 2,3-BIS-O-(TRIMETHYLSILYL)-, CYCLIC METHYLBORONATE
Formula C14H31BO6Si2, MW 362, CAS# 54400-90-7, Entry# 235856

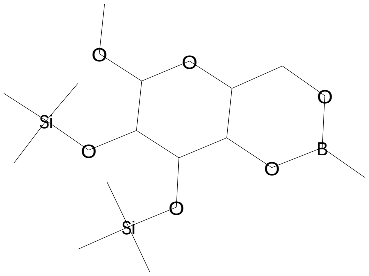

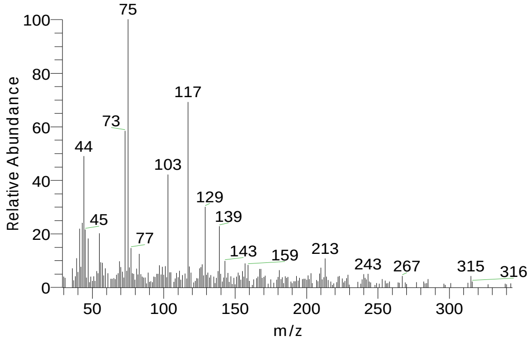

# My GC-MS Report

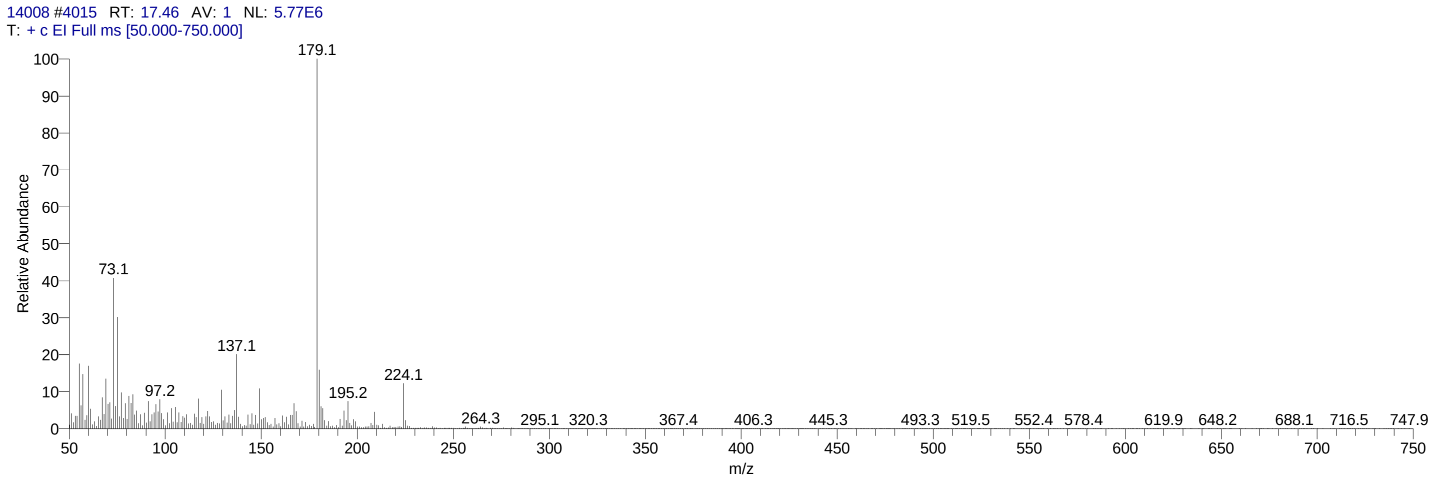


| RT | Compound Name | Area % | MF | Molecular Formula | Molecular Weight | Cas # | Library |
|---|---|---|---|---|---|---|---|
| 17.46 | 3-HYDROXY-2-METHYL-7-[(1E)-1-PROPENYL]-2,3-DIHYDRO-4H,5H-PYRANO[4,3-B]PYRAN-4,5-DIONE # | 1.34 | 718 | C12H12O5 | 236 | 1402-20-6 | WileyRegistry8e |
| 17.46 | 4H,5H-Pyrano(4,3-b)pyran-4,5-dione , 2,3-dihydro-3-à-hydroxy-2-á-methyl-7-propenyl- | 1.34 | 714 | C12H12O5 | 236 | 1402-20-6 | mainlib |
| 17.46 | à-D-MANNOPYRANOSIDE, METHYL, CYCLIC 2,3:4,6-BIS(METHYLBORONATE) | 1.34 | 686 | C9H16B2O6 | 242 | 54400-85-0 | WileyRegistry8e |
| 17.46 | 2-Heptanone, 6-(3-acetyl-2-methyl-1-cyclopropen-1-yl)-6-methyl- | 1.34 | 710 | C14H22O2 | 222 | 65868-86-2 | mainlib |
| 17.46 | 2-HEPTANONE, 6-(3-ACETYL-2-METHYL-1-CYCLOPROPEN-1-YL)-6-METHYL- | 1.34 | 710 | C14H22O2 | 222 | 65868-86-2 | WileyRegistry8e |

| Compound Structure | Hit Spectrum |
|---|---|

Formula C12H12O5, MW 236, CAS# 1402-20-6, Entry# 395252
2,3-DIHYDRO-3-à-HYDROXY-2-á-METHYL-7-PROPENYL-4H,5H-PYRANO(4,3-B)PYRAN-4,5-DIONE

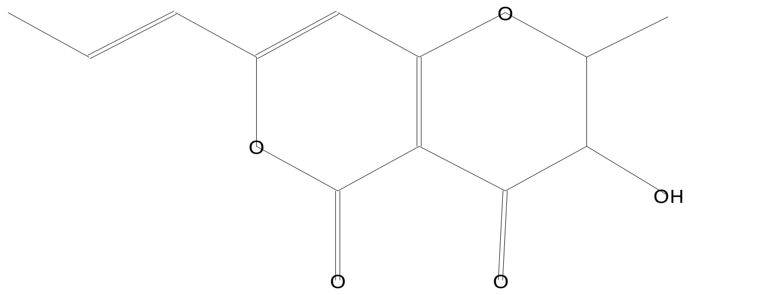


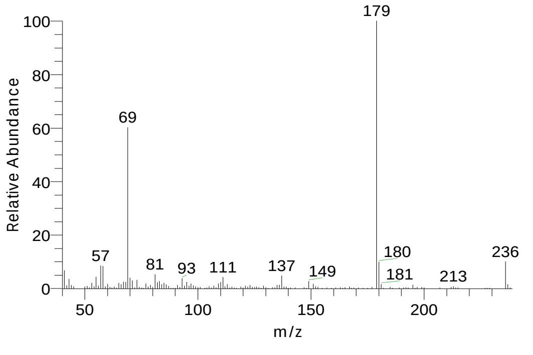


4H,5H-Pyrano(4,3-b)pyran-4,5-dione, 2,3-dihydro-3-à-hydroxy-2-á-methyl-7-propenyl-
Formula C12H12O5, MW 236, CAS# 1402-20-6, Entry# 166398
2,3-Dihydro-3-à-hydroxy-2-á-methyl-7-propenyl-4H,5H-pyrano(4,3-b)pyran-4,5-dione

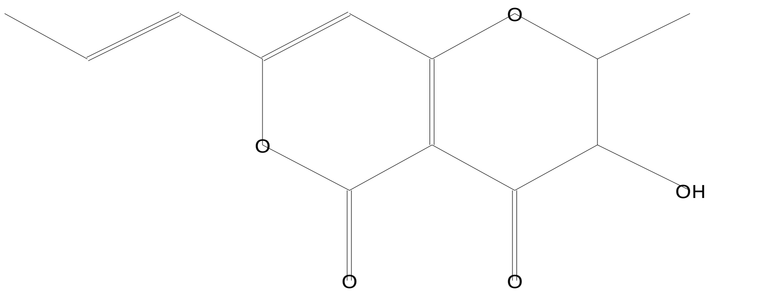


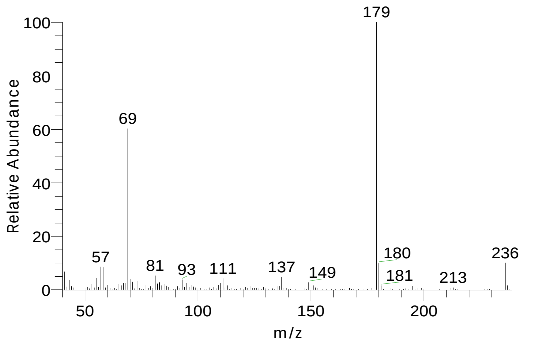

# My GC-MS Report

Compound Structure | Hit Spectrum

à-D-MANNOPYRANOSIDE, METHYL, CYCLIC 2,3:4,6-BIS(METHYLBORONATE)
Formula C9H16B2O6, MW 242, CAS# 54400-85-0, Entry# 131119
A-MANNOPYRANOSIDE-1-METHYL-2,3-4,6-DI-METHYLBORONATE

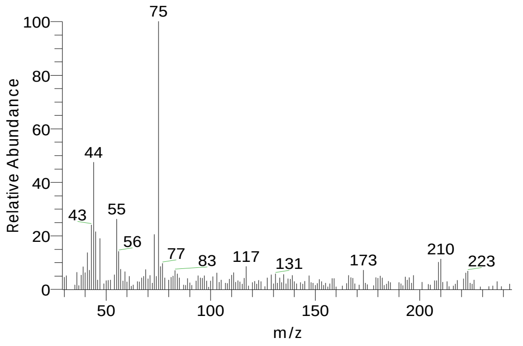


2-Heptanone, 6-(3-acetyl-2-methyl-1-cyclopropen-1-yl)-6-methyl-
Formula C14H22O2, MW 222, CAS# 65868-86-2, Entry# 13216
6-(3-Acetyl-2-methyl-1-cyclopropen-1-yl)-6-methyl-2-heptanone #

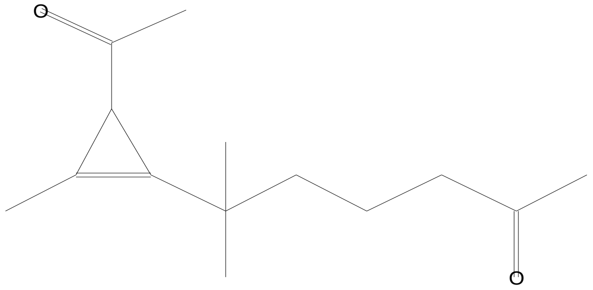


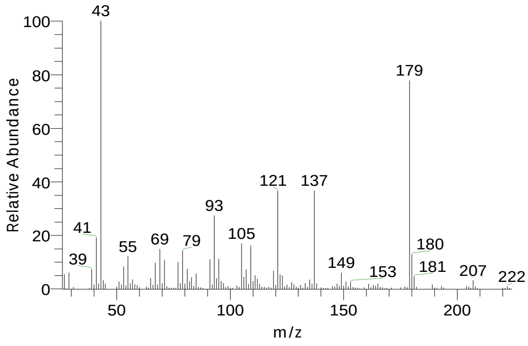


2-HEPTANONE, 6-(3-ACETYL-2-METHYL-1-CYCLOPROPEN-1-YL)-6-METHYL-
Formula C14H22O2, MW 222, CAS# 65868-86-2, Entry# 376659
6-(3-ACETYL-2-METHYL-1-CYCLOPROPEN-1-YL)-6-METHYL-2-HEPTANONE #

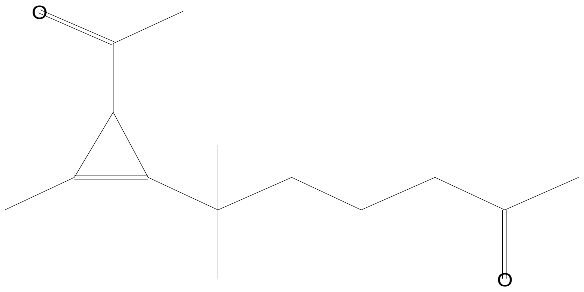


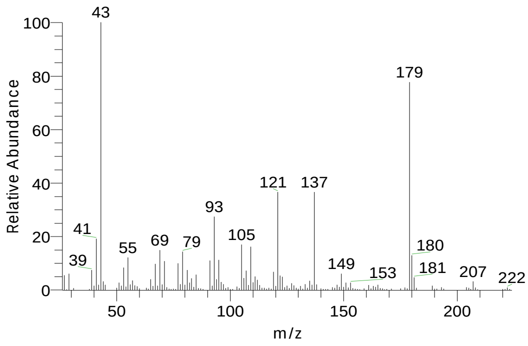


14008 #4162 RT: 17.96 AV: 1 NL: 5.32E6
T: + c EI Full ms [50.000-750.000]

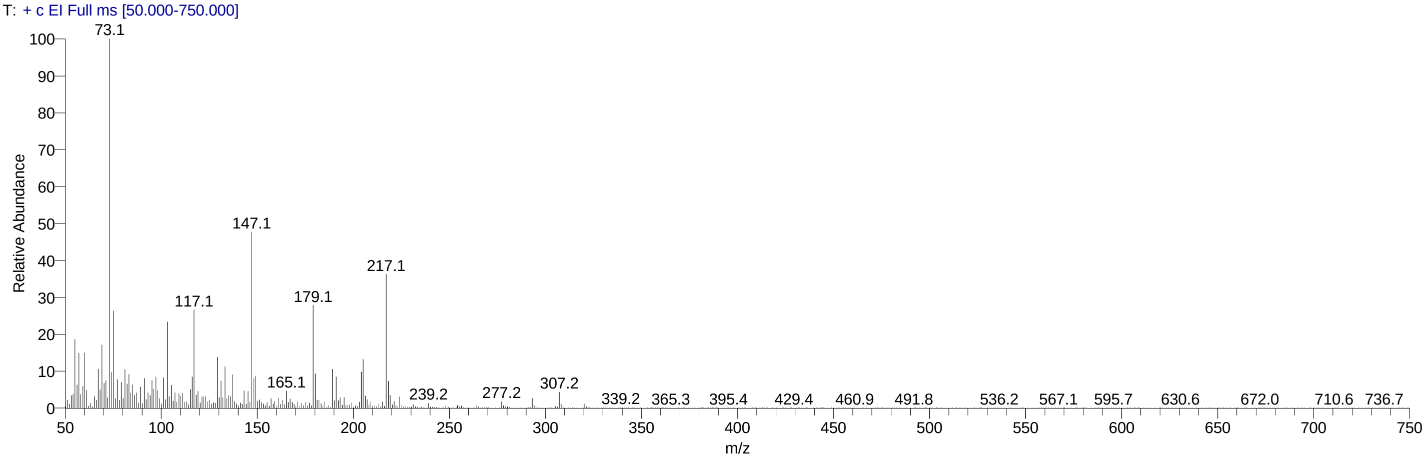


| RT | Compound Name | Area % | MF | Molecular Formula | Molecular Weight | Cas # | Library |
|---|---|---|---|---|---|---|---|
| 17.96 | BUTANAL, 2,3,4-TRIS[(TRIMETHYLSILYL)OXY]-, (R*,R*)- | 0.64 | 883 | C13H32O4Si3 | 336 | 56297-94-0 | WileyRegistry8e |
| 17.96 | L-Threitol, 4TMS derivative | 0.64 | 772 | C16H42O4Si4 | 410 | NA | mainlib |

# My GC-MS Report

| RT | Compound Name | Area % | MF | Molecular Formula | Molecular Weight | Cas # | Library |
|---|---|---|---|---|---|---|---|
| 17.96 | á-D-Galactopyranoside, methyl 2,3-bis-O-(trimethylsilyl)-, cyclic methylboronate | 0.64 | 701 | C14H31BO6Si2 | 362 | 56211-08-6 | mainlib |
| 17.96 | meso-Erythritol, 4TMS derivative | 0.64 | 770 | C16H42O4Si4 | 410 | NA | mainlib |
| 17.96 | á-D-GALACTOPYRANOSIDE, METHYL 2,3-BIS-O-(TRIMETHYLSILYL)-, CYCLIC METHYLBORONATE | 0.64 | 701 | C14H31BO6Si2 | 362 | 56211-08-6 | WileyRegistry8e |

Compound Structure | Hit Spectrum

BUTANAL, 2,3,4-TRIS[(TRIMETHYLSILYL)OXY]-, (R*,R*)-
Formula C13H32O4Si3, MW 336, CAS# 56297-94-0, Entry# 218633
2,3,4-TRIS[(TRIMETHYLSILYL)OXY]BUTANAL #

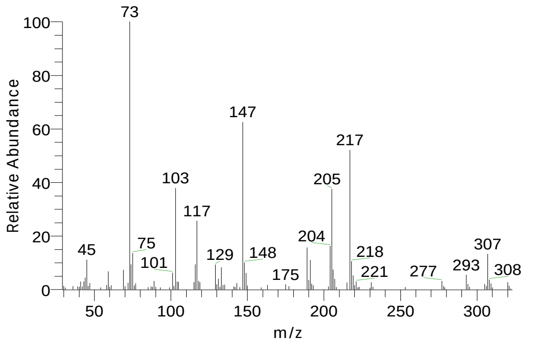


L-Threitol, 4TMS derivative
Formula C16H42O4Si4, MW 410, CAS# NA, Entry# 42676
L-Threitol, tetrakis(trimethylsilyl) ether

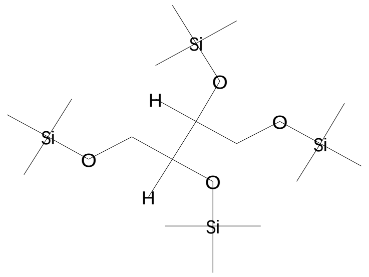


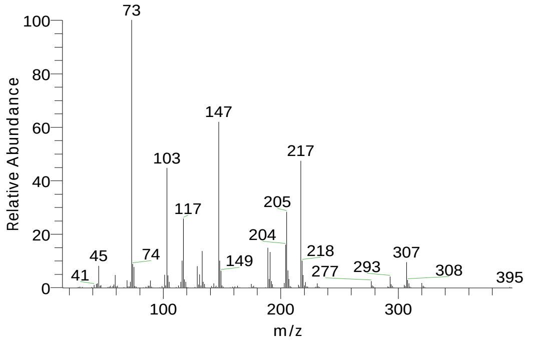


á-D-Galactopyranoside, methyl 2,3-bis-O-(trimethylsilyl)-, cyclic methylboronate
Formula C14H31BO6Si2, MW 362, CAS# 56211-08-6, Entry# 41509

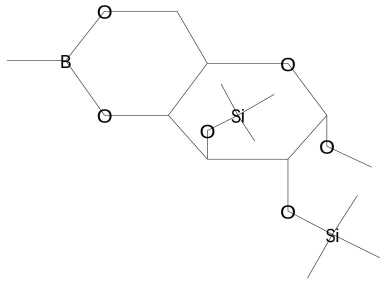


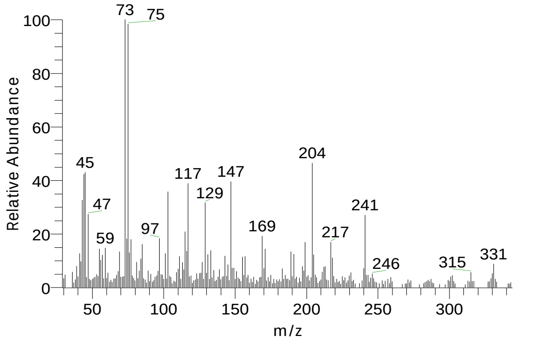


meso-Erythritol, 4TMS derivative
Formula C16H42O4Si4, MW 410, CAS# NA, Entry# 42678
meso-Erythritol, tetrakis(trimethylsilyl) ether

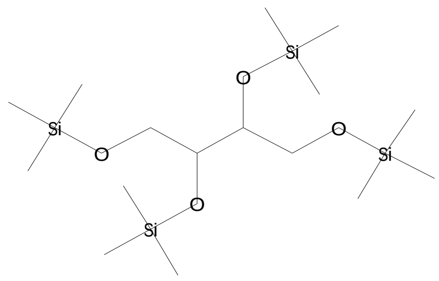


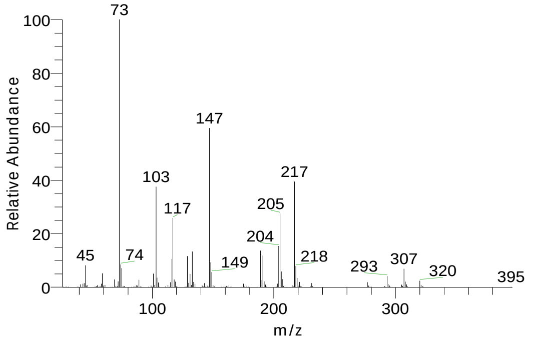

# My GC-MS Report

Compound Structure | Hit Spectrum

á-D-GALACTOPYRANOSIDE, METHYL 2,3-BIS-O-(TRIMETHYLSILYL)-, CYCLIC METHYLBORONATE
Formula C14H31BO6Si2, MW 362, CAS# 56211-08-6, Entry# 235854

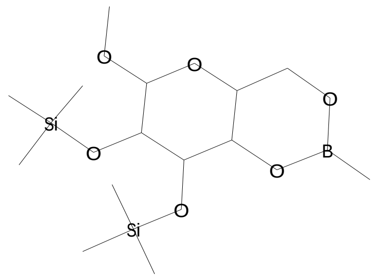


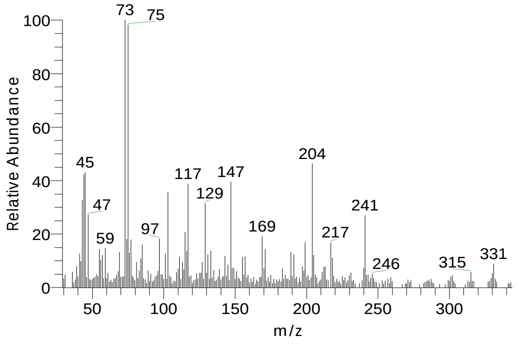


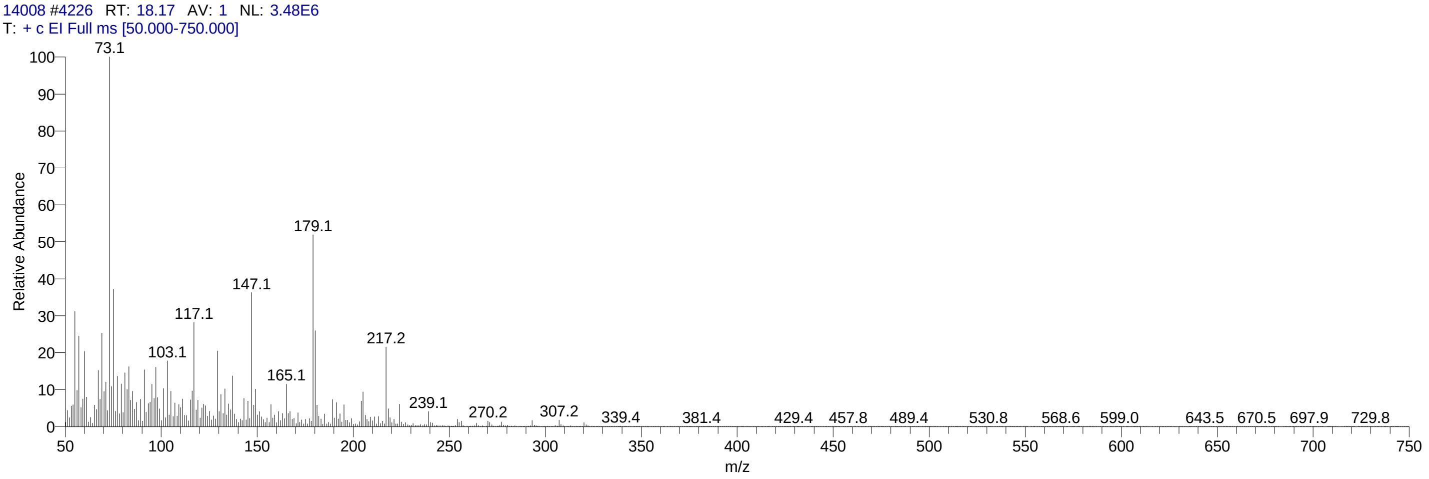


| RT | Compound Name | Area % | MF | Molecular Formula | Molecular Weight | Cas # | Library |
|---|---|---|---|---|---|---|---|
| 18.17 | à-D-GLUCOPYRANOSIDE, METHYL 2-(ACETYLAMINO)-2-DEOXY-3-O-(TRIMETHYLSILYL)-, CYCLIC METHYLBORONATE | 0.32 | 753 | C13H26BNO6Si | 331 | 54477-01-9 | WileyRegistry8e |
| 18.17 | á-D-Galactopyranoside, methyl 2,3-bis-O-(trimethylsilyl)-, cyclic butylboronate | 0.32 | 718 | C17H37BO6Si2 | 404 | 56211-10-0 | mainlib |
| 18.17 | á-D-GALACTOPYRANOSIDE, METHYL 2,3-BIS-O-(TRIMETHYLSILYL)-, CYCLIC BUTYLBORONATE | 0.32 | 718 | C17H37BO6Si2 | 404 | 56211-10-0 | WileyRegistry8e |
| 18.17 | á-D-Galactopyranoside, methyl 2,3-bis-O-(trimethylsilyl)-, cyclic methylboronate | 0.32 | 716 | C14H31BO6Si2 | 362 | 56211-08-6 | mainlib |
| 18.17 | á-D-GALACTOPYRANOSIDE, METHYL 2,3-BIS-O-(TRIMETHYLSILYL)-, CYCLIC METHYLBORONATE | 0.32 | 716 | C14H31BO6Si2 | 362 | 56211-08-6 | WileyRegistry8e |

# My GC-MS Report

Compound Structure | Hit Spectrum

Formula C13H26BNO6Si, MW 331, CAS# 54477-01-9, Entry# 215088

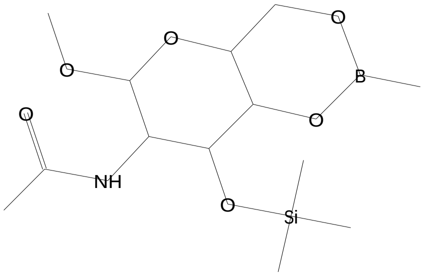


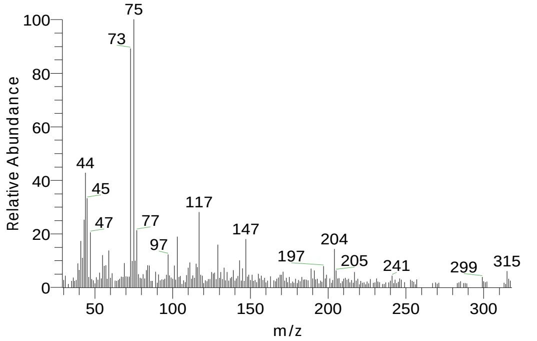


á-D-Galactopyranoside, methyl 2,3-bis-O-(trimethylsilyl)-, cyclic butylboronate
Formula C17H37BO6Si2, MW 404, CAS# 56211-10-0, Entry# 42381

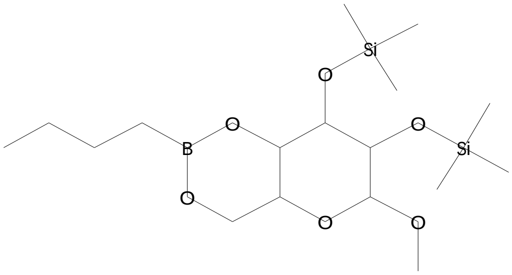


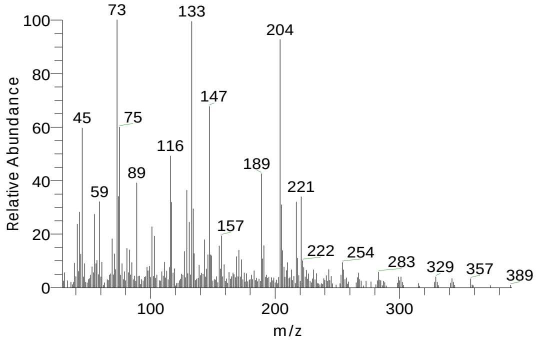


á-D-GALACTOPYRANOSIDE, METHYL 2,3-BIS-O-(TRIMETHYLSILYL)-, CYCLIC BUTYLBORONATE
Formula C17H37BO6Si2, MW 404, CAS# 56211-10-0, Entry# 257666

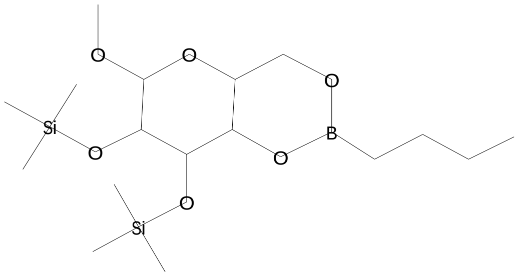


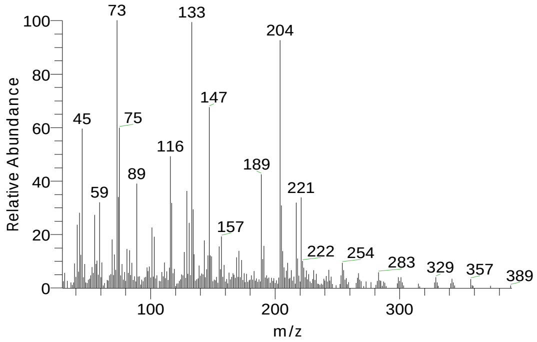


á-D-Galactopyranoside, methyl 2,3-bis-O-(trimethylsilyl)-, cyclic methylboronate
Formula C14H31BO6Si2, MW 362, CAS# 56211-08-6, Entry# 41509

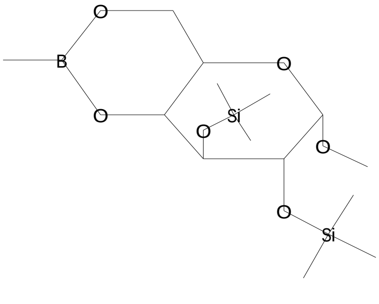


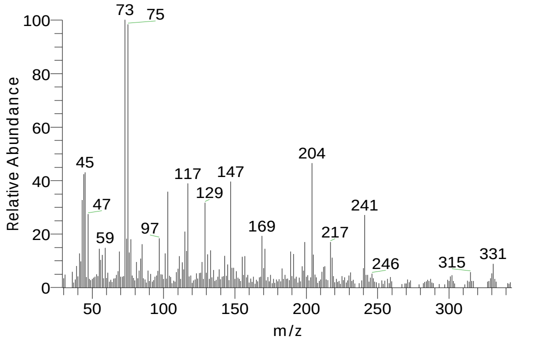

# My GC-MS Report

Compound Structure | Hit Spectrum

á-D-GALACTOPYRANOSIDE, METHYL 2,3-BIS-O-(TRIMETHYLSILYL)-, CYCLIC METHYLBORONATE
Formula C14H31BO6Si2, MW 362, CAS# 56211-08-6, Entry# 235854

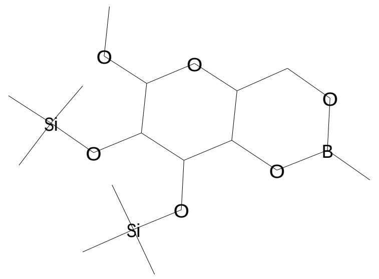


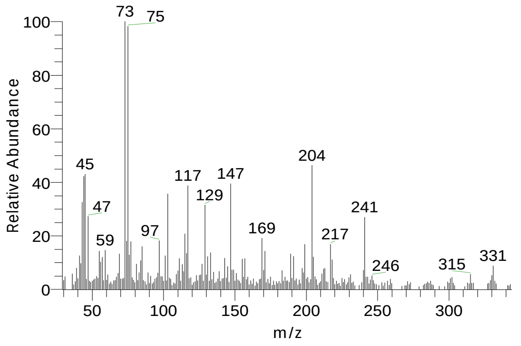


14008 #4273 RT: 18.33 AV: 1 NL: 7.07E6
T: + c EI Full ms [50.000-750.000]

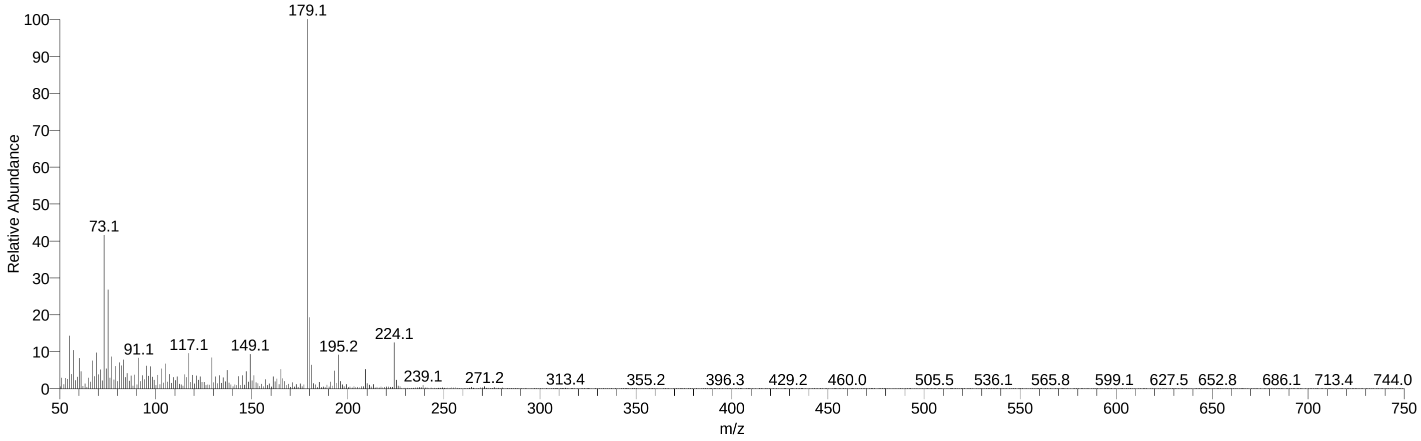


| RT | Compound Name | Area % | MF | Molecular Formula | Molecular Weight | Cas # | Library |
|---|---|---|---|---|---|---|---|
| 18.33 | 3-HYDROXY-2-METHYL-7-[(1E)-1-PROPENYL]-2,3-DIHYDRO-4H,5H-PYRANO[4,3-B]PYRAN-4,5-DIONE # | 1.31 | 729 | C12H12O5 | 236 | 1402-20-6 | WileyRegistry8e |
| 18.33 | 4H,5H-Pyrano(4,3-b)pyran-4,5-dione, 2,3-dihydro-3-à-hydroxy-2-á-methyl-7-propenyl- | 1.31 | 727 | C12H12O5 | 236 | 1402-20-6 | mainlib |
| 18.33 | 5,8,11,14-Eicosatetraynoic acid, TMS derivative | 1.31 | 654 | C23H32O2Si | 368 | NA | mainlib |
| 18.33 | à-D-MANNOPYRANOSIDE, METHYL, CYCLIC 2,3:4,6-BIS(METHYLBORONATE) | 1.31 | 677 | C9H16B2O6 | 242 | 54400-85-0 | WileyRegistry8e |
| 18.33 | Benzaldehyde, 2-hydroxy-4-methoxy-3,6-dimethyl- | 1.31 | 795 | C10H12O3 | 180 | 34883-15-3 | mainlib |

Compound Structure | Hit Spectrum

Formula C12H12O5, MW 236, CAS# 1402-20-6, Entry# 395252
2,3-DIHYDRO-3-à-HYDROXY-2-á-METHYL-7-PROPENYL-4H,5H-PYRANO(4,3-B)PYRAN-4,5-DIONE

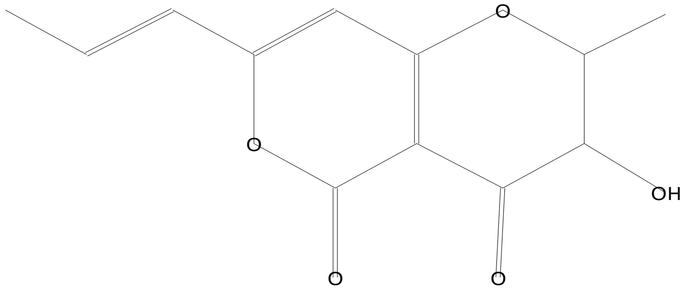


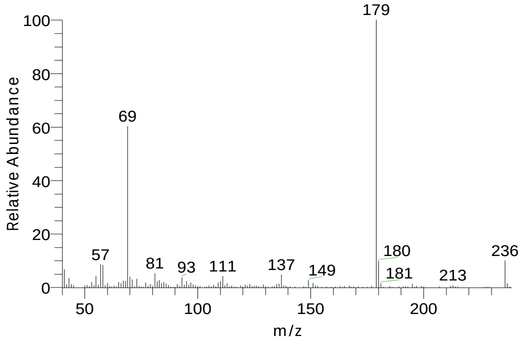

# My GC-MS Report

Compound Structure | Hit Spectrum

4H,5H-Pyrano(4,3-b)pyran-4,5-dione, 2,3-dihydro-3-à-hydroxy-2-á-methyl-7-propenyl-
Formula C12H12O5, MW 236, CAS# 1402-20-6, Entry# 166398
2,3-Dihydro-3-à-hydroxy-2-á-methyl-7-propenyl-4H,5H-pyrano(4,3-b)pyran-4,5-dione

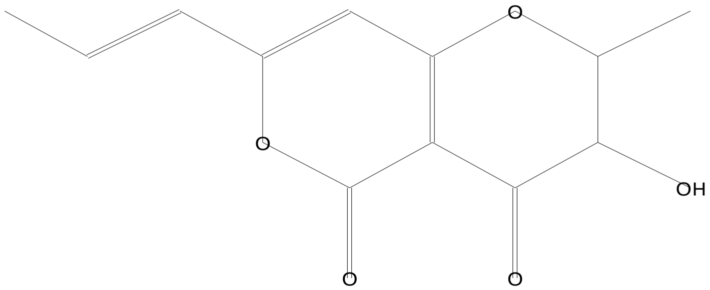


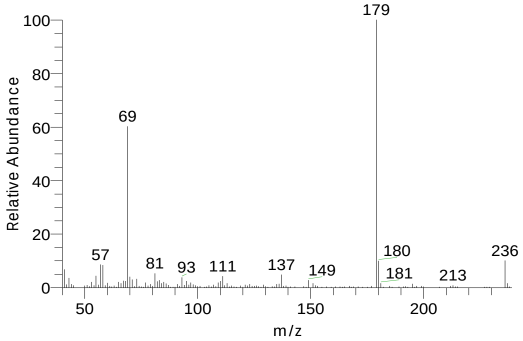


5,8,11,14-Eicosatetraynoic acid, TMS derivative
Formula C23H32O2Si, MW 368, CAS# NA, Entry# 41370
5,8,11,14-Eicosatetraynoic acid, trimethylsilyl ester

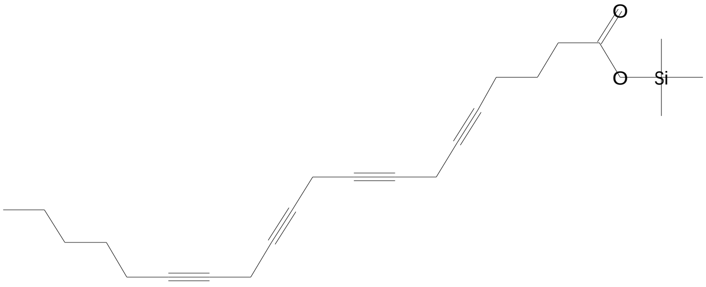


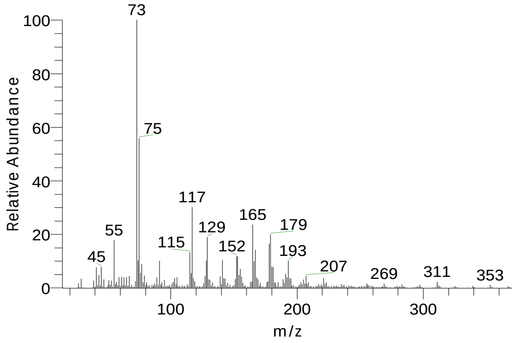


à-D-MANNOPYRANOSIDE, METHYL, CYCLIC 2,3:4,6-BIS(METHYLBORONATE)
Formula C9H16B2O6, MW 242, CAS# 54400-85-0, Entry# 131119
A-MANNOPYRANOSIDE-1-METHYL-2,3-4,6-DI-METHYLBORONATE

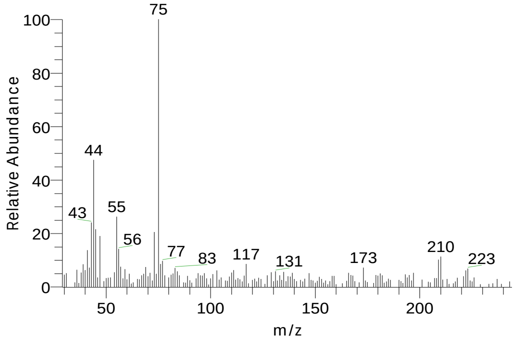


Benzaldehyde, 2-hydroxy-4-methoxy-3,6-dimethyl-
Formula C10H12O3, MW 180, CAS# 34883-15-3, Entry# 167458
2-Hydroxy-4-methoxy-3,6-dimethylbenzaldehyde #

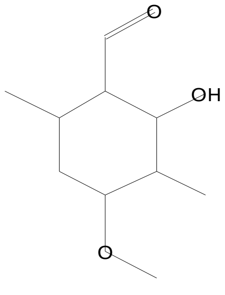


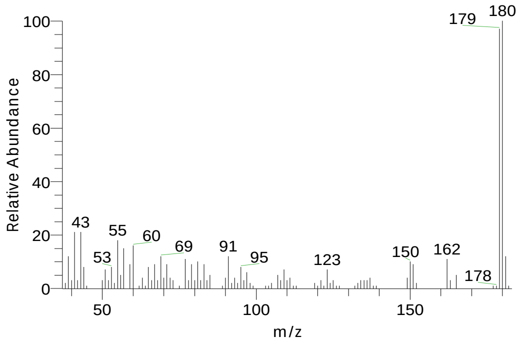

# My GC-MS Report

14008 #4534 RT: 19.20 AV: 1 NL: 3.93E6
T: + c EI Full ms [50.000-750.000]

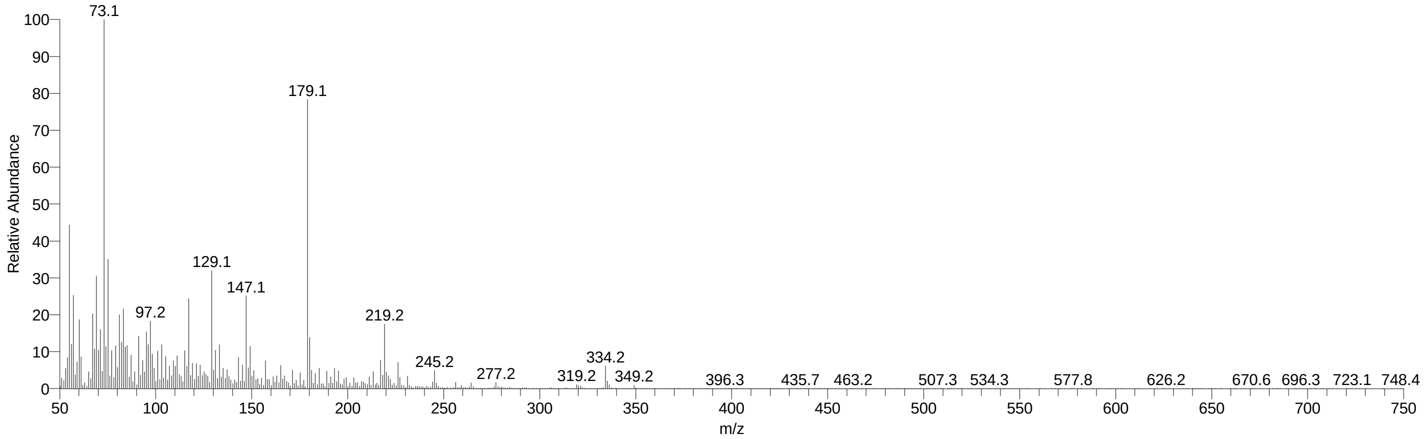


| RT | Compound Name | Area % | MF | Molecular Formula | Molecular Weight | Cas # | Library |
|---|---|---|---|---|---|---|---|
| 19.20 | á-D-Galactopyranoside, methyl 2,6-bis-O-(trimethylsilyl)-, cyclic methylboronate | 0.98 | 693 | C14H31BO6Si2 | 362 | 56211-06-4 | mainlib |
| 19.20 | á-D-GALACTOPYRANOSIDE, METHYL 2,6-BIS-O-(TRIMETHYLSILYL)-, CYCLIC METHYLBORONATE | 0.98 | 693 | C14H31BO6Si2 | 362 | 56211-06-4 | WileyRegistry8e |
| 19.20 | D-GLUCOSE, 6-O-à-D-GALACTOPYRANOSYL-, BIS-O-(TRIMETHYLSILYL) DERIV., CYCLIC TRIS(METHYLBORONATE) | 0.98 | 686 | C21H41B3O11Si2 | 558 | 72347-76-3 | WileyRegistry8e |
| 19.20 | 5,8,11-Eicosatriynoic acid, TMS derivative | 0.98 | 671 | C23H36O2Si | 372 | NA | mainlib |
| 19.20 | 5,8,11,14-Eicosatetraynoic acid, TMS derivative | 0.98 | 665 | C23H32O2Si | 368 | NA | mainlib |

Compound Structure | Hit Spectrum

á-D-Galactopyranoside, methyl 2,6-bis-O-(trimethylsilyl)-, cyclic methylboronate
Formula C14H31BO6Si2, MW 362, CAS# 56211-06-4, Entry# 41379

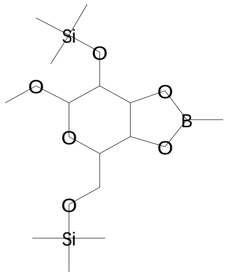


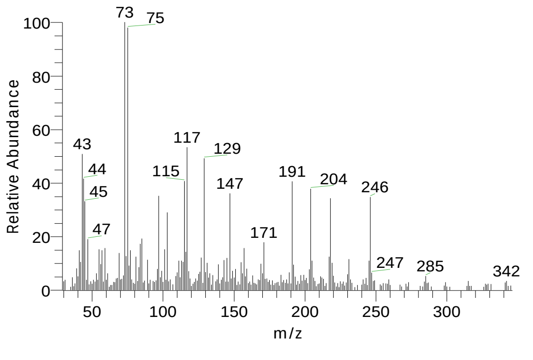


á-D-GALACTOPYRANOSIDE, METHYL 2,6-BIS-O-(TRIMETHYLSILYL)-, CYCLIC METHYLBORONATE
Formula C14H31BO6Si2, MW 362, CAS# 56211-06-4, Entry# 235850

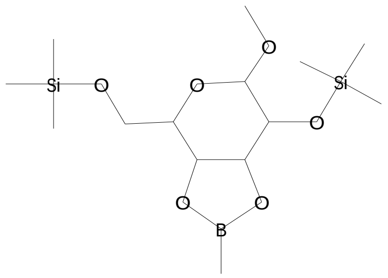


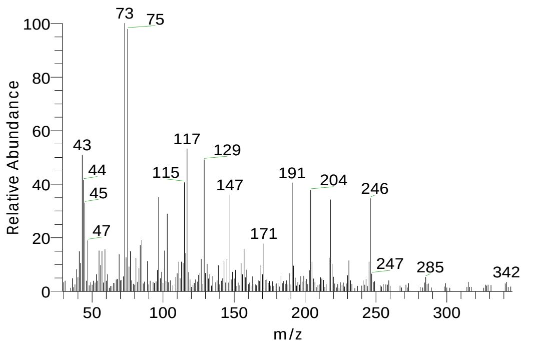

# *My GC-MS Report*

Compound Structure | Hit Spectrum

Formula C21H41B3O11Si2, MW 558, CAS# 72347-76-3, Entry# 293114

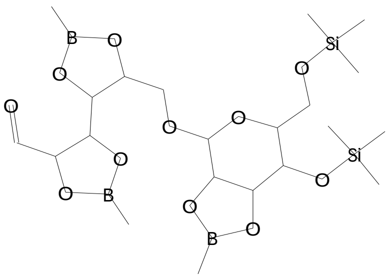


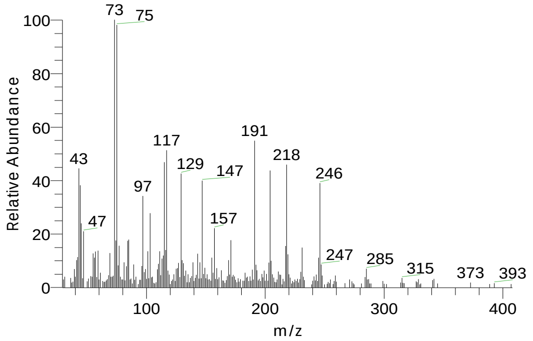


5,8,11-Eicosatriynoic acid, TMS derivative
Formula C23H36O2Si, MW 372, CAS# NA, Entry# 41372
5,8,11-Eicosatriynoic acid, trimethylsilyl ester

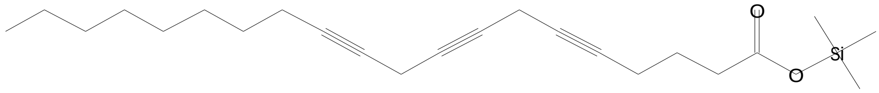


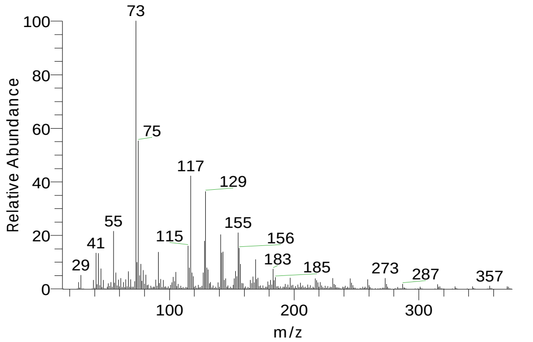


5,8,11,14-Eicosatetraynoic acid, TMS derivative
Formula C23H32O2Si, MW 368, CAS# NA, Entry# 41370
5,8,11,14-Eicosatetraynoic acid, trimethylsilyl ester

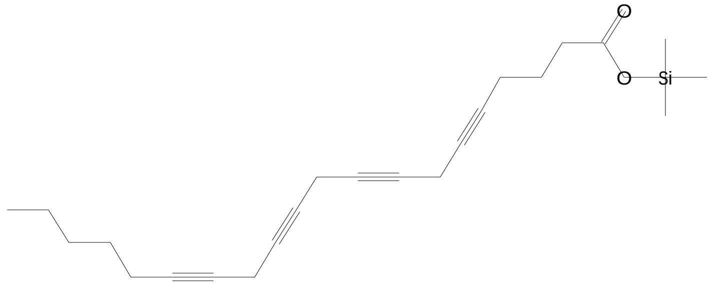


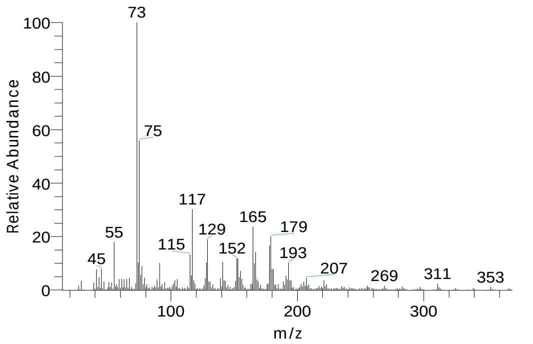


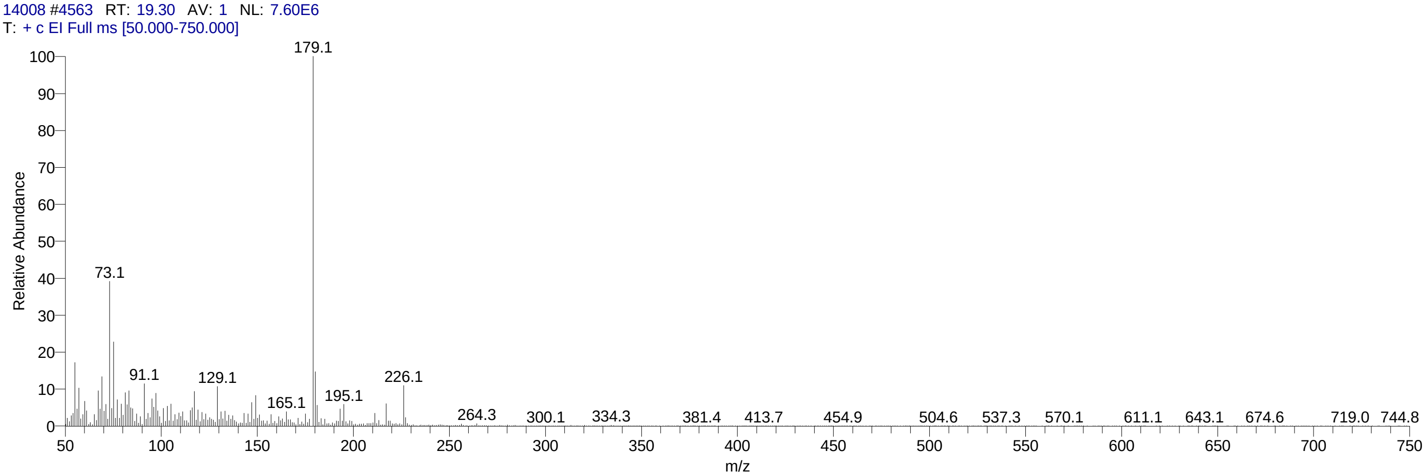


| RT | Compound Name | Area % | MF | Molecular Formula | Molecular Weight | Cas # | Library |
|---|---|---|---|---|---|---|---|
| 19.30 | 3-HYDROXY-2-METHYL-7-[(1E)-1-PROPENYL]-2,3-DIHYDRO-4H,5H-PYRANO[4,3-B]PYRAN-4,5-DIONE # | 1.93 | 727 | C12H12O5 | 236 | 1402-20-6 | WileyRegistry8e |

# *My GC-MS Report*

| RT | Compound Name | Area % | MF | Molecular Formula | Molecular Weight | Cas # | Library |
|---|---|---|---|---|---|---|---|
| 19.30 | 4H,5H-Pyrano(4,3-b)pyran-4,5-dione , 2,3-dihydro-3-à-hydroxy-2-á-methyl-7-propenyl- | 1.93 | 724 | C12H12O5 | 236 | 1402-20-6 | mainlib |
| 19.30 | á-D-GLUCOPYRANOSIDE, METHYL 2,3-BIS-O-(TRIMETHYLSILYL)-, CYCLIC METHYLBORONATE | 1.93 | 654 | C14H31BO6Si2 | 362 | 56211-07-5 | WileyRegistry8e |
| 19.30 | 5,8,11,14-Eicosatetraynoic acid, TMS derivative | 1.93 | 646 | C23H32O2Si | 368 | NA | mainlib |
| 19.30 | 10-Acetoxy-2-hydroxy-1,2,6a,6b,9,9,12a-heptamethyl-1,3,4,5,6,6a,6b,7,8,8a,9,10,11,12,12a,12b,13,14b-octadecahydro-2H-picene-4a-carboxylic acid, methyl ester | 1.93 | 642 | C33H52O5 | 528 | 14356-56-0 | mainlib |

| Compound Structure | Hit Spectrum |
|---|---|

Formula C12H12O5, MW 236, CAS# 1402-20-6, Entry# 395252
2,3-DIHYDRO-3-à-HYDROXY-2-á-METHYL-7-PROPENYL-4H,5H-PYRANO(4,3-B)PYRAN-4,5-DIONE

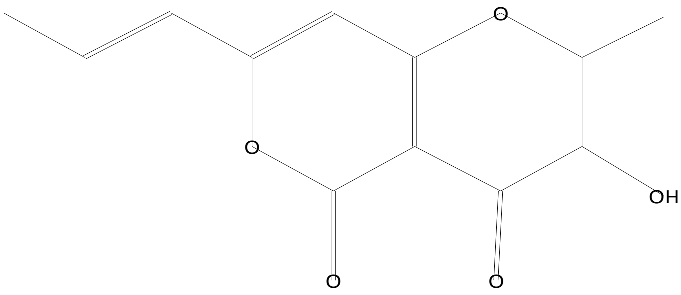


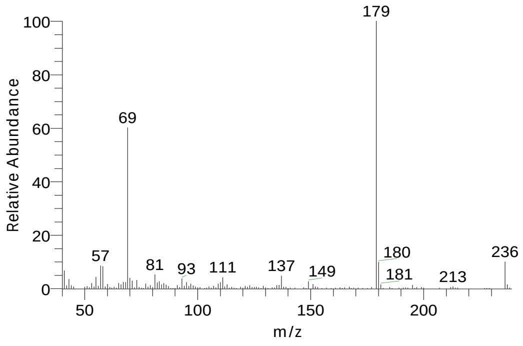


4H,5H-Pyrano(4,3-b)pyran-4,5-dione, 2,3-dihydro-3-à-hydroxy-2-á-methyl-7-propenyl-
Formula C12H12O5, MW 236, CAS# 1402-20-6, Entry# 166398
2,3-Dihydro-3-à-hydroxy-2-á-methyl-7-propenyl-4H,5H-pyrano(4,3-b)pyran-4,5-dione

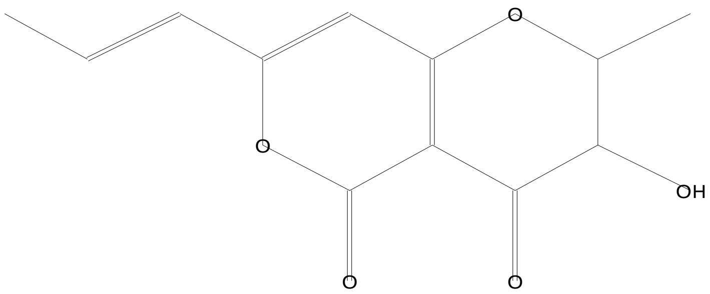


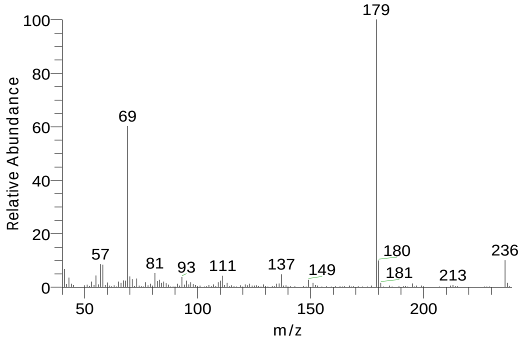


á-D-GLUCOPYRANOSIDE, METHYL 2,3-BIS-O-(TRIMETHYLSILYL)-, CYCLIC METHYLBORONATE
Formula C14H31BO6Si2, MW 362, CAS# 56211-07-5, Entry# 235857

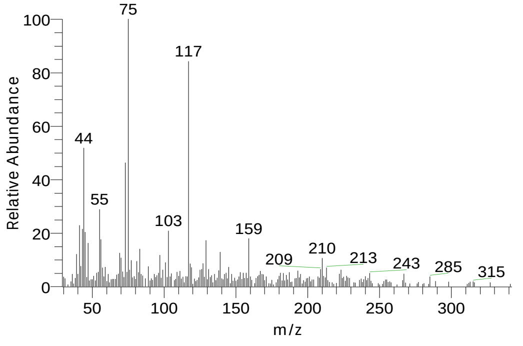

# *My GC-MS Report*

Compound Structure | Hit Spectrum

5,8,11,14-Eicosatetraynoic acid, TMS derivative
Formula C23H32O2Si, MW 368, CAS# NA, Entry# 41370
5,8,11,14-Eicosatetraynoic acid, trimethylsilyl ester

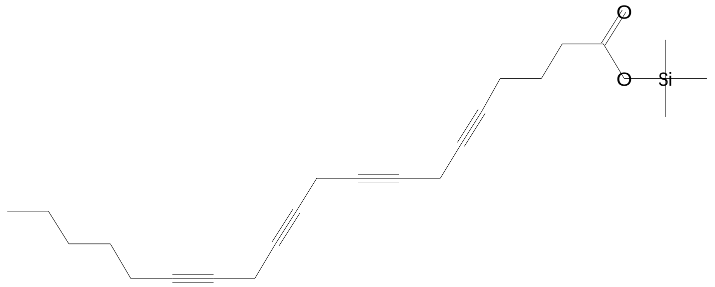


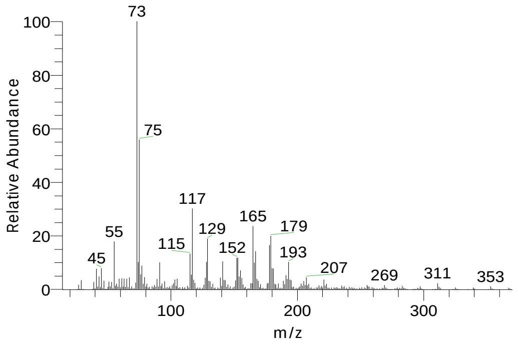


Formula C33H52O5, MW 528, CAS# 14356-56-0, Entry# 7689
Methyl 3-(acetyloxy)-20-hydroxyurs-12-en-28-oate #

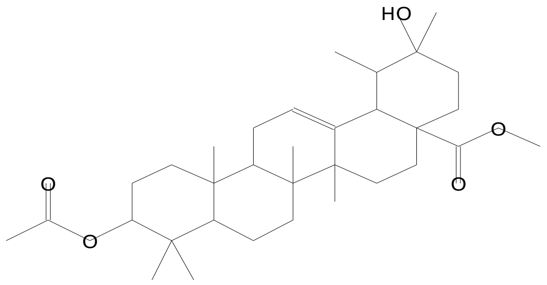


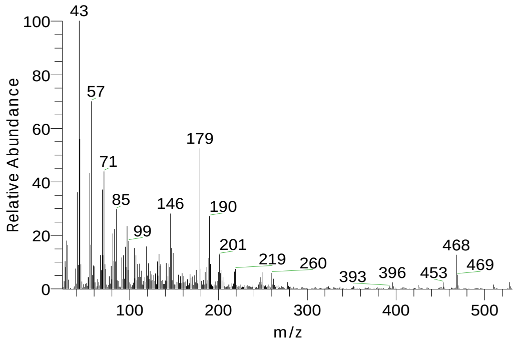


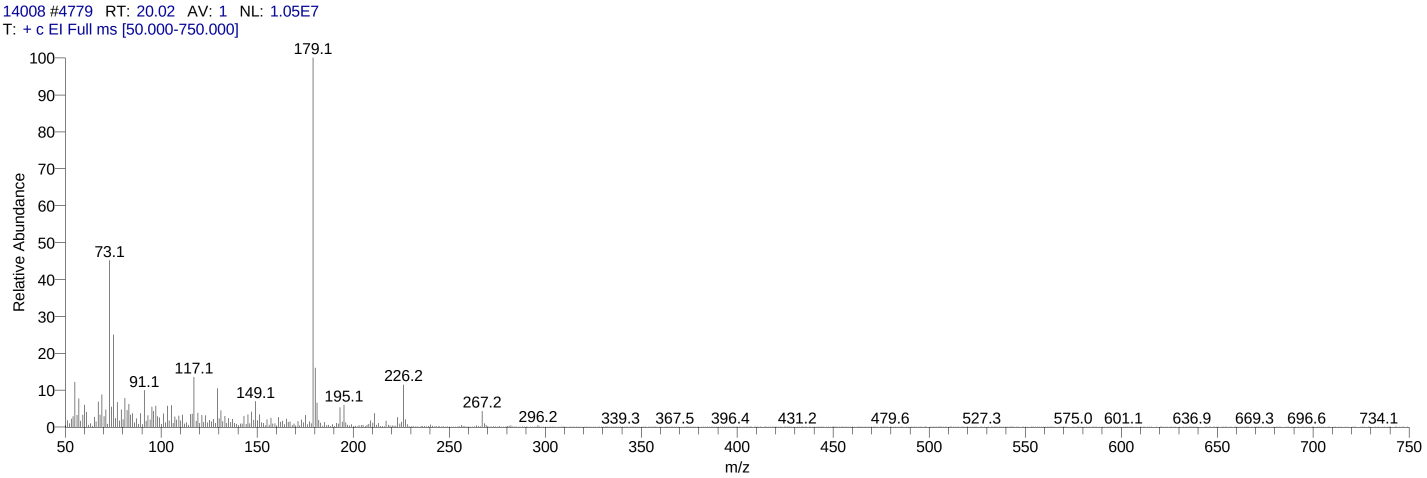


| RT | Compound Name | Area % | MF | Molecular Formula | Molecular Weight | Cas # | Library |
|---|---|---|---|---|---|---|---|
| 20.02 | 1,2-BIS(TRIMETHYLSILYOXY)-4-TRIMETHYLSILYLOXYMETHYLBENZENE | 0.69 | 659 | C16H32O3Si3 | 356 | NA | WileyRegistry8e |
| 20.02 | á-D-GLUCOPYRANOSIDE, METHYL 2,3-BIS-O-(TRIMETHYLSILYL)-, CYCLIC METHYLBORONATE | 0.69 | 652 | C14H31BO6Si2 | 362 | 56211-07-5 | WileyRegistry8e |
| 20.02 | 5,8,11,14-Eicosatetraynoic acid, TMS derivative | 0.69 | 645 | C23H32O2Si | 368 | NA | mainlib |
| 20.02 | Myrtenoic acid, trimethylsilyl ester | 0.69 | 732 | C13H22O2Si | 238 | NA | mainlib |
| 20.02 | MYRTENSAEURE, TRIMETHYLSILYLESTER | 0.69 | 732 | C13H22O2Si | 238 | NA | WileyRegistry8e |

# *My GC-MS Report*

Compound Structure | Hit Spectrum

1,2-BIS(TRIMETHYLSILYOXY)-4-TRIMETHYLSILYLOXYMETHYLBENZENE
Formula C16H32O3Si3, MW 356, CAS# NA, Entry# 232178

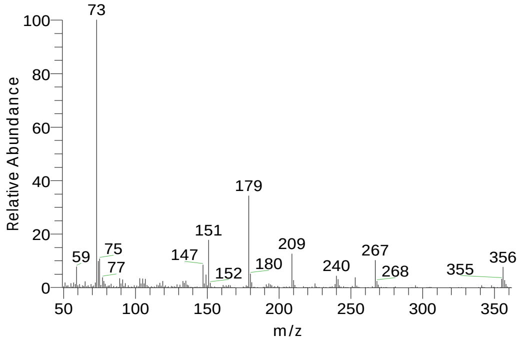


á-D-GLUCOPYRANOSIDE, METHYL 2,3-BIS-O-(TRIMETHYLSILYL)-, CYCLIC METHYLBORONATE
Formula C14H31BO6Si2, MW 362, CAS# 56211-07-5, Entry# 235857

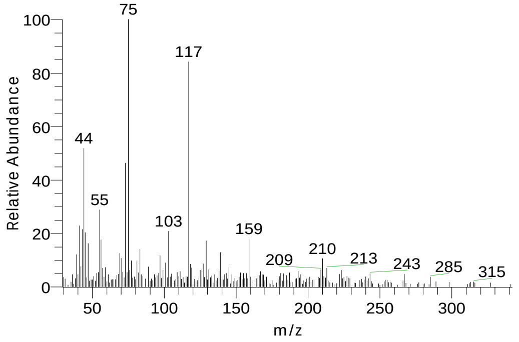


5,8,11,14-Eicosatetraynoic acid, TMS derivative
Formula C23H32O2Si, MW 368, CAS# NA, Entry# 41370
5,8,11,14-Eicosatetraynoic acid, trimethylsilyl ester

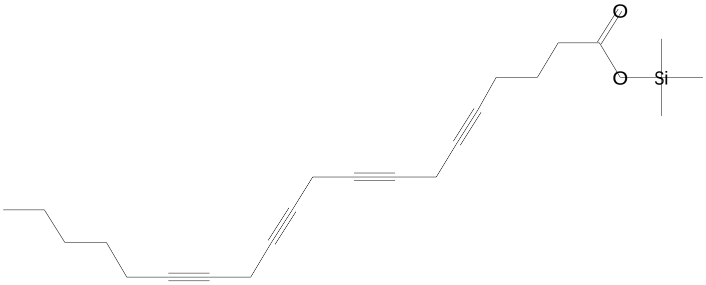


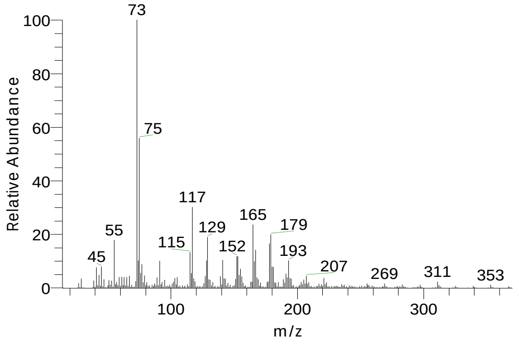


Myrtenoic acid, trimethylsilyl ester
Formula C13H22O2Si, MW 238, CAS# NA, Entry# 43024
Trimethylsilyl 6,6-dimethylbicyclo[3.1.1]hept-2-ene-2-carboxylate #

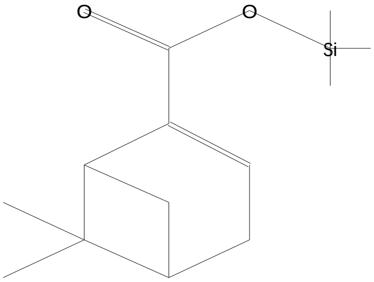


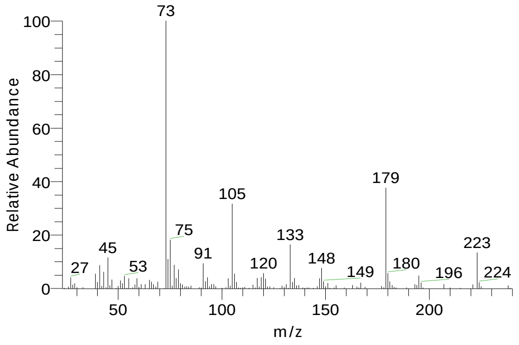

# My GC-MS Report

Compound Structure | Hit Spectrum

MYRTENSAEURE, TRIMETHYLSILYLESTER
Formula C13H22O2Si, MW 238, CAS# NA, Entry# 380344
TRIMETHYLSILYL 6,6-DIMETHYLBICYCLO[3.1.1]HEPT-2-ENE-2-CARBOXYLATE

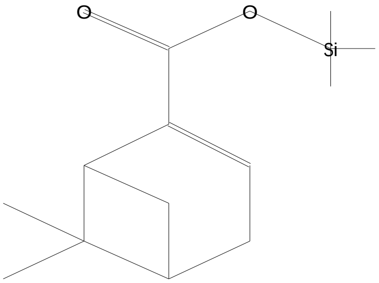


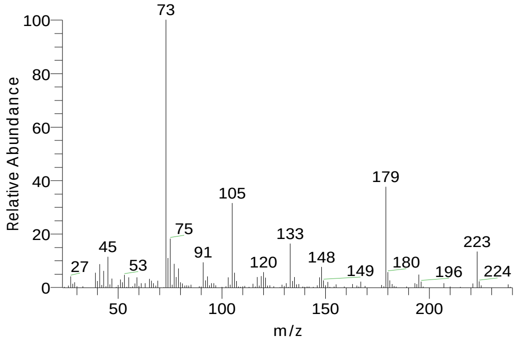


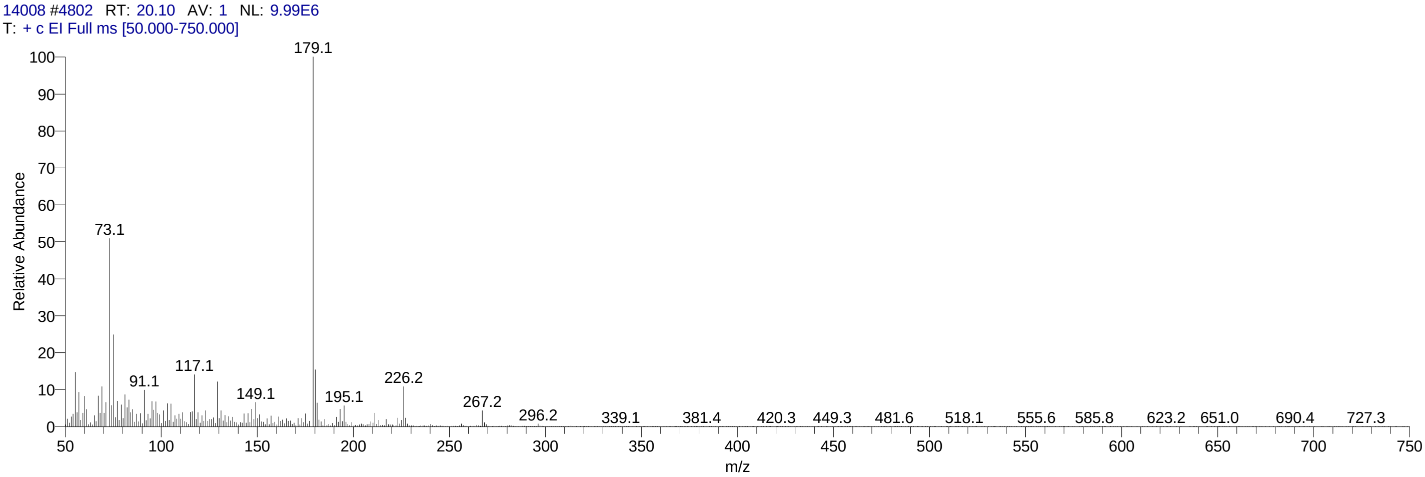


| RT | Compound Name | Area % | MF | Molecular Formula | Molecular Weight | Cas # | Library |
|---|---|---|---|---|---|---|---|
| 20.10 | á-D-GLUCOPYRANOSIDE, METHYL 2,3-BIS-O-(TRIMETHYLSILYL)-, CYCLIC METHYLBORONATE | 0.93 | 658 | C14H31BO6Si2 | 362 | 56211-07-5 | WileyRegistry8e |
| 20.10 | à-D-GLUCOPYRANOSIDE, METHYL 2-(ACETYLAMINO)-2-DEOXY-3-O-(TRIMETHYLSILYL)-, CYCLIC METHYLBORONATE | 0.93 | 656 | C13H26BNO6Si | 331 | 54477-01-9 | WileyRegistry8e |
| 20.10 | 5,8,11,14-Eicosatetraynoic acid, TMS derivative | 0.93 | 645 | C23H32O2Si | 368 | NA | mainlib |
| 20.10 | à-D-Glucopyranoside, methyl 2-(acetylamino)-2-deoxy-3-O-(trimethylsilyl)-, cyclic methylboronate | 0.93 | 650 | C13H26BNO6Si | 331 | 54477-01-9 | mainlib |
| 20.10 | 1,2-BIS(TRIMETHYLSILYOXY)-4-TRIMETHYLSILYLOXYMETHYLBENZENE | 0.93 | 650 | C16H32O3Si3 | 356 | NA | WileyRegistry8e |

Compound Structure | Hit Spectrum

á-D-GLUCOPYRANOSIDE, METHYL 2,3-BIS-O-(TRIMETHYLSILYL)-, CYCLIC METHYLBORONATE
Formula C14H31BO6Si2, MW 362, CAS# 56211-07-5, Entry# 235857

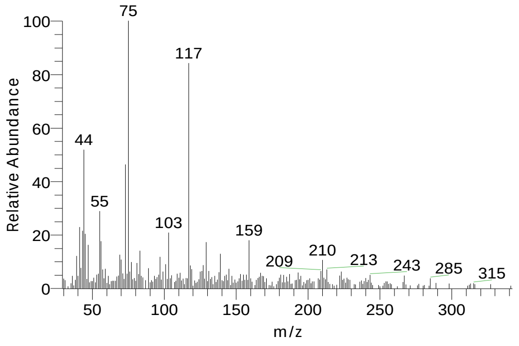

# *My GC-MS Report*

Compound Structure | Hit Spectrum

Formula C13H26BNO6Si, MW 331, CAS# 54477-01-9, Entry# 215088

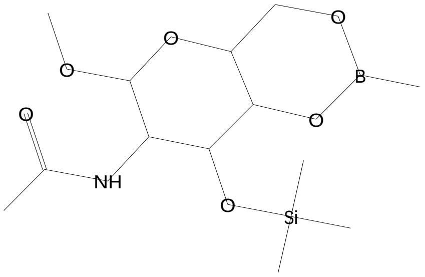


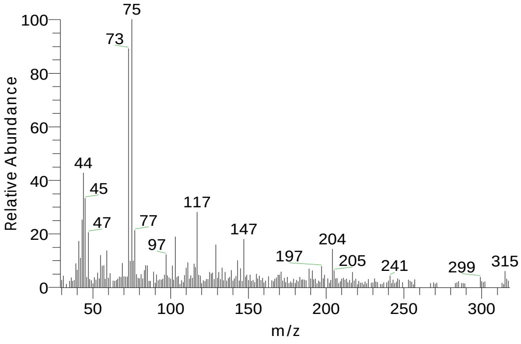


5,8,11,14-Eicosatetraynoic acid, TMS derivative
Formula C23H32O2Si, MW 368, CAS# NA, Entry# 41370
5,8,11,14-Eicosatetraynoic acid, trimethylsilyl ester

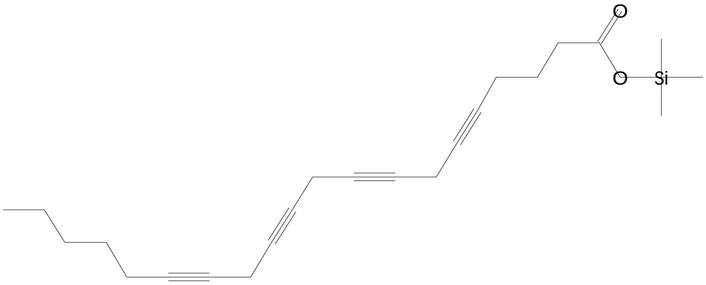


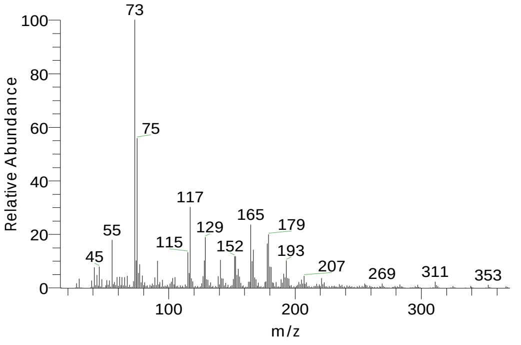


Formula C13H26BNO6Si, MW 331, CAS# 54477-01-9, Entry# 45257

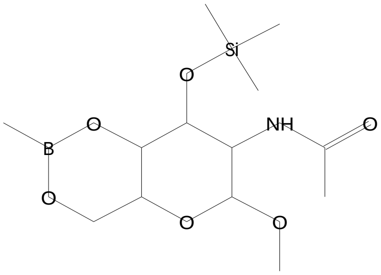


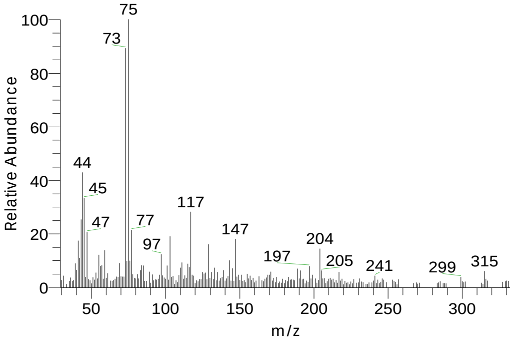


1,2-BIS(TRIMETHYLSILYOXY)-4-TRIMETHYLSILYLOXYMETHYLBENZENE
Formula C16H32O3Si3, MW 356, CAS# NA, Entry# 232178

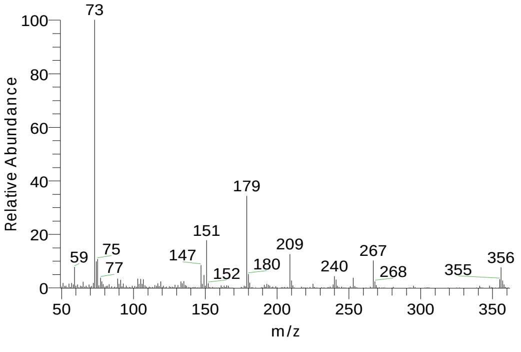

# *My GC-MS Report*

14008 #5177 RT: 21.36 AV: 1 NL: 7.23E6
T: + c EI Full ms [50.000-750.000]

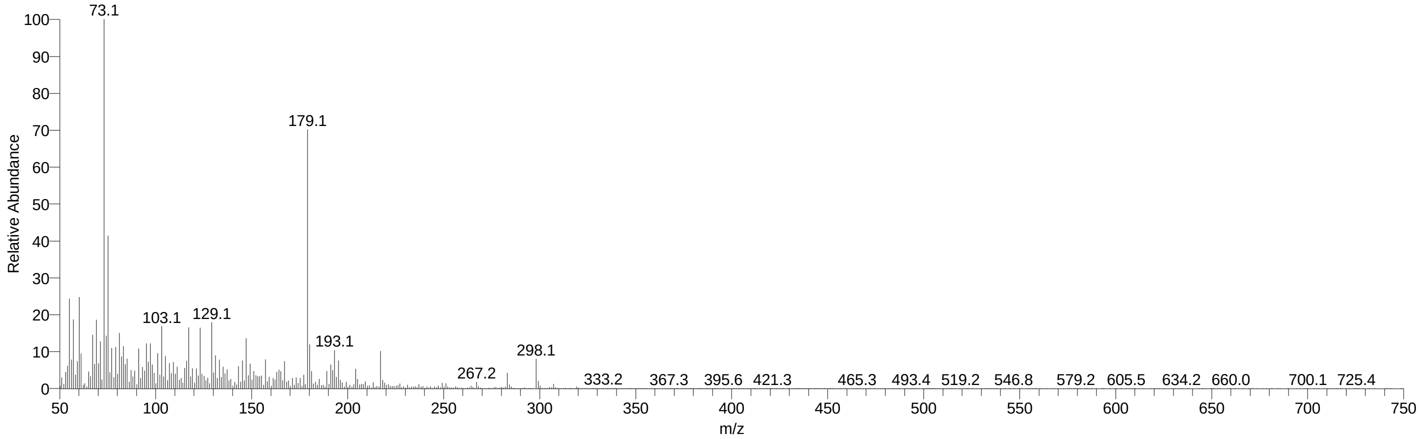


| RT | Compound Name | Area % | MF | Molecular Formula | Molecular Weight | Cas # | Library |
|---|---|---|---|---|---|---|---|
| 21.36 | à-D-GLUCOPYRANOSIDE, METHYL 2-(ACETYLAMINO)-2-DEOXY-3-O-(TRIMETHYLSILYL)-, CYCLIC METHYLBORONATE | 1.68 | 739 | C13H26BNO6Si | 331 | 54477-01-9 | WileyRegistry8e |
| 21.36 | à-D-Glucopyranoside, methyl 2-(acetylamino)-2-deoxy-3-O-(trimethylsilyl)-, cyclic methylboronate | 1.68 | 731 | C13H26BNO6Si | 331 | 54477-01-9 | mainlib |
| 21.36 | DASYCARPIDAN-1-METHANOL, ACETATE (ESTER) | 1.68 | 707 | C20H26N2O2 | 326 | 55724-48-6 | WileyRegistry8e |
| 21.36 | 5,8,11,14-Eicosatetraynoic acid, TMS derivative | 1.68 | 697 | C23H32O2Si | 368 | NA | mainlib |
| 21.36 | Dasycarpidan-1-methanol, acetate (ester) | 1.68 | 713 | C20H26N2O2 | 326 | 55724-48-6 | mainlib |

| Compound Structure | Hit Spectrum |
|---|---|

Formula C13H26BNO6Si, MW 331, CAS# 54477-01-9, Entry# 215088

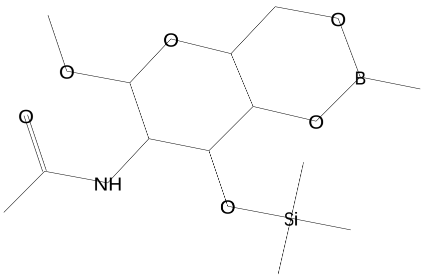


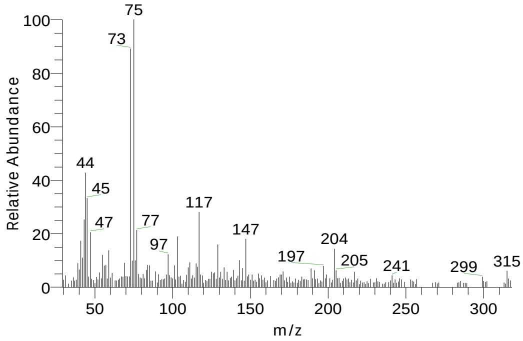


Formula C13H26BNO6Si, MW 331, CAS# 54477-01-9, Entry# 45257

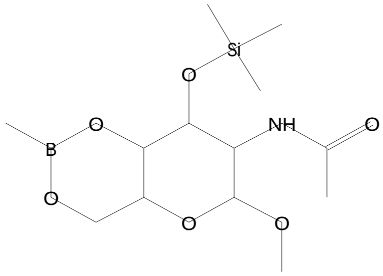


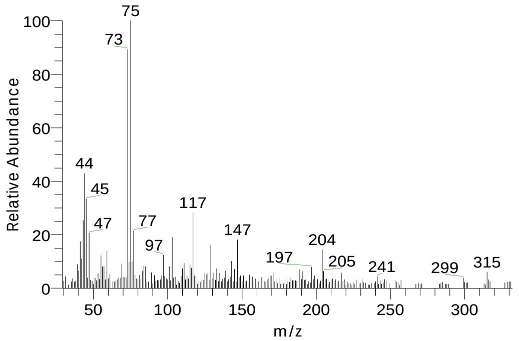

# *My GC-MS Report*

| Compound Structure | Hit Spectrum |
|---|---|

DASYCARPIDAN-1-METHANOL, ACETATE (ESTER)
Formula C20H26N2O2, MW 326, CAS# 55724-48-6, Entry# 211583

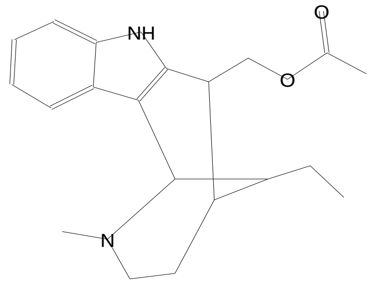


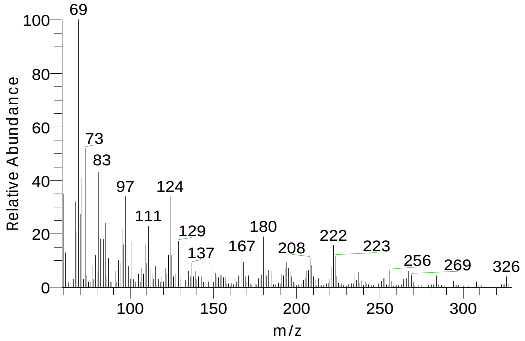


5,8,11,14-Eicosatetraynoic acid, TMS derivative
Formula C23H32O2Si, MW 368, CAS# NA, Entry# 41370
5,8,11,14-Eicosatetraynoic acid, trimethylsilyl ester

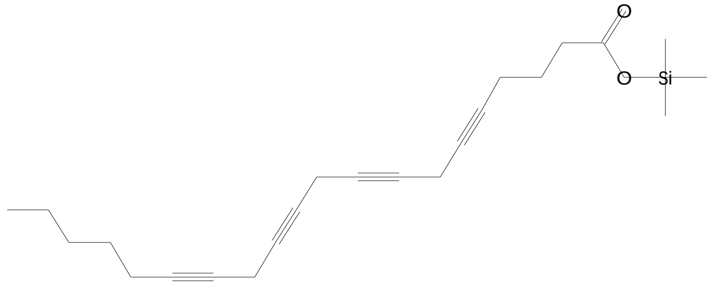


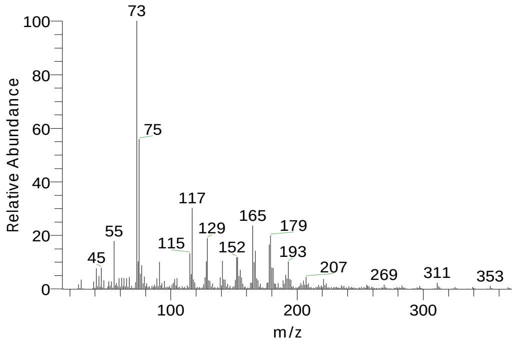


Dasycarpidan-1-methanol, acetate (ester)
Formula C20H26N2O2, MW 326, CAS# 55724-48-6, Entry# 35331
$:28OWHRWZYNUQDQEF-UHFFFAOYSA-N

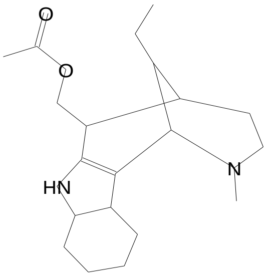


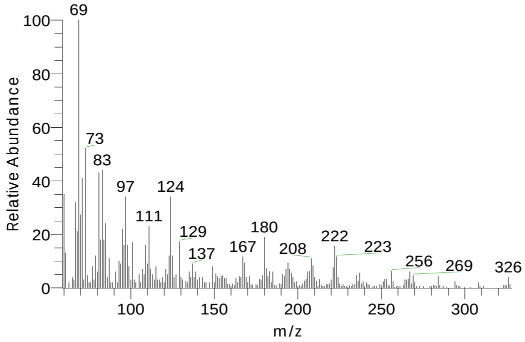


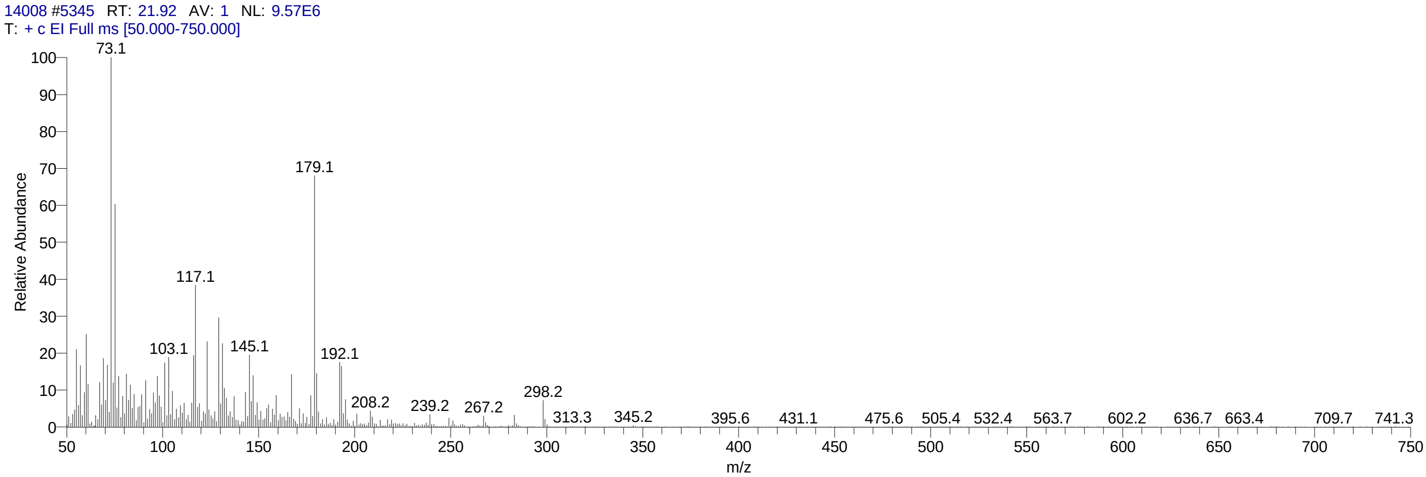


| RT | Compound Name | Area % | MF | Molecular Formula | Molecular Weight | Cas # | Library |
|---|---|---|---|---|---|---|---|
| 21.92 | à-D-GLUCOPYRANOSIDE, METHYL 2-(ACETYLAMINO)-2-DEOXY-3-O-(TRIMETHYLSILYL)-, CYCLIC METHYLBORONATE | 1.34 | 709 | C13H26BNO6Si | 331 | 54477-01-9 | WileyRegistry8e |

# *My GC-MS Report*

| RT | Compound Name | Area % | MF | Molecular Formula | Molecular Weight | Cas # | Library |
|---|---|---|---|---|---|---|---|
| 21.92 | 5,8,11,14-Eicosatetraynoic acid, TMS derivative | 1.34 | 692 | C23H32O2Si | 368 | NA | mainlib |
| 21.92 | à-D-Glucopyranoside, methyl 2-(acetylamino)-2-deoxy-3-O-(trimethylsilyl)-, cyclic methylboronate | 1.34 | 702 | C13H26BNO6Si | 331 | 54477-01-9 | mainlib |
| 21.92 | á-D-GLUCOPYRANOSIDE, METHYL 2,3-BIS-O-(TRIMETHYLSILYL)-, CYCLIC METHYLBORONATE | 1.34 | 704 | C14H31BO6Si2 | 362 | 56211-07-5 | WileyRegistry8e |
| 21.92 | 5,8,11,14-Eicosatetraynoic acid, TBDMS derivative | 1.34 | 675 | C26H38O2Si | 410 | NA | mainlib |

Compound Structure | Hit Spectrum

Formula C13H26BNO6Si, MW 331, CAS# 54477-01-9, Entry# 215088

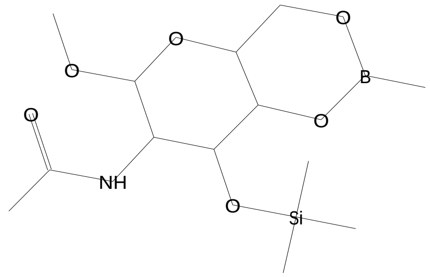


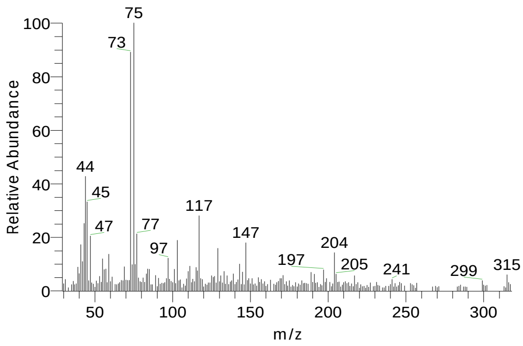


5,8,11,14-Eicosatetraynoic acid, TMS derivative
Formula C23H32O2Si, MW 368, CAS# NA, Entry# 41370
5,8,11,14-Eicosatetraynoic acid, trimethylsilyl ester

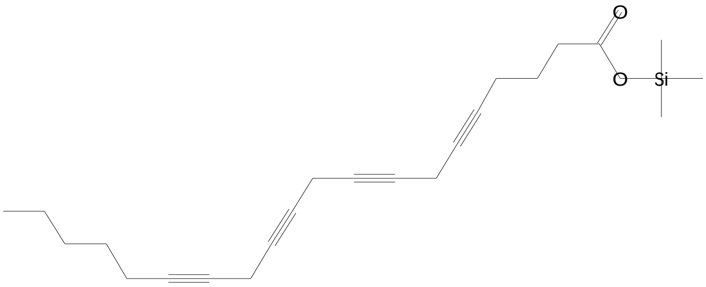


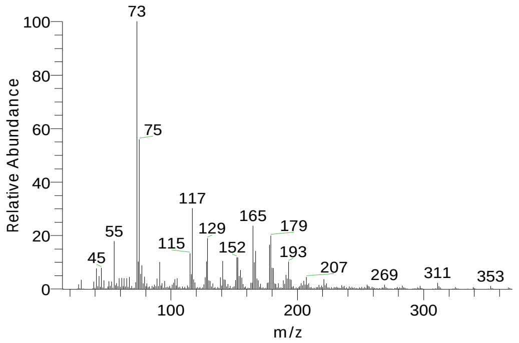


Formula C13H26BNO6Si, MW 331, CAS# 54477-01-9, Entry# 45257

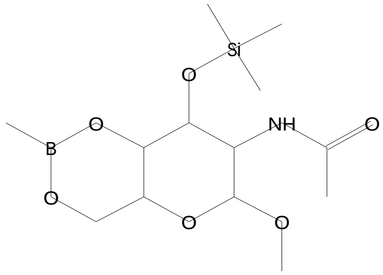


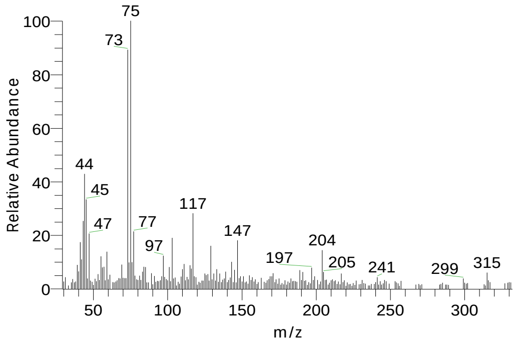

# *My GC-MS Report*

Compound Structure | Hit Spectrum

á-D-GLUCOPYRANOSIDE, METHYL 2,3-BIS-O-(TRIMETHYLSILYL)-, CYCLIC METHYLBORONATE
Formula C14H31BO6Si2, MW 362, CAS# 56211-07-5, Entry# 235857

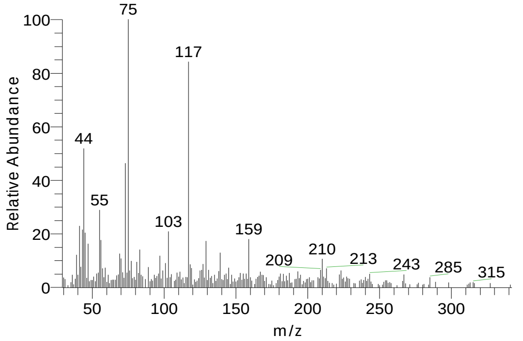


5,8,11,14-Eicosatetraynoic acid, TBDMS derivative
Formula C26H38O2Si, MW 410, CAS# NA, Entry# 45314
5,8,11,14-Eicosatetraynoic acid, tert-butyldimethylsilyl ester

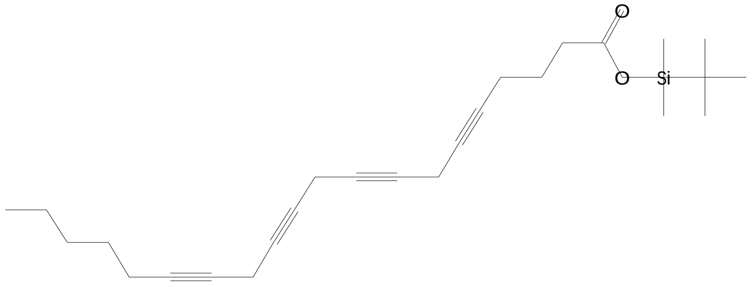


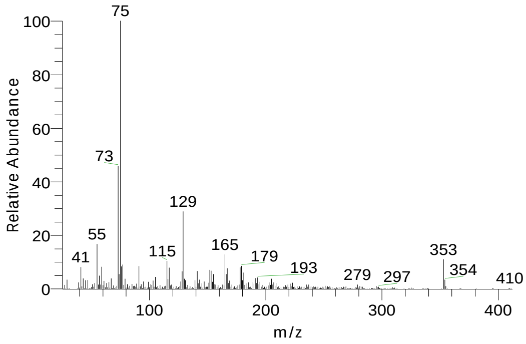


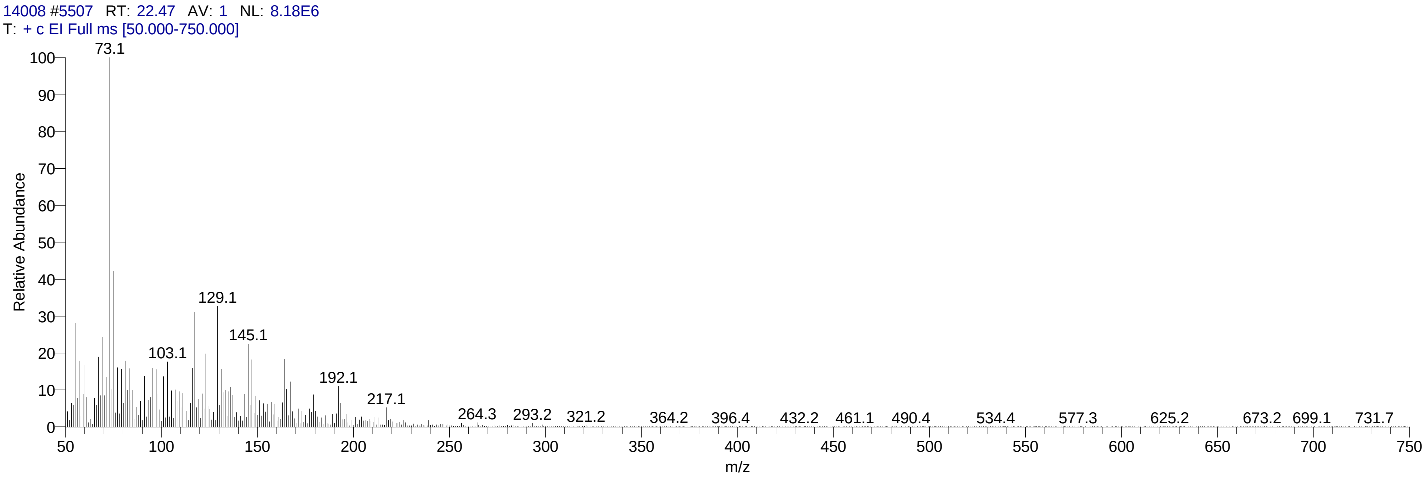


| RT | Compound Name | Area % | MF | Molecular Formula | Molecular Weight | Cas # | Library |
|---|---|---|---|---|---|---|---|
| 22.47 | 5,8,11-Eicosatrienoic acid, (Z)-, TMS derivative | 0.69 | 716 | C23H42O2Si | 378 | NA | mainlib |
| 22.47 | á-D-GLUCOPYRANOSIDE, METHYL 2,3-BIS-O-(TRIMETHYLSILYL)-, CYCLIC METHYLBORONATE | 0.69 | 722 | C14H31BO6Si2 | 362 | 56211-07-5 | WileyRegistry8e |
| 22.47 | Traumatic acid, (E)-, 2TMS derivative | 0.69 | 710 | C18H36O4Si2 | 372 | NA | mainlib |
| 22.47 | Octadecanoic acid, 9,10-epoxy-18-(trimethylsiloxy)-, methyl ester, cis- | 0.69 | 743 | C22H44O4Si | 400 | 22032-78-6 | mainlib |
| 22.47 | á-D-Galactopyranoside, methyl 2,6-bis-O-(trimethylsilyl)-, cyclic methylboronate | 0.69 | 706 | C14H31BO6Si2 | 362 | 56211-06-4 | mainlib |

# My GC-MS Report

Compound Structure | Hit Spectrum

5,8,11-Eicosatrienoic acid, (Z)-, TMS derivative
Formula C23H42O2Si, MW 378, CAS# NA, Entry# 41326
cis-5,8,11-Eicosatrienoic acid, trimethylsilyl ester

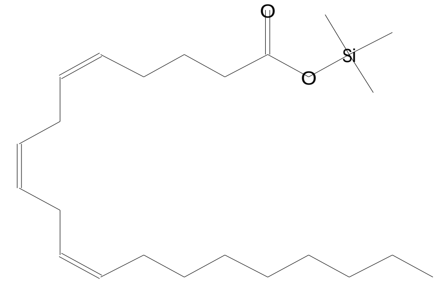


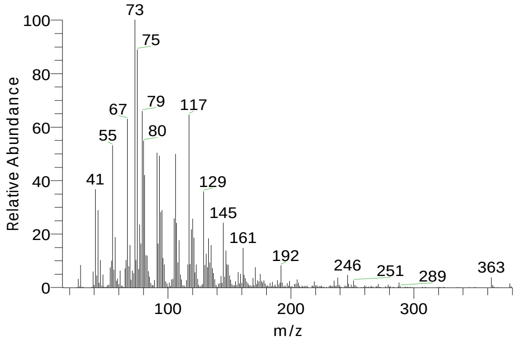


á-D-GLUCOPYRANOSIDE, METHYL 2,3-BIS-O-(TRIMETHYLSILYL)-, CYCLIC METHYLBORONATE
Formula C14H31BO6Si2, MW 362, CAS# 56211-07-5, Entry# 235857

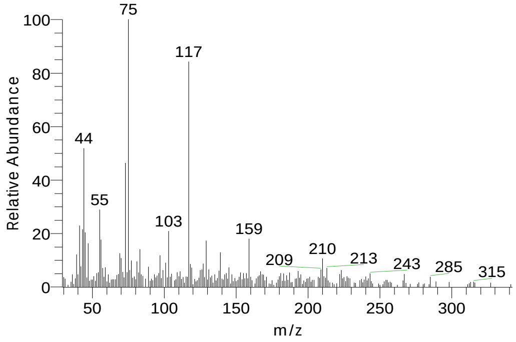


Traumatic acid, (E)-, 2TMS derivative
Formula C18H36O4Si2, MW 372, CAS# NA, Entry# 41329
trans-Traumatic acid, bis(trimethylsilyl) ester

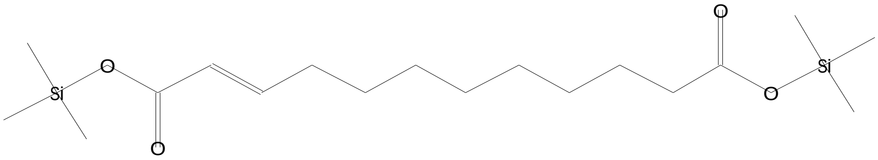


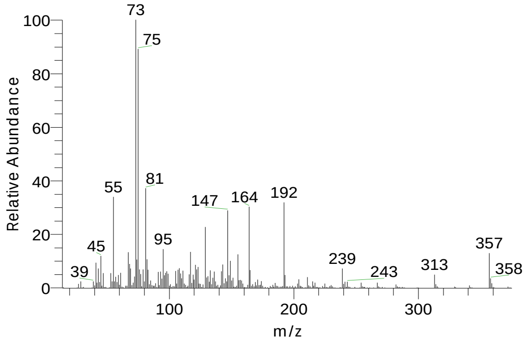


Octadecanoic acid, 9,10-epoxy-18-(trimethylsiloxy)-, methyl ester, cis-
Formula C22H44O4Si, MW 400, CAS# 22032-78-6, Entry# 45171
Methyl 8-(3-(8-[(trimethylsilyl)oxy]octyl)-2-oxiranyl)octanoate #

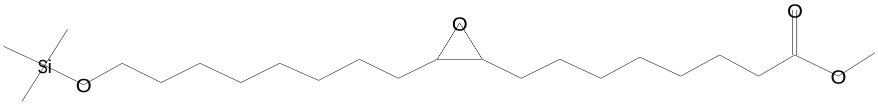


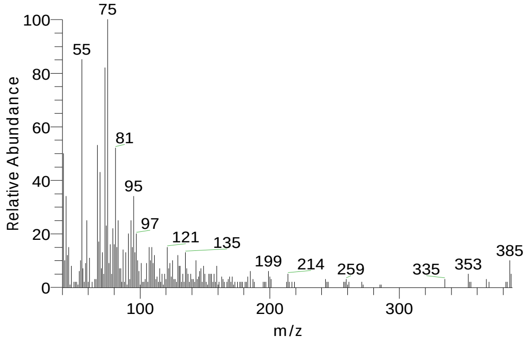

# *My GC-MS Report*

Compound Structure | Hit Spectrum

á-D-Galactopyranoside, methyl 2,6-bis-O-(trimethylsilyl)-, cyclic methylboronate
Formula C14H31BO6Si2, MW 362, CAS# 56211-06-4, Entry# 41379

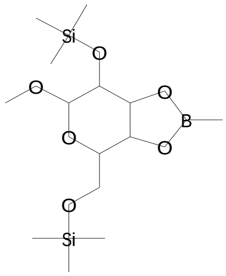


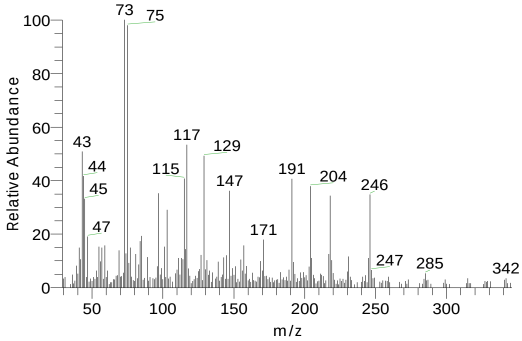


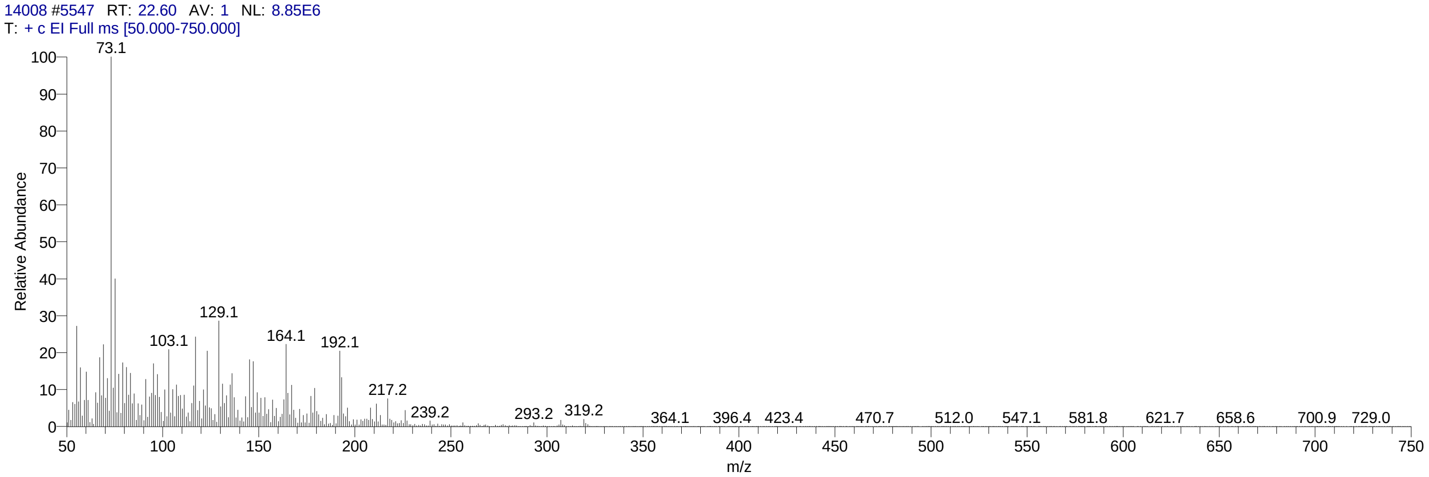


| RT | Compound Name | Area % | MF | Molecular Formula | Molecular Weight | Cas # | Library |
|---|---|---|---|---|---|---|---|
| 22.60 | 5,8,11-Eicosatrienoic acid, (Z)-, TMS derivative | 1.25 | 697 | C23H42O2Si | 378 | NA | mainlib |
| 22.60 | á-D-GLUCOPYRANOSIDE, METHYL 2,3-BIS-O-(TRIMETHYLSILYL)-, CYCLIC METHYLBORONATE | 1.25 | 707 | C14H31BO6Si2 | 362 | 56211-07-5 | WileyRegistry8e |
| 22.60 | Traumatic acid, (E)-, 2TMS derivative | 1.25 | 702 | C18H36O4Si2 | 372 | NA | mainlib |
| 22.60 | Panaxydol, TMS | 1.25 | 694 | C20H32O2Si | 332 | NA | mainlib |
| 22.60 | à-D-GLUCOPYRANOSIDE, METHYL 2,3-BIS-O-(TRIMETHYLSILYL)-, CYCLIC METHYLBORONATE | 1.25 | 710 | C14H31BO6Si2 | 362 | 54400-90-7 | WileyRegistry8e |

Compound Structure | Hit Spectrum

5,8,11-Eicosatrienoic acid, (Z)-, TMS derivative
Formula C23H42O2Si, MW 378, CAS# NA, Entry# 41326
cis-5,8,11-Eicosatrienoic acid, trimethylsilyl ester

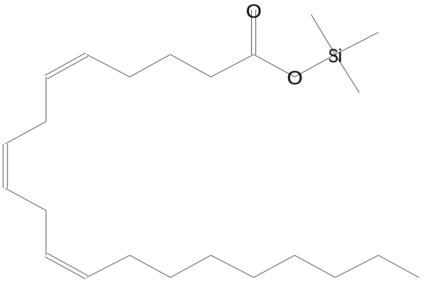


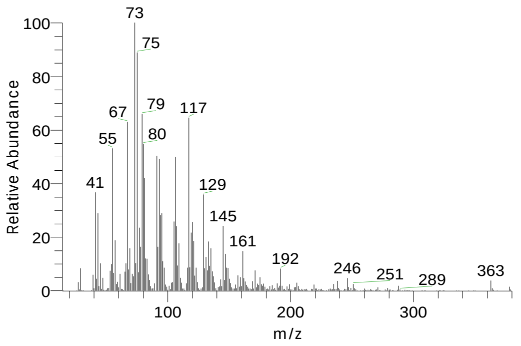

# My GC-MS Report

Compound Structure | Hit Spectrum

á-D-GLUCOPYRANOSIDE, METHYL 2,3-BIS-O-(TRIMETHYLSILYL)-, CYCLIC METHYLBORONATE
Formula C14H31BO6Si2, MW 362, CAS# 56211-07-5, Entry# 235857

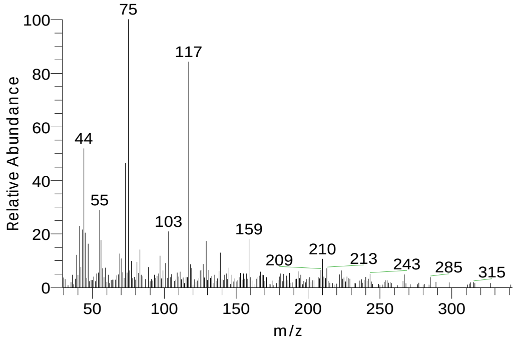


Traumatic acid, (E)-, 2TMS derivative
Formula C18H36O4Si2, MW 372, CAS# NA, Entry# 41329
trans-Traumatic acid, bis(trimethylsilyl) ester

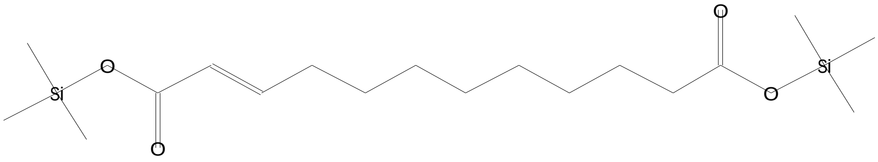


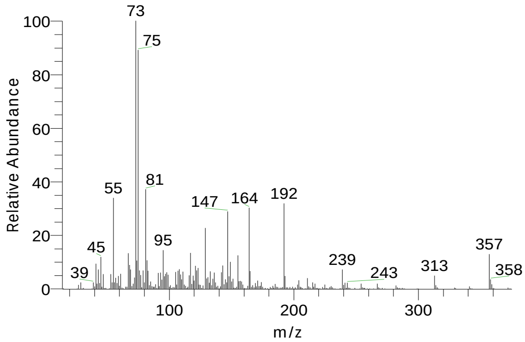


Panaxydol, TMS
Formula C20H32O2Si, MW 332, CAS# NA, Entry# 43136
(((R)-8-((2R,3S)-3-Heptyloxiran-2-yl)octa-1-en-4,6-diyn-3-yl)oxy)trimethylsilane

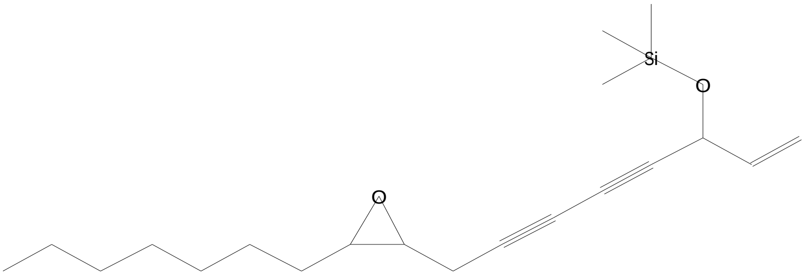


SI 682, RSI 694, mainlib, Entry# 43136, CAS# NA, Panaxydol, TMS

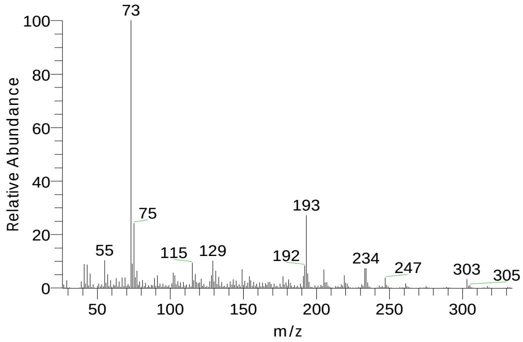


à-D-GLUCOPYRANOSIDE, METHYL 2,3-BIS-O-(TRIMETHYLSILYL)-, CYCLIC METHYLBORONATE
Formula C14H31BO6Si2, MW 362, CAS# 54400-90-7, Entry# 235856

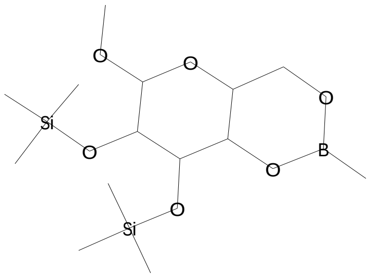


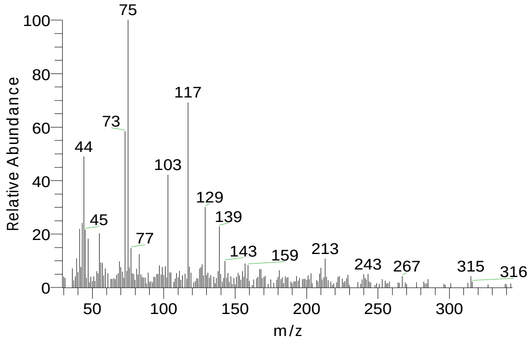

# *My GC-MS Report*

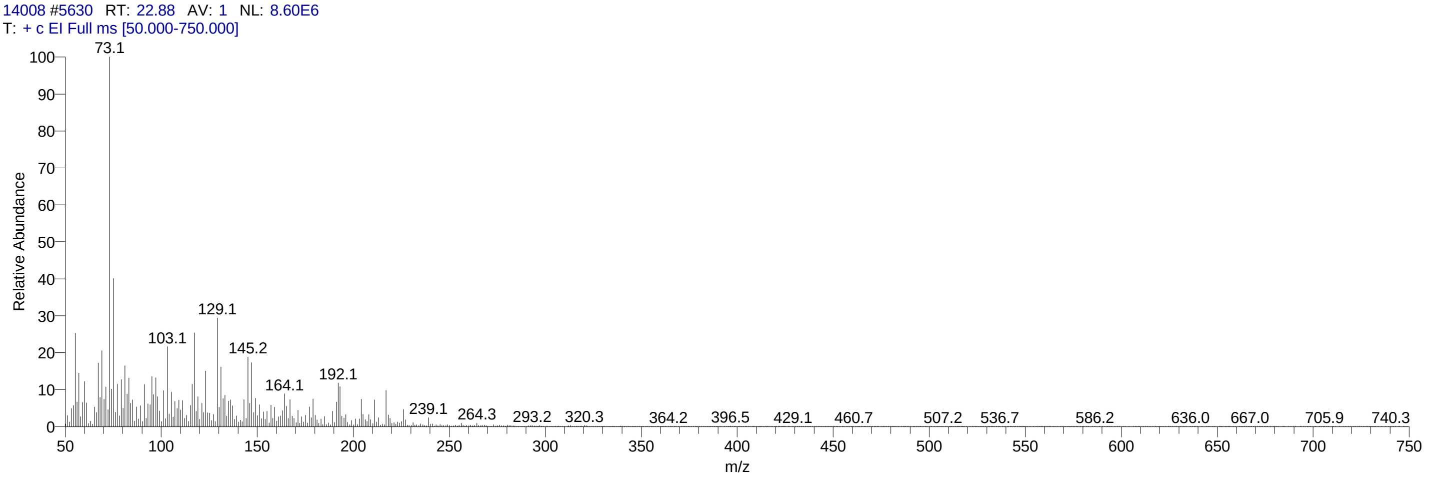


| RT | Compound Name | Area % | MF | Molecular Formula | Molecular Weight | Cas # | Library |
|---|---|---|---|---|---|---|---|
| 22.88 | á-D-Galactopyranoside, methyl 2,6-bis-O-(trimethylsilyl)-, cyclic methylboronate | 0.36 | 727 | C14H31BO6Si2 | 362 | 56211-06-4 | mainlib |
| 22.88 | á-D-GALACTOPYRANOSIDE, METHYL 2,6-BIS-O-(TRIMETHYLSILYL)-, CYCLIC METHYLBORONATE | 0.36 | 727 | C14H31BO6Si2 | 362 | 56211-06-4 | WileyRegistry8e |
| 22.88 | á-D-GLUCOPYRANOSIDE, METHYL 2,3-BIS-O-(TRIMETHYLSILYL)-, CYCLIC METHYLBORONATE | 0.36 | 730 | C14H31BO6Si2 | 362 | 56211-07-5 | WileyRegistry8e |
| 22.88 | 5,8,11-Eicosatrienoic acid, (Z)-, TMS derivative | 0.36 | 711 | C23H42O2Si | 378 | NA | mainlib |
| 22.88 | 2-Oleoylglycerol, 2TMS derivative | 0.36 | 731 | C27H56O4Si2 | 500 | 56554-42-8 | mainlib |

Compound Structure | Hit Spectrum

á-D-Galactopyranoside, methyl 2,6-bis-O-(trimethylsilyl)-, cyclic methylboronate
Formula C14H31BO6Si2, MW 362, CAS# 56211-06-4, Entry# 41379

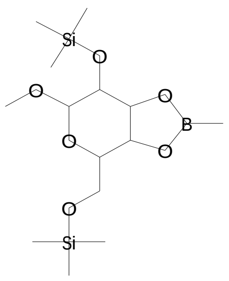


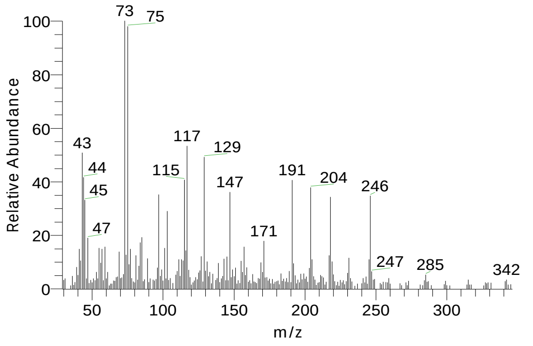


á-D-GALACTOPYRANOSIDE, METHYL 2,6-BIS-O-(TRIMETHYLSILYL)-, CYCLIC METHYLBORONATE
Formula C14H31BO6Si2, MW 362, CAS# 56211-06-4, Entry# 235850

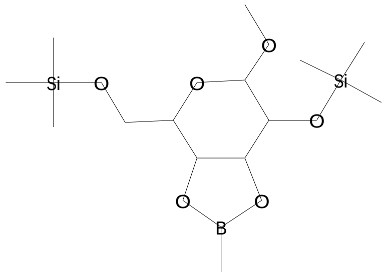


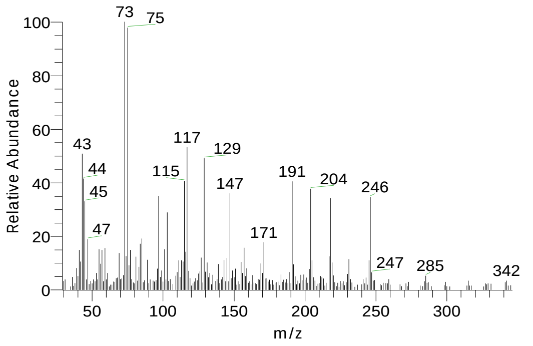

# My GC-MS Report

Compound Structure | Hit Spectrum

á-D-GLUCOPYRANOSIDE, METHYL 2,3-BIS-O-(TRIMETHYLSILYL)-, CYCLIC METHYLBORONATE
Formula C14H31BO6Si2, MW 362, CAS# 56211-07-5, Entry# 235857

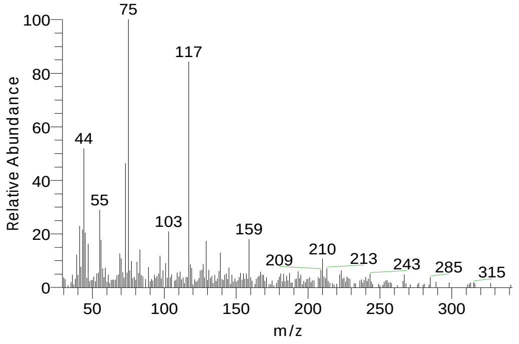


5,8,11-Eicosatrienoic acid, (Z)-, TMS derivative
Formula C23H42O2Si, MW 378, CAS# NA, Entry# 41326
cis-5,8,11-Eicosatrienoic acid, trimethylsilyl ester

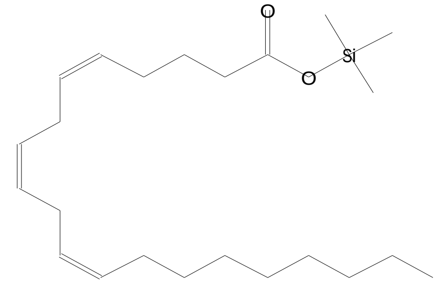


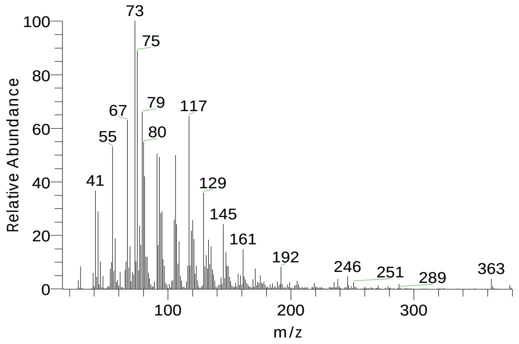


2-Oleoylglycerol, 2TMS derivative
Formula C27H56O4Si2, MW 500, CAS# 56554-42-8, Entry# 41917
9-Octadecenoic acid, 2-[(trimethylsilyl)oxy]-1-[[(trimethylsilyl)oxy]methyl]ethyl ester

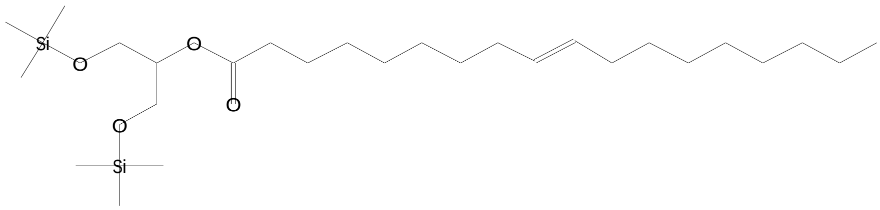


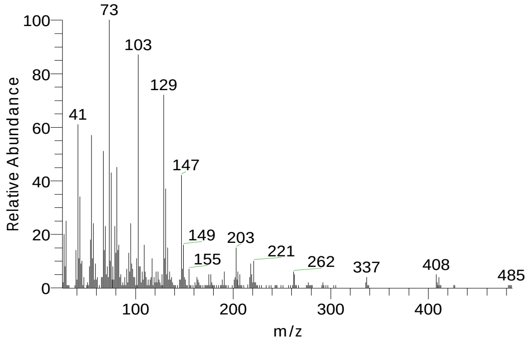


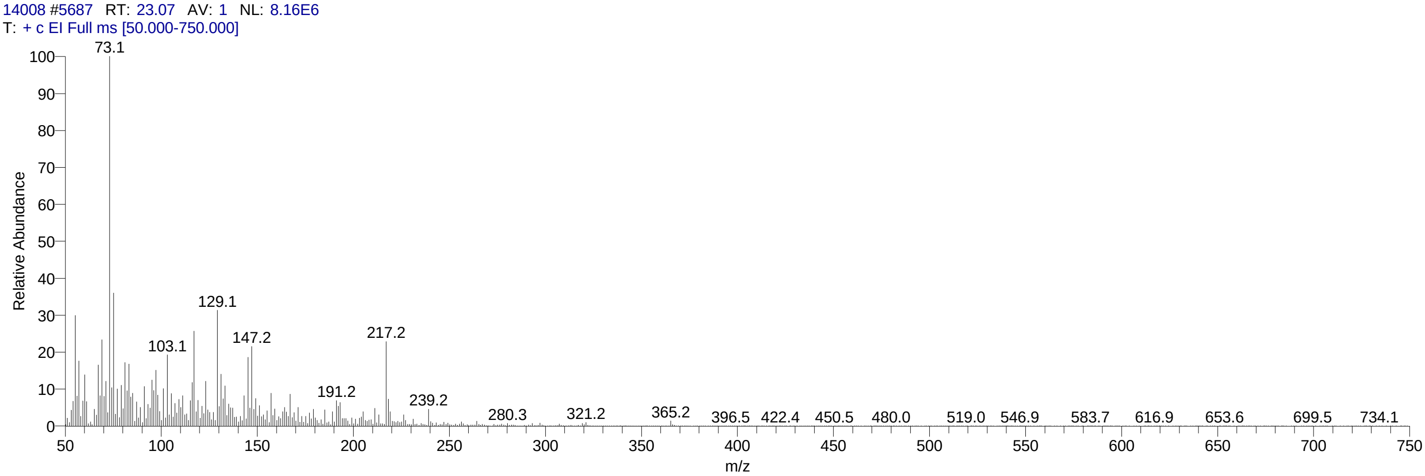


| RT | Compound Name | Area % | MF | Molecular Formula | Molecular Weight | Cas # | Library |
|---|---|---|---|---|---|---|---|
| 23.07 | á-D-Galactopyranoside, methyl 2,6-bis-O-(trimethylsilyl)-, cyclic methylboronate | 0.38 | 734 | C14H31BO6Si2 | 362 | 56211-06-4 | mainlib |

# *My GC-MS Report*

| RT | Compound Name | Area % | MF | Molecular Formula | Molecular Weight | Cas # | Library |
|---|---|---|---|---|---|---|---|
| 23.07 | á-D-GALACTOPYRANOSIDE, METHYL 2,6-BIS-O-(TRIMETHYLSILYL)-, CYCLIC METHYLBORONATE | 0.38 | 734 | C14H31BO6Si2 | 362 | 56211-06-4 | WileyRegistry8e |
| 23.07 | á-D-GLUCOPYRANOSIDE, METHYL 2,3-BIS-O-(TRIMETHYLSILYL)-, CYCLIC METHYLBORONATE | 0.38 | 739 | C14H31BO6Si2 | 362 | 56211-07-5 | WileyRegistry8e |
| 23.07 | D-GLUCOSE, 6-O-à-D-GALACTOPYRANOSYL-, BIS-O-(TRIMETHYLSILYL) DERIV., CYCLIC TRIS(METHYLBORONATE) | 0.38 | 719 | C21H41B3O11Si2 | 558 | 72347-76-3 | WileyRegistry8e |
| 23.07 | 5,8,11-Eicosatrienoic acid, (Z)-, TMS derivative | 0.38 | 704 | C23H42O2Si | 378 | NA | mainlib |

Compound Structure | Hit Spectrum

á-D-Galactopyranoside, methyl 2,6-bis-O-(trimethylsilyl)-, cyclic methylboronate
Formula C14H31BO6Si2, MW 362, CAS# 56211-06-4, Entry# 41379

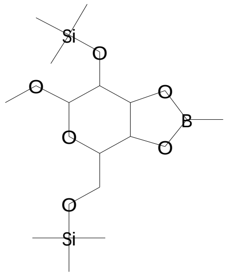


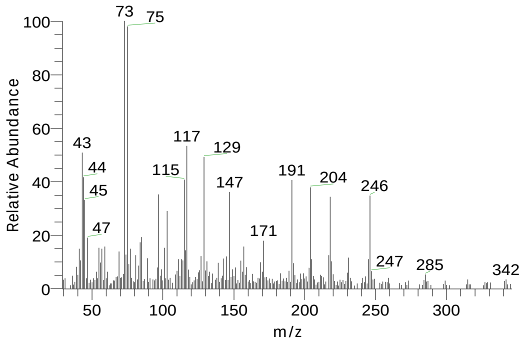


á-D-GALACTOPYRANOSIDE, METHYL 2,6-BIS-O-(TRIMETHYLSILYL)-, CYCLIC METHYLBORONATE
Formula C14H31BO6Si2, MW 362, CAS# 56211-06-4, Entry# 235850

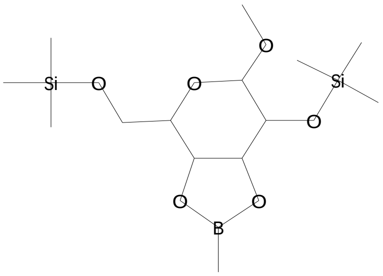


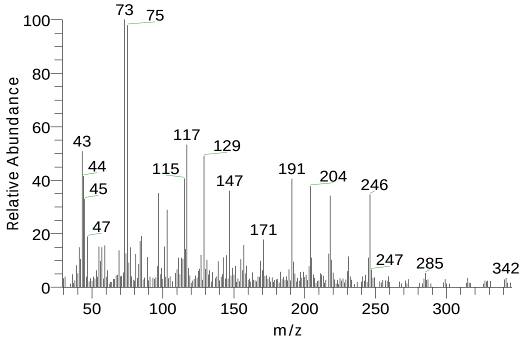


á-D-GLUCOPYRANOSIDE, METHYL 2,3-BIS-O-(TRIMETHYLSILYL)-, CYCLIC METHYLBORONATE
Formula C14H31BO6Si2, MW 362, CAS# 56211-07-5, Entry# 235857

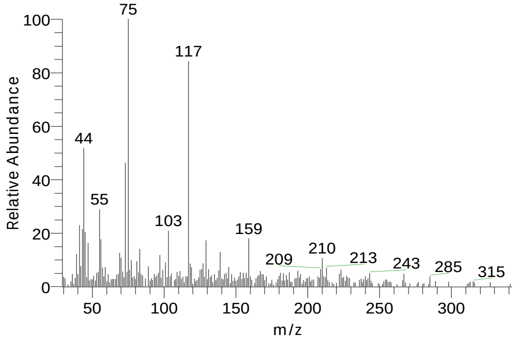

# My GC-MS Report

Compound Structure | Hit Spectrum

Formula C21H41B3O11Si2, MW 558, CAS# 72347-76-3, Entry# 293114

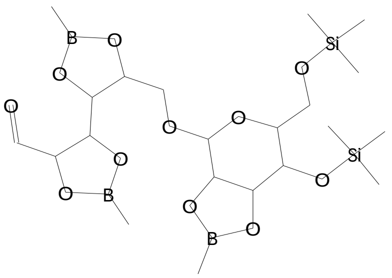

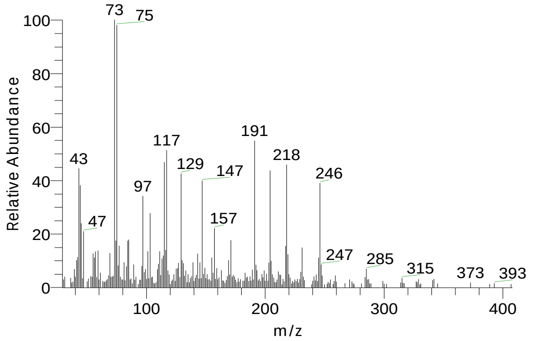


5,8,11-Eicosatrienoic acid, (Z)-, TMS derivative
Formula C23H42O2Si, MW 378, CAS# NA, Entry# 41326
cis-5,8,11-Eicosatrienoic acid, trimethylsilyl ester

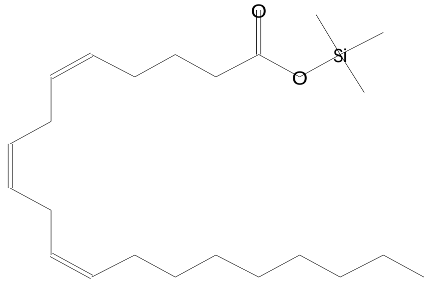

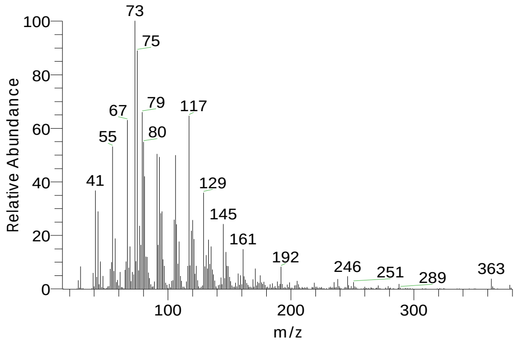


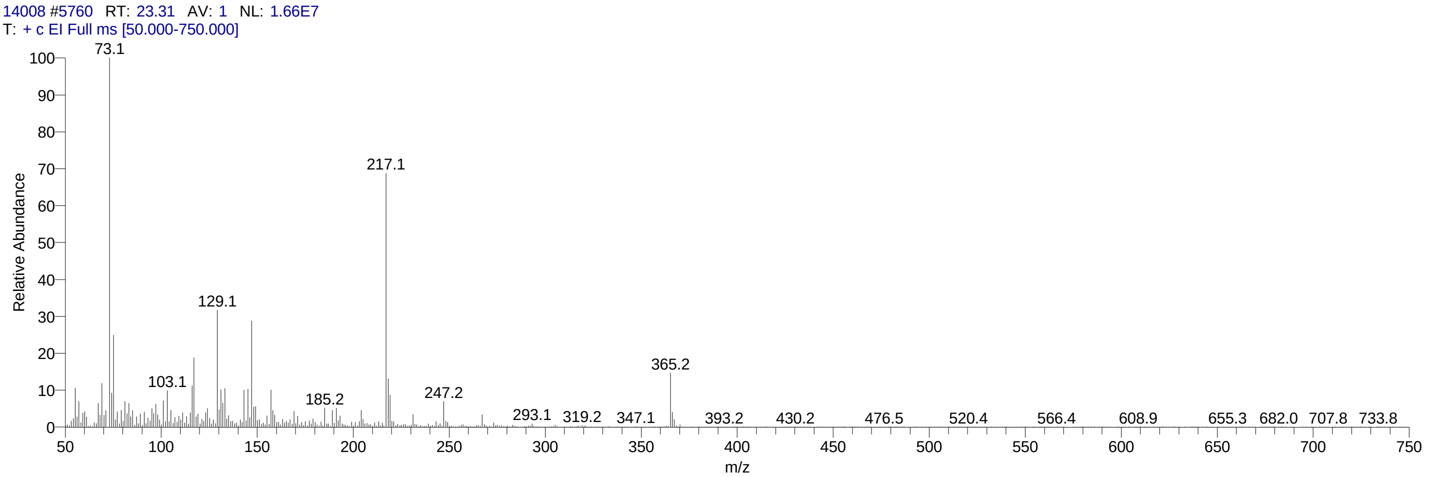


| RT | Compound Name | Area % | MF | Molecular Formula | Molecular Weight | Cas # | Library |
|---|---|---|---|---|---|---|---|
| 23.31 | MANNOFURANOSIDE, METHYL 2,3,5,6-TETRAKIS-O-(TRIMETHYLSILYL)-, à-D- | 2.56 | 772 | C19H46O6Si4 | 482 | 6737-01-5 | WileyRegistry8e |
| 23.31 | Uridine, 3TMS derivative | 2.56 | 717 | C18H36N2O6Si3 | 460 | 10457-16-6 | mainlib |
| 23.31 | à-D-Galactopyranose, 1,2,3-tris-O-(trimethylsilyl)-, cyclic methylboronate | 2.56 | 723 | C16H37BO6Si3 | 420 | 56196-95-3 | mainlib |
| 23.31 | à-D-GALACTOPYRANOSE, 1,2,3-TRIS-O-(TRIMETHYLSILYL)-, CYCLIC METHYLBORONATE | 2.56 | 723 | C16H37BO6Si3 | 420 | 56196-95-3 | WileyRegistry8e |
| 23.31 | à-D-Glucopyranosiduronic acid, 3-(5-ethylhexahydro-2,4,6-trioxo-5-pyrimidinyl)-1,1-dimethylpropyl 2,3,4-tris-O-(trimethylsilyl)-, methyl ester | 2.56 | 704 | C27H52N2O10Si3 | 648 | 55556-81-5 | mainlib |

# My GC-MS Report

Compound Structure | Hit Spectrum

MANNOFURANOSIDE, METHYL 2,3,5,6-TETRAKIS-O-(TRIMETHYLSILYL)-, à-D-
Formula C19H46O6Si4, MW 482, CAS# 6737-01-5, Entry# 282094
METHYL 2,3,5,6-TETRAKIS-O-(TRIMETHYLSILYL)HEXOFURANOSIDE #

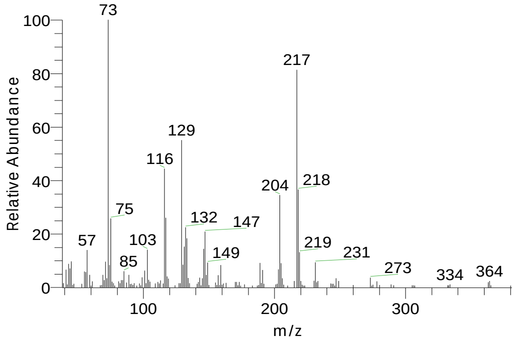


Uridine, 3TMS derivative
Formula C18H36N2O6Si3, MW 460, CAS# 10457-16-6, Entry# 43369
Uridine, 2',3',5'-tris-O-(trimethylsilyl)-

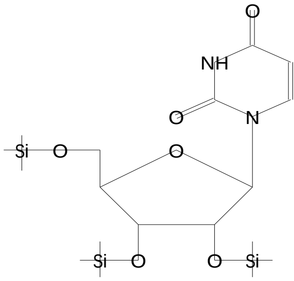


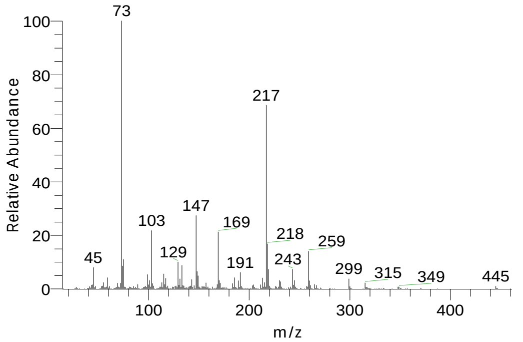


à-D-Galactopyranose, 1,2,3-tris-O-(trimethylsilyl)-, cyclic methylboronate
Formula C16H37BO6Si3, MW 420, CAS# 56196-95-3, Entry# 41501

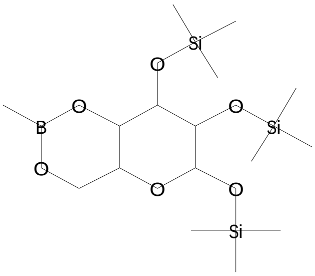


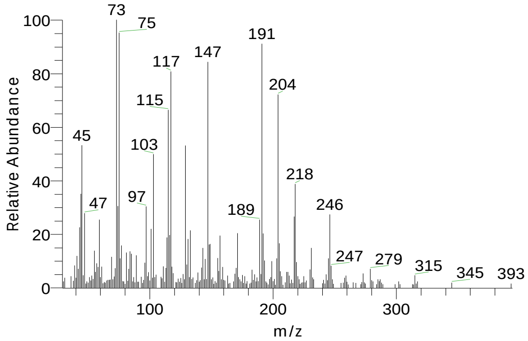


à-D-GALACTOPYRANOSE, 1,2,3-TRIS-O-(TRIMETHYLSILYL)-, CYCLIC METHYLBORONATE
Formula C16H37BO6Si3, MW 420, CAS# 56196-95-3, Entry# 264348

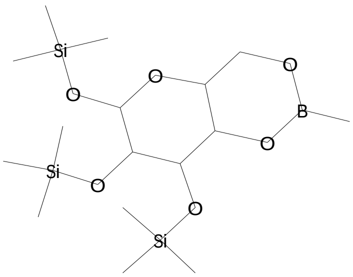


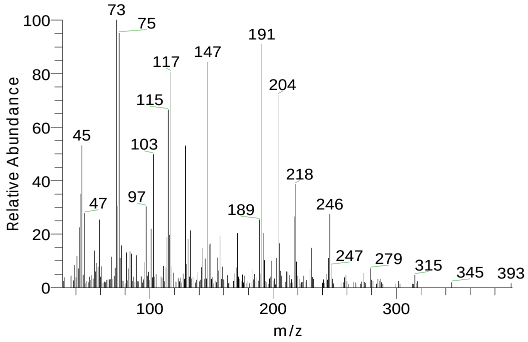

# My GC-MS Report

Compound Structure | Hit Spectrum

Formula C27H52N2O10Si3, MW 648, CAS# 55556-81-5, Entry# 43396

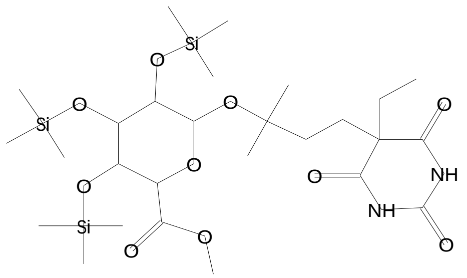


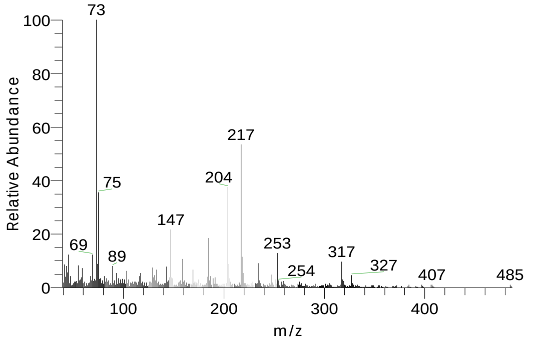


14008 #5862 RT: 23.66 AV: 1 NL: 9.16E6
T: + c EI Full ms [50.000-750.000]

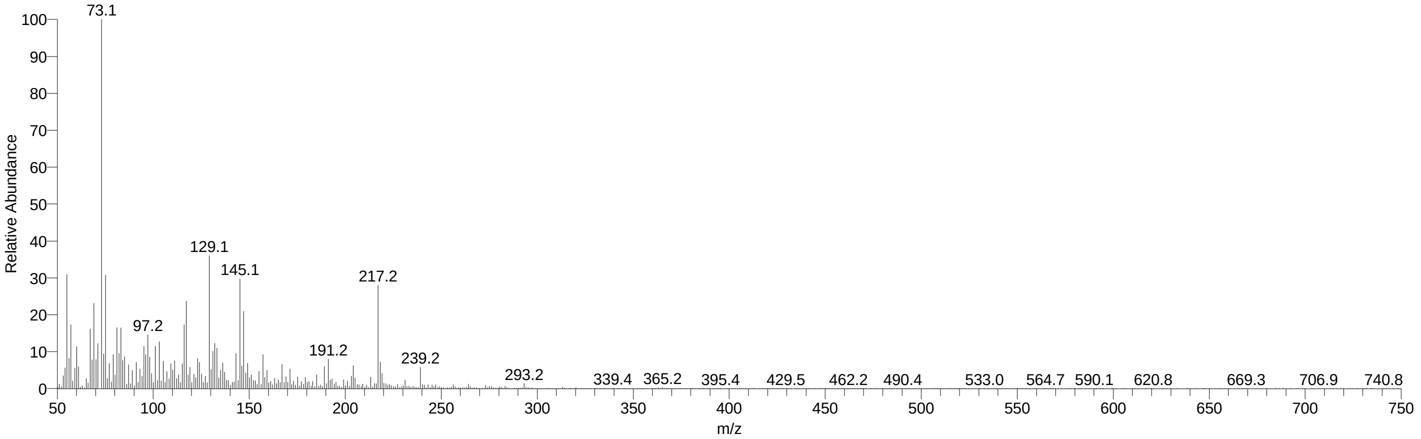


| RT | Compound Name | Area % | MF | Molecular Formula | Molecular Weight | Cas # | Library |
|---|---|---|---|---|---|---|---|
| 23.66 | á-D-Galactopyranoside, methyl 2,6-bis-O-(trimethylsilyl)-, cyclic methylboronate | 0.47 | 754 | C14H31BO6Si2 | 362 | 56211-06-4 | mainlib |
| 23.66 | á-D-GALACTOPYRANOSIDE, METHYL 2,6-BIS-O-(TRIMETHYLSILYL)-, CYCLIC METHYLBORONATE | 0.47 | 754 | C14H31BO6Si2 | 362 | 56211-06-4 | WileyRegistry8e |
| 23.66 | á-D-Galactopyranoside, methyl 2,3-bis-O-(trimethylsilyl)-, cyclic butylboronate | 0.47 | 735 | C17H37BO6Si2 | 404 | 56211-10-0 | mainlib |
| 23.66 | á-D-GALACTOPYRANOSIDE, METHYL 2,3-BIS-O-(TRIMETHYLSILYL)-, CYCLIC BUTYLBORONATE | 0.47 | 735 | C17H37BO6Si2 | 404 | 56211-10-0 | WileyRegistry8e |
| 23.66 | D-GLUCOSE, 6-O-à-D-GALACTOPYRANOSYL-, BIS-O-(TRIMETHYLSILYL) DERIV., CYCLIC TRIS(METHYLBORONATE) | 0.47 | 738 | C21H41B3O11Si2 | 558 | 72347-76-3 | WileyRegistry8e |

# My GC-MS Report

Compound Structure | Hit Spectrum

á-D-Galactopyranoside, methyl 2,6-bis-O-(trimethylsilyl)-, cyclic methylboronate
Formula C14H31BO6Si2, MW 362, CAS# 56211-06-4, Entry# 41379

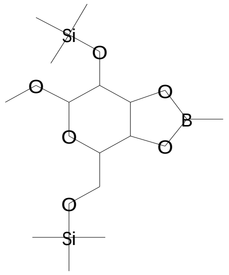

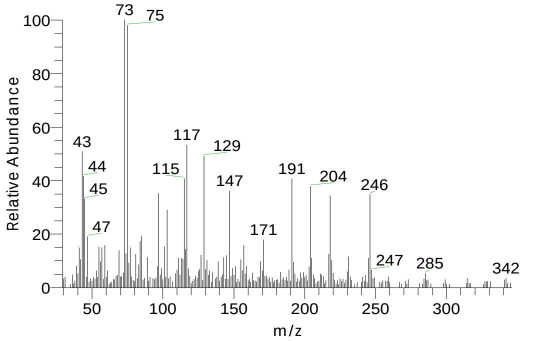


á-D-GALACTOPYRANOSIDE, METHYL 2,6-BIS-O-(TRIMETHYLSILYL)-, CYCLIC METHYLBORONATE
Formula C14H31BO6Si2, MW 362, CAS# 56211-06-4, Entry# 235850

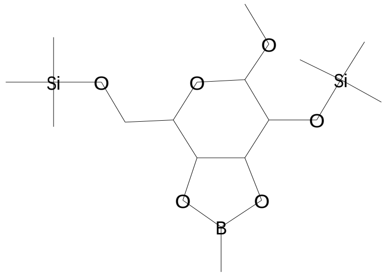

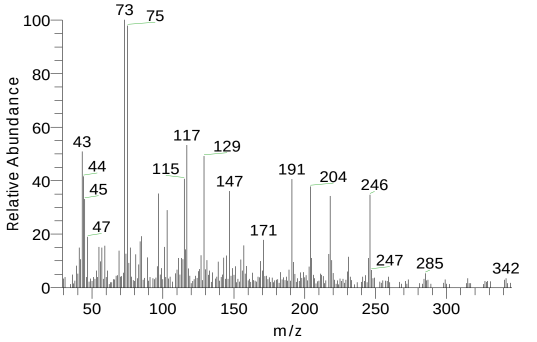


á-D-Galactopyranoside, methyl 2,3-bis-O-(trimethylsilyl)-, cyclic butylboronate
Formula C17H37BO6Si2, MW 404, CAS# 56211-10-0, Entry# 42381

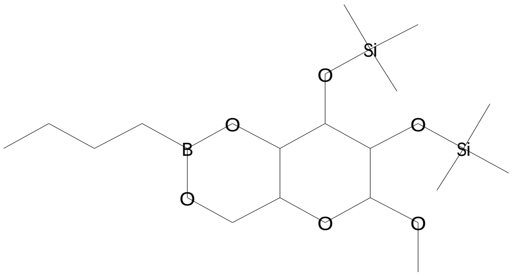

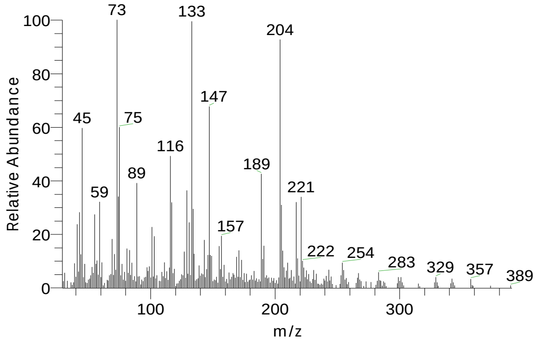


á-D-GALACTOPYRANOSIDE, METHYL 2,3-BIS-O-(TRIMETHYLSILYL)-, CYCLIC BUTYLBORONATE
Formula C17H37BO6Si2, MW 404, CAS# 56211-10-0, Entry# 257666

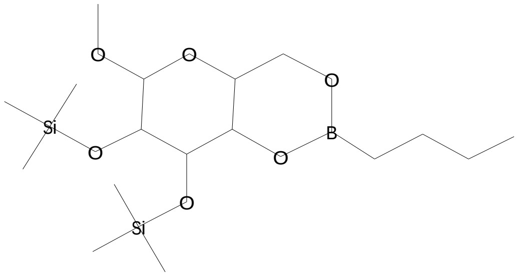

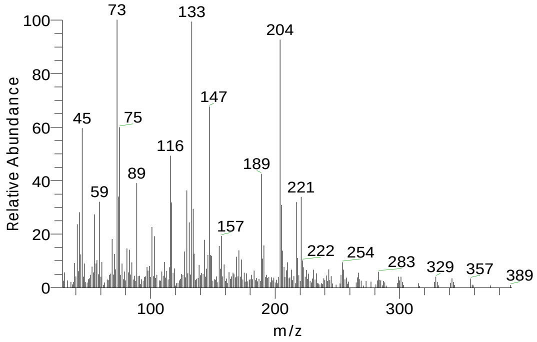

# My GC-MS Report

Compound Structure | Hit Spectrum

Formula C21H41B3O11Si2, MW 558, CAS# 72347-76-3, Entry# 293114

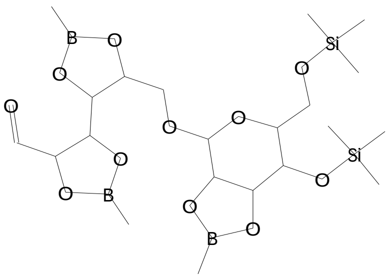


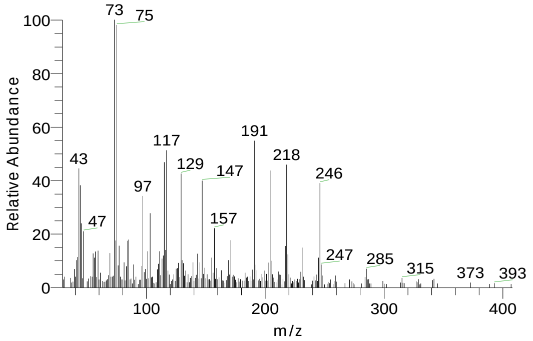


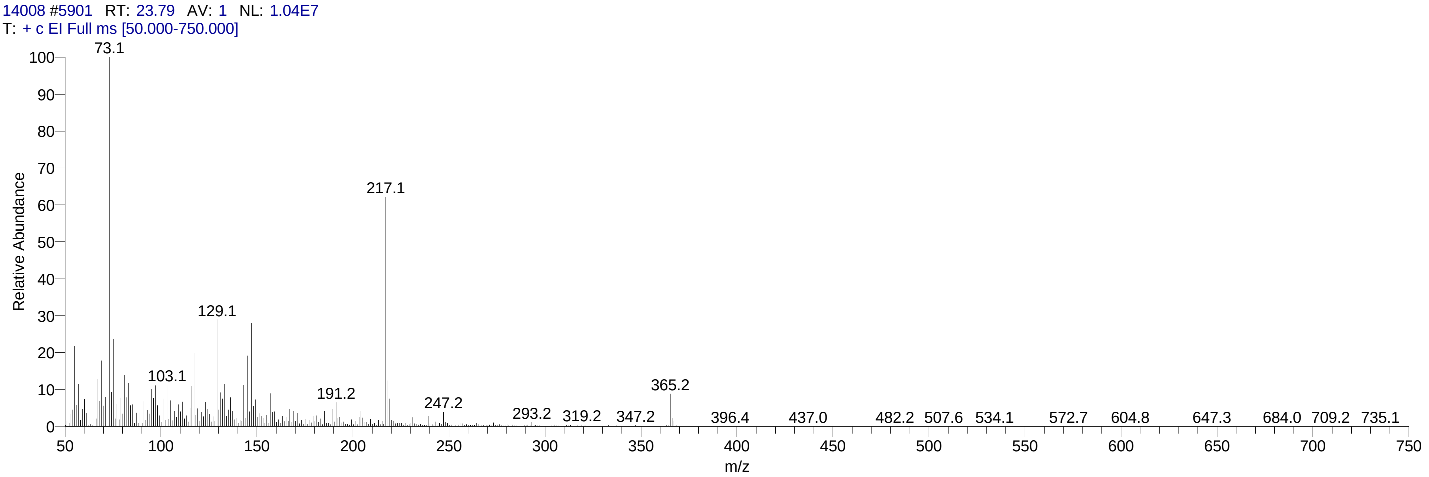


| RT | Compound Name | Area % | MF | Molecular Formula | Molecular Weight | Cas # | Library |
|---|---|---|---|---|---|---|---|
| 23.79 | á-D-Galactopyranoside, methyl 2,6-bis-O-(trimethylsilyl)-, cyclic methylboronate | 0.44 | 734 | C14H31BO6Si2 | 362 | 56211-06-4 | mainlib |
| 23.79 | á-D-GALACTOPYRANOSIDE, METHYL 2,6-BIS-O-(TRIMETHYLSILYL)-, CYCLIC METHYLBORONATE | 0.44 | 734 | C14H31BO6Si2 | 362 | 56211-06-4 | WileyRegistry8e |
| 23.79 | D-GLUCOSE, 6-O-à-D-GALACTOPYRANOSYL-, BIS-O-(TRIMETHYLSILYL) DERIV., CYCLIC TRIS(METHYLBORONATE) | 0.44 | 722 | C21H41B3O11Si2 | 558 | 72347-76-3 | WileyRegistry8e |
| 23.79 | à-D-Glucopyranosiduronic acid, 3-(5-ethylhexahydro-2,4,6-trioxo-5-pyrimidinyl)-1,1-dimethylpropyl 2,3,4-tris-O-(trimethylsilyl)-, methyl ester | 0.44 | 710 | C27H52N2O10Si3 | 648 | 55556-81-5 | mainlib |
| 23.79 | à-D-GALACTOPYRANOSIDE, METHYL 2,3-BIS-O-(TRIMETHYLSILYL)-, CYCLIC BUTYLBORONATE | 0.44 | 715 | C17H37BO6Si2 | 404 | 56211-11-1 | WileyRegistry8e |

# My GC-MS Report

Compound Structure | Hit Spectrum

á-D-Galactopyranoside, methyl 2,6-bis-O-(trimethylsilyl)-, cyclic methylboronate
Formula C14H31BO6Si2, MW 362, CAS# 56211-06-4, Entry# 41379

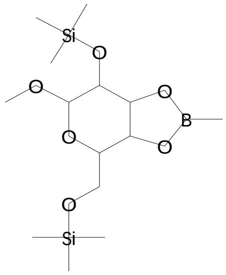

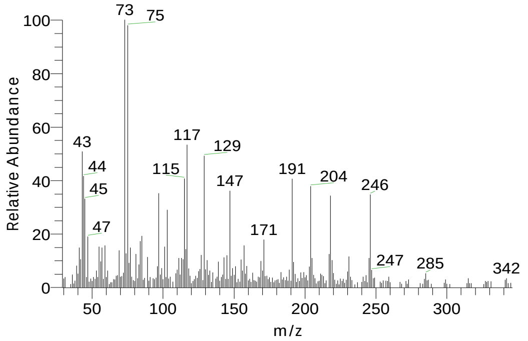


á-D-GALACTOPYRANOSIDE, METHYL 2,6-BIS-O-(TRIMETHYLSILYL)-, CYCLIC METHYLBORONATE
Formula C14H31BO6Si2, MW 362, CAS# 56211-06-4, Entry# 235850

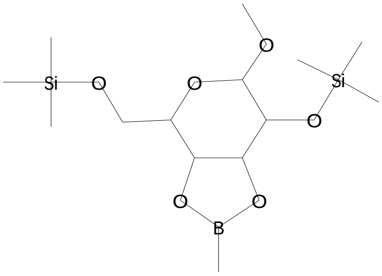

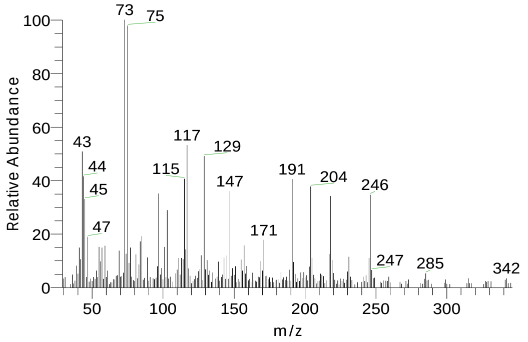


Formula C21H41B3O11Si2, MW 558, CAS# 72347-76-3, Entry# 293114

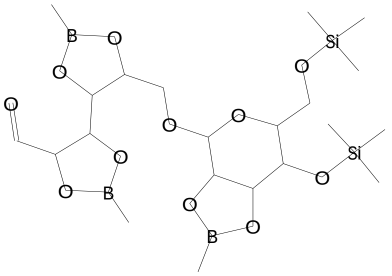

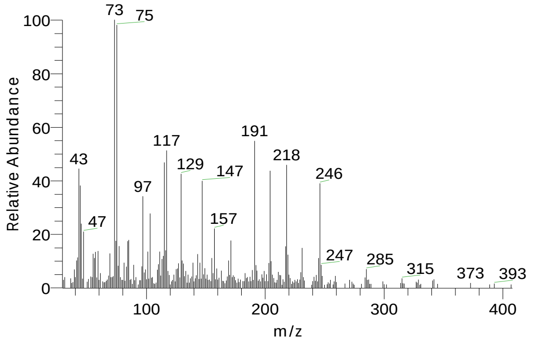


Formula C27H52N2O10Si3, MW 648, CAS# 55556-81-5, Entry# 43396

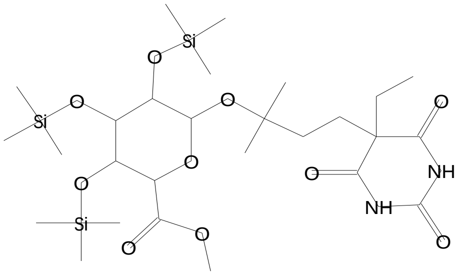

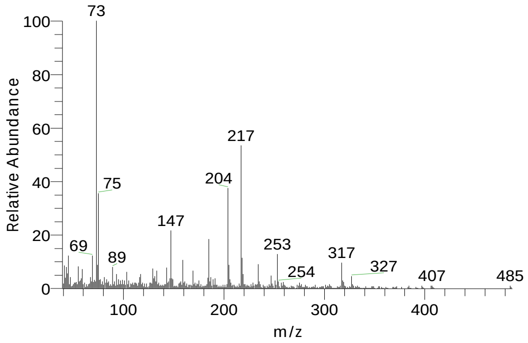

# *My GC-MS Report*

Compound Structure

à-D-GALACTOPYRANOSIDE, METHYL 2,3-BIS-O-(TRIMETHYLSILYL)-, CYCLIC BUTYLBORONATE
Formula C17H37BO6Si2, MW 404, CAS# 56211-11-1, Entry# 257663

Hit Spectrum

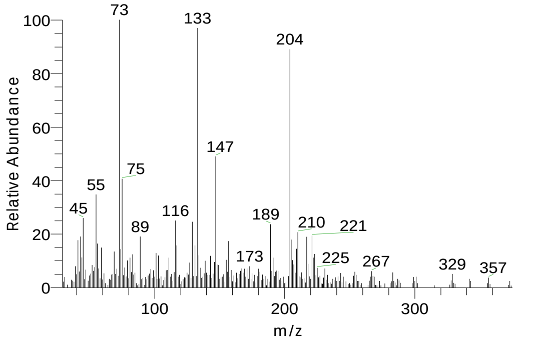


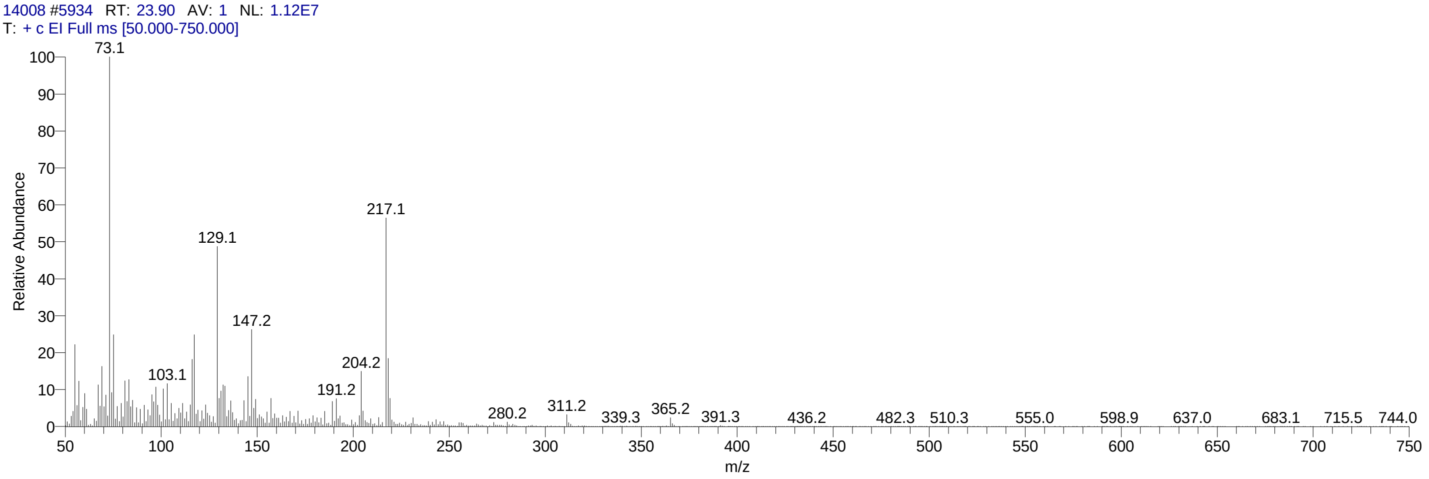


| RT | Compound Name | Area % | MF | Molecular Formula | Molecular Weight | Cas # | Library |
|---|---|---|---|---|---|---|---|
| 23.90 | á-D-Galactopyranoside, methyl 2,3-bis-O-(trimethylsilyl)-, cyclic butylboronate | 1.27 | 751 | C17H37BO6Si2 | 404 | 56211-10-0 | mainlib |
| 23.90 | á-D-GALACTOPYRANOSIDE, METHYL 2,3-BIS-O-(TRIMETHYLSILYL)-, CYCLIC BUTYLBORONATE | 1.27 | 751 | C17H37BO6Si2 | 404 | 56211-10-0 | WileyRegistry8e |
| 23.90 | á-D-Galactopyranoside, methyl 2,6-bis-O-(trimethylsilyl)-, cyclic butylboronate | 1.27 | 746 | C17H37BO6Si2 | 404 | 56211-13-3 | mainlib |
| 23.90 | á-D-GALACTOPYRANOSIDE, METHYL 2,6-BIS-O-(TRIMETHYLSILYL)-, CYCLIC BUTYLBORONATE | 1.27 | 746 | C17H37BO6Si2 | 404 | 56211-13-3 | WileyRegistry8e |
| 23.90 | á-D-Galactopyranoside, methyl 2,6-bis-O-(trimethylsilyl)-, cyclic methylboronate | 1.27 | 748 | C14H31BO6Si2 | 362 | 56211-06-4 | mainlib |

Compound Structure

á-D-Galactopyranoside, methyl 2,3-bis-O-(trimethylsilyl)-, cyclic butylboronate
Formula C17H37BO6Si2, MW 404, CAS# 56211-10-0, Entry# 42381

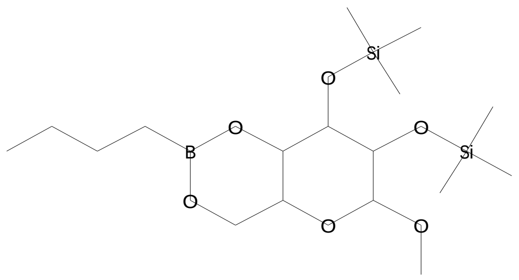


Hit Spectrum

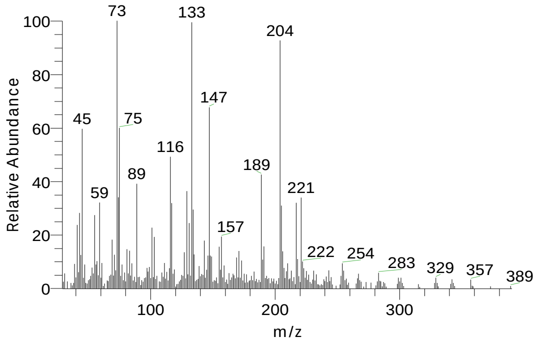

# My GC-MS Report

Compound Structure | Hit Spectrum

á-D-GALACTOPYRANOSIDE, METHYL 2,3-BIS-O-(TRIMETHYLSILYL)-, CYCLIC BUTYLBORONATE
Formula C17H37BO6Si2, MW 404, CAS# 56211-10-0, Entry# 257666

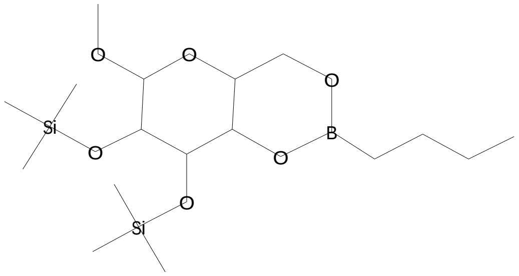

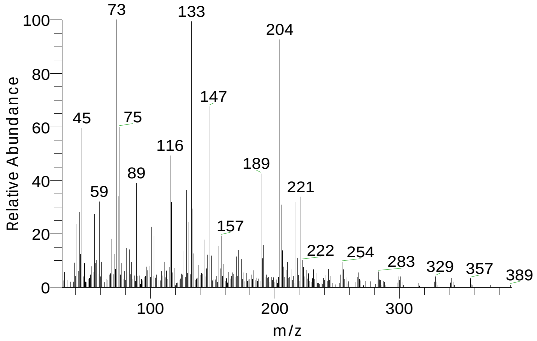


á-D-Galactopyranoside, methyl 2,6-bis-O-(trimethylsilyl)-, cyclic butylboronate
Formula C17H37BO6Si2, MW 404, CAS# 56211-13-3, Entry# 42856

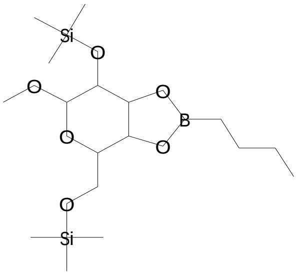

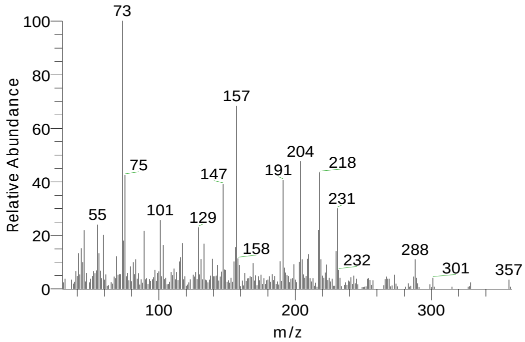


á-D-GALACTOPYRANOSIDE, METHYL 2,6-BIS-O-(TRIMETHYLSILYL)-, CYCLIC BUTYLBORONATE
Formula C17H37BO6Si2, MW 404, CAS# 56211-13-3, Entry# 257660

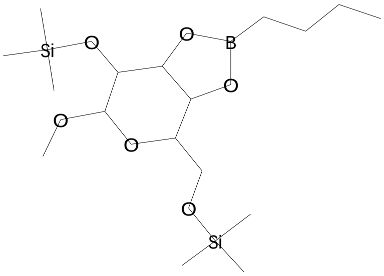

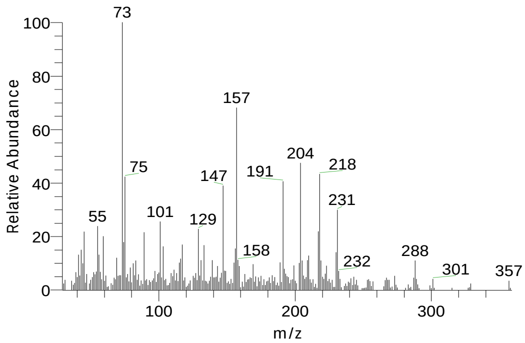


á-D-Galactopyranoside, methyl 2,6-bis-O-(trimethylsilyl)-, cyclic methylboronate
Formula C14H31BO6Si2, MW 362, CAS# 56211-06-4, Entry# 41379

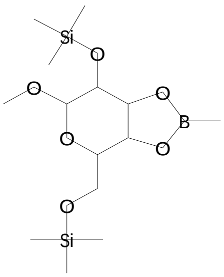

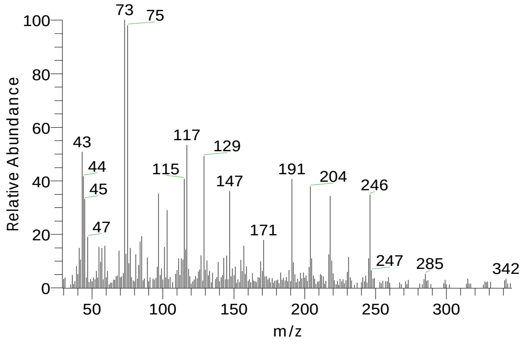

# *My GC-MS Report*

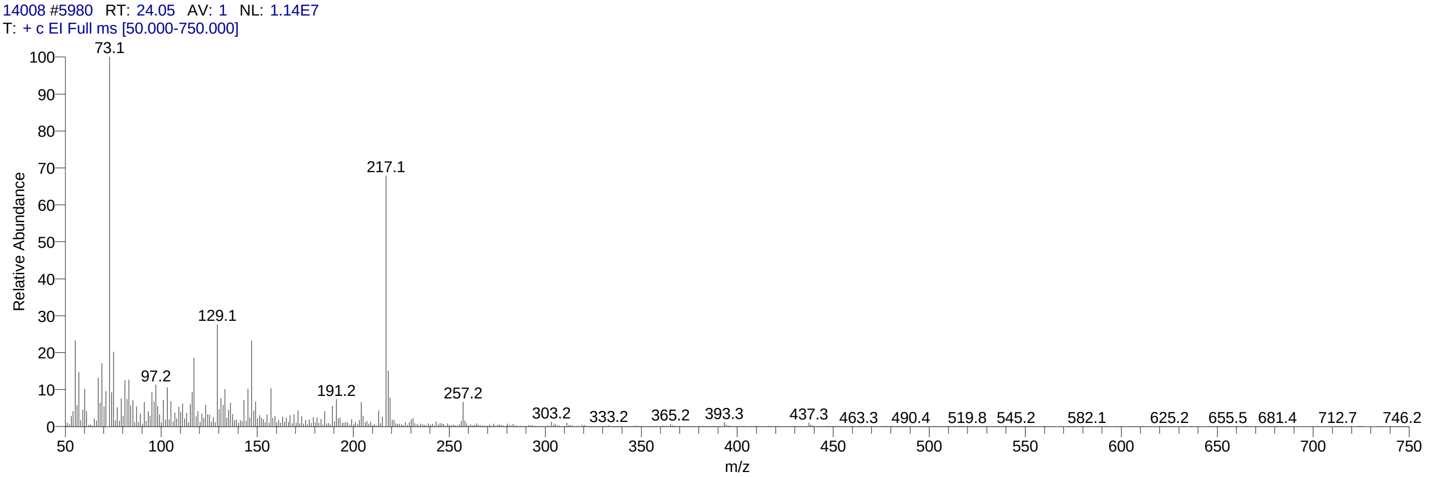


| RT | Compound Name | Area % | MF | Molecular Formula | Molecular Weight | Cas # | Library |
|---|---|---|---|---|---|---|---|
| 24.05 | á-D-Galactopyranoside, methyl 2,6-bis-O-(trimethylsilyl)-, cyclic methylboronate | 0.85 | 739 | C14H31BO6Si2 | 362 | 56211-06-4 | mainlib |
| 24.05 | á-D-GALACTOPYRANOSIDE, METHYL 2,6-BIS-O-(TRIMETHYLSILYL)-, CYCLIC METHYLBORONATE | 0.85 | 739 | C14H31BO6Si2 | 362 | 56211-06-4 | WileyRegistry8e |
| 24.05 | D-GLUCOSE, 6-O-à-D-GALACTOPYRANOSYL-, BIS-O-(TRIMETHYLSILYL) DERIV., CYCLIC TRIS(METHYLBORONATE) | 0.85 | 733 | C21H41B3O11Si2 | 558 | 72347-76-3 | WileyRegistry8e |
| 24.05 | à-D-Glucopyranosiduronic acid, 3-(5-ethylhexahydro-2,4,6-trioxo-5-pyrimidinyl)-1,1-dimethylpropyl 2,3,4-tris-O-(trimethylsilyl)-, methyl ester | 0.85 | 711 | C27H52N2O10Si3 | 648 | 55556-81-5 | mainlib |
| 24.05 | à-D-Galactopyranoside, methyl 2,6-bis-O-(trimethylsilyl)-, cyclic methylboronate | 0.85 | 738 | C14H31BO6Si2 | 362 | 54400-89-4 | mainlib |

Compound Structure | Hit Spectrum

á-D-Galactopyranoside, methyl 2,6-bis-O-(trimethylsilyl)-, cyclic methylboronate
Formula C14H31BO6Si2, MW 362, CAS# 56211-06-4, Entry# 41379

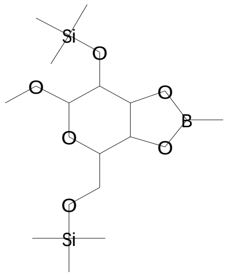


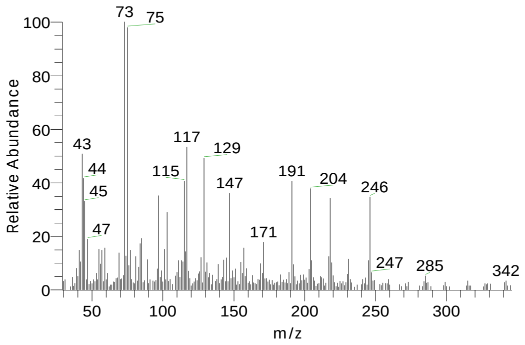

# *My GC-MS Report*

Compound Structure | Hit Spectrum

á-D-GALACTOPYRANOSIDE, METHYL 2,6-BIS-O-(TRIMETHYLSILYL)-, CYCLIC METHYLBORONATE
Formula C14H31BO6Si2, MW 362, CAS# 56211-06-4, Entry# 235850

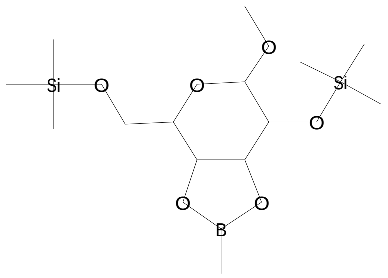

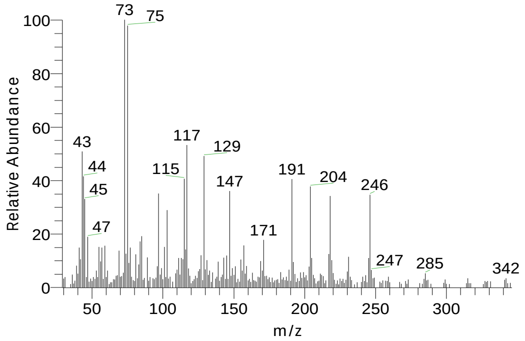


Formula C21H41B3O11Si2, MW 558, CAS# 72347-76-3, Entry# 293114

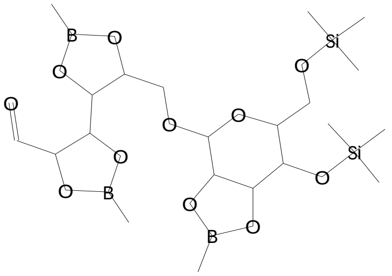

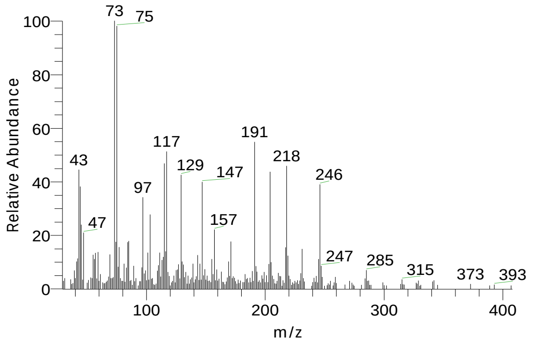


Formula C27H52N2O10Si3, MW 648, CAS# 55556-81-5, Entry# 43396

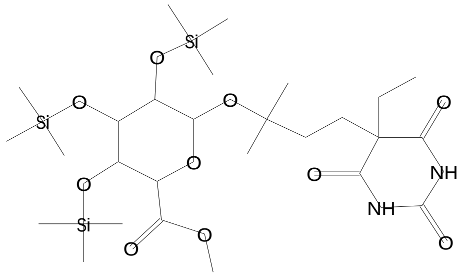

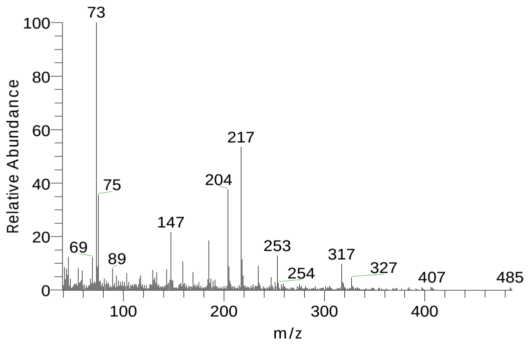


à-D-Galactopyranoside, methyl 2,6-bis-O-(trimethylsilyl)-, cyclic methylboronate
Formula C14H31BO6Si2, MW 362, CAS# 54400-89-4, Entry# 42101

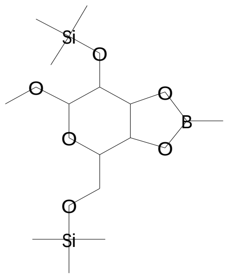

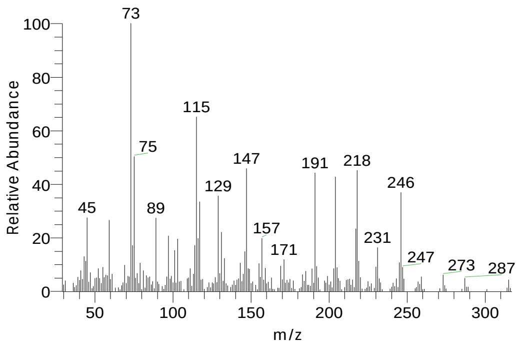

# My GC-MS Report

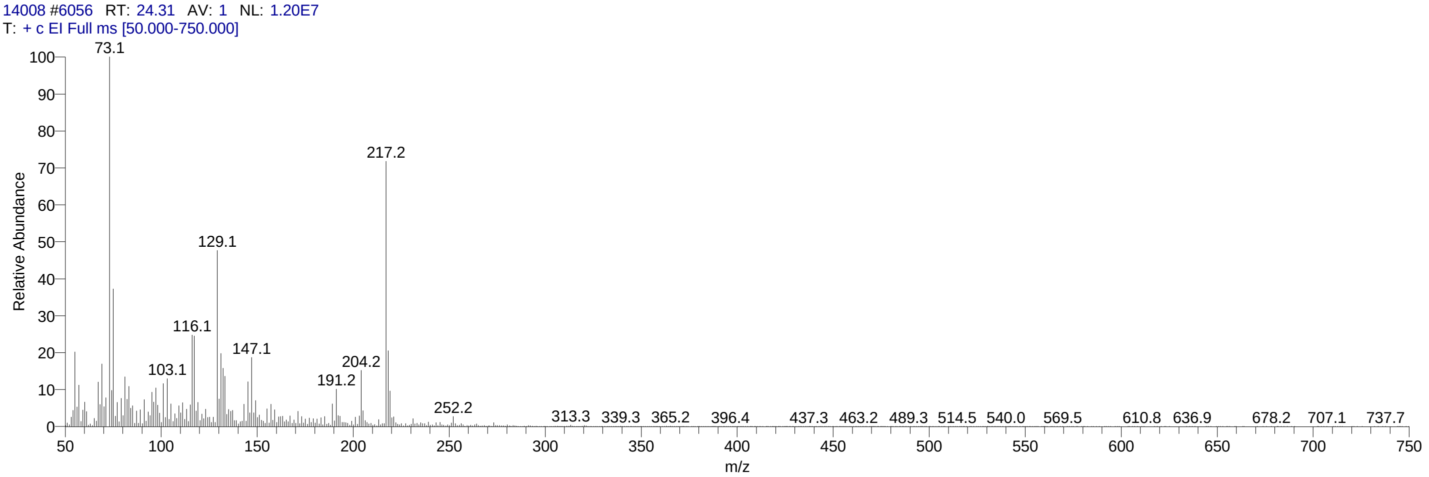


| RT | Compound Name | Area % | MF | Molecular Formula | Molecular Weight | Cas # | Library |
|---|---|---|---|---|---|---|---|
| 24.31 | á-D-Galactopyranoside, methyl 2,3-bis-O-(trimethylsilyl)-, cyclic butylboronate | 1.80 | 740 | C17H37BO6Si2 | 404 | 56211-10-0 | mainlib |
| 24.31 | á-D-GALACTOPYRANOSIDE, METHYL 2,3-BIS-O-(TRIMETHYLSILYL)-, CYCLIC BUTYLBORONATE | 1.80 | 740 | C17H37BO6Si2 | 404 | 56211-10-0 | WileyRegistry8e |
| 24.31 | MANNOFURANOSIDE, METHYL 2,3,5,6-TETRAKIS-O-(TRIMETHYLSILYL)-, à-D- | 1.80 | 814 | C19H46O6Si4 | 482 | 6737-01-5 | WileyRegistry8e |
| 24.31 | á-D-GALACTOPYRANOSIDE, METHYL 2,6-BIS-O-(TRIMETHYLSILYL)-, CYCLIC BUTYLBORONATE | 1.80 | 731 | C17H37BO6Si2 | 404 | 56211-13-3 | WileyRegistry8e |
| 24.31 | à-D-Galactopyranose, 1,2,3-tris-O-(trimethylsilyl)-, cyclic methylboronate | 1.80 | 745 | C16H37BO6Si3 | 420 | 56196-95-3 | mainlib |

Compound Structure | Hit Spectrum

á-D-Galactopyranoside, methyl 2,3-bis-O-(trimethylsilyl)-, cyclic butylboronate
Formula C17H37BO6Si2, MW 404, CAS# 56211-10-0, Entry# 42381

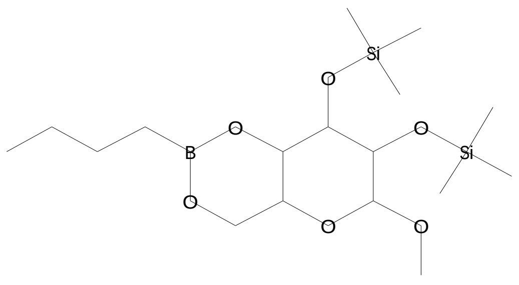

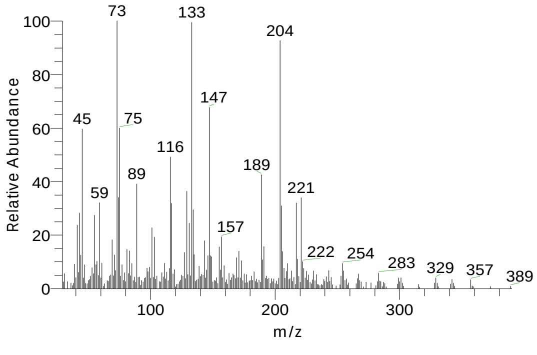


á-D-GALACTOPYRANOSIDE, METHYL 2,3-BIS-O-(TRIMETHYLSILYL)-, CYCLIC BUTYLBORONATE
Formula C17H37BO6Si2, MW 404, CAS# 56211-10-0, Entry# 257666

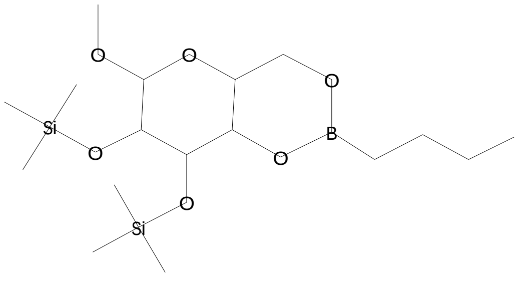

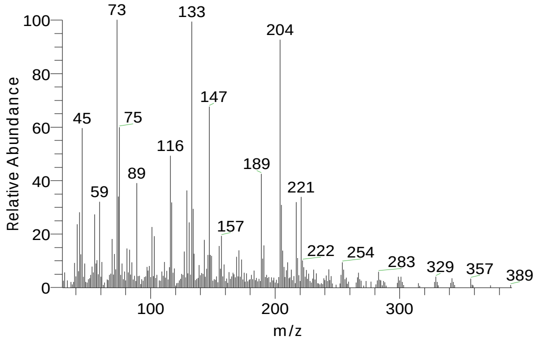

# My GC-MS Report

Compound Structure | Hit Spectrum

MANNOFURANOSIDE, METHYL 2,3,5,6-TETRAKIS-O-(TRIMETHYLSILYL)-, à-D-
Formula C19H46O6Si4, MW 482, CAS# 6737-01-5, Entry# 282094
METHYL 2,3,5,6-TETRAKIS-O-(TRIMETHYLSILYL)HEXOFURANOSIDE #

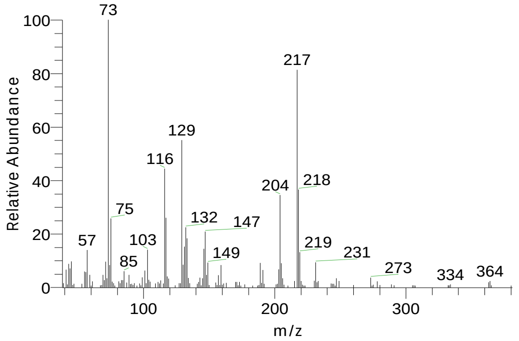


á-D-GALACTOPYRANOSIDE, METHYL 2,6-BIS-O-(TRIMETHYLSILYL)-, CYCLIC BUTYLBORONATE
Formula C17H37BO6Si2, MW 404, CAS# 56211-13-3, Entry# 257660

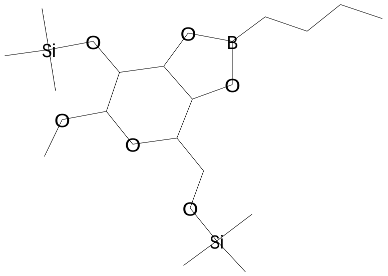


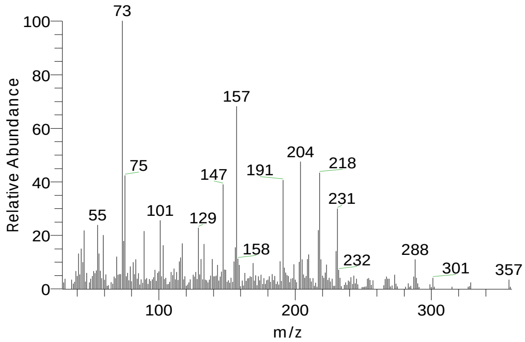


à-D-Galactopyranose, 1,2,3-tris-O-(trimethylsilyl)-, cyclic methylboronate
Formula C16H37BO6Si3, MW 420, CAS# 56196-95-3, Entry# 41501

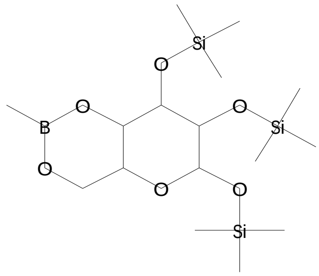


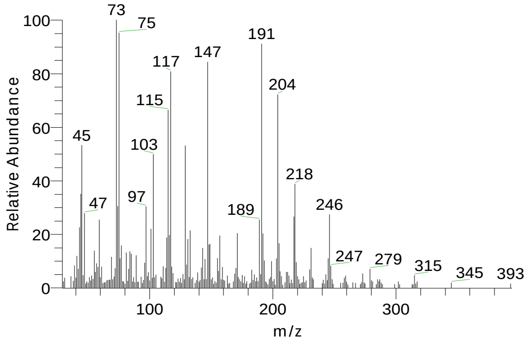


14008 #6093 RT: 24.43 AV: 1 NL: 1.55E7
T: + c EI Full ms [50.000-750.000]

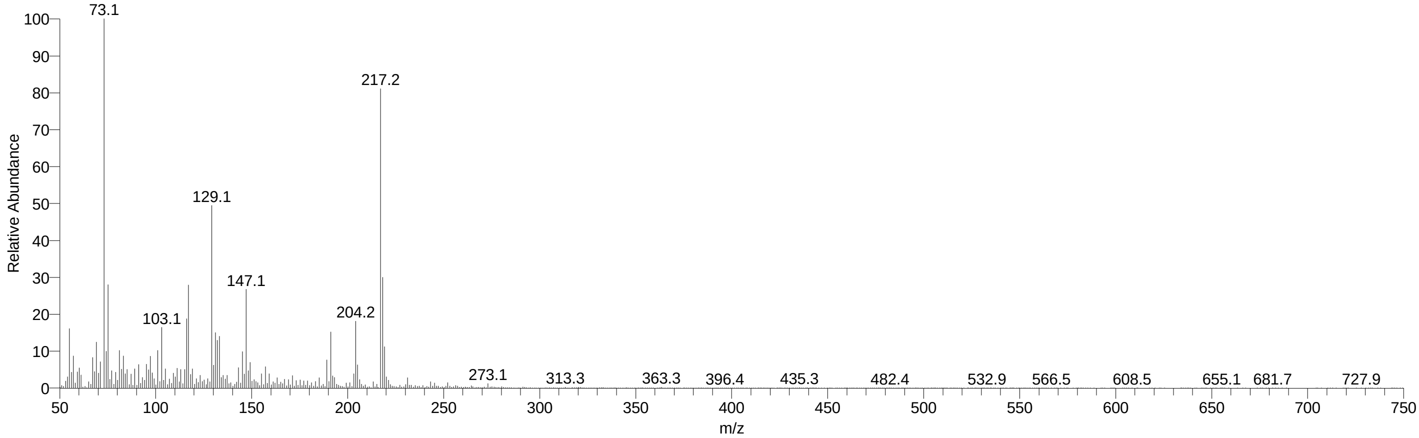


| RT | Compound Name | Area % | MF | Molecular Formula | Molecular Weight | Cas # | Library |
|---|---|---|---|---|---|---|---|
| 24.43 | MANNOFURANOSIDE, METHYL 2,3,5,6-TETRAKIS-O-(TRIMETHYLSILYL)-, à-D- | 2.50 | 838 | C19H46O6Si4 | 482 | 6737-01-5 | WileyRegistry8e |

# *My GC-MS Report*

| RT | Compound Name | Area % | MF | Molecular Formula | Molecular Weight | Cas # | Library |
|---|---|---|---|---|---|---|---|
| 24.43 | GLUCOFURANOSIDE, METHYL 2,3,5,6-TETRAKIS-O-(TRIMETHYLSILYL)-, à-D- | 2.50 | 836 | C19H46O6Si4 | 482 | 6736-96-5 | WileyRegistry8e |
| 24.43 | Methyl à-D-glucofuranoside, 4TMS derivative | 2.50 | 836 | C19H46O6Si4 | 482 | 6736-96-5 | mainlib |
| 24.43 | à-D-Galactopyranose, 1,2,3-tris-O-(trimethylsilyl)-, cyclic methylboronate | 2.50 | 761 | C16H37BO6Si3 | 420 | 56196-95-3 | mainlib |
| 24.43 | à-D-GALACTOPYRANOSE, 1,2,3-TRIS-O-(TRIMETHYLSILYL)-, CYCLIC METHYLBORONATE | 2.50 | 761 | C16H37BO6Si3 | 420 | 56196-95-3 | WileyRegistry8e |

| Compound Structure | Hit Spectrum |
|---|---|

MANNOFURANOSIDE, METHYL 2,3,5,6-TETRAKIS-O-(TRIMETHYLSILYL)-, à-D-
Formula C19H46O6Si4, MW 482, CAS# 6737-01-5, Entry# 282094
METHYL 2,3,5,6-TETRAKIS-O-(TRIMETHYLSILYL)HEXOFURANOSIDE #

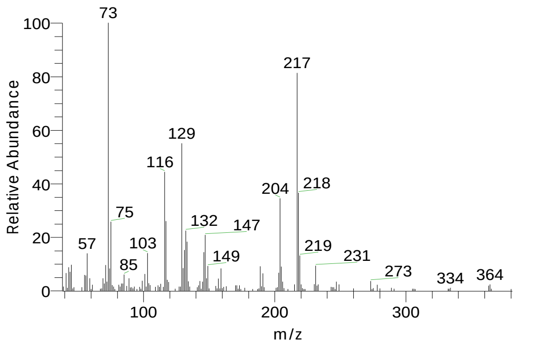


GLUCOFURANOSIDE, METHYL 2,3,5,6-TETRAKIS-O-(TRIMETHYLSILYL)-, à-D-
Formula C19H46O6Si4, MW 482, CAS# 6736-96-5, Entry# 282095
METHYL 2,3,5,6-TETRAKIS-O-(TRIMETHYLSILYL)HEXOFURANOSIDE #

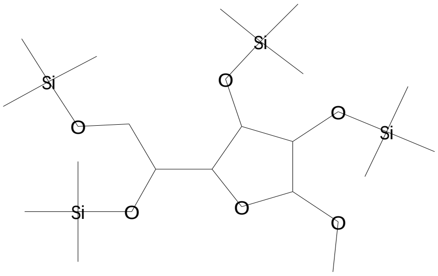


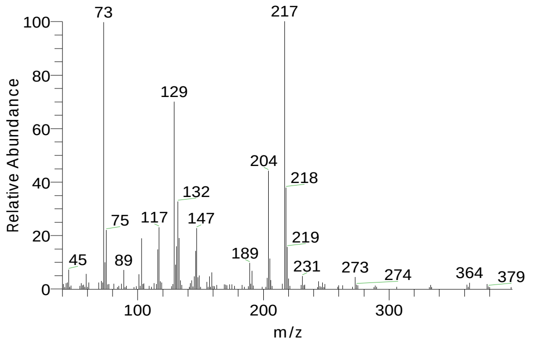


Methyl à-D-glucofuranoside, 4TMS derivative
Formula C19H46O6Si4, MW 482, CAS# 6736-96-5, Entry# 194105
Glucofuranoside, methyl 2,3,5,6-tetrakis-O-(trimethylsilyl)-, à-D-

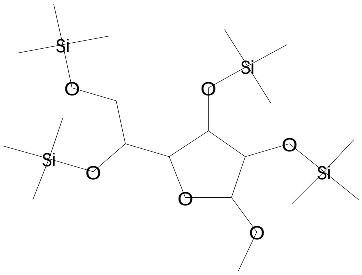


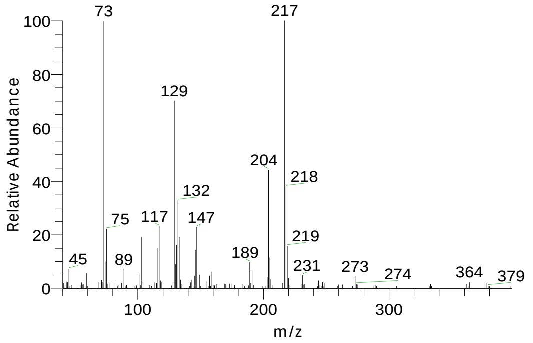

# My GC-MS Report

Compound Structure | Hit Spectrum

à-D-Galactopyranose, 1,2,3-tris-O-(trimethylsilyl)-, cyclic methylboronate
Formula C16H37BO6Si3, MW 420, CAS# 56196-95-3, Entry# 41501

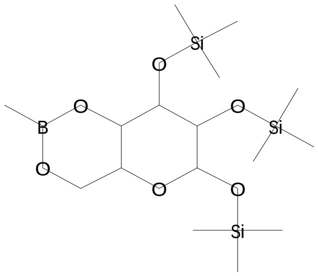


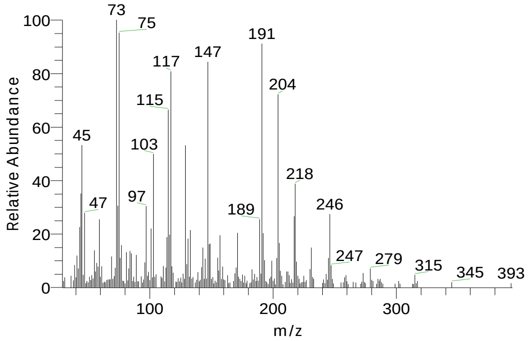


à-D-GALACTOPYRANOSE, 1,2,3-TRIS-O-(TRIMETHYLSILYL)-, CYCLIC METHYLBORONATE
Formula C16H37BO6Si3, MW 420, CAS# 56196-95-3, Entry# 264348

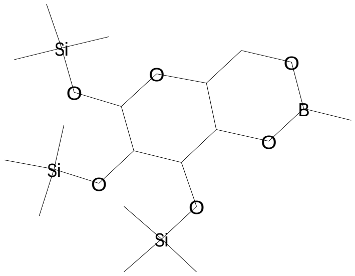


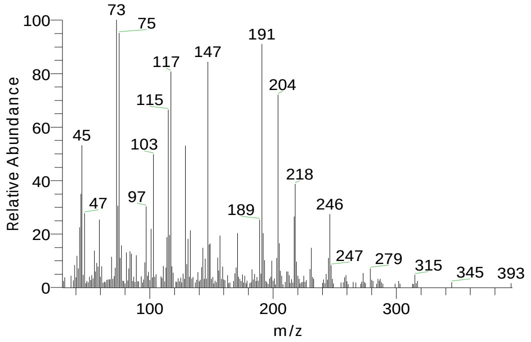


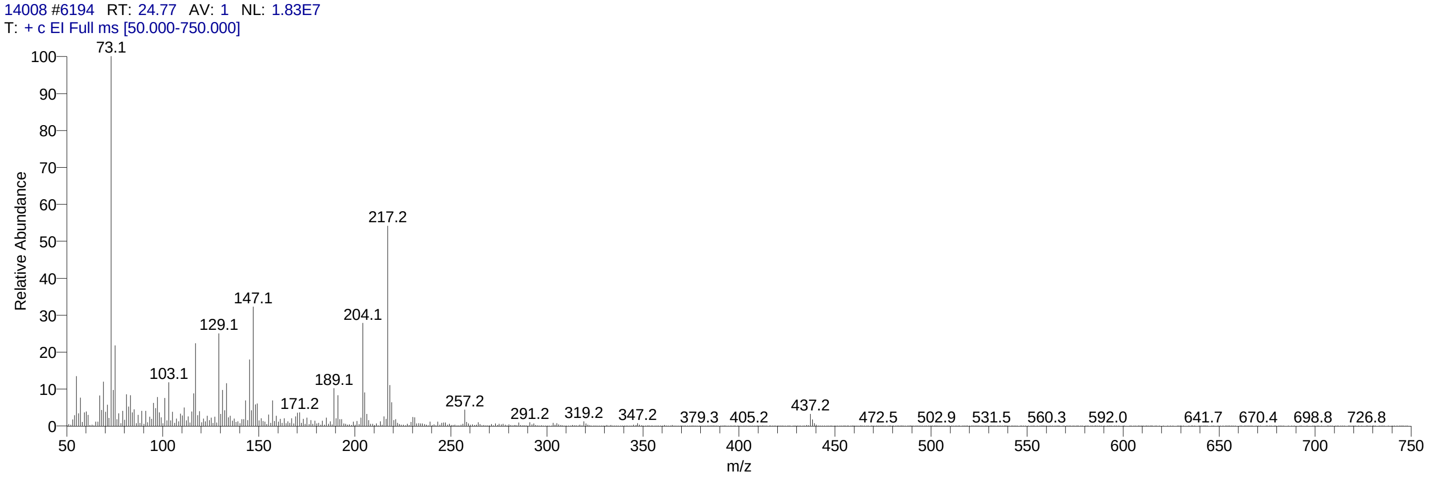


| RT | Compound Name | Area % | MF | Molecular Formula | Molecular Weight | Cas # | Library |
|---|---|---|---|---|---|---|---|
| 24.77 | à-D-Galactopyranose, 1,2,3-tris-O-(trimethylsilyl)-, cyclic methylboronate | 1.77 | 769 | C16H37BO6Si3 | 420 | 56196-95-3 | mainlib |
| 24.77 | à-D-GALACTOPYRANOSE, 1,2,3-TRIS-O-(TRIMETHYLSILYL)-, CYCLIC METHYLBORONATE | 1.77 | 769 | C16H37BO6Si3 | 420 | 56196-95-3 | WileyRegistry8e |
| 24.77 | á-D-Galactopyranoside, methyl 2,3-bis-O-(trimethylsilyl)-, cyclic butylboronate | 1.77 | 758 | C17H37BO6Si2 | 404 | 56211-10-0 | mainlib |
| 24.77 | á-D-GALACTOPYRANOSIDE, METHYL 2,3-BIS-O-(TRIMETHYLSILYL)-, CYCLIC BUTYLBORONATE | 1.77 | 758 | C17H37BO6Si2 | 404 | 56211-10-0 | WileyRegistry8e |
| 24.77 | D-(-)-Tagatofuranose, pentakis(trimethylsilyl) ether (isomer 1) | 1.77 | 765 | C21H52O6Si5 | 540 | NA | mainlib |

# My GC-MS Report

Compound Structure | Hit Spectrum

à-D-Galactopyranose, 1,2,3-tris-O-(trimethylsilyl)-, cyclic methylboronate
Formula C16H37BO6Si3, MW 420, CAS# 56196-95-3, Entry# 41501

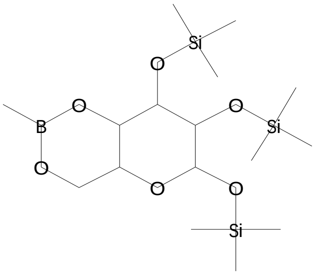

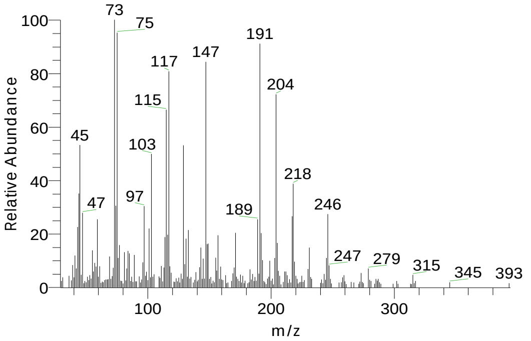


à-D-GALACTOPYRANOSE, 1,2,3-TRIS-O-(TRIMETHYLSILYL)-, CYCLIC METHYLBORONATE
Formula C16H37BO6Si3, MW 420, CAS# 56196-95-3, Entry# 264348

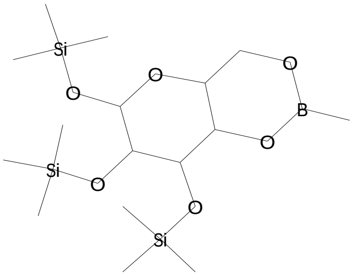

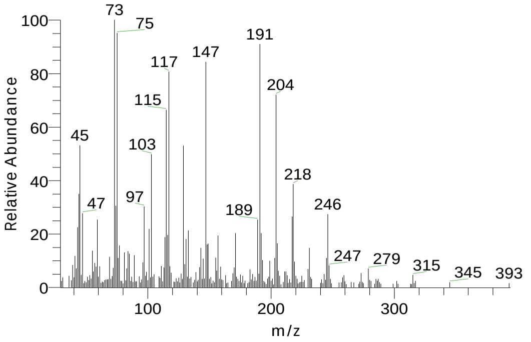


á-D-Galactopyranoside, methyl 2,3-bis-O-(trimethylsilyl)-, cyclic butylboronate
Formula C17H37BO6Si2, MW 404, CAS# 56211-10-0, Entry# 42381

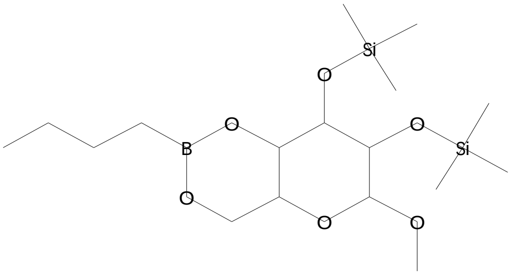

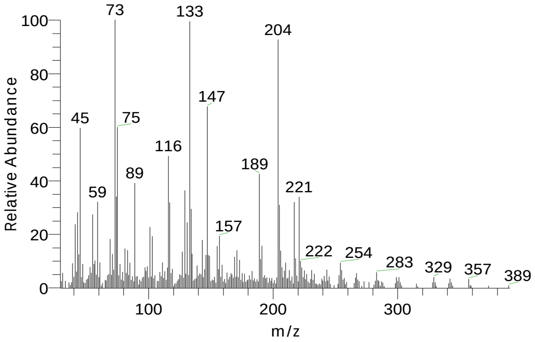


á-D-GALACTOPYRANOSIDE, METHYL 2,3-BIS-O-(TRIMETHYLSILYL)-, CYCLIC BUTYLBORONATE
Formula C17H37BO6Si2, MW 404, CAS# 56211-10-0, Entry# 257666

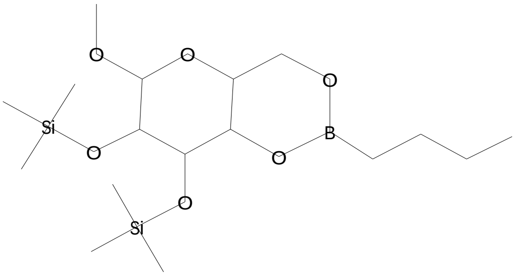

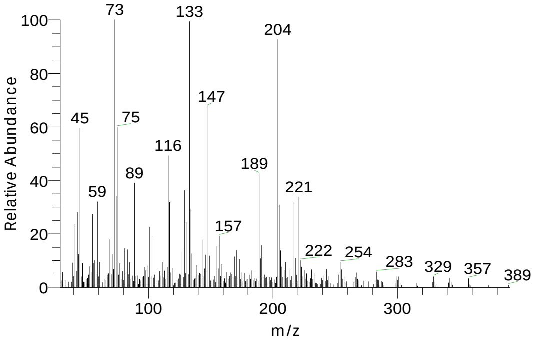

# My GC-MS Report

Compound Structure | Hit Spectrum

D-(-)-Tagatofuranose, pentakis(trimethylsilyl) ether (isomer 1)
Formula C21H52O6Si5, MW 540, CAS# NA, Entry# 43360
$:28PLNWQGWZBNJIQM-UHFFFAOYSA-N

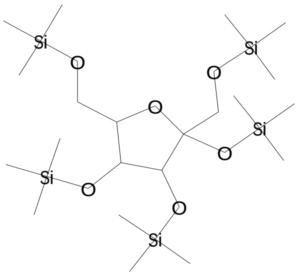

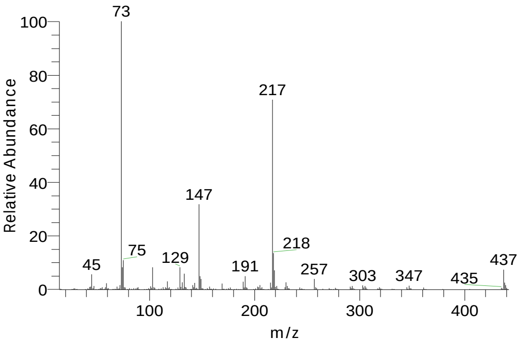


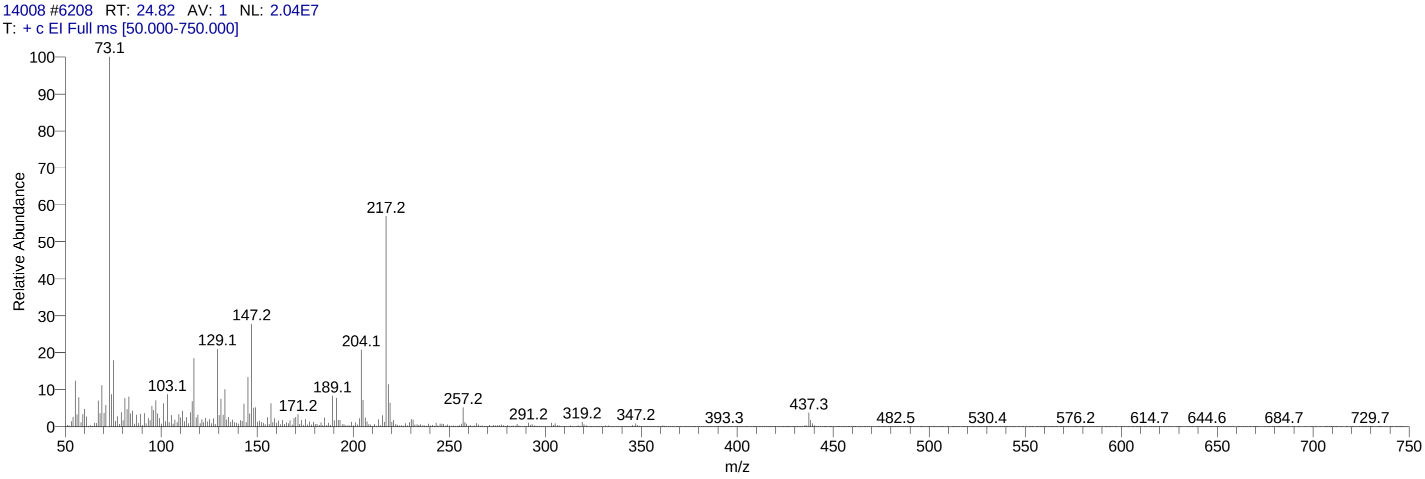


| RT | Compound Name | Area % | MF | Molecular Formula | Molecular Weight | Cas # | Library |
|---|---|---|---|---|---|---|---|
| 24.82 | D-(-)-Tagatofuranose, pentakis(trimethylsilyl) ether (isomer 1) | 0.91 | 793 | C21H52O6Si5 | 540 | NA | mainlib |
| 24.82 | D-(-)-Fructofuranose, pentakis(trimethylsilyl) ether (isomer 2) | 0.91 | 782 | C21H52O6Si5 | 540 | NA | mainlib |
| 24.82 | D-(-)-Fructofuranose, pentakis(trimethylsilyl) ether (isomer 1) | 0.91 | 779 | C21H52O6Si5 | 540 | NA | mainlib |
| 24.82 | D-Psicofuranose, pentakis(trimethylsilyl) ether (isomer 2) | 0.91 | 777 | C21H52O6Si5 | 540 | NA | mainlib |
| 24.82 | D-Psicofuranose, pentakis(trimethylsilyl) ether (isomer 1) | 0.91 | 791 | C21H52O6Si5 | 540 | NA | mainlib |


Compound Structure | Hit Spectrum

D-(-)-Tagatofuranose, pentakis(trimethylsilyl) ether (isomer 1)
Formula C21H52O6Si5, MW 540, CAS# NA, Entry# 43360
$:28PLNWQGWZBNJIQM-UHFFFAOYSA-N


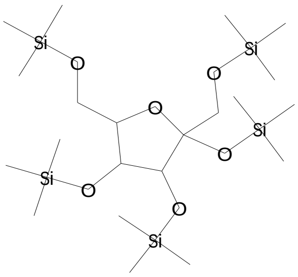

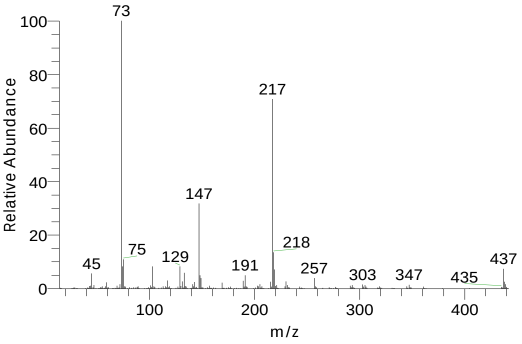

# *My GC-MS Report*

Compound Structure | Hit Spectrum

D-(-)-Fructofuranose, pentakis(trimethylsilyl) ether (isomer 2)
Formula C21H52O6Si5, MW 540, CAS# NA, Entry# 43365
$:28PLNWQGWZBNJIQM-UHFFFAOYSA-N

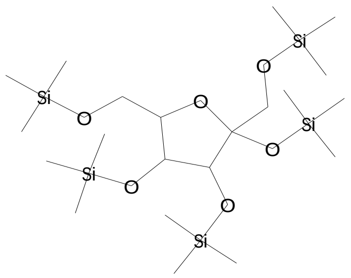

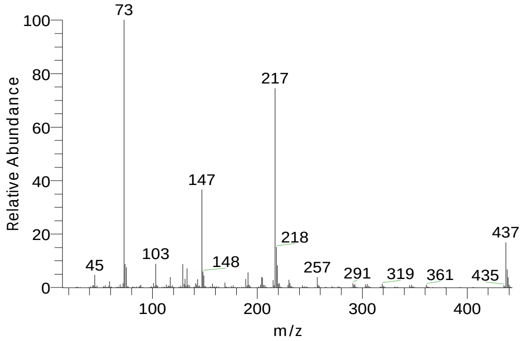


D-(-)-Fructofuranose, pentakis(trimethylsilyl) ether (isomer 1)
Formula C21H52O6Si5, MW 540, CAS# NA, Entry# 43364
$:28PLNWQGWZBNJIQM-UHFFFAOYSA-N

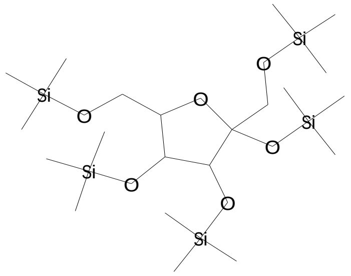

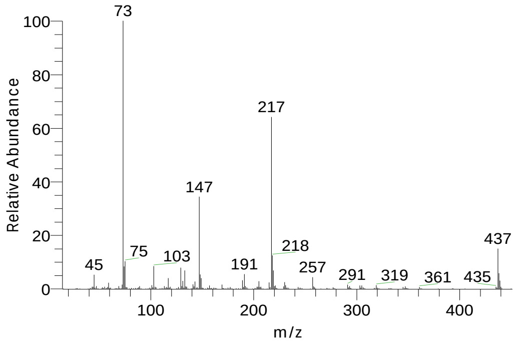


D-Psicofuranose, pentakis(trimethylsilyl) ether (isomer 2)
Formula C21H52O6Si5, MW 540, CAS# NA, Entry# 43362
$:28PLNWQGWZBNJIQM-UHFFFAOYSA-N

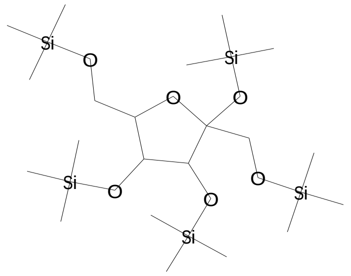

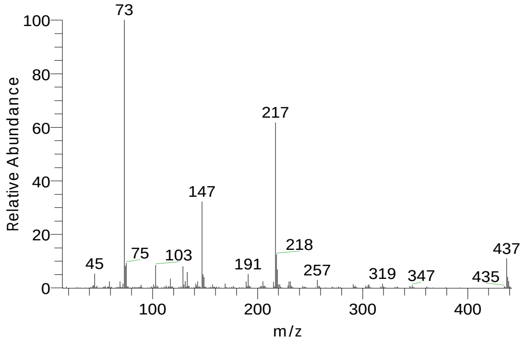


D-Psicofuranose, pentakis(trimethylsilyl) ether (isomer 1)
Formula C21H52O6Si5, MW 540, CAS# NA, Entry# 43361
$:28PLNWQGWZBNJIQM-UHFFFAOYSA-N

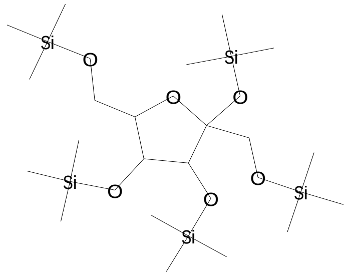

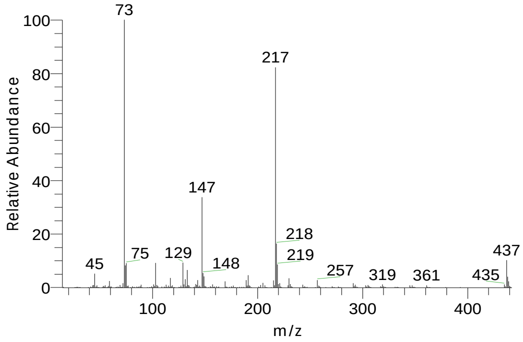

# My GC-MS Report

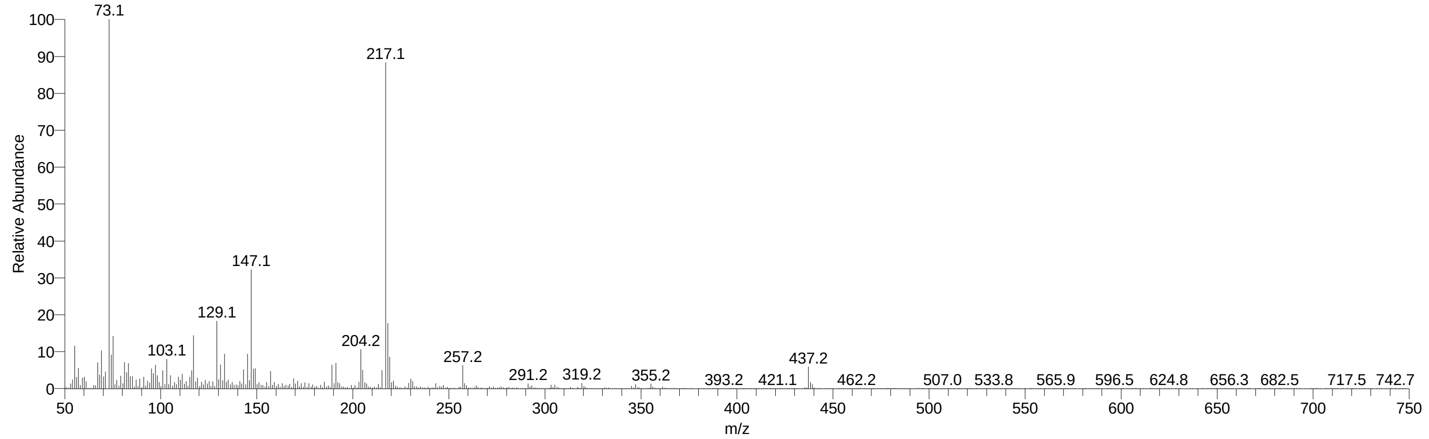


| RT | Compound Name | Area % | MF | Molecular Formula | Molecular Weight | Cas # | Library |
|---|---|---|---|---|---|---|---|
| 24.95 | D-(-)-Tagatofuranose, pentakis(trimethylsilyl) ether (isomer 1) | 2.37 | 838 | C21H52O6Si5 | 540 | NA | mainlib |
| 24.95 | D-(-)-Fructofuranose, pentakis(trimethylsilyl) ether (isomer 2) | 2.37 | 831 | C21H52O6Si5 | 540 | NA | mainlib |
| 24.95 | D-(-)-Fructofuranose, pentakis(trimethylsilyl) ether (isomer 1) | 2.37 | 829 | C21H52O6Si5 | 540 | NA | mainlib |
| 24.95 | D-Psicofuranose, pentakis(trimethylsilyl) ether (isomer 1) | 2.37 | 837 | C21H52O6Si5 | 540 | NA | mainlib |
| 24.95 | D-Psicofuranose, pentakis(trimethylsilyl) ether (isomer 2) | 2.37 | 825 | C21H52O6Si5 | 540 | NA | mainlib |

Compound Structure | Hit Spectrum

D-(-)-Tagatofuranose, pentakis(trimethylsilyl) ether (isomer 1)
Formula C21H52O6Si5, MW 540, CAS# NA, Entry# 43360
$:28PLNWQGWZBNJIQM-UHFFFAOYSA-N

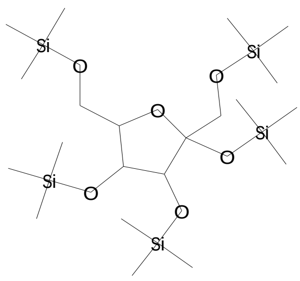

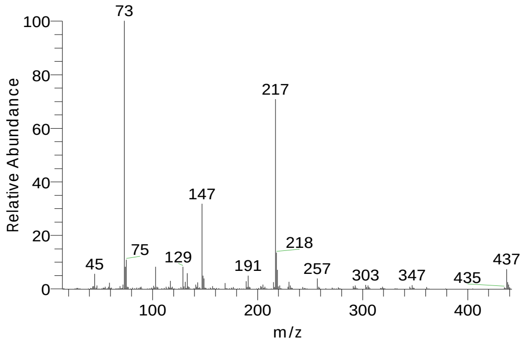


D-(-)-Fructofuranose, pentakis(trimethylsilyl) ether (isomer 2)
Formula C21H52O6Si5, MW 540, CAS# NA, Entry# 43365
$:28PLNWQGWZBNJIQM-UHFFFAOYSA-N

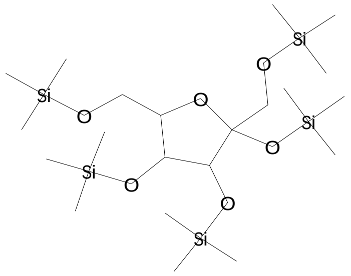

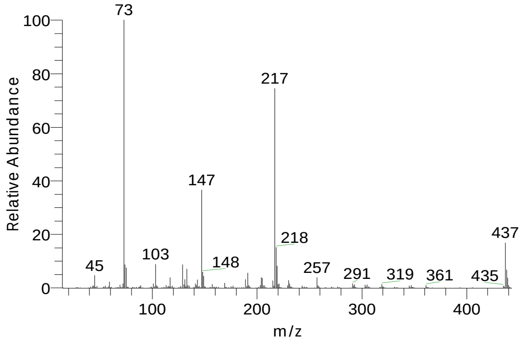

# *My GC-MS Report*

| Compound Structure | Hit Spectrum |
|---|---|

D-(-)-Fructofuranose, pentakis(trimethylsilyl) ether (isomer 1)
Formula C21H52O6Si5, MW 540, CAS# NA, Entry# 43364
$:28PLNWQGWZBNJIQM-UHFFFAOYSA-N

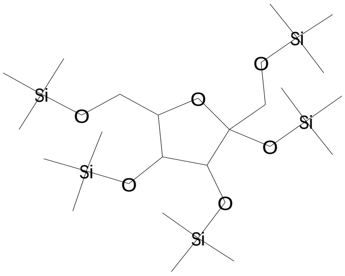


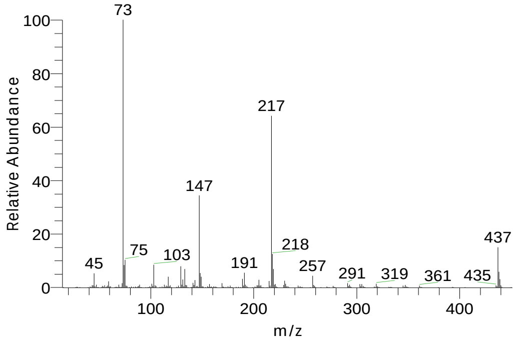


D-Psicofuranose, pentakis(trimethylsilyl) ether (isomer 1)
Formula C21H52O6Si5, MW 540, CAS# NA, Entry# 43361
$:28PLNWQGWZBNJIQM-UHFFFAOYSA-N

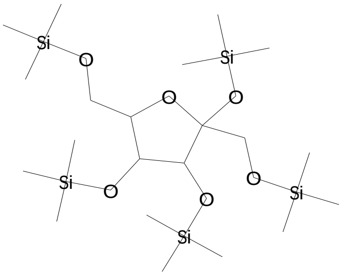

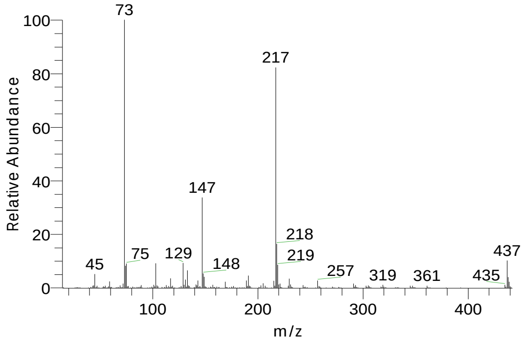


D-Psicofuranose, pentakis(trimethylsilyl) ether (isomer 2)
Formula C21H52O6Si5, MW 540, CAS# NA, Entry# 43362
$:28PLNWQGWZBNJIQM-UHFFFAOYSA-N

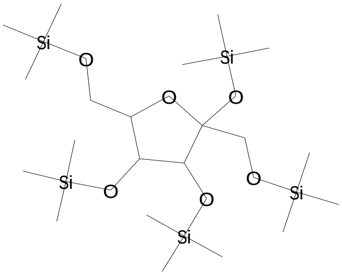

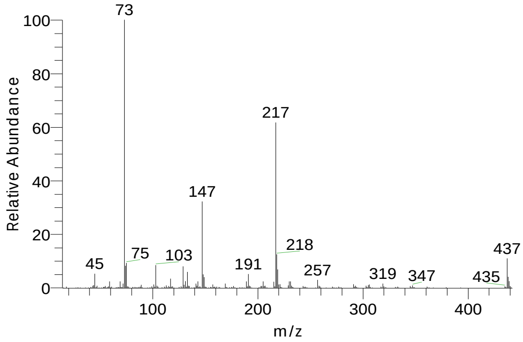


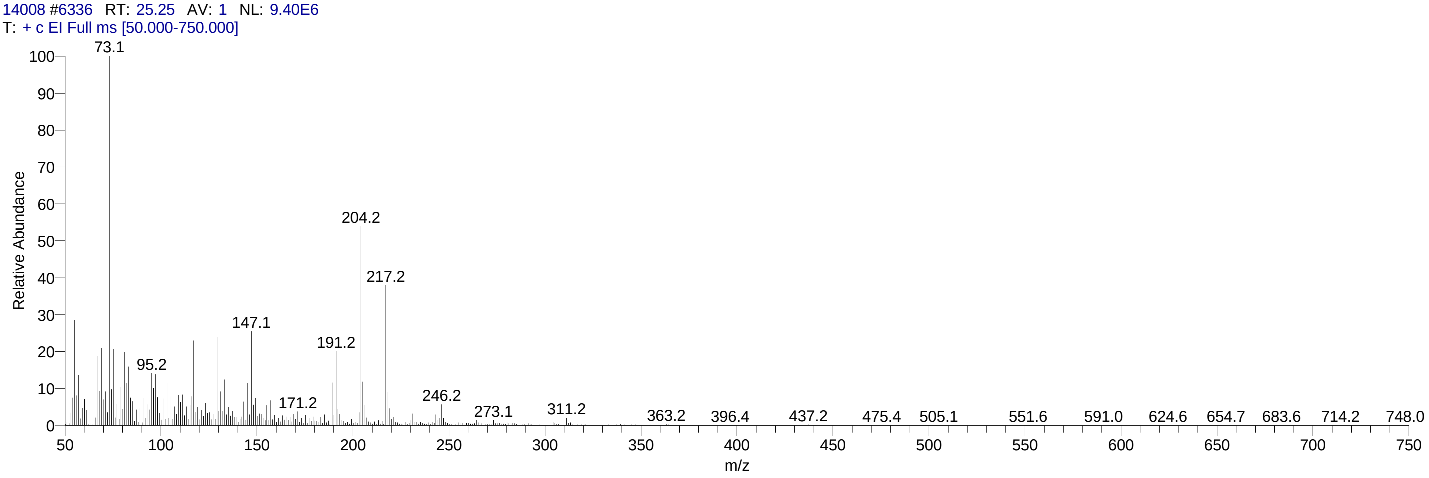


| RT | Compound Name | Area % | MF | Molecular Formula | Molecular Weight | Cas # | Library |
|---|---|---|---|---|---|---|---|
| 25.25 | à-D-Galactopyranose, 1,2,3-tris-O-(trimethylsilyl)-, cyclic methylboronate | 0.47 | 787 | C16H37BO6Si3 | 420 | 56196-95-3 | mainlib |

# My GC-MS Report

| RT | Compound Name | Area % | MF | Molecular Formula | Molecular Weight | Cas # | Library |
|---|---|---|---|---|---|---|---|
| 25.25 | à-D-GALACTOPYRANOSE, 1,2,3-TRIS-O-(TRIMETHYLSILYL)-, CYCLIC METHYLBORONATE | 0.47 | 787 | C16H37BO6Si3 | 420 | 56196-95-3 | WileyRegistry8e |
| 25.25 | á-D-Galactopyranoside, methyl 2,3-bis-O-(trimethylsilyl)-, cyclic butylboronate | 0.47 | 773 | C17H37BO6Si2 | 404 | 56211-10-0 | mainlib |
| 25.25 | á-D-GALACTOPYRANOSIDE, METHYL 2,3-BIS-O-(TRIMETHYLSILYL)-, CYCLIC BUTYLBORONATE | 0.47 | 773 | C17H37BO6Si2 | 404 | 56211-10-0 | WileyRegistry8e |
| 25.25 | à-D-MANNOPYRANOSIDE, METHYL 2,3-BIS-O-(TRIMETHYLSILYL)-, CYCLIC BUTYLBORONATE | 0.47 | 806 | C17H37BO6Si2 | 404 | 56211-09-7 | WileyRegistry8e |

Compound Structure | Hit Spectrum

à-D-Galactopyranose, 1,2,3-tris-O-(trimethylsilyl)-, cyclic methylboronate
Formula C16H37BO6Si3, MW 420, CAS# 56196-95-3, Entry# 41501

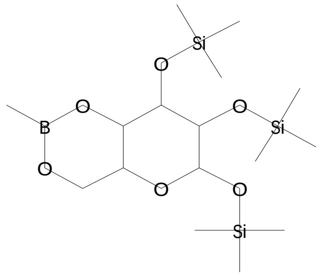


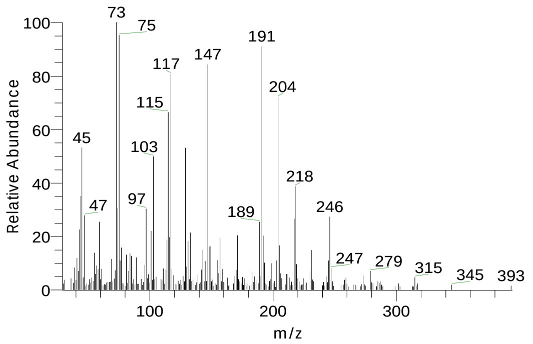


à-D-GALACTOPYRANOSE, 1,2,3-TRIS-O-(TRIMETHYLSILYL)-, CYCLIC METHYLBORONATE
Formula C16H37BO6Si3, MW 420, CAS# 56196-95-3, Entry# 264348

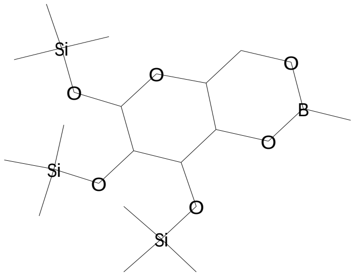


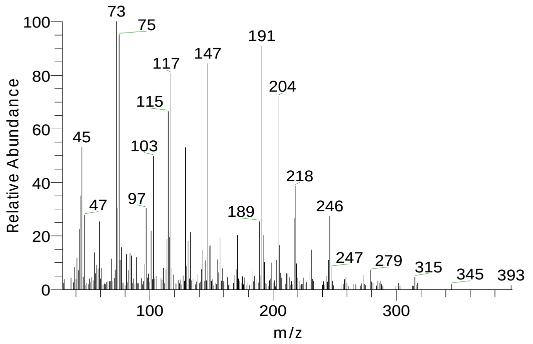


á-D-Galactopyranoside, methyl 2,3-bis-O-(trimethylsilyl)-, cyclic butylboronate
Formula C17H37BO6Si2, MW 404, CAS# 56211-10-0, Entry# 42381

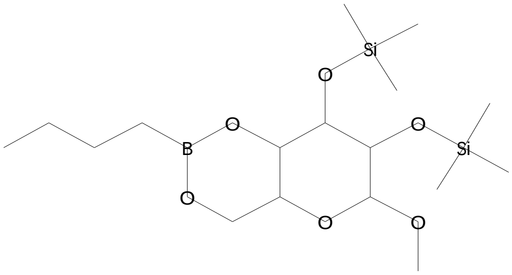


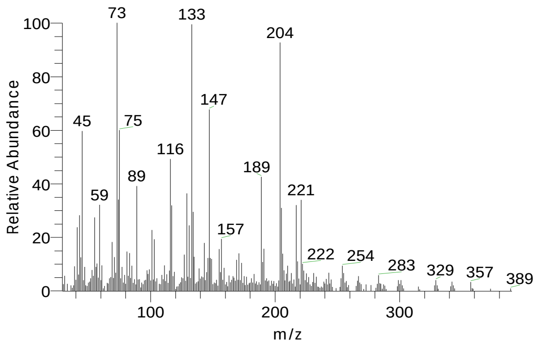

# My GC-MS Report

Compound Structure | Hit Spectrum

á-D-GALACTOPYRANOSIDE, METHYL 2,3-BIS-O-(TRIMETHYLSILYL)-, CYCLIC BUTYLBORONATE
Formula C17H37BO6Si2, MW 404, CAS# 56211-10-0, Entry# 257666

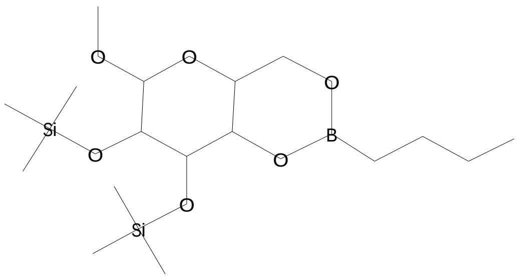

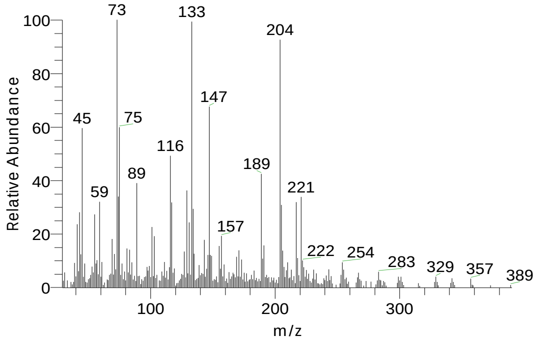


à-D-MANNOPYRANOSIDE, METHYL 2,3-BIS-O-(TRIMETHYLSILYL)-, CYCLIC BUTYLBORONATE
Formula C17H37BO6Si2, MW 404, CAS# 56211-09-7, Entry# 257667

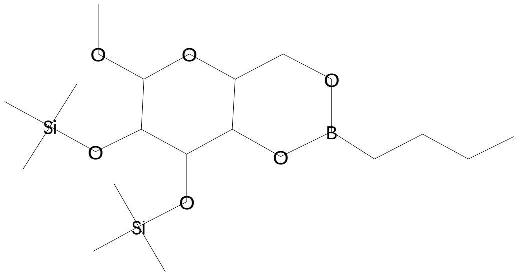

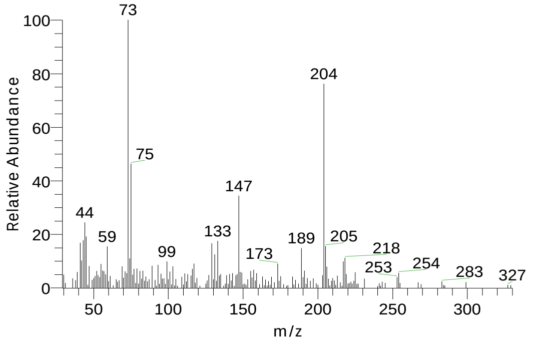


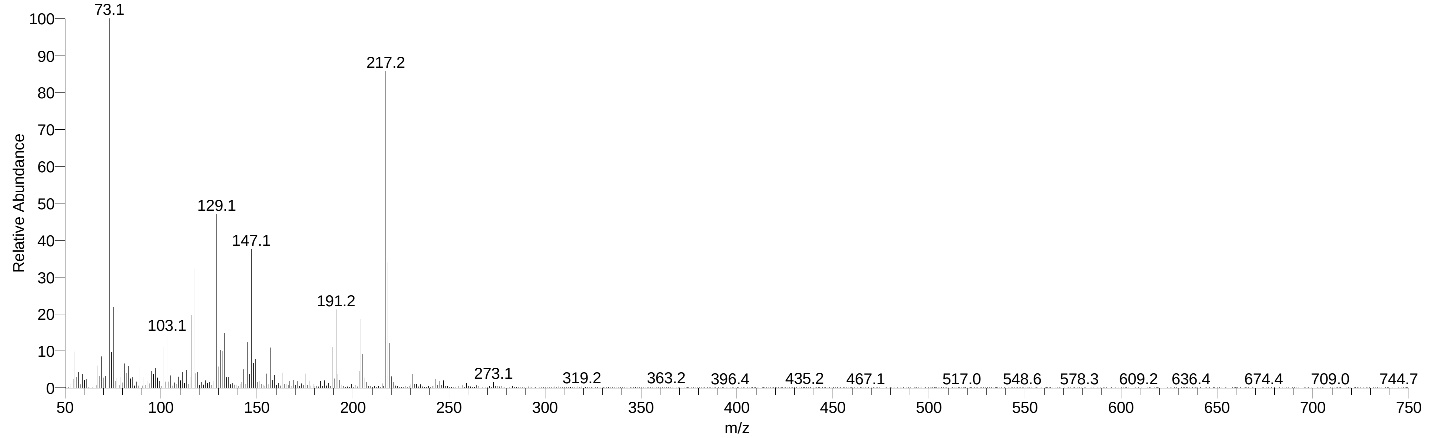


| RT | Compound Name | Area % | MF | Molecular Formula | Molecular Weight | Cas # | Library |
|---|---|---|---|---|---|---|---|
| 25.44 | MANNOFURANOSIDE, METHYL 2,3,5,6-TETRAKIS-O-(TRIMETHYLSILYL)-, à-D- | 8.97 | 839 | C19H46O6Si4 | 482 | 6737-01-5 | WileyRegistry8e |
| 25.44 | Methyl à-D-glucofuranoside, 4TMS derivative | 8.97 | 841 | C19H46O6Si4 | 482 | 6736-96-5 | mainlib |
| 25.44 | GLUCOFURANOSIDE, METHYL 2,3,5,6-TETRAKIS-O-(TRIMETHYLSILYL)-, à-D- | 8.97 | 841 | C19H46O6Si4 | 482 | 6736-96-5 | WileyRegistry8e |
| 25.44 | 1,5-Anhydrohexitol, 4TMS derivative | 8.97 | 797 | C18H44O5Si4 | 452 | NA | mainlib |
| 25.44 | á-DL-ARABINOPYRANOSE, 1,2,3,4-TETRAKIS-O-(TRIMETHYLSILYL)- | 8.97 | 807 | C17H42O5Si4 | 438 | 56271-64-8 | WileyRegistry8e |

# *My GC-MS Report*

Compound Structure | Hit Spectrum

MANNOFURANOSIDE, METHYL 2,3,5,6-TETRAKIS-O-(TRIMETHYLSILYL)-, à-D-
Formula C19H46O6Si4, MW 482, CAS# 6737-01-5, Entry# 282094
METHYL 2,3,5,6-TETRAKIS-O-(TRIMETHYLSILYL)HEXOFURANOSIDE #

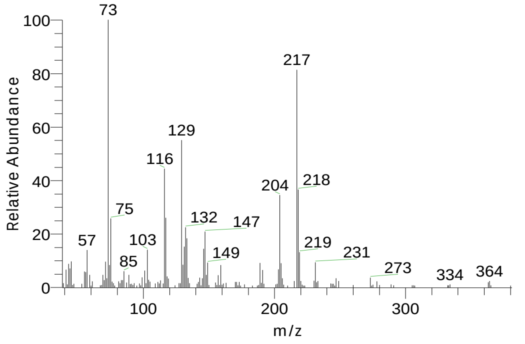


Methyl à-D-glucofuranoside, 4TMS derivative
Formula C19H46O6Si4, MW 482, CAS# 6736-96-5, Entry# 194105
Glucofuranoside, methyl 2,3,5,6-tetrakis-O-(trimethylsilyl)-, à-D-

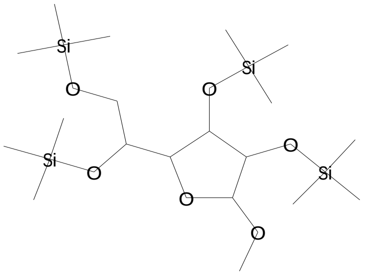

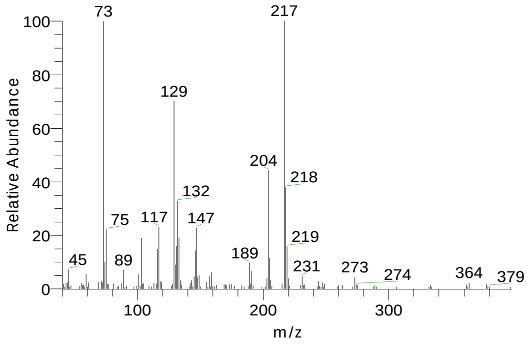


GLUCOFURANOSIDE, METHYL 2,3,5,6-TETRAKIS-O-(TRIMETHYLSILYL)-, à-D-
Formula C19H46O6Si4, MW 482, CAS# 6736-96-5, Entry# 282095
METHYL 2,3,5,6-TETRAKIS-O-(TRIMETHYLSILYL)HEXOFURANOSIDE #

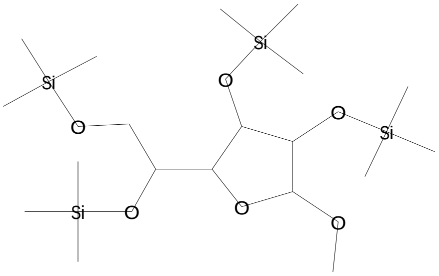

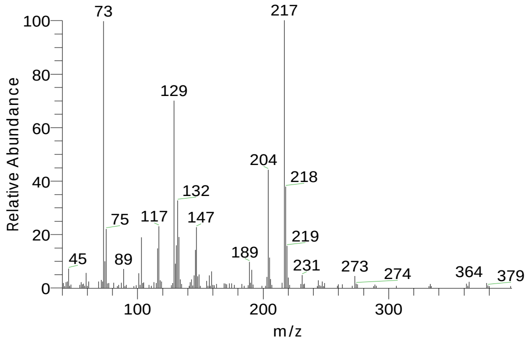


1,5-Anhydrohexitol, 4TMS derivative
Formula C18H44O5Si4, MW 452, CAS# NA, Entry# 42675
1,5-Anhydro-D-sorbitol, tetrakis(trimethylsilyl) ether

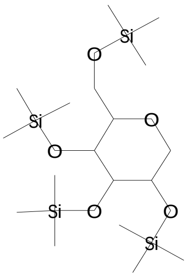

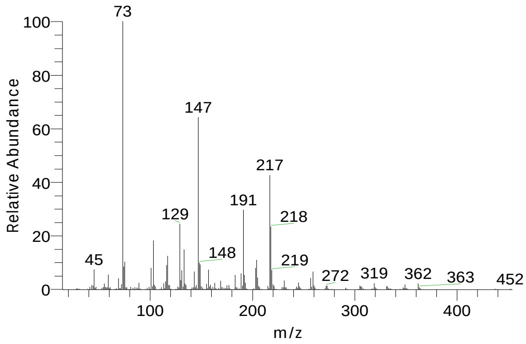

# My GC-MS Report

| Compound Structure | Hit Spectrum |
|---|---|

á-DL-ARABINOPYRANOSE, 1,2,3,4-TETRAKIS-O-(TRIMETHYLSILYL)-
Formula C17H42O5Si4, MW 438, CAS# 56271-64-8, Entry# 270549
1,2,3,4-TETRAKIS-O-(TRIMETHYLSILYL)PENTOPYRANOSE #

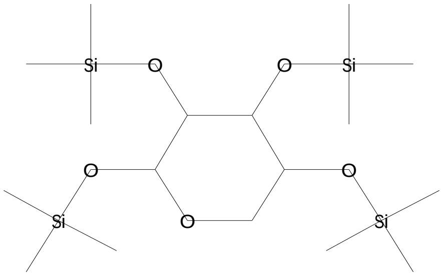

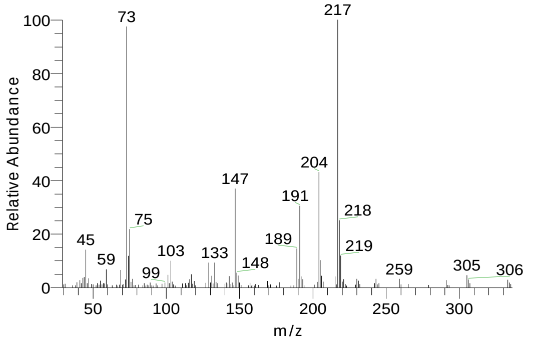

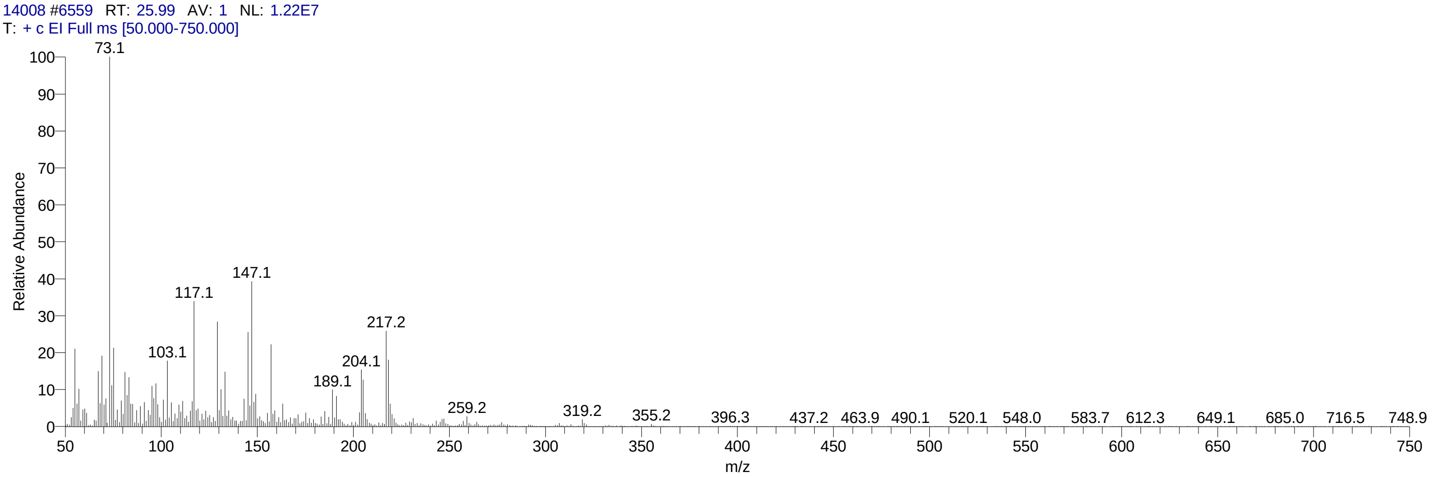


| RT | Compound Name | Area % | MF | Molecular Formula | Molecular Weight | Cas # | Library |
|---|---|---|---|---|---|---|---|
| 25.99 | à-D-Galactopyranose, 1,2,3-tris-O-(trimethylsilyl)-, cyclic methylboronate | 1.81 | 782 | C16H37BO6Si3 | 420 | 56196-95-3 | mainlib |
| 25.99 | à-D-GALACTOPYRANOSE, 1,2,3-TRIS-O-(TRIMETHYLSILYL)-, CYCLIC METHYLBORONATE | 1.81 | 782 | C16H37BO6Si3 | 420 | 56196-95-3 | WileyRegistry8e |
| 25.99 | á-D-Galactopyranoside, methyl 2,6-bis-O-(trimethylsilyl)-, cyclic methylboronate | 1.81 | 766 | C14H31BO6Si2 | 362 | 56211-06-4 | mainlib |
| 25.99 | á-D-GALACTOPYRANOSIDE, METHYL 2,6-BIS-O-(TRIMETHYLSILYL)-, CYCLIC METHYLBORONATE | 1.81 | 766 | C14H31BO6Si2 | 362 | 56211-06-4 | WileyRegistry8e |
| 25.99 | á-D-Galactopyranoside, methyl 2,3-bis-O-(trimethylsilyl)-, cyclic methylboronate | 1.81 | 754 | C14H31BO6Si2 | 362 | 56211-08-6 | mainlib |

| Compound Structure | Hit Spectrum |
|---|---|

à-D-Galactopyranose, 1,2,3-tris-O-(trimethylsilyl)-, cyclic methylboronate
Formula C16H37BO6Si3, MW 420, CAS# 56196-95-3, Entry# 41501

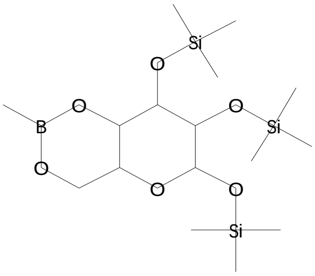

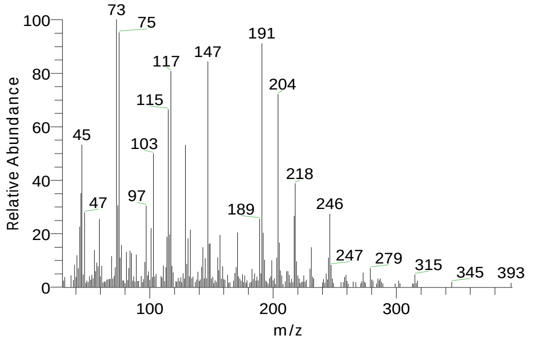

# My GC-MS Report

Compound Structure | Hit Spectrum

à-D-GALACTOPYRANOSE, 1,2,3-TRIS-O-(TRIMETHYLSILYL)-, CYCLIC METHYLBORONATE
Formula C16H37BO6Si3, MW 420, CAS# 56196-95-3, Entry# 264348

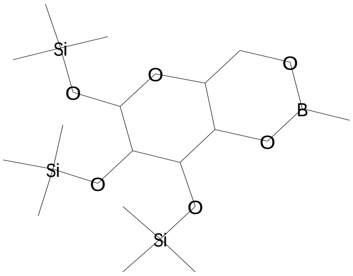

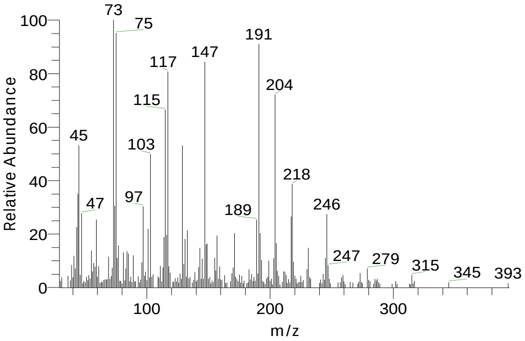


á-D-Galactopyranoside, methyl 2,6-bis-O-(trimethylsilyl)-, cyclic methylboronate
Formula C14H31BO6Si2, MW 362, CAS# 56211-06-4, Entry# 41379

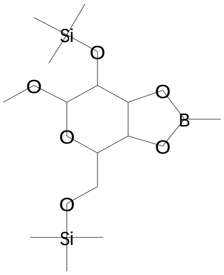

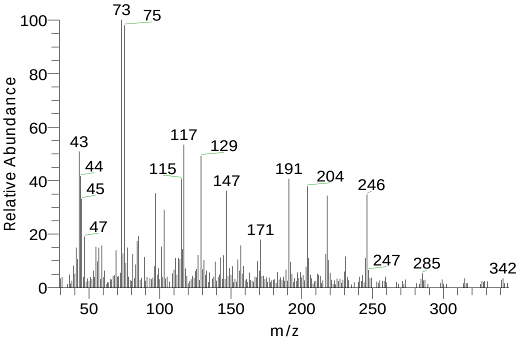


á-D-GALACTOPYRANOSIDE, METHYL 2,6-BIS-O-(TRIMETHYLSILYL)-, CYCLIC METHYLBORONATE
Formula C14H31BO6Si2, MW 362, CAS# 56211-06-4, Entry# 235850

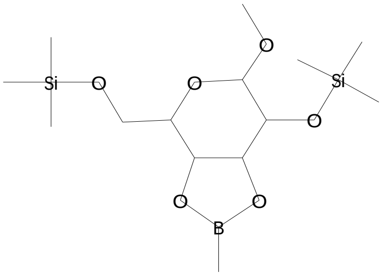

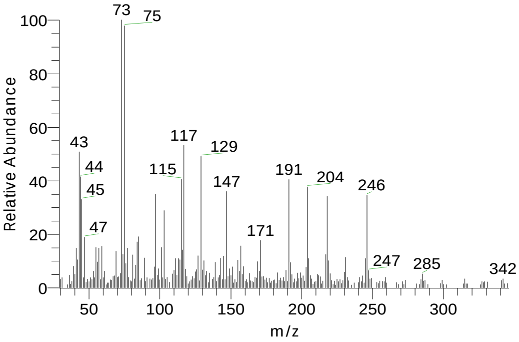


á-D-Galactopyranoside, methyl 2,3-bis-O-(trimethylsilyl)-, cyclic methylboronate
Formula C14H31BO6Si2, MW 362, CAS# 56211-08-6, Entry# 41509

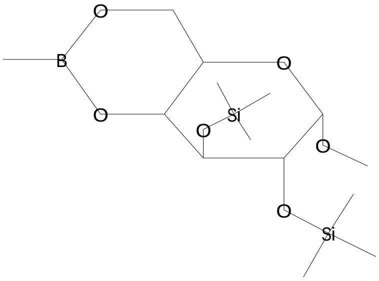

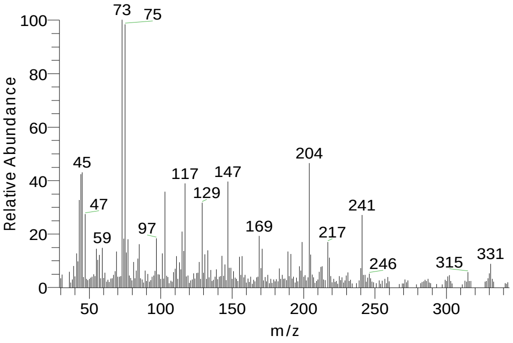

# My GC-MS Report

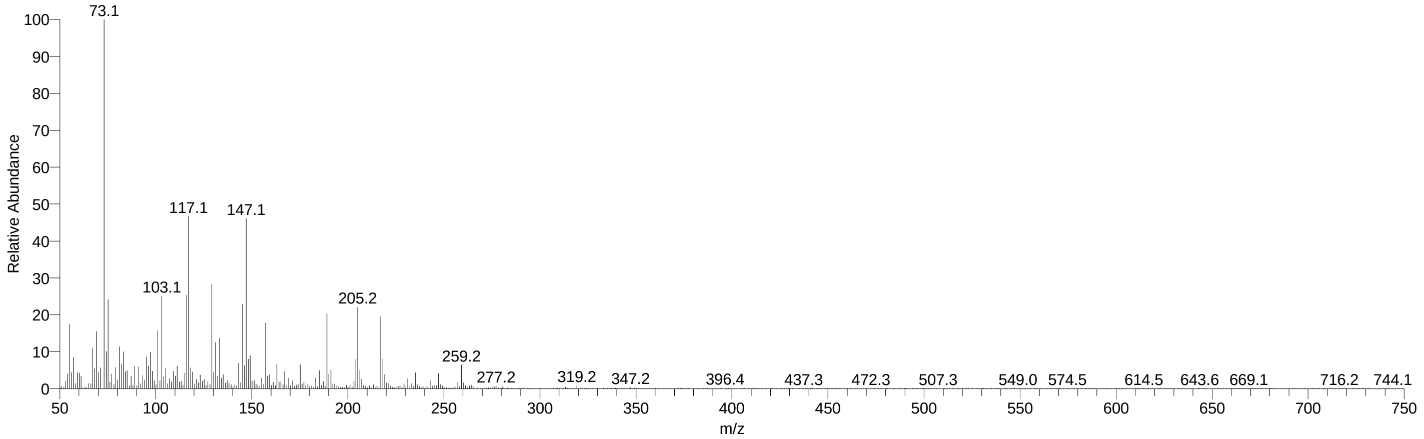


| RT | Compound Name | Area % | MF | Molecular Formula | Molecular Weight | Cas # | Library |
|---|---|---|---|---|---|---|---|
| 26.20 | á-D-Galactopyranoside, methyl 2,3-bis-O-(trimethylsilyl)-, cyclic butylboronate | 3.20 | 741 | C17H37BO6Si2 | 404 | 56211-10-0 | mainlib |
| 26.20 | á-D-GALACTOPYRANOSIDE, METHYL 2,3-BIS-O-(TRIMETHYLSILYL)-, CYCLIC BUTYLBORONATE | 3.20 | 741 | C17H37BO6Si2 | 404 | 56211-10-0 | WileyRegistry8e |
| 26.20 | à-D-Galactopyranoside, methyl 2,3-bis-O-(trimethylsilyl)-, cyclic methylboronate | 3.20 | 738 | C14H31BO6Si2 | 362 | 54400-88-3 | mainlib |
| 26.20 | à-D-GALACTOPYRANOSIDE, METHYL 2,3-BIS-O-(TRIMETHYLSILYL)-, CYCLIC METHYLBORONATE | 3.20 | 738 | C14H31BO6Si2 | 362 | 54400-88-3 | WileyRegistry8e |
| 26.20 | MANNOONIC ACID, 2,3,5,6-TETRAKIS-O-(TRIMETHYLSILYL)-, LACTONE | 3.20 | 728 | C18H42O6Si4 | 466 | 55515-30-5 | WileyRegistry8e |

| Compound Structure | Hit Spectrum |
|---|---|

á-D-Galactopyranoside, methyl 2,3-bis-O-(trimethylsilyl)-, cyclic butylboronate
Formula C17H37BO6Si2, MW 404, CAS# 56211-10-0, Entry# 42381

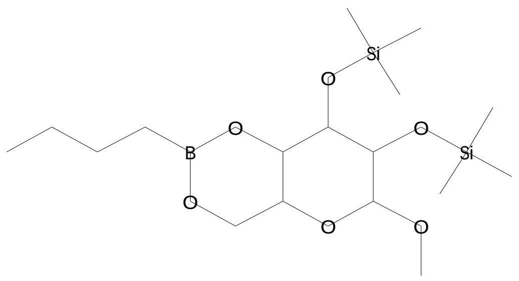

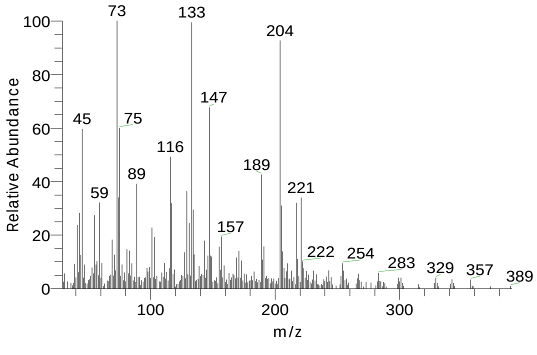


á-D-GALACTOPYRANOSIDE, METHYL 2,3-BIS-O-(TRIMETHYLSILYL)-, CYCLIC BUTYLBORONATE
Formula C17H37BO6Si2, MW 404, CAS# 56211-10-0, Entry# 257666

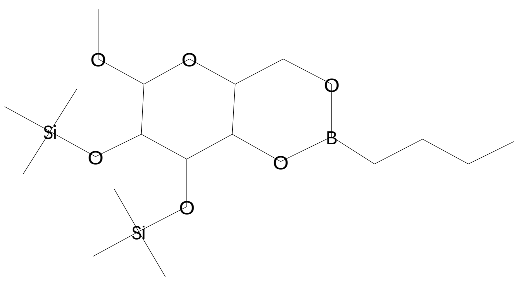

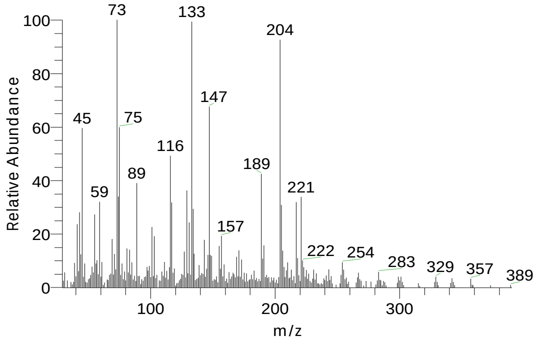

# *My GC-MS Report*

Compound Structure | Hit Spectrum

à-D-Galactopyranoside, methyl 2,3-bis-O-(trimethylsilyl)-, cyclic methylboronate
Formula C14H31BO6Si2, MW 362, CAS# 54400-88-3, Entry# 42380

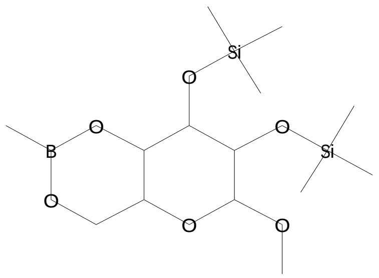


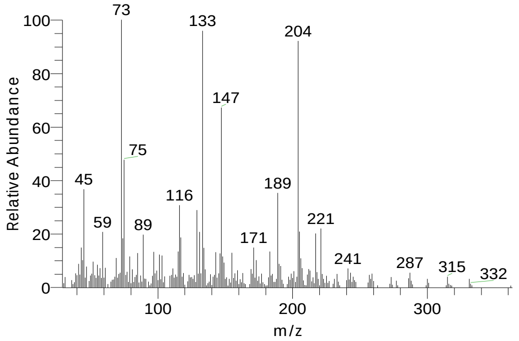


à-D-GALACTOPYRANOSIDE, METHYL 2,3-BIS-O-(TRIMETHYLSILYL)-, CYCLIC METHYLBORONATE
Formula C14H31BO6Si2, MW 362, CAS# 54400-88-3, Entry# 235852

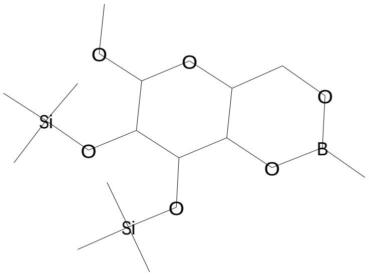


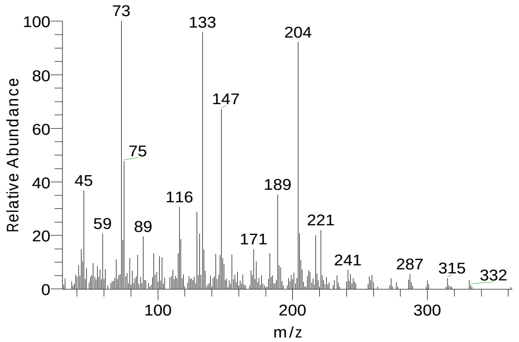


MANNOONIC ACID, 2,3,5,6-TETRAKIS-O-(TRIMETHYLSILYL)-, LACTONE
Formula C18H42O6Si4, MW 466, CAS# 55515-30-5, Entry# 278427
2,3,5,6-TETRA-O-TRIMETHYLSILYL-MANNONO-1,4-LACTONE

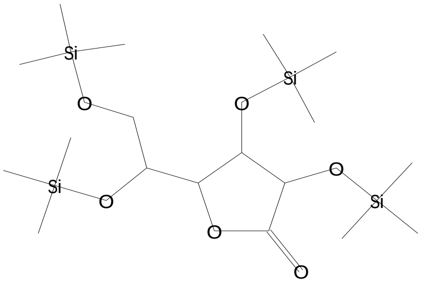


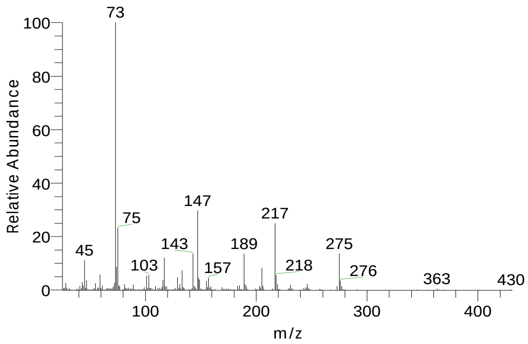


14008 #6681 RT: 26.40 AV: 1 NL: 8.37E6
T: + c EI Full ms [50.000-750.000]

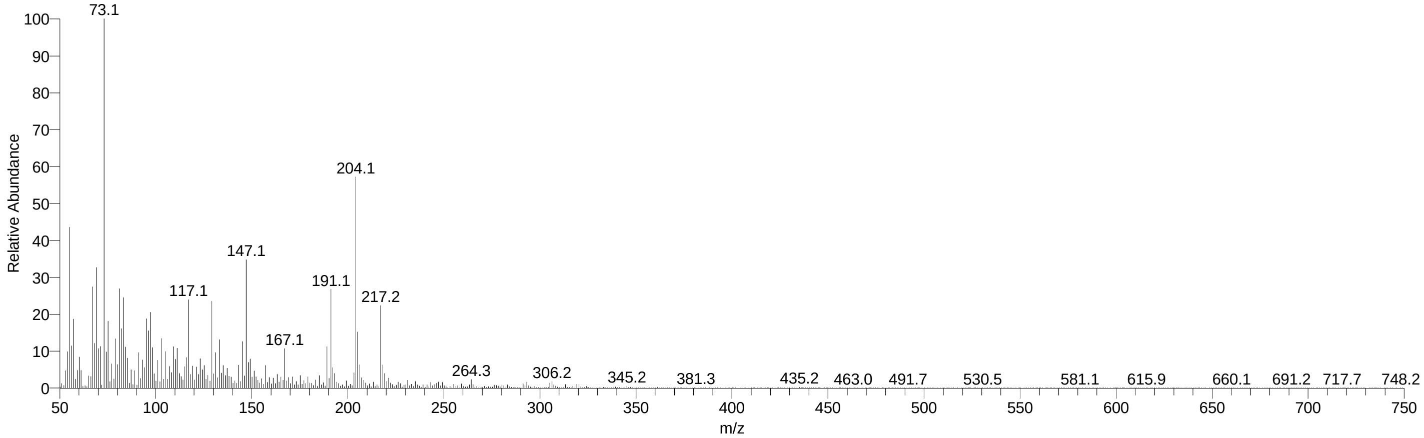


| RT | Compound Name | Area % | MF | Molecular Formula | Molecular Weight | Cas # | Library |
|---|---|---|---|---|---|---|---|
| 26.40 | á-D-Galactopyranoside, methyl 2,3-bis-O-(trimethylsilyl)-, cyclic butylboronate | 0.39 | 762 | C17H37BO6Si2 | 404 | 56211-10-0 | mainlib |

# *My GC-MS Report*

| RT | Compound Name | Area % | MF | Molecular Formula | Molecular Weight | Cas # | Library |
|---|---|---|---|---|---|---|---|
| 26.40 | á-D-GALACTOPYRANOSIDE, METHYL 2,3-BIS-O-(TRIMETHYLSILYL)-, CYCLIC BUTYLBORONATE | 0.39 | 762 | C17H37BO6Si2 | 404 | 56211-10-0 | WileyRegistry8e |
| 26.40 | á-D-Galactopyranoside, methyl 2,3-bis-O-(trimethylsilyl)-, cyclic methylboronate | 0.39 | 755 | C14H31BO6Si2 | 362 | 56211-08-6 | mainlib |
| 26.40 | á-D-GALACTOPYRANOSIDE, METHYL 2,3-BIS-O-(TRIMETHYLSILYL)-, CYCLIC METHYLBORONATE | 0.39 | 755 | C14H31BO6Si2 | 362 | 56211-08-6 | WileyRegistry8e |
| 26.40 | à-D-GALACTOPYRANOSIDE, METHYL 2,3-BIS-O-(TRIMETHYLSILYL)-, CYCLIC BUTYLBORONATE | 0.39 | 753 | C17H37BO6Si2 | 404 | 56211-11-1 | WileyRegistry8e |

Compound Structure | Hit Spectrum

á-D-Galactopyranoside, methyl 2,3-bis-O-(trimethylsilyl)-, cyclic butylboronate
Formula C17H37BO6Si2, MW 404, CAS# 56211-10-0, Entry# 42381

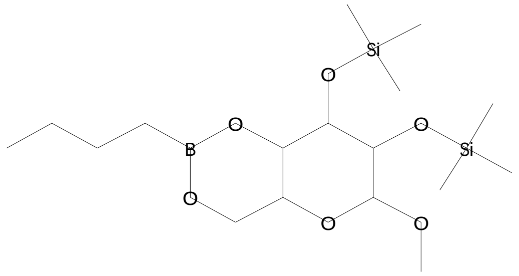


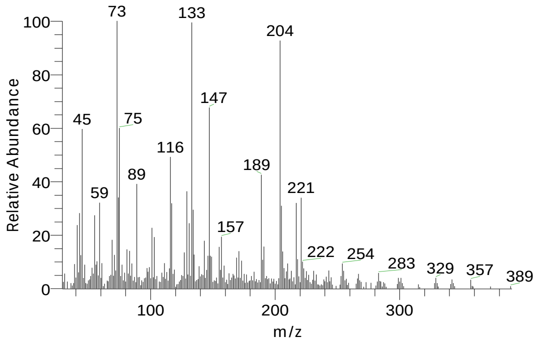


á-D-GALACTOPYRANOSIDE, METHYL 2,3-BIS-O-(TRIMETHYLSILYL)-, CYCLIC BUTYLBORONATE
Formula C17H37BO6Si2, MW 404, CAS# 56211-10-0, Entry# 257666

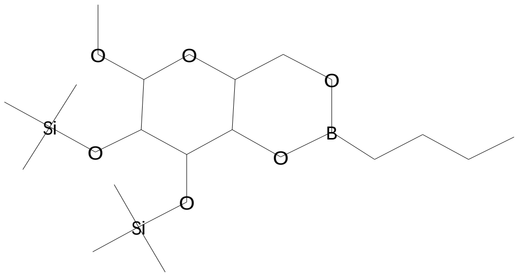


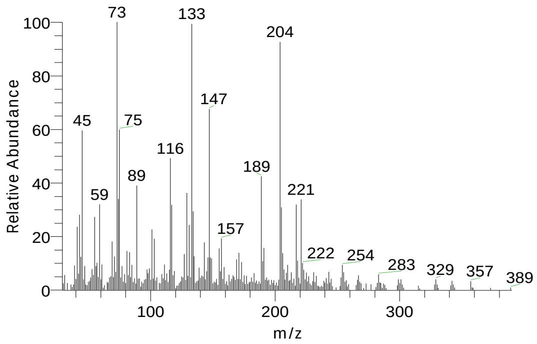


á-D-Galactopyranoside, methyl 2,3-bis-O-(trimethylsilyl)-, cyclic methylboronate
Formula C14H31BO6Si2, MW 362, CAS# 56211-08-6, Entry# 41509

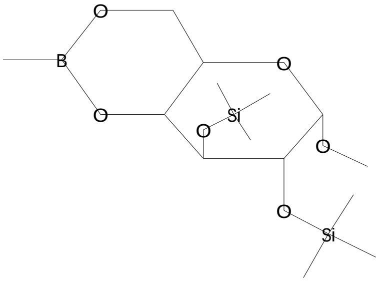


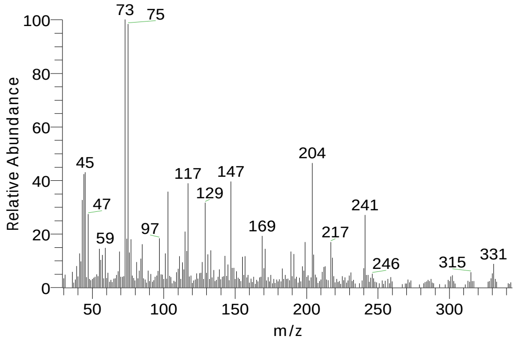

# My GC-MS Report

Compound Structure | Hit Spectrum

á-D-GALACTOPYRANOSIDE, METHYL 2,3-BIS-O-(TRIMETHYLSILYL)-, CYCLIC METHYLBORONATE
Formula C14H31BO6Si2, MW 362, CAS# 56211-08-6, Entry# 235854

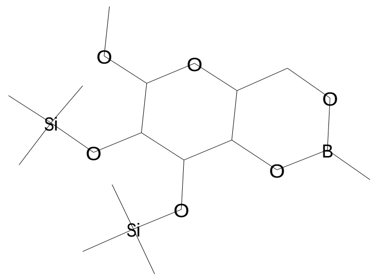


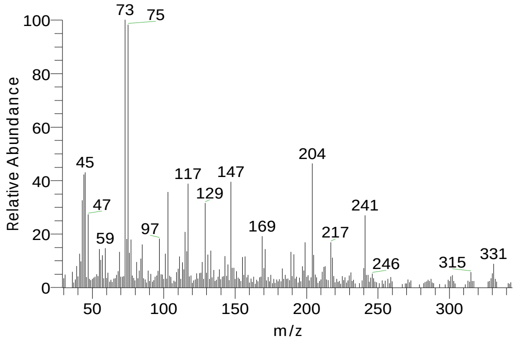


à-D-GALACTOPYRANOSIDE, METHYL 2,3-BIS-O-(TRIMETHYLSILYL)-, CYCLIC BUTYLBORONATE
Formula C17H37BO6Si2, MW 404, CAS# 56211-11-1, Entry# 257663

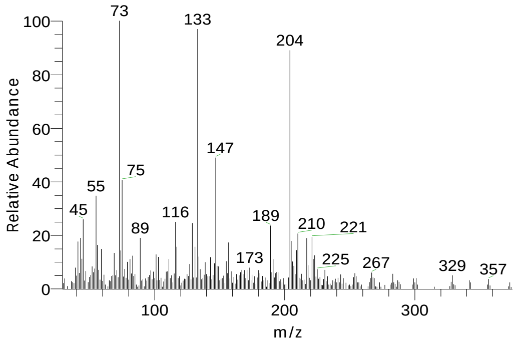


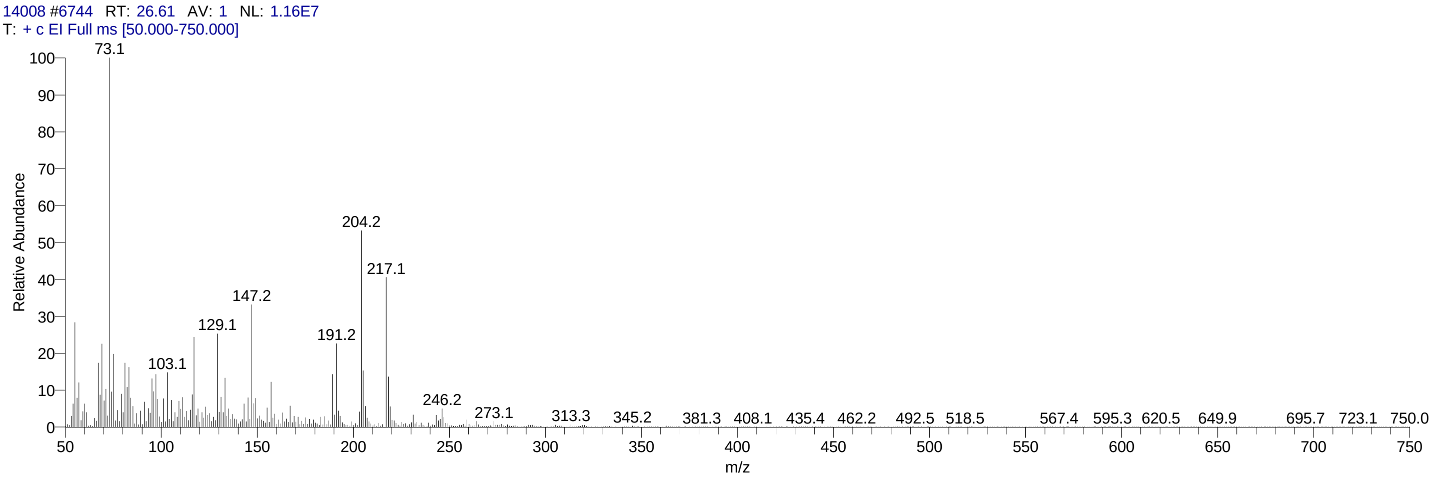


| RT | Compound Name | Area % | MF | Molecular Formula | Molecular Weight | Cas # | Library |
|---|---|---|---|---|---|---|---|
| 26.61 | à-D-Galactopyranose, 1,2,3-tris-O-(trimethylsilyl)-, cyclic methylboronate | 1.47 | 799 | C16H37BO6Si3 | 420 | 56196-95-3 | mainlib |
| 26.61 | à-D-GALACTOPYRANOSE, 1,2,3-TRIS-O-(TRIMETHYLSILYL)-, CYCLIC METHYLBORONATE | 1.47 | 799 | C16H37BO6Si3 | 420 | 56196-95-3 | WileyRegistry8e |
| 26.61 | á-D-Galactopyranoside, methyl 2,6-bis-O-(trimethylsilyl)-, cyclic methylboronate | 1.47 | 777 | C14H31BO6Si2 | 362 | 56211-06-4 | mainlib |
| 26.61 | á-D-GALACTOPYRANOSIDE, METHYL 2,6-BIS-O-(TRIMETHYLSILYL)-, CYCLIC METHYLBORONATE | 1.47 | 776 | C14H31BO6Si2 | 362 | 56211-06-4 | WileyRegistry8e |
| 26.61 | D-GLUCOSE, 6-O-à-D-GALACTOPYRANOSYL-, BIS-O-(TRIMETHYLSILYL) DERIV., CYCLIC TRIS(METHYLBORONATE) | 1.47 | 772 | C21H41B3O11Si2 | 558 | 72347-76-3 | WileyRegistry8e |

# My GC-MS Report

Compound Structure | Hit Spectrum

à-D-Galactopyranose, 1,2,3-tris-O-(trimethylsilyl)-, cyclic methylboronate
Formula C16H37BO6Si3, MW 420, CAS# 56196-95-3, Entry# 41501

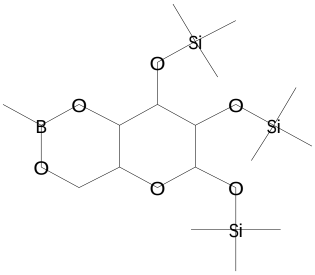

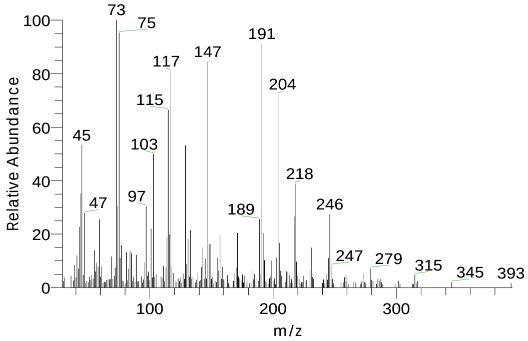


à-D-GALACTOPYRANOSE, 1,2,3-TRIS-O-(TRIMETHYLSILYL)-, CYCLIC METHYLBORONATE
Formula C16H37BO6Si3, MW 420, CAS# 56196-95-3, Entry# 264348

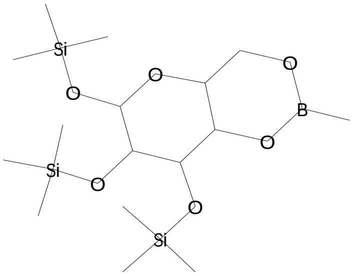

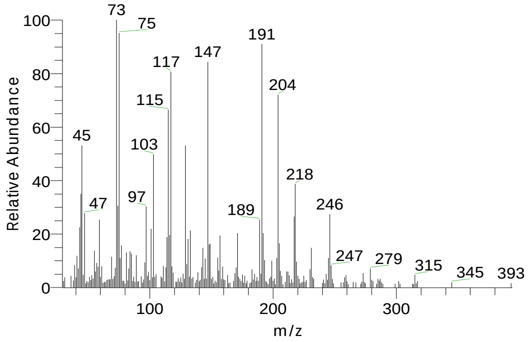


á-D-Galactopyranoside, methyl 2,6-bis-O-(trimethylsilyl)-, cyclic methylboronate
Formula C14H31BO6Si2, MW 362, CAS# 56211-06-4, Entry# 41379

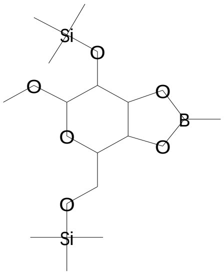

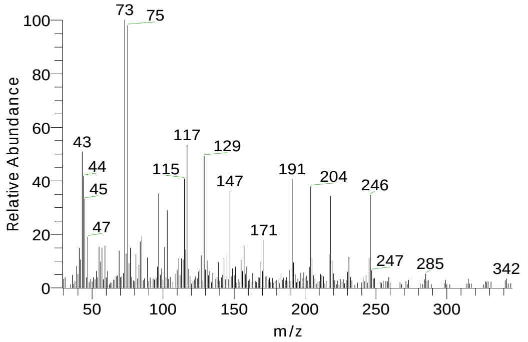


á-D-GALACTOPYRANOSIDE, METHYL 2,6-BIS-O-(TRIMETHYLSILYL)-, CYCLIC METHYLBORONATE
Formula C14H31BO6Si2, MW 362, CAS# 56211-06-4, Entry# 235850

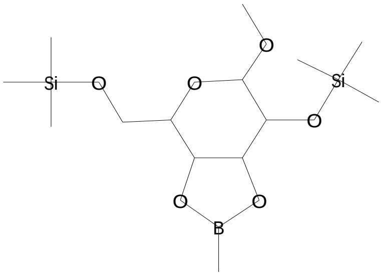

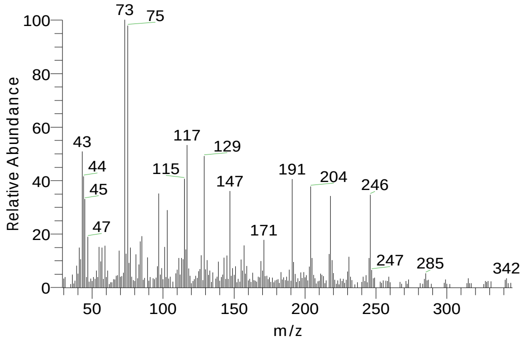

# My GC-MS Report

Compound Structure | Hit Spectrum

Formula C21H41B3O11Si2, MW 558, CAS# 72347-76-3, Entry# 293114

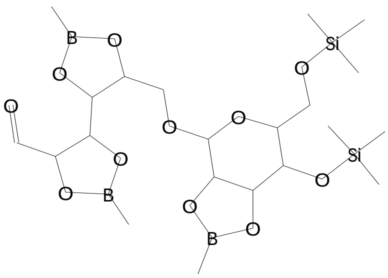

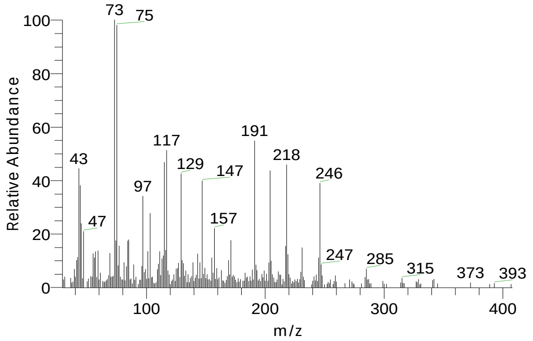


14008 #6802 RT: 26.81 AV: 1 NL: 2.00E7
T: + c EI Full ms [50.000-750.000]

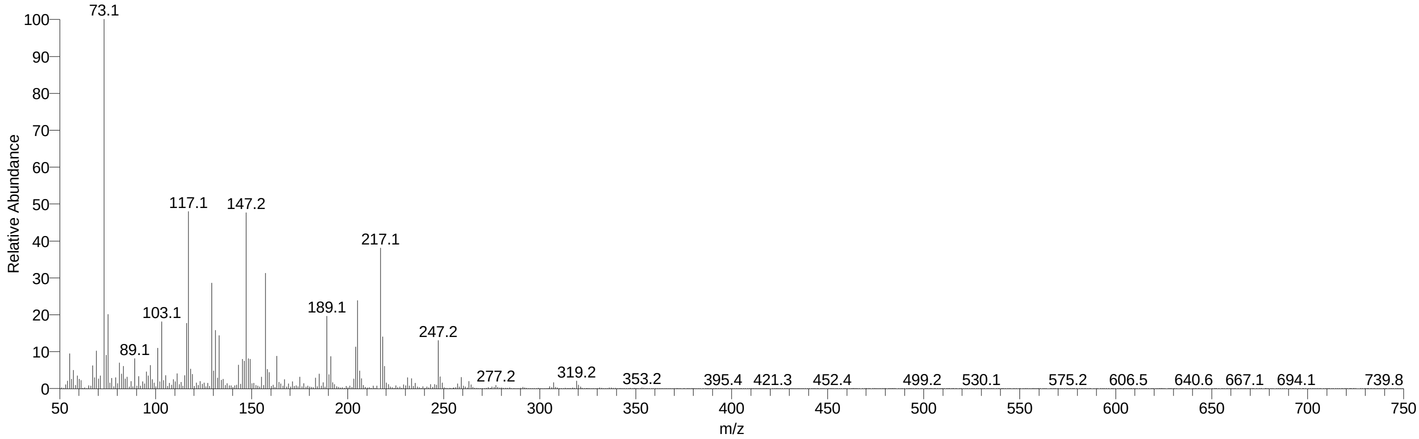


| RT | Compound Name | Area % | MF | Molecular Formula | Molecular Weight | Cas # | Library |
|---|---|---|---|---|---|---|---|
| 26.81 | 1,5-Anhydrohexitol, 4TMS derivative | 6.38 | 754 | C18H44O5Si4 | 452 | NA | mainlib |
| 26.81 | MANNOONIC ACID, 2,3,5,6-TETRAKIS-O-(TRIMETHYLSILYL)-, LACTONE | 6.38 | 755 | C18H42O6Si4 | 466 | 55515-30-5 | WileyRegistry8e |
| 26.81 | D-MANNONIC ACID, 2,3,5,6-TETRAKIS-O-(TRIMETHYLSILYL)-, ç-LACTONE | 6.38 | 755 | C18H42O6Si4 | 466 | 1986-18-1 | WileyRegistry8e |
| 26.81 | à-D-Galactopyranose, 1,2,3-tris-O-(trimethylsilyl)-, cyclic methylboronate | 6.38 | 751 | C16H37BO6Si3 | 420 | 56196-95-3 | mainlib |
| 26.81 | à-D-GALACTOPYRANOSE, 1,2,3-TRIS-O-(TRIMETHYLSILYL)-, CYCLIC METHYLBORONATE | 6.38 | 751 | C16H37BO6Si3 | 420 | 56196-95-3 | WileyRegistry8e |

Compound Structure | Hit Spectrum

1,5-Anhydrohexitol, 4TMS derivative
Formula C18H44O5Si4, MW 452, CAS# NA, Entry# 42675
1,5-Anhydro-D-sorbitol, tetrakis(trimethylsilyl) ether

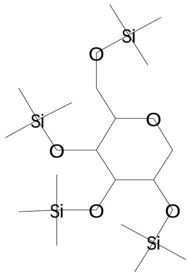

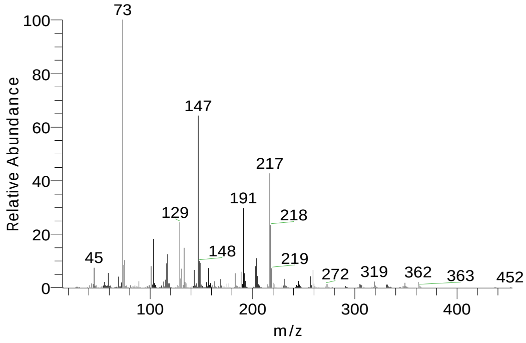

# My GC-MS Report

Compound Structure | Hit Spectrum

MANNOONIC ACID, 2,3,5,6-TETRAKIS-O-(TRIMETHYLSILYL)-, LACTONE
Formula C18H42O6Si4, MW 466, CAS# 55515-30-5, Entry# 278427
2,3,5,6-TETRA-O-TRIMETHYLSILYL-MANNONO-1,4-LACTONE

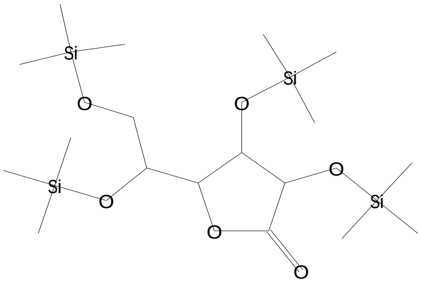

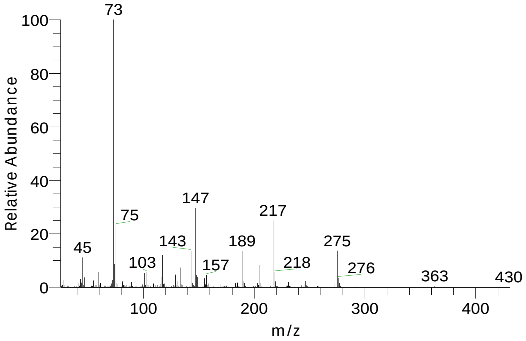


D-MANNONIC ACID, 2,3,5,6-TETRAKIS-O-(TRIMETHYLSILYL)-, ç-LACTONE
Formula C18H42O6Si4, MW 466, CAS# 1986-18-1, Entry# 392684

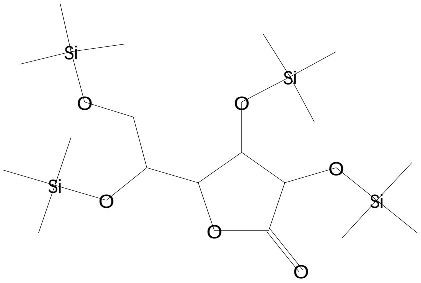

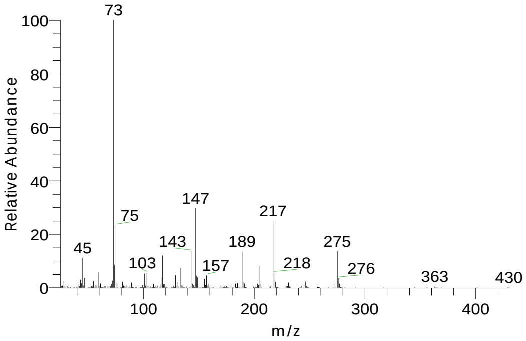


à-D-Galactopyranose, 1,2,3-tris-O-(trimethylsilyl)-, cyclic methylboronate
Formula C16H37BO6Si3, MW 420, CAS# 56196-95-3, Entry# 41501

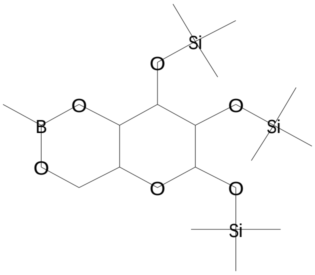

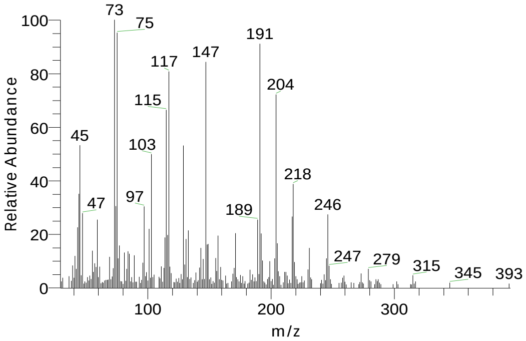


à-D-GALACTOPYRANOSE, 1,2,3-TRIS-O-(TRIMETHYLSILYL)-, CYCLIC METHYLBORONATE
Formula C16H37BO6Si3, MW 420, CAS# 56196-95-3, Entry# 264348

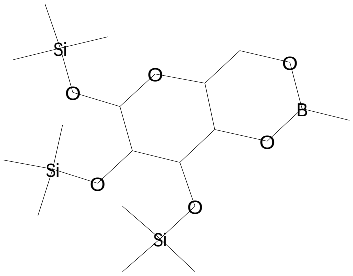

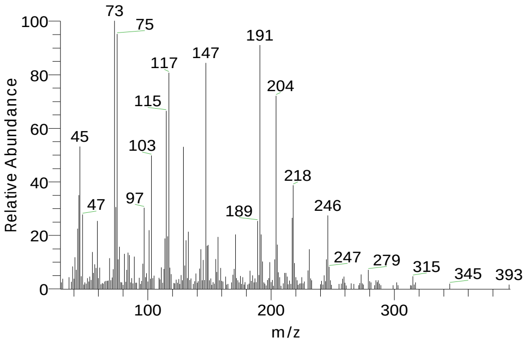

# *My GC-MS Report*

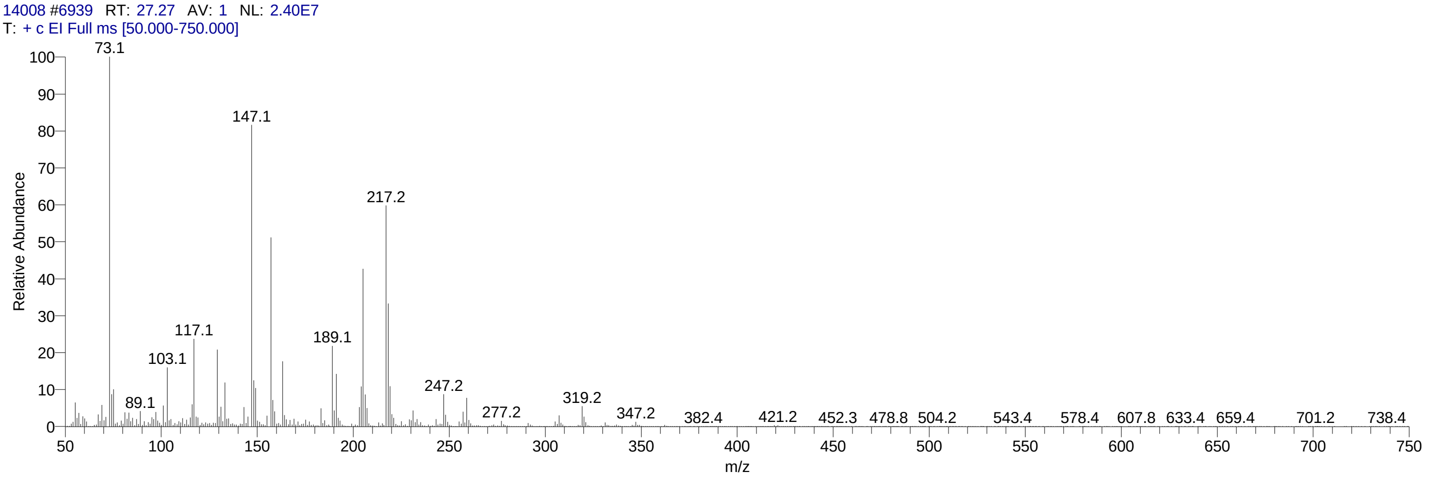


| RT | Compound Name | Area % | MF | Molecular Formula | Molecular Weight | Cas # | Library |
|---|---|---|---|---|---|---|---|
| 27.27 | 1,5-Anhydrohexitol, 4TMS derivative | 6.27 | 798 | C18H44O5Si4 | 452 | NA | mainlib |
| 27.27 | Dulcitol, 6TMS derivative | 6.27 | 759 | C24H62O6Si6 | 614 | 35-23-4 | mainlib |
| 27.27 | D-Sorbitol, 6TMS derivative | 6.27 | 752 | C24H62O6Si6 | 614 | NA | mainlib |
| 27.27 | BUTANAL, 2,3,4-TRIS[(TRIMETHYLSILYL)OXY]-, (R*,R*)- | 6.27 | 870 | C13H32O4Si3 | 336 | 56297-94-0 | WileyRegistry8e |
| 27.27 | L-Fucitol, 5TMS derivative | 6.27 | 752 | C21H54O5Si5 | 526 | NA | mainlib |

| Compound Structure | Hit Spectrum |
|---|---|

1,5-Anhydrohexitol, 4TMS derivative
Formula C18H44O5Si4, MW 452, CAS# NA, Entry# 42675
1,5-Anhydro-D-sorbitol, tetrakis(trimethylsilyl) ether

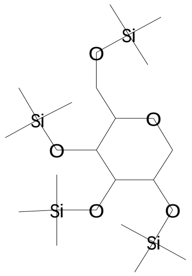

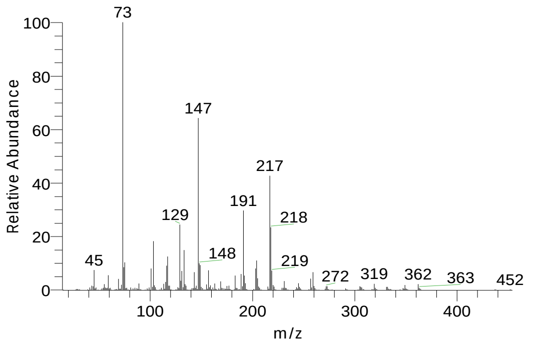


Dulcitol, 6TMS derivative
Formula C24H62O6Si6, MW 614, CAS# 35-23-4, Entry# 42684
Dulcitol, hexakis(trimethylsilyl) ether

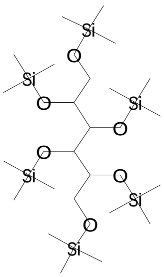

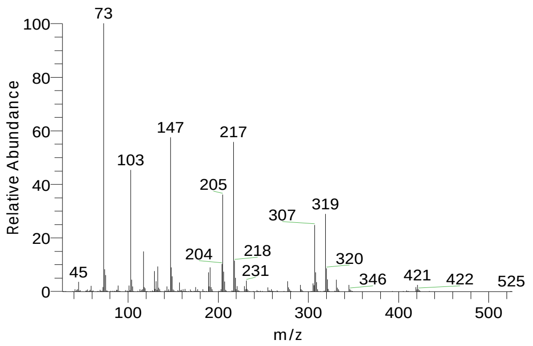

# My GC-MS Report

Compound Structure | Hit Spectrum

D-Sorbitol, 6TMS derivative
Formula C24H62O6Si6, MW 614, CAS# NA, Entry# 42749
D-Sorbitol, hexakis(trimethylsilyl) ether

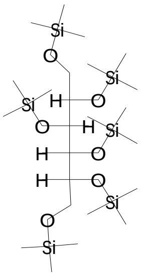


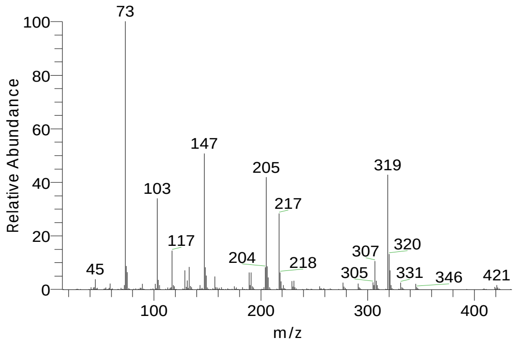


BUTANAL, 2,3,4-TRIS[(TRIMETHYLSILYL)OXY]-, (R*,R*)-
Formula C13H32O4Si3, MW 336, CAS# 56297-94-0, Entry# 218633
2,3,4-TRIS[(TRIMETHYLSILYL)OXY]BUTANAL #

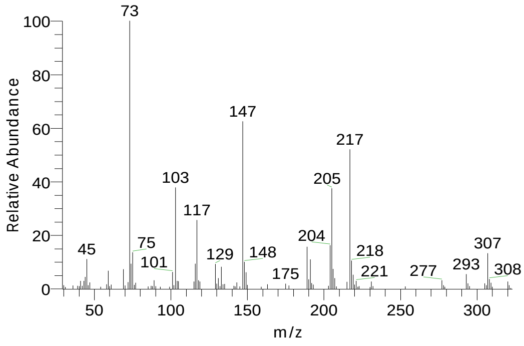


L-Fucitol, 5TMS derivative
Formula C21H54O5Si5, MW 526, CAS# NA, Entry# 42200
L-Fucitol, pentakis(trimethylsilyl) ether

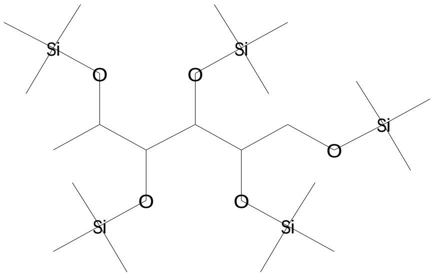


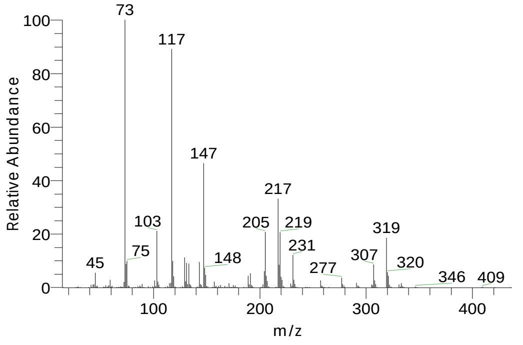


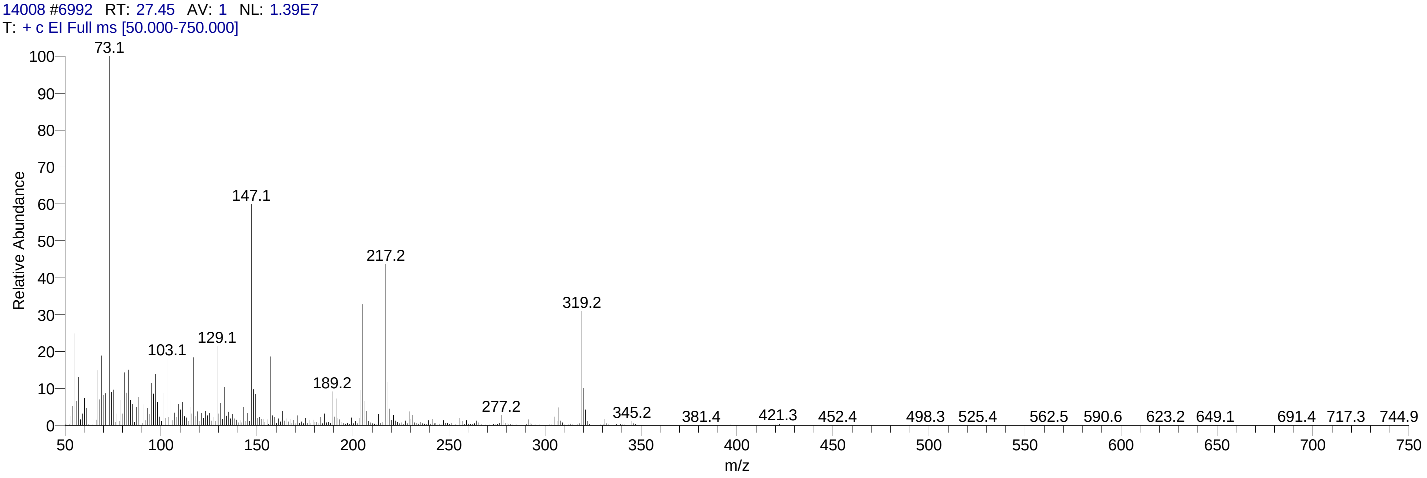


| RT | Compound Name | Area % | MF | Molecular Formula | Molecular Weight | Cas # | Library |
|---|---|---|---|---|---|---|---|
| 27.45 | D-Mannitol, 6TMS derivative | 0.99 | 821 | C24H62O6Si6 | 614 | 14317-07-8 | mainlib |
| 27.45 | D-Sorbitol, 6TMS derivative | 0.99 | 817 | C24H62O6Si6 | 614 | NA | mainlib |

# My GC-MS Report

| RT | Compound Name | Area % | MF | Molecular Formula | Molecular Weight | Cas # | Library |
|---|---|---|---|---|---|---|---|
| 27.45 | D-GLUCITOL, 1,2,3,4,5,6-HEXAKIS-O-(TRIMETHYLSILYL)- | 0.99 | 822 | C24H62O6Si6 | 614 | 14199-80-5 | WileyRegistry8e |
| 27.45 | Dulcitol, 6TMS derivative | 0.99 | 794 | C24H62O6Si6 | 614 | 35-23-4 | mainlib |
| 27.45 | D-GLUCITOL, 1,2,3,4,5,6-HEXAKIS-O-(TRIMETHYLSILYL)- | 0.99 | 829 | C24H62O6Si6 | 614 | 14199-80-5 | WileyRegistry8e |

| Compound Structure | Hit Spectrum |
|---|---|

D-Mannitol, 6TMS derivative
Formula C24H62O6Si6, MW 614, CAS# 14317-07-8, Entry# 42760
D-Mannitol, 1,2,3,4,5,6-hexakis-O-(trimethylsilyl)-

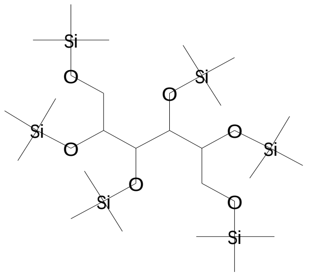


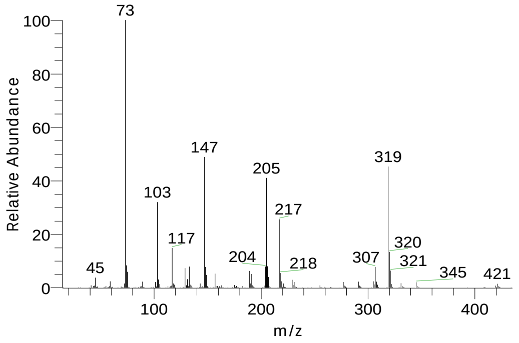


D-Sorbitol, 6TMS derivative
Formula C24H62O6Si6, MW 614, CAS# NA, Entry# 42749
D-Sorbitol, hexakis(trimethylsilyl) ether

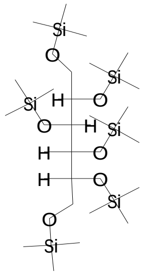


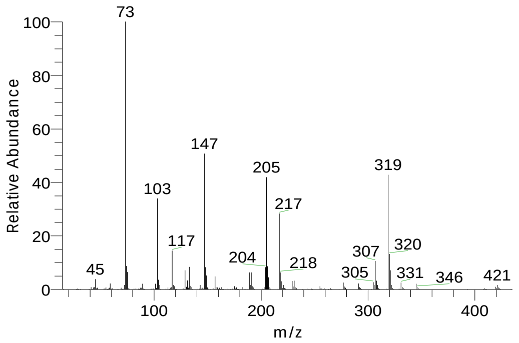


D-GLUCITOL, 1,2,3,4,5,6-HEXAKIS-O-(TRIMETHYLSILYL)-
Formula C24H62O6Si6, MW 614, CAS# 14199-80-5, Entry# 297756
1,2,3,4,5,6-HEXAKIS-O-(TRIMETHYLSILYL)HEXITOL #

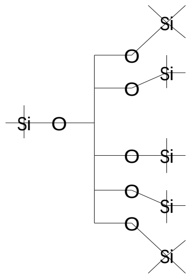


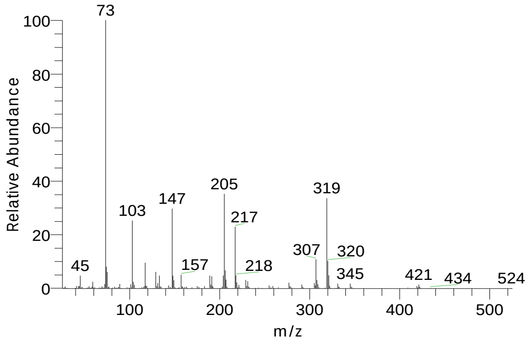


Dulcitol, 6TMS derivative
Formula C24H62O6Si6, MW 614, CAS# 35-23-4, Entry# 42684
Dulcitol, hexakis(trimethylsilyl) ether

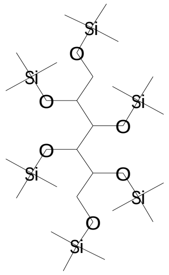


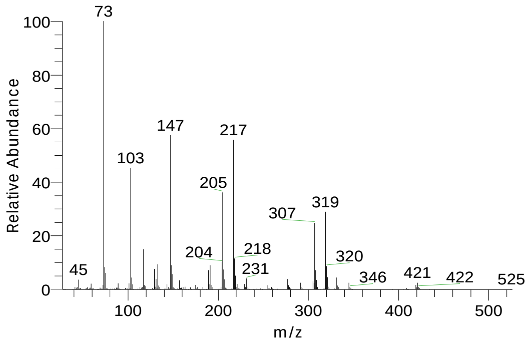

# My GC-MS Report

Compound Structure | Hit Spectrum

D-GLUCITOL, 1,2,3,4,5,6-HEXAKIS-O-(TRIMETHYLSILYL)-
Formula C24H62O6Si6, MW 614, CAS# 14199-80-5, Entry# 297755
1,2,3,4,5,6-HEXAKIS-O-(TRIMETHYLSILYL)HEXITOL #

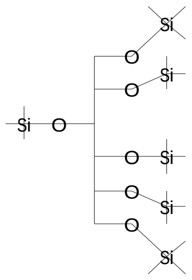

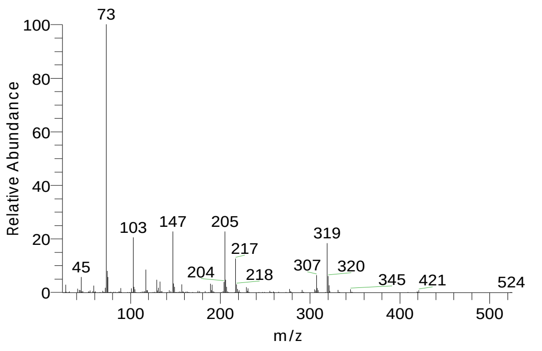

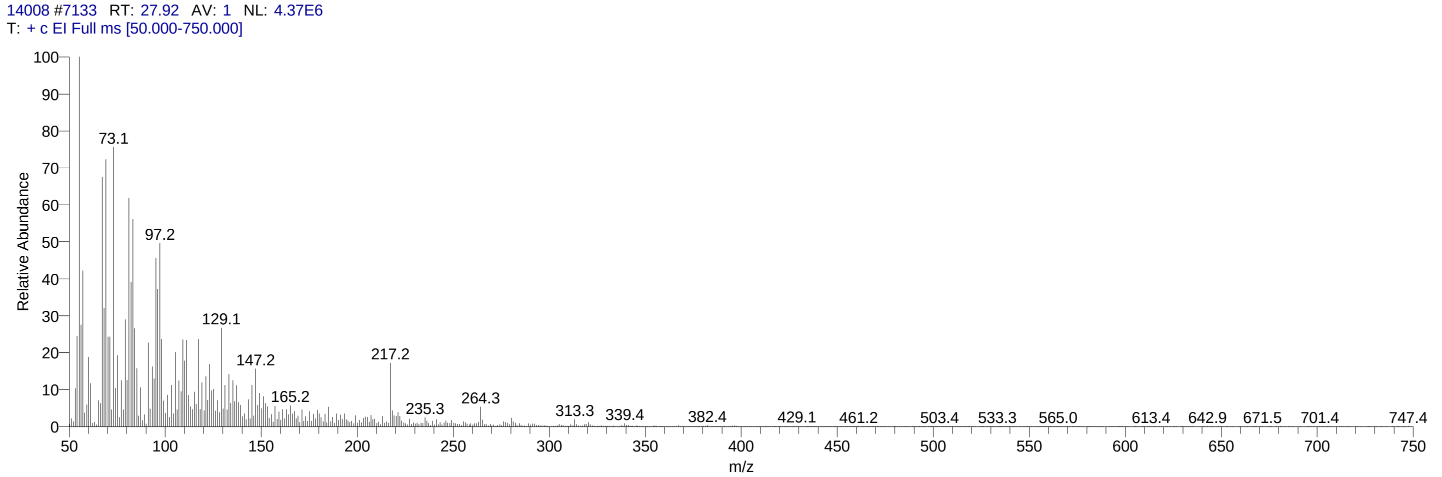

| RT | Compound Name | Area % | MF | Molecular Formula | Molecular Weight | Cas # | Library |
|---|---|---|---|---|---|---|---|
| 27.92 | 9-OCTADECENOIC ACID (Z)- | 0.37 | 823 | C18H34O2 | 282 | 112-80-1 | WileyRegistry8e |
| 27.92 | Oleic Acid | 0.37 | 796 | C18H34O2 | 282 | 112-80-1 | replib |
| 27.92 | 2-HYDROXY-3-[(9E)-9-OCTADECENOYLOXY]PROPYL (9E)-9-OCTADECENOATE # | 0.37 | 788 | C39H72O5 | 620 | 2465-32-9 | WileyRegistry8e |
| 27.92 | HEXADECADIENOIC ACID, METHYL ESTER | 0.37 | 799 | C17H30O2 | 266 | 29961-54-4 | WileyRegistry8e |
| 27.92 | cis-13-Eicosenoic acid | 0.37 | 791 | C20H38O2 | 310 | 17735-94-3 | mainlib |

Compound Structure | Hit Spectrum

9-OCTADECENOIC ACID (Z)-
Formula C18H34O2, MW 282, CAS# 112-80-1, Entry# 172910
OCTADEC-9-ENOIC ACID

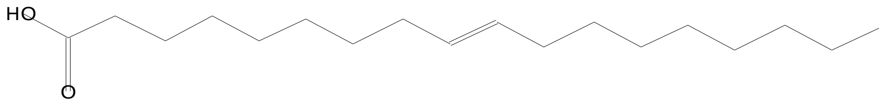

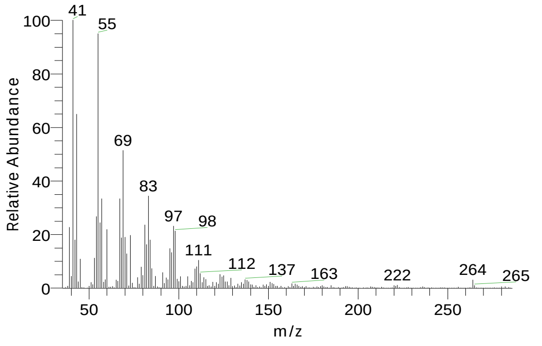

# My GC-MS Report

Compound Structure | Hit Spectrum

Oleic Acid
Formula C18H34O2, MW 282, CAS# 112-80-1, Entry# 4977
9-Octadecenoic acid (Z)-

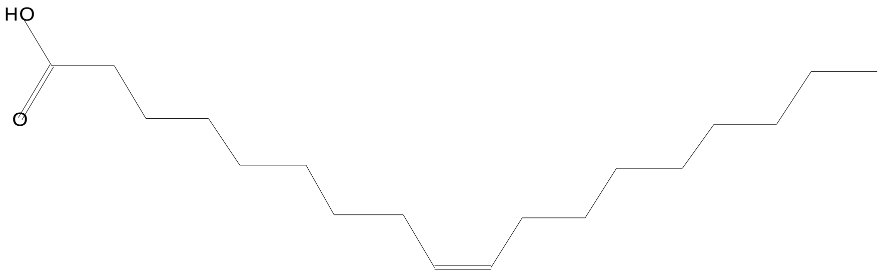


SI 783, RSI 796, replib, Entry# 4977, CAS# 112-80-1, Oleic Acid

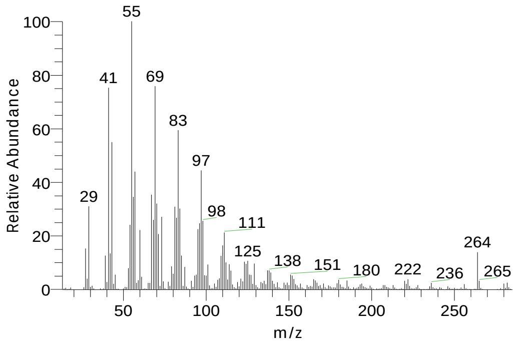


2-HYDROXY-3-[(9E)-9-OCTADECENOYLOXY]PROPYL (9E)-9-OCTADECENOATE #
Formula C39H72O5, MW 620, CAS# 2465-32-9, Entry# 298152
(Z,Z)-1,3-DIOCTADECENOYL GLYCEROL

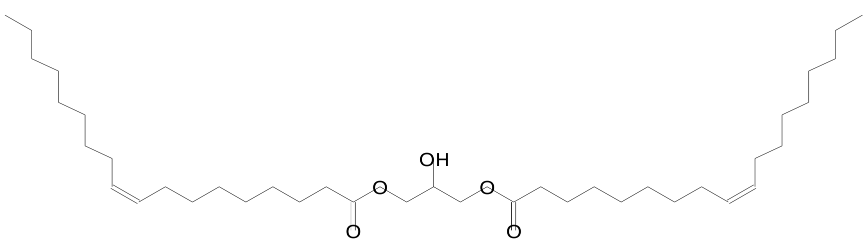


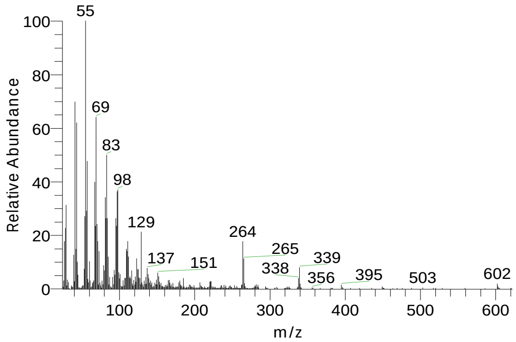


HEXADECADIENOIC ACID, METHYL ESTER
Formula C17H30O2, MW 266, CAS# 29961-54-4, Entry# 157129
METHYL HEXADECADIENOATE

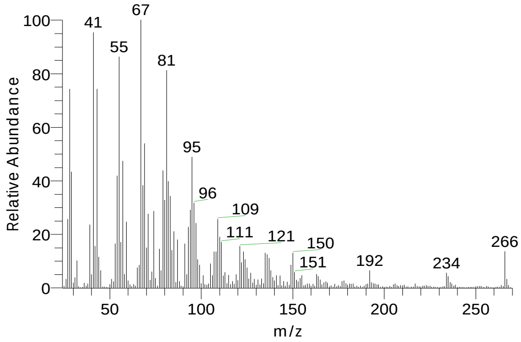


cis-13-Eicosenoic acid
Formula C20H38O2, MW 310, CAS# 17735-94-3, Entry# 20259
$:28URXZXNYJPAJJOQ-FPLPWBNLSA-N

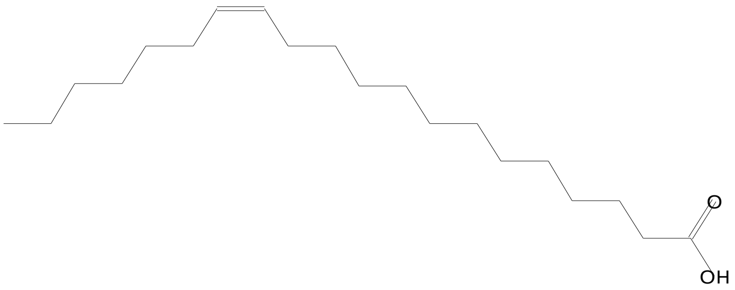


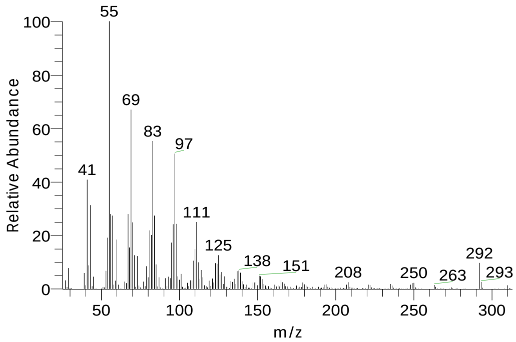

# My GC-MS Report

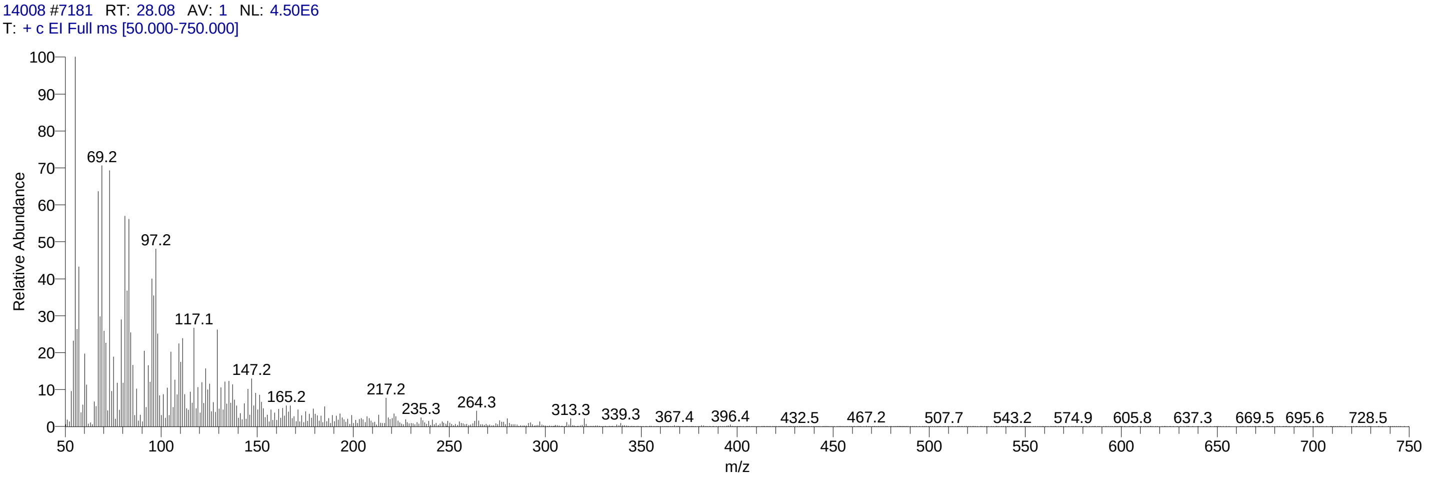


| RT | Compound Name | Area % | MF | Molecular Formula | Molecular Weight | Cas # | Library |
|---|---|---|---|---|---|---|---|
| 28.08 | 9-OCTADECENOIC ACID (Z)- | 0.62 | 833 | C18H34O2 | 282 | 112-80-1 | WileyRegistry8e |
| 28.08 | cis-13-Eicosenoic acid | 0.62 | 807 | C20H38O2 | 310 | 17735-94-3 | mainlib |
| 28.08 | 2-HYDROXY-3-[(9E)-9-OCTADECENOYLOXY]PROPYL (9E)-9-OCTADECENOATE # | 0.62 | 794 | C39H72O5 | 620 | 2465-32-9 | WileyRegistry8e |
| 28.08 | Oleic Acid | 0.62 | 802 | C18H34O2 | 282 | 112-80-1 | replib |
| 28.08 | HEXADECADIENOIC ACID, METHYL ESTER | 0.62 | 801 | C17H30O2 | 266 | 29961-54-4 | WileyRegistry8e |

Compound Structure | Hit Spectrum

9-OCTADECENOIC ACID (Z)-
Formula C18H34O2, MW 282, CAS# 112-80-1, Entry# 172910
OCTADEC-9-ENOIC ACID

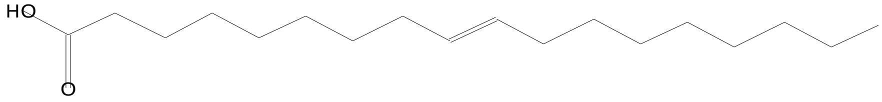


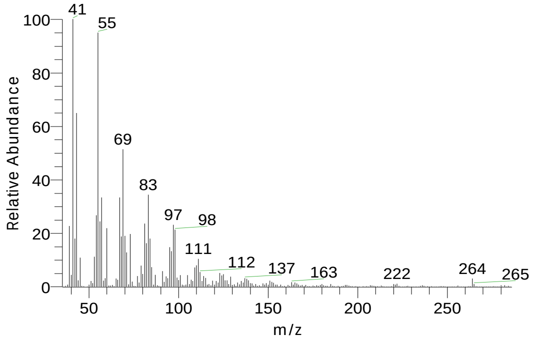


cis-13-Eicosenoic acid
Formula C20H38O2, MW 310, CAS# 17735-94-3, Entry# 20259
$:28URXZXNYJPAJJOQ-FPLPWBNLSA-N

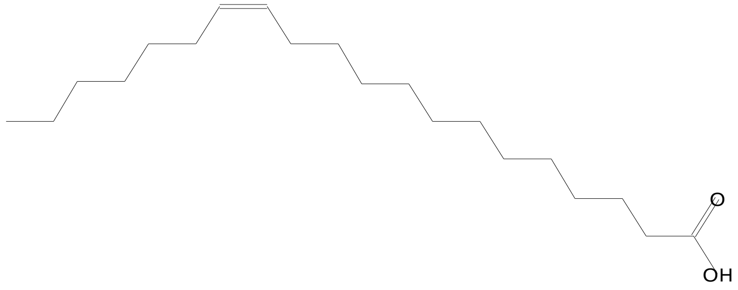


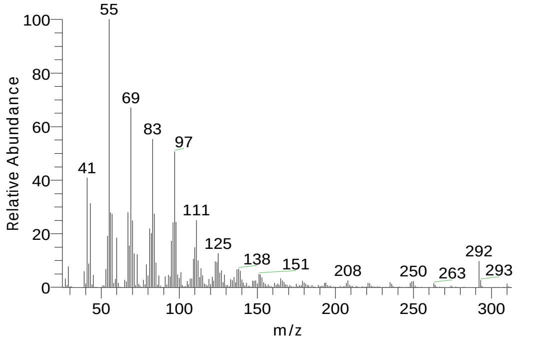

# *My GC-MS Report*

Compound Structure | Hit Spectrum

2-HYDROXY-3-[(9E)-9-OCTADECENOYLOXY]PROPYL (9E)-9-OCTADECENOATE #
Formula C39H72O5, MW 620, CAS# 2465-32-9, Entry# 298152
(Z,Z)-1,3-DIOCTADECENOYL GLYCEROL

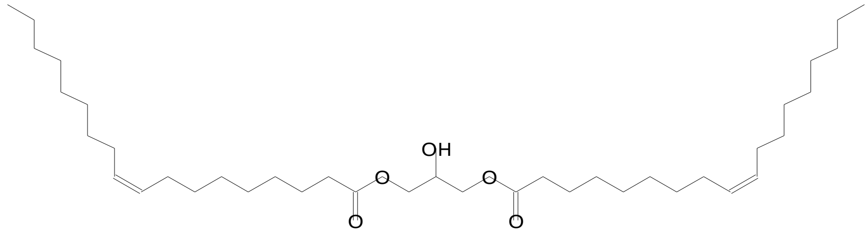


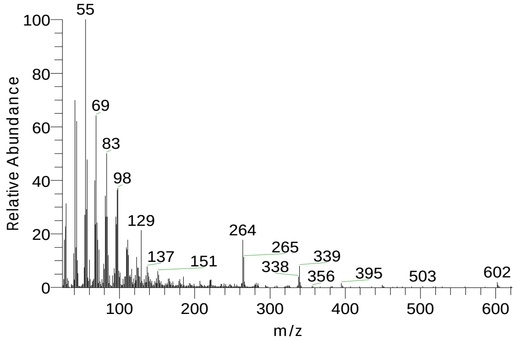


Oleic Acid
Formula C18H34O2, MW 282, CAS# 112-80-1, Entry# 4977
9-Octadecenoic acid (Z)-

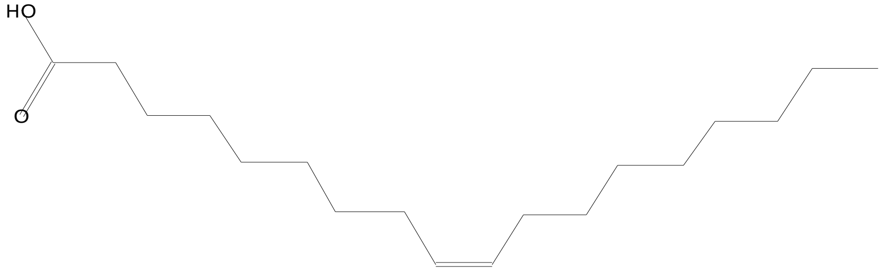


SI 785, RSI 802, replib, Entry# 4977, CAS# 112-80-1, Oleic Acid

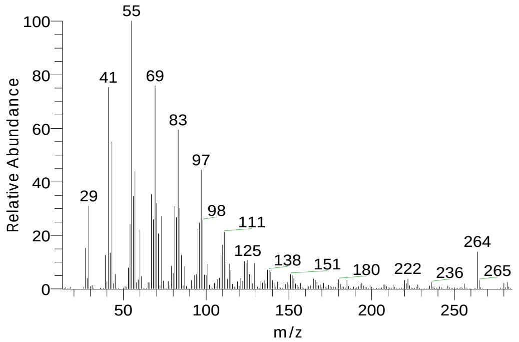


HEXADECADIENOIC ACID, METHYL ESTER
Formula C17H30O2, MW 266, CAS# 29961-54-4, Entry# 157129
METHYL HEXADECADIENOATE

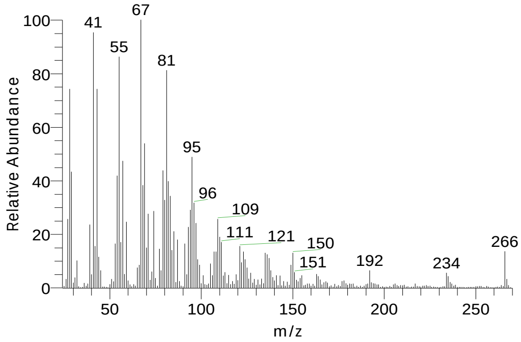


14008 #7261 RT: 28.35 AV: 1 NL: 7.24E6
T: + c EI Full ms [50.000-750.000]

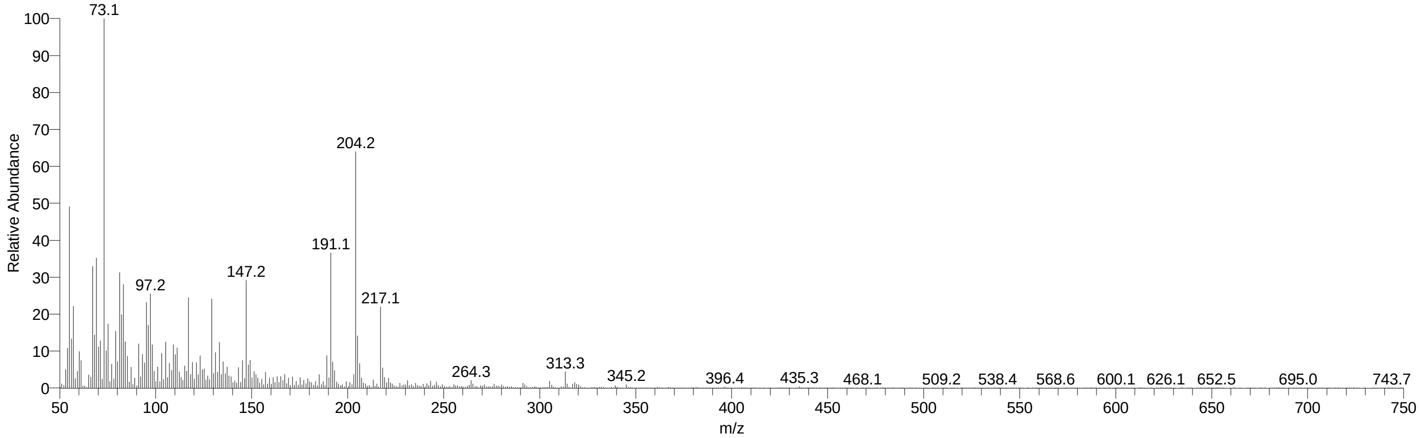


| RT | Compound Name | Area % | MF | Molecular Formula | Molecular Weight | Cas # | Library |
|---|---|---|---|---|---|---|---|
| 28.35 | á-D-Galactopyranoside, methyl 2,3-bis-O-(trimethylsilyl)-, cyclic butylboronate | 0.56 | 756 | C17H37BO6Si2 | 404 | 56211-10-0 | mainlib |

# *My GC-MS Report*

| RT | Compound Name | Area % | MF | Molecular Formula | Molecular Weight | Cas # | Library |
|---|---|---|---|---|---|---|---|
| 28.35 | á-D-GALACTOPYRANOSIDE, METHYL 2,3-BIS-O-(TRIMETHYLSILYL)-, CYCLIC BUTYLBORONATE | 0.56 | 756 | C17H37BO6Si2 | 404 | 56211-10-0 | WileyRegistry8e |
| 28.35 | à-D-Galactopyranose, 1,2,3-tris-O-(trimethylsilyl)-, cyclic methylboronate | 0.56 | 756 | C16H37BO6Si3 | 420 | 56196-95-3 | mainlib |
| 28.35 | à-D-GALACTOPYRANOSE, 1,2,3-TRIS-O-(TRIMETHYLSILYL)-, CYCLIC METHYLBORONATE | 0.56 | 756 | C16H37BO6Si3 | 420 | 56196-95-3 | WileyRegistry8e |
| 28.35 | à-D-GALACTOPYRANOSIDE, METHYL 2,3-BIS-O-(TRIMETHYLSILYL)-, CYCLIC BUTYLBORONATE | 0.56 | 745 | C17H37BO6Si2 | 404 | 56211-11-1 | WileyRegistry8e |

| Compound Structure | Hit Spectrum |
|---|---|

á-D-Galactopyranoside, methyl 2,3-bis-O-(trimethylsilyl)-, cyclic butylboronate
Formula C17H37BO6Si2, MW 404, CAS# 56211-10-0, Entry# 42381

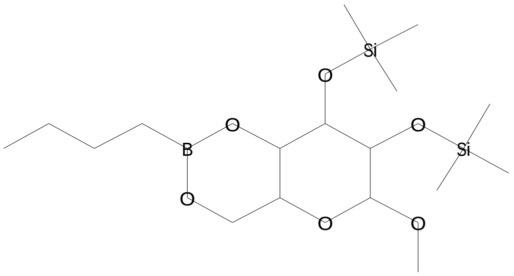

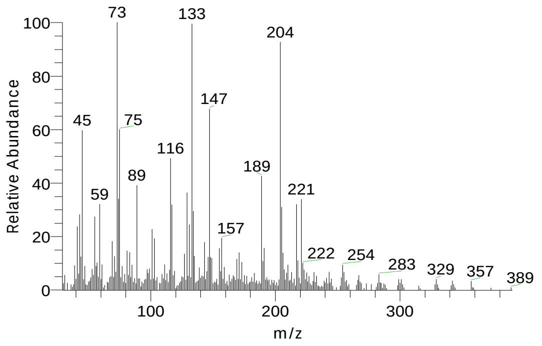


á-D-GALACTOPYRANOSIDE, METHYL 2,3-BIS-O-(TRIMETHYLSILYL)-, CYCLIC BUTYLBORONATE
Formula C17H37BO6Si2, MW 404, CAS# 56211-10-0, Entry# 257666

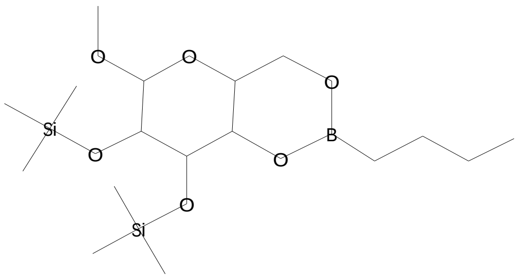

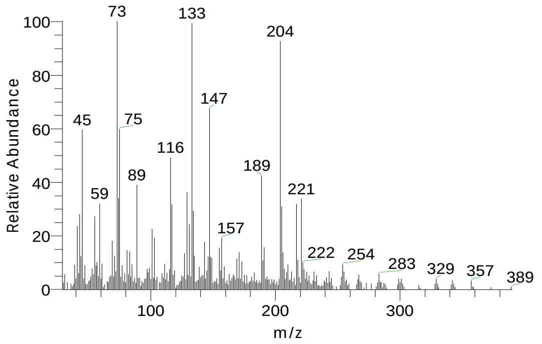


à-D-Galactopyranose, 1,2,3-tris-O-(trimethylsilyl)-, cyclic methylboronate
Formula C16H37BO6Si3, MW 420, CAS# 56196-95-3, Entry# 41501

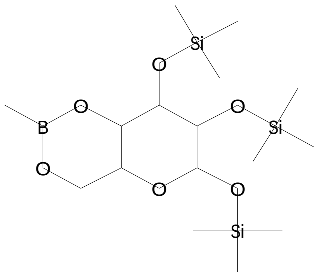

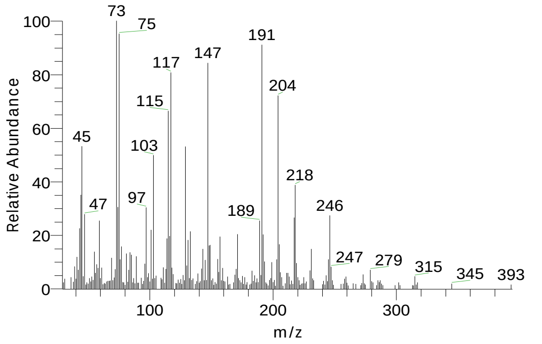

# *My GC-MS Report*

Compound Structure | Hit Spectrum

à-D-GALACTOPYRANOSE, 1,2,3-TRIS-O-(TRIMETHYLSILYL)-, CYCLIC METHYLBORONATE
Formula C16H37BO6Si3, MW 420, CAS# 56196-95-3, Entry# 264348

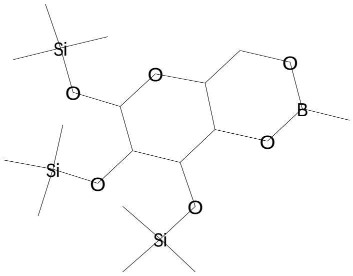


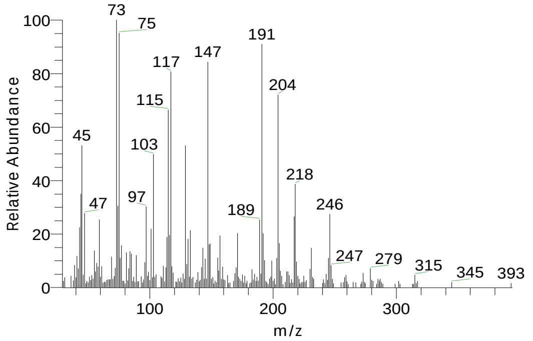


à-D-GALACTOPYRANOSIDE, METHYL 2,3-BIS-O-(TRIMETHYLSILYL)-, CYCLIC BUTYLBORONATE
Formula C17H37BO6Si2, MW 404, CAS# 56211-11-1, Entry# 257663

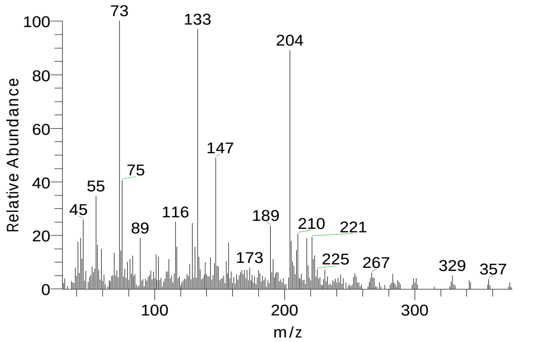


14008 #7326 RT: 28.57 AV: 1 NL: 9.92E6
T: + c EI Full ms [50.000-750.000]

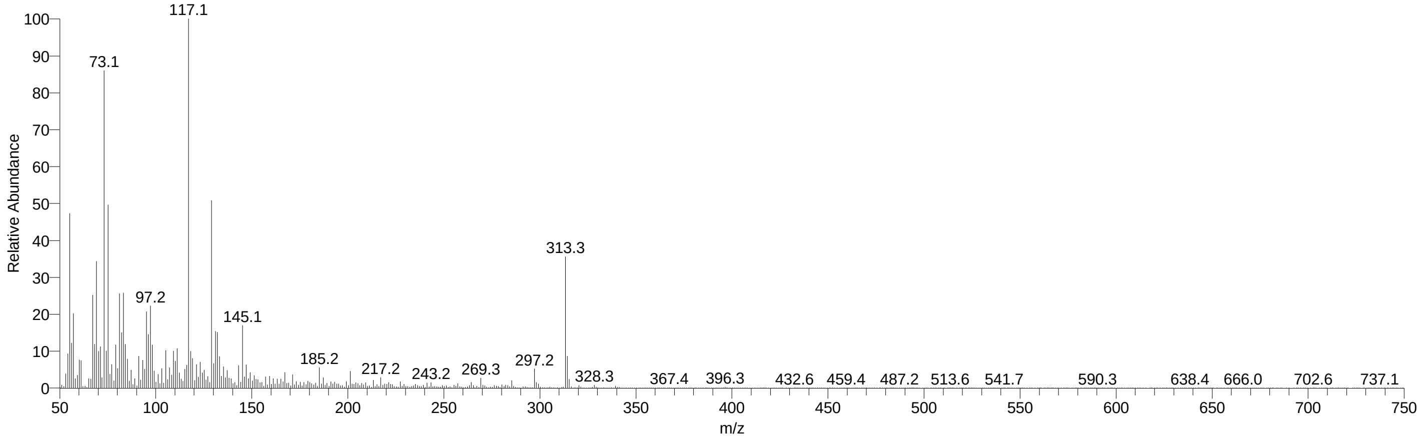


| RT | Compound Name | Area % | MF | Molecular Formula | Molecular Weight | Cas # | Library |
|---|---|---|---|---|---|---|---|
| 28.57 | Palmitic Acid, TMS derivative | 2.45 | 785 | C19H40O2Si | 328 | 55520-89-3 | replib |
| 28.57 | HEXADECANOIC ACID, TRIMETHYLSILYL ESTER | 2.45 | 785 | C19H40O2Si | 328 | 55520-89-3 | WileyRegistry8e |
| 28.57 | HEXADECANOIC ACID, TRIMETHYLSILYL ESTER | 2.45 | 781 | C19H40O2Si | 328 | 55520-89-3 | WileyRegistry8e |
| 28.57 | 13-Methyltetradec-9-enoic acid, TMS derivative | 2.45 | 783 | C18H36O2Si | 312 | NA | mainlib |
| 28.57 | Palmitic Acid, TMS derivative | 2.45 | 756 | C19H40O2Si | 328 | 55520-89-3 | mainlib |

# *My GC-MS Report*

Compound Structure | Hit Spectrum

Palmitic Acid, TMS derivative
Formula C19H40O2Si, MW 328, CAS# 55520-89-3, Entry# 9711
Hexadecanoic acid, trimethylsilyl ester

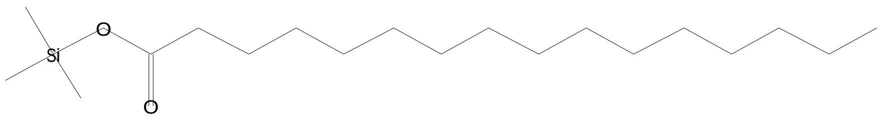


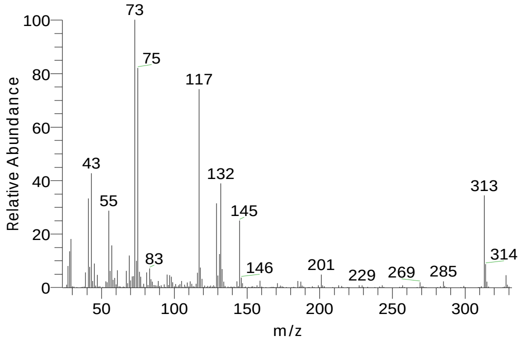


HEXADECANOIC ACID, TRIMETHYLSILYL ESTER
Formula C19H40O2Si, MW 328, CAS# 55520-89-3, Entry# 390490
TRIMETHYLSILYL PALMITATE #

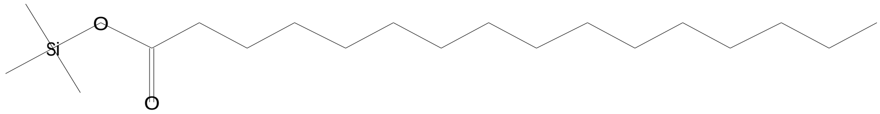


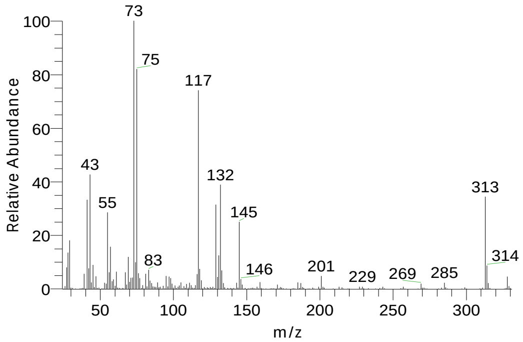


HEXADECANOIC ACID, TRIMETHYLSILYL ESTER
Formula C19H40O2Si, MW 328, CAS# 55520-89-3, Entry# 213028
TRIMETHYLSILYL PALMITATE #

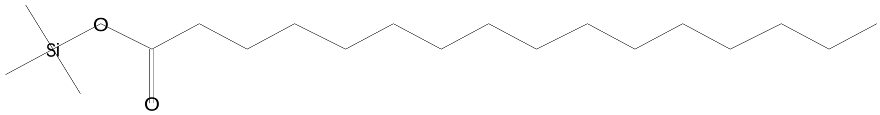


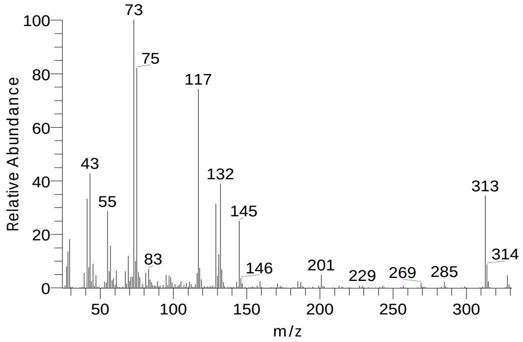


13-Methyltetradec-9-enoic acid, TMS derivative
Formula C18H36O2Si, MW 312, CAS# NA, Entry# 41374
13-methyltetradec-9-enoic acid trimethylsilyl ester

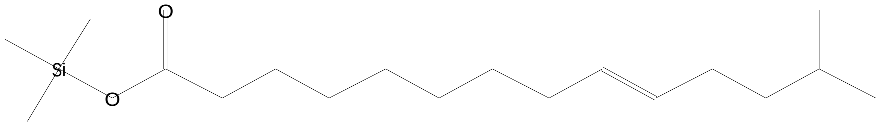


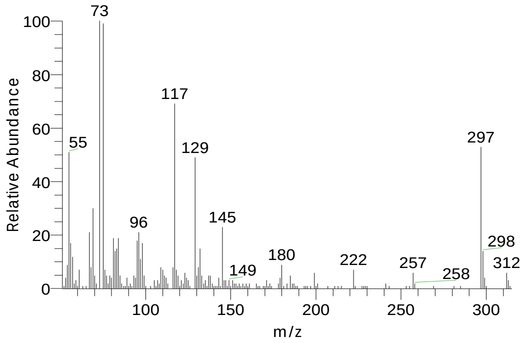

# *My GC-MS Report*

Compound Structure | Hit Spectrum

Palmitic Acid, TMS derivative
Formula C19H40O2Si, MW 328, CAS# 55520-89-3, Entry# 97113
Hexadecanoic acid, trimethylsilyl ester

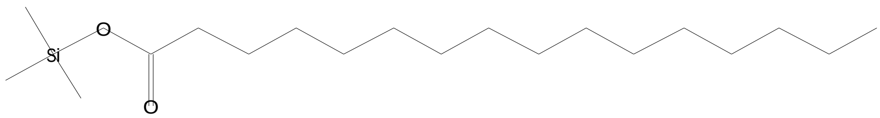


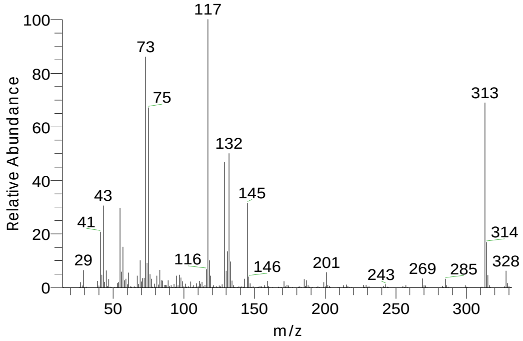


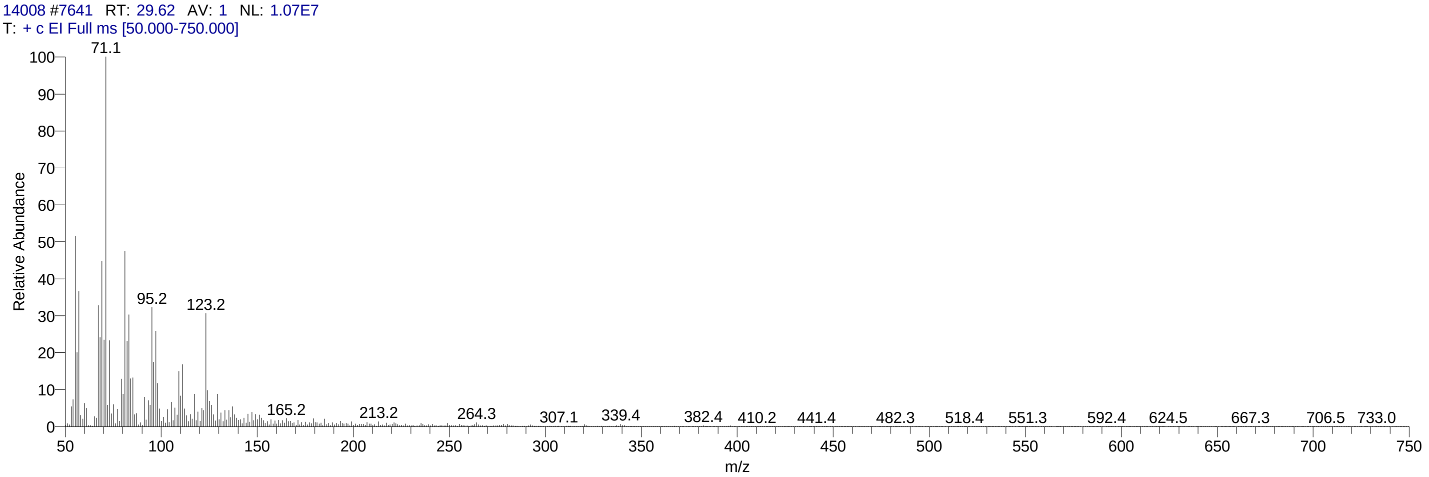


| RT | Compound Name | Area % | MF | Molecular Formula | Molecular Weight | Cas # | Library |
|---|---|---|---|---|---|---|---|
| 29.62 | Z-(13,14-Epoxy)tetradec-11-en-1-ol acetate | 2.98 | 806 | C16H28O3 | 268 | NA | mainlib |
| 29.62 | Ethanol, 2-(9-octadecenyloxy)-, (Z)- | 2.98 | 782 | C20H40O2 | 312 | 5353-25-3 | mainlib |
| 29.62 | ETHANOL, 2-(9-OCTADECENYLOXY)-, (Z)- | 2.98 | 781 | C20H40O2 | 312 | 5353-25-3 | WileyRegistry8e |
| 29.62 | 12-Methyl-E,E-2,13-octadecadien-1-ol | 2.98 | 813 | C19H36O | 280 | NA | mainlib |
| 29.62 | 17-Octadecynoic acid | 2.98 | 782 | C18H32O2 | 280 | 34450-18-5 | mainlib |

Compound Structure | Hit Spectrum

Z-(13,14-Epoxy)tetradec-11-en-1-ol acetate
Formula C16H28O3, MW 268, CAS# NA, Entry# 10459
(11Z)-12-(2-Oxiranyl)-11-dodecenyl acetate #

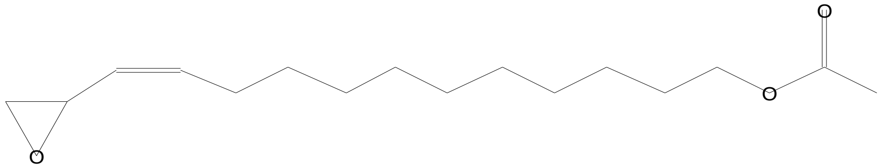


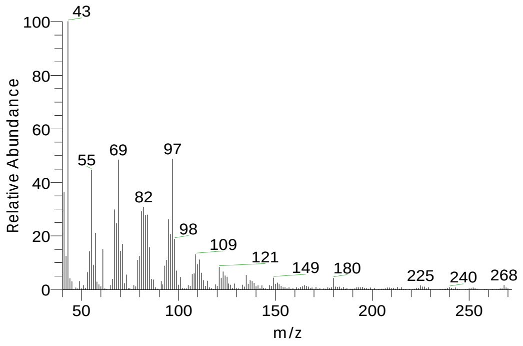

# My GC-MS Report

Compound Structure | Hit Spectrum

Ethanol, 2-(9-octadecenyloxy)-, (Z)-
Formula C20H40O2, MW 312, CAS# 5353-25-3, Entry# 20581
2-cis-9-Octadecenyloxyethanol

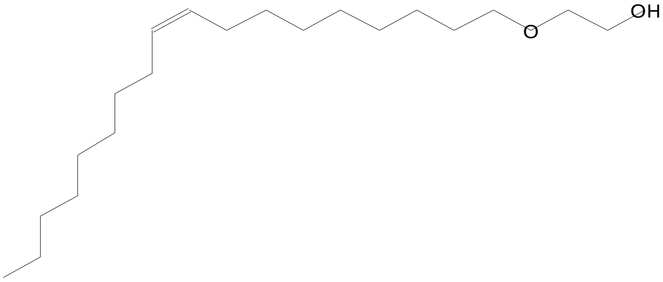


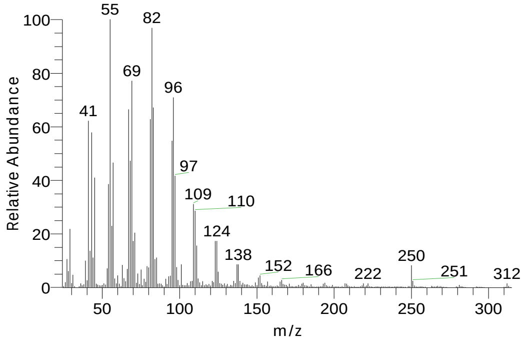


ETHANOL, 2-(9-OCTADECENYLOXY)-, (Z)-
Formula C20H40O2, MW 312, CAS# 5353-25-3, Entry# 200393
2-[(9Z)-9-OCTADECENYLOXY]ETHANOL #

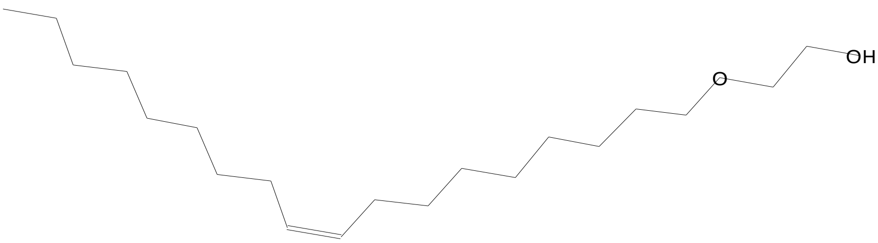


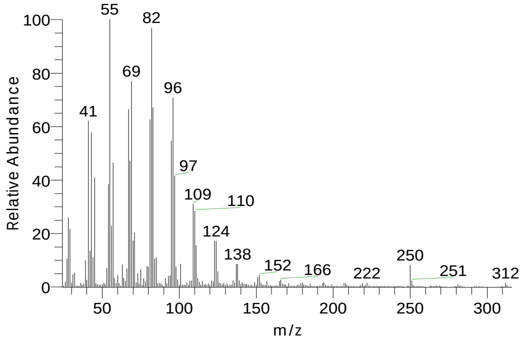


12-Methyl-E,E-2,13-octadecadien-1-ol
Formula C19H36O, MW 280, CAS# NA, Entry# 19016
(2E,15Z)-14-Methyl-2,15-octadecadien-1-ol #

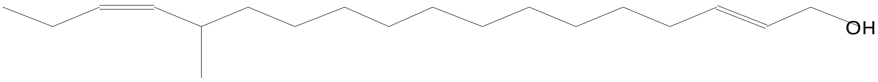


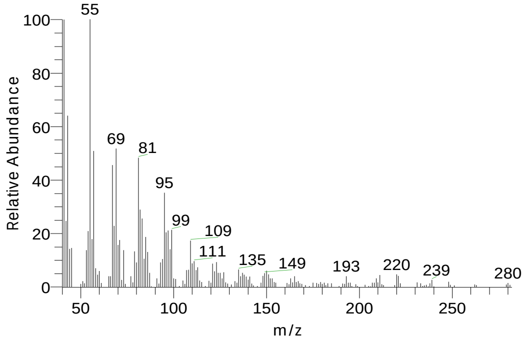


17-Octadecynoic acid
Formula C18H32O2, MW 280, CAS# 34450-18-5, Entry# 20510
$:28DZIILFGADWDKMF-UHFFFAOYSA-N

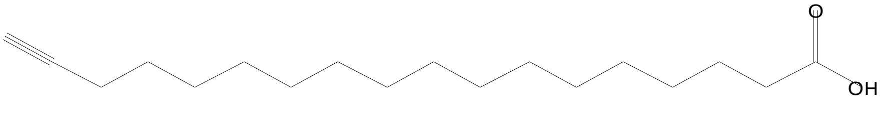


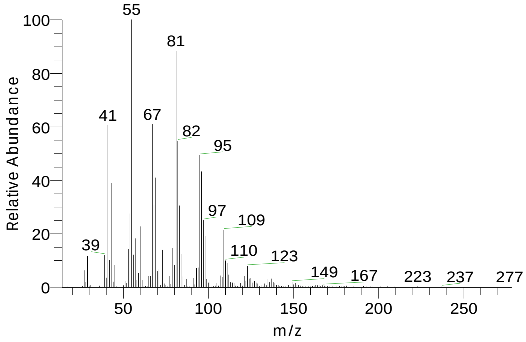

# *My GC-MS Report*

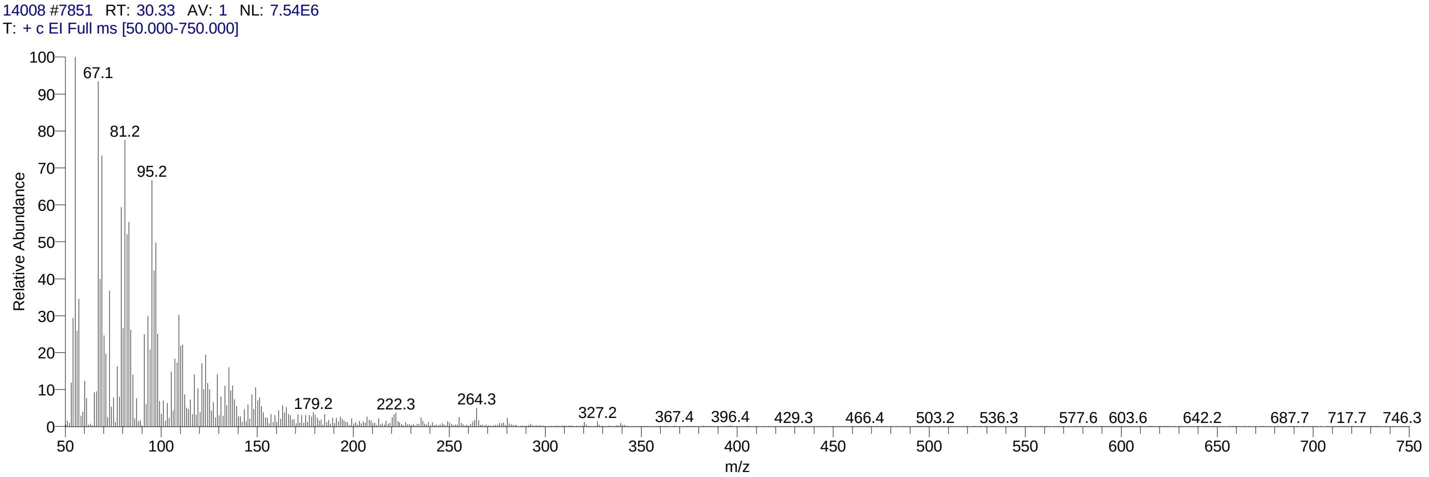


| RT | Compound Name | Area % | MF | Molecular Formula | Molecular Weight | Cas # | Library |
|---|---|---|---|---|---|---|---|
| 30.33 | 9,12-Octadecadienoyl chloride, (Z,Z)- | 6.55 | 867 | C18H31ClO | 298 | 7459-33-8 | replib |
| 30.33 | (9E,12E)-9,12-OCTADECADIENOYL CHLORIDE # | 6.55 | 866 | C18H31ClO | 298 | 7459-33-8 | WileyRegistry8e |
| 30.33 | 9,12-Octadecadienoic acid (Z,Z)-, 2-hydroxy-1-(hydroxymethyl)ethyl ester | 6.55 | 829 | C21H38O4 | 354 | 3443-82-1 | replib |
| 30.33 | E,E,Z-1,3,12-Nonadecatriene-5,14-diol | 6.55 | 823 | C19H34O2 | 294 | NA | mainlib |
| 30.33 | 9,12-Octadecadienoic acid (Z,Z)- | 6.55 | 838 | C18H32O2 | 280 | 60-33-3 | replib |

Compound Structure | Hit Spectrum

9,12-Octadecadienoyl chloride, (Z,Z)-
Formula C18H31ClO, MW 298, CAS# 7459-33-8, Entry# 4940
Linoleoyl chloride

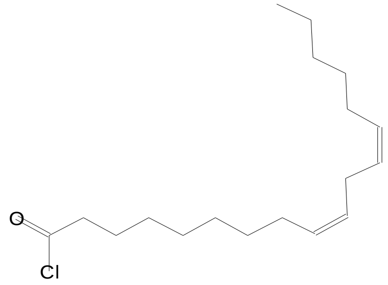


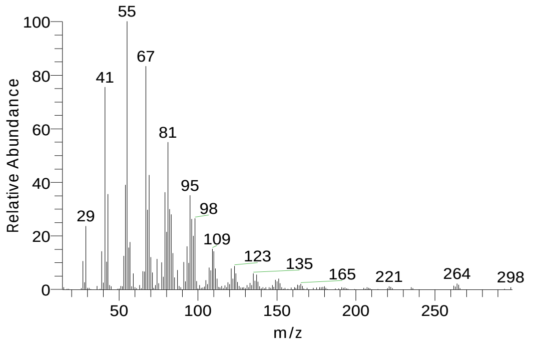


(9E,12E)-9,12-OCTADECADIENOYL CHLORIDE #
Formula C18H31ClO, MW 298, CAS# 7459-33-8, Entry# 187801
(9E,12E)-9,12-OCTADECADIENOYL CHLORIDE

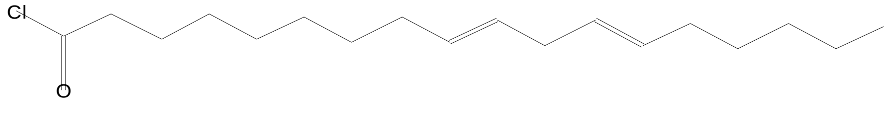


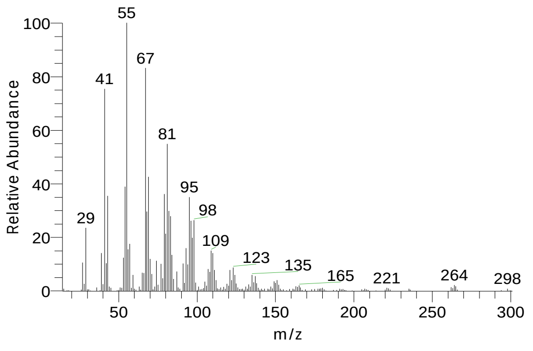

# *My GC-MS Report*

Compound Structure | Hit Spectrum

9,12-Octadecadienoic acid (Z,Z)-, 2-hydroxy-1-(hydroxymethyl)ethyl ester
Formula C21H38O4, MW 354, CAS# 3443-82-1, Entry# 8055
Linolein, 2-mono-

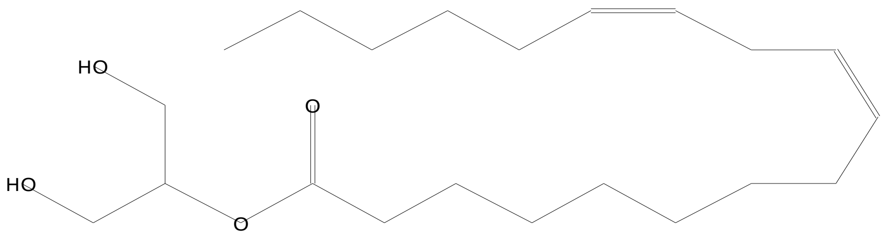


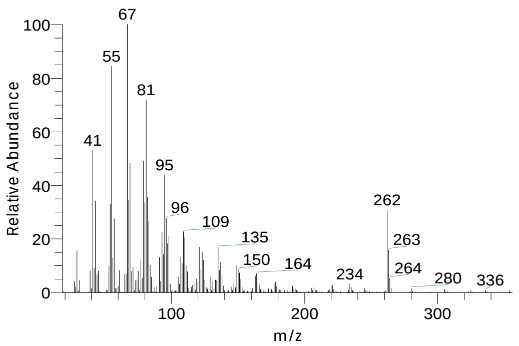


E,E,Z-1,3,12-Nonadecatriene-5,14-diol
Formula C19H34O2, MW 294, CAS# NA, Entry# 21026
(3E,12Z)-1,3,12-Nonadecatriene-5,14-diol #

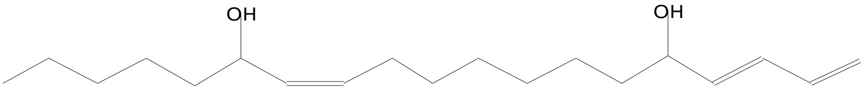


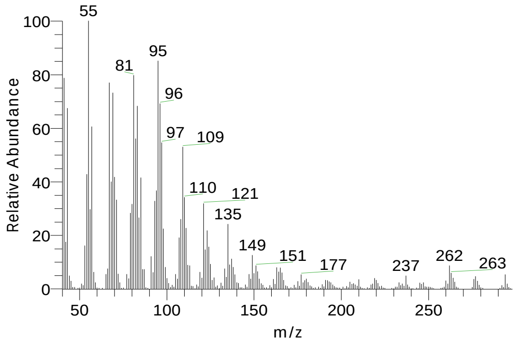


9,12-Octadecadienoic acid (Z,Z)-
Formula C18H32O2, MW 280, CAS# 60-33-3, Entry# 8057
cis-9,cis-12-Octadecadienoic acid

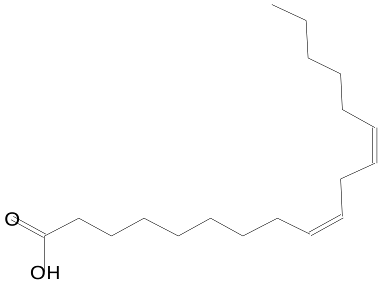


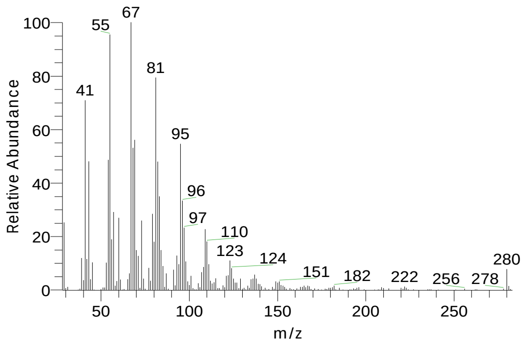


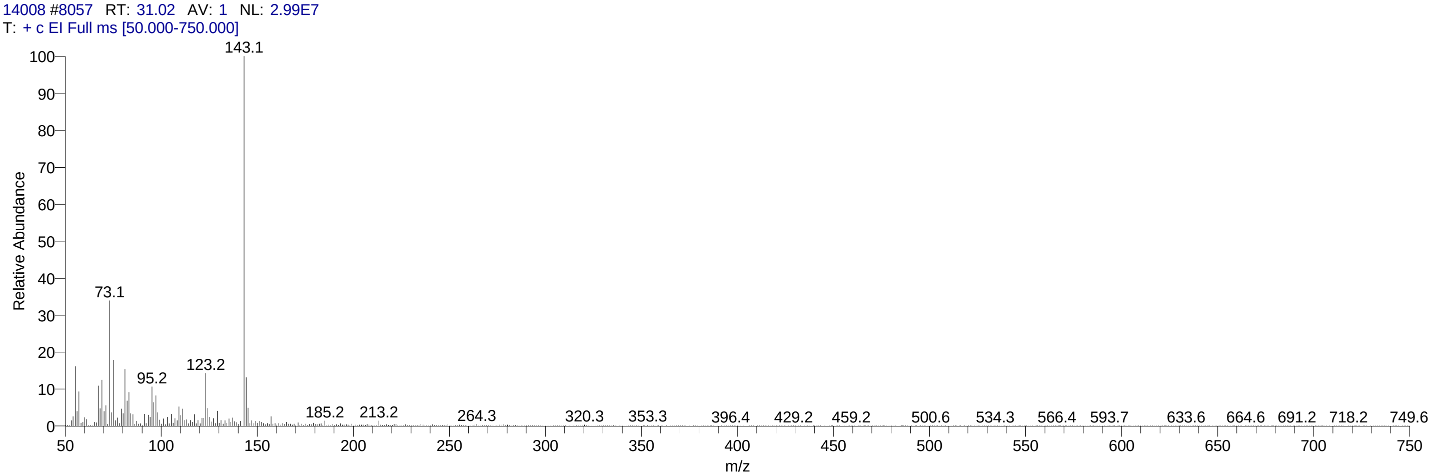


| RT | Compound Name | Area % | MF | Molecular Formula | Molecular Weight | Cas # | Library |
|---|---|---|---|---|---|---|---|
| 31.02 | Phytol, TMS derivative | 5.25 | 842 | C23H48OSi | 368 | 57397-39-4 | mainlib |
| 31.02 | PHOSPHORIC ACID, DIOCTADECYL ESTER | 5.25 | 829 | C36H75O4P | 602 | 3037-89-6 | WileyRegistry8e |

# My GC-MS Report

| RT | Compound Name | Area % | MF | Molecular Formula | Molecular Weight | Cas # | Library |
|---|---|---|---|---|---|---|---|
| 31.02 | Phytol, TMS derivative | 5.25 | 866 | C23H48OSi | 368 | 57397-39-4 | replib |
| 31.02 | SILANE, [(3,7,11,15-TETRAMETHYL-2-HEXADECENYL)OXY]TRIMETHYL- | 5.25 | 866 | C23H48OSi | 368 | 57397-39-4 | WileyRegistry8e |
| 31.02 | Phytol, TMS derivative | 5.25 | 816 | C23H48OSi | 368 | 57397-39-4 | replib |

Compound Structure | Hit Spectrum

Phytol, TMS derivative
Formula C23H48OSi, MW 368, CAS# 57397-39-4, Entry# 130652
Silane, [(3,7,11,15-tetramethyl-2-hexadecenyl)oxy]trimethyl-

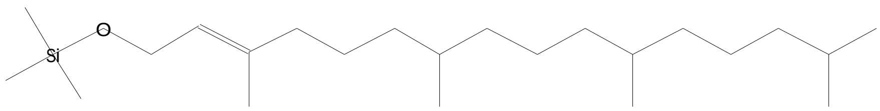


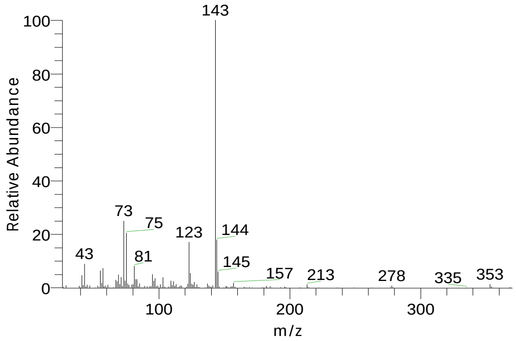


PHOSPHORIC ACID, DIOCTADECYL ESTER
Formula C36H75O4P, MW 602, CAS# 3037-89-6, Entry# 296924
DIOCTADECYL HYDROGEN PHOSPHATE

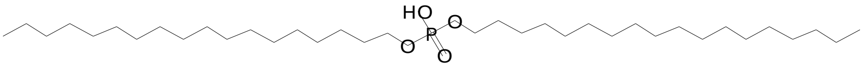


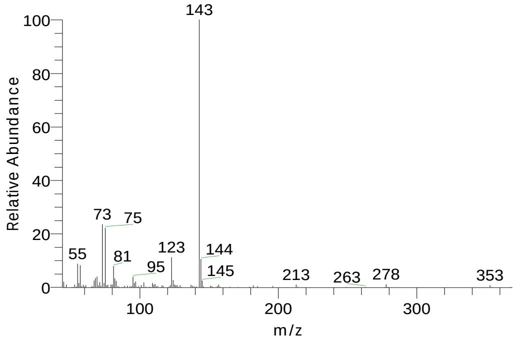


Phytol, TMS derivative
Formula C23H48OSi, MW 368, CAS# 57397-39-4, Entry# 22669
Silane, [(3,7,11,15-tetramethyl-2-hexadecenyl)oxy]trimethyl-

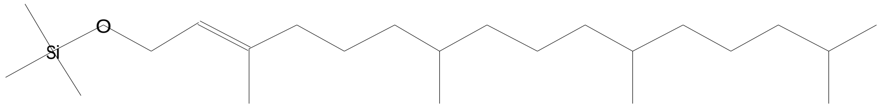


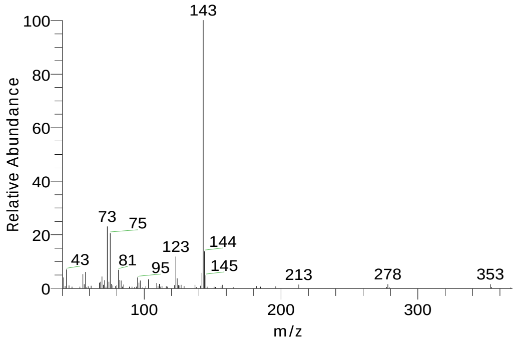


SILANE, [(3,7,11,15-TETRAMETHYL-2-HEXADECENYL)OXY]TRIMETHYL-
Formula C23H48OSi, MW 368, CAS# 57397-39-4, Entry# 239918
(2E)-3,7,11,15-TETRAMETHYL-2-HEXADECENYL TRIMETHYLSILYL ETHER #

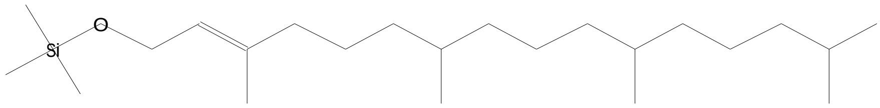


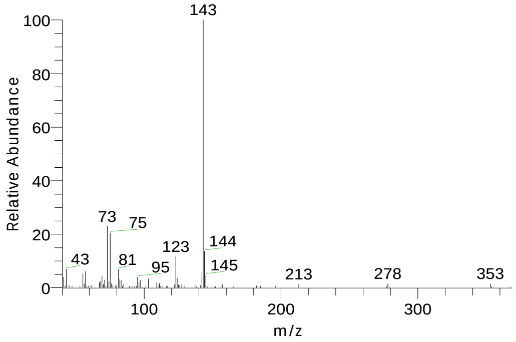

# My GC-MS Report

Compound Structure | Hit Spectrum

Phytol, TMS derivative
Formula C23H48OSi, MW 368, CAS# 57397-39-4, Entry# 22670
Silane, [(3,7,11,15-tetramethyl-2-hexadecenyl)oxy]trimethyl-

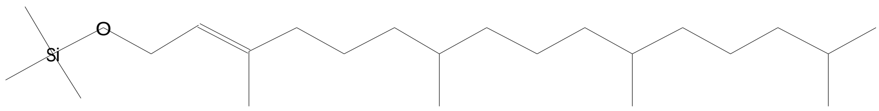


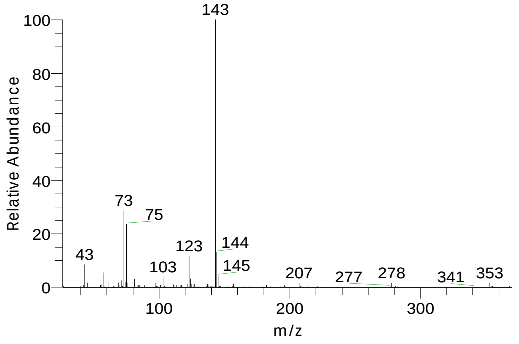


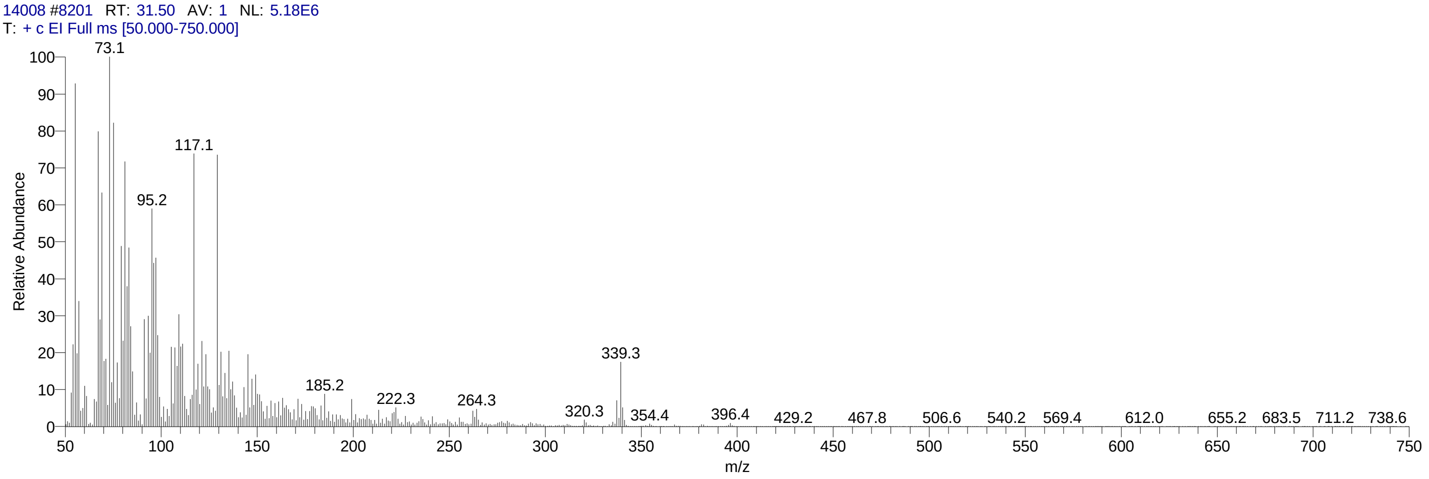


| RT | Compound Name | Area % | MF | Molecular Formula | Molecular Weight | Cas # | Library |
|---|---|---|---|---|---|---|---|
| 31.50 | 9,12-Octadecadienoic acid (Z,Z)-, TMS derivative | 5.61 | 785 | C21H40O2Si | 352 | 56259-07-5 | replib |
| 31.50 | Linoelaidic acid, trimethylsilyl ester | 5.61 | 782 | C21H40O2Si | 352 | NA | mainlib |
| 31.50 | 9,12-Octadecadienoic acid (Z,Z)-, TMS derivative | 5.61 | 778 | C21H40O2Si | 352 | 56259-07-5 | replib |
| 31.50 | LINOLSAEURE, TRIMETHYLSILYLESTER | 5.61 | 778 | C21H40O2Si | 352 | NA | WileyRegistry8e |
| 31.50 | 9,12-OCTADECADIENOIC ACID (Z,Z)-, TRIMETHYLSILYL ESTER | 5.61 | 777 | C21H40O2Si | 352 | 56259-07-5 | WileyRegistry8e |

Compound Structure | Hit Spectrum

9,12-Octadecadienoic acid (Z,Z)-, TMS derivative
Formula C21H40O2Si, MW 352, CAS# 56259-07-5, Entry# 10621
9,12-Octadecadienoic acid (Z,Z)-, trimethylsilyl ester

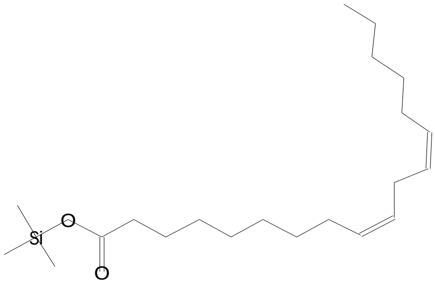


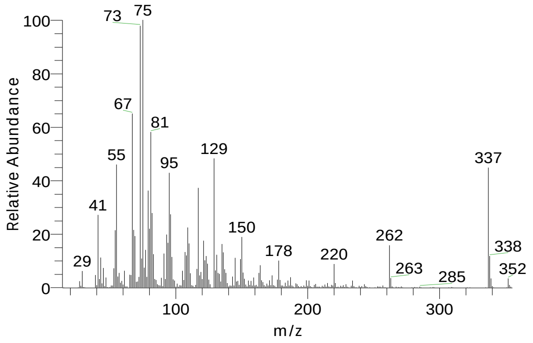

# My GC-MS Report

Compound Structure | Hit Spectrum

Linoelaidic acid, trimethylsilyl ester
Formula C21H40O2Si, MW 352, CAS# NA, Entry# 41313
$:28MXGBYOVWUYSJSN-OKLKQMLOSA-N

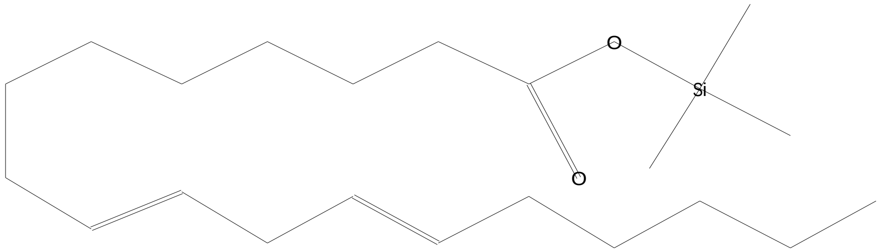


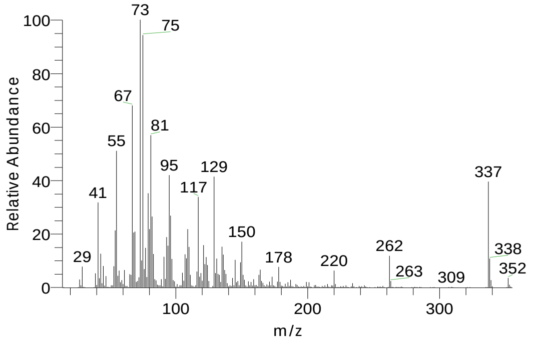


9,12-Octadecadienoic acid (Z,Z)-, TMS derivative
Formula C21H40O2Si, MW 352, CAS# 56259-07-5, Entry# 10624
9,12-Octadecadienoic acid (Z,Z)-, trimethylsilyl ester

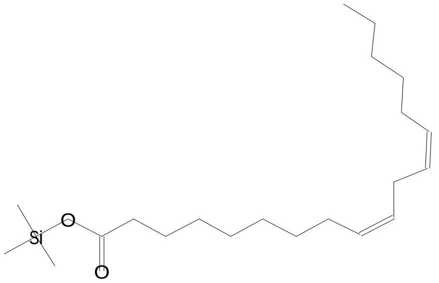


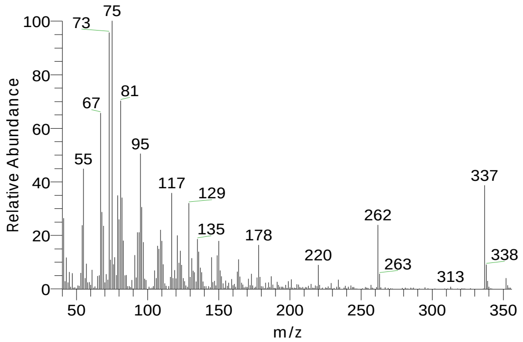


LINOLSAEURE, TRIMETHYLSILYLESTER
Formula C21H40O2Si, MW 352, CAS# NA, Entry# 385587
TRIMETHYLSILYL 9,12-OCTADECADIENOATE

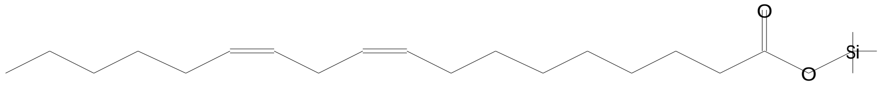


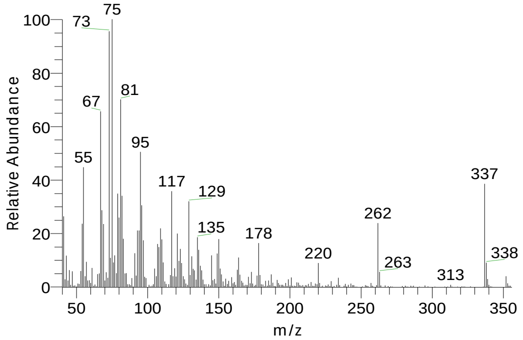


9,12-OCTADECADIENOIC ACID (Z,Z)-, TRIMETHYLSILYL ESTER
Formula C21H40O2Si, MW 352, CAS# 56259-07-5, Entry# 230120
TRIMETHYLSILYL (9E,12E)-9,12-OCTADECADIENOATE #

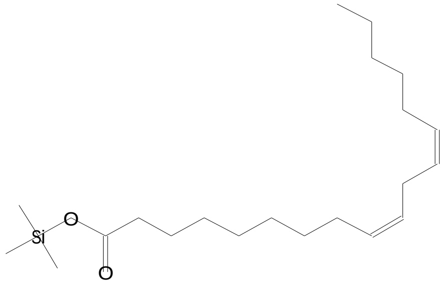


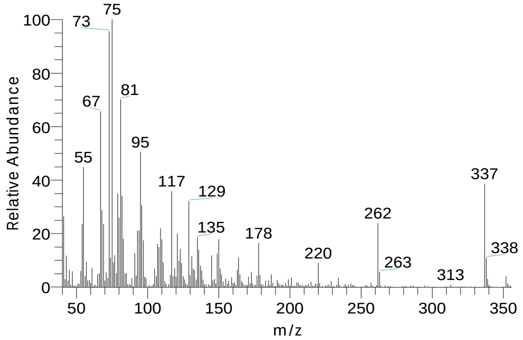

# My GC-MS Report

14008 #9813 RT: 36.91 AV: 1 NL: 4.87E6
T: + c EI Full ms [50.000-750.000]

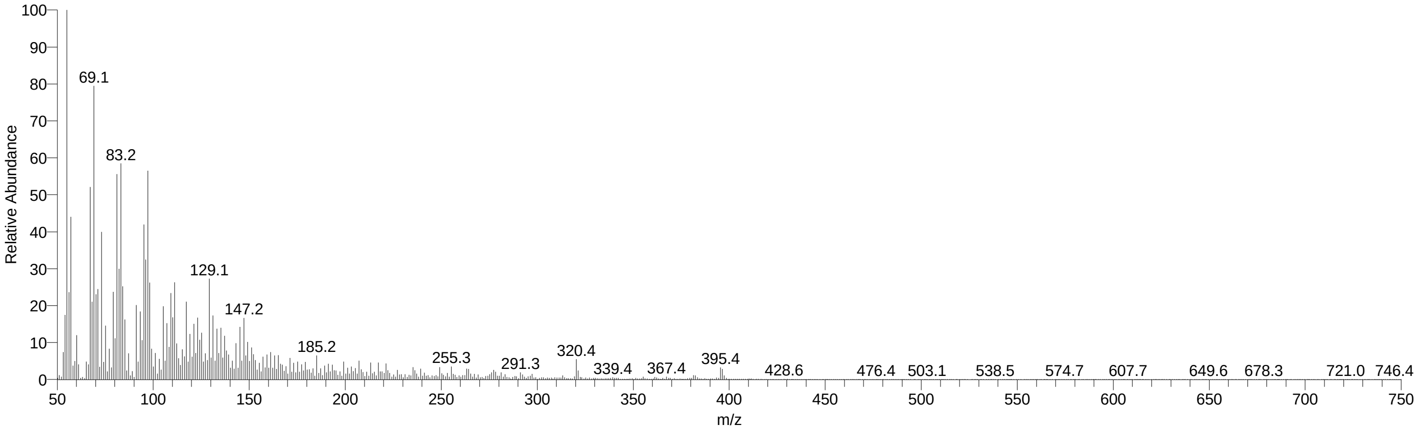


| RT | Compound Name | Area % | MF | Molecular Formula | Molecular Weight | Cas # | Library |
|---|---|---|---|---|---|---|---|
| 36.91 | 2-HYDROXY-3-[(9E)-9-OCTADEC ENOYLOXY]PROPYL (9E)-9-OCTADECENOATE # | 1.40 | 793 | C39H72O5 | 620 | 2465-32-9 | WileyRegistry8e |
| 36.91 | 9-OCTADECENOIC ACID (Z)- | 1.40 | 796 | C18H34O2 | 282 | 112-80-1 | WileyRegistry8e |
| 36.91 | cis-13-Eicosenoic acid | 1.40 | 795 | C20H38O2 | 310 | 17735-94-3 | mainlib |
| 36.91 | (E)-13-Docosenoic acid | 1.40 | 778 | C22H42O2 | 338 | 506-33-2 | mainlib |
| 36.91 | 9-Octadecenoic acid, 1,2,3-propanetriyl ester, (E,E,E)- | 1.40 | 758 | C57H104O6 | 884 | 537-39-3 | mainlib |

Compound Structure | Hit Spectrum

2-HYDROXY-3-[(9E)-9-OCTADECENOYLOXY]PROPYL (9E)-9-OCTADECENOATE #
Formula C39H72O5, MW 620, CAS# 2465-32-9, Entry# 298152
(Z,Z)-1,3-DIOCTADECENOYL GLYCEROL

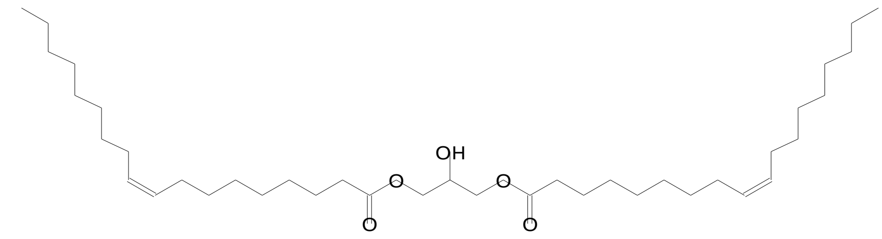


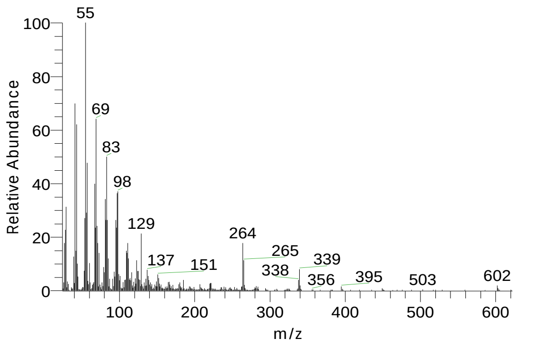


9-OCTADECENOIC ACID (Z)-
Formula C18H34O2, MW 282, CAS# 112-80-1, Entry# 172910
OCTADEC-9-ENOIC ACID

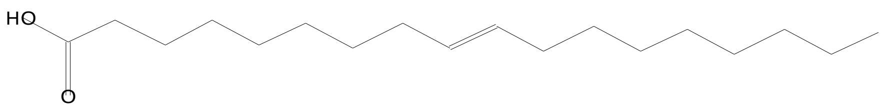


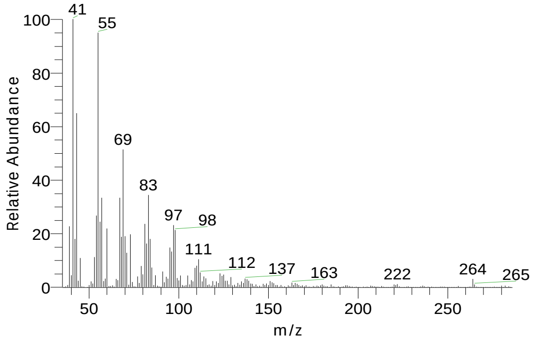

# My GC-MS Report

Compound Structure | Hit Spectrum

cis-13-Eicosenoic acid
Formula C20H38O2, MW 310, CAS# 17735-94-3, Entry# 20259
$:28URXZXNYJPAJJOQ-FPLPWBNLSA-N

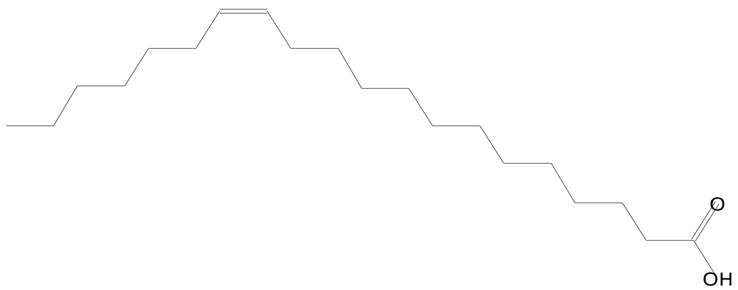


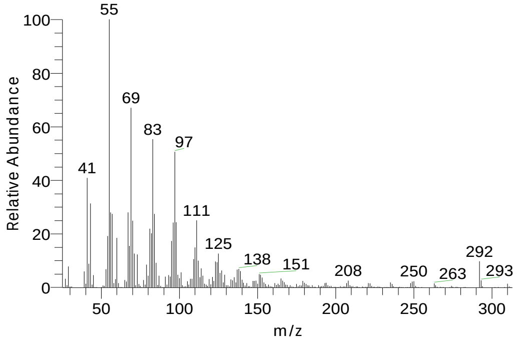


(E)-13-Docosenoic acid
Formula C22H42O2, MW 338, CAS# 506-33-2, Entry# 2721
13-Docosenoic acid, (E)-

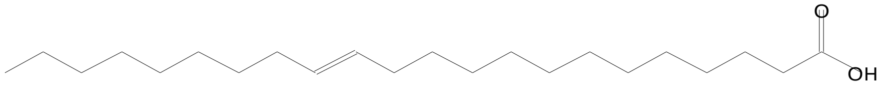


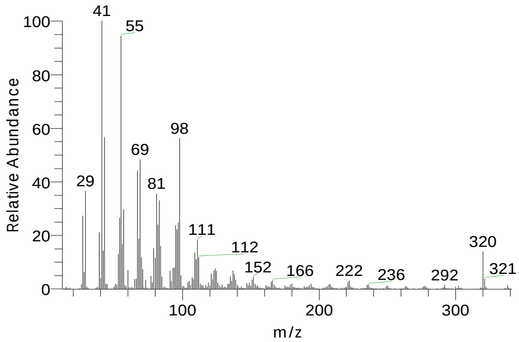


9-Octadecenoic acid, 1,2,3-propanetriyl ester, (E,E,E)-
Formula C57H104O6, MW 884, CAS# 537-39-3, Entry# 20268
2,3-Bis[(9E)-9-octadecenoyloxy]propyl (9E)-9-octadecenoate #

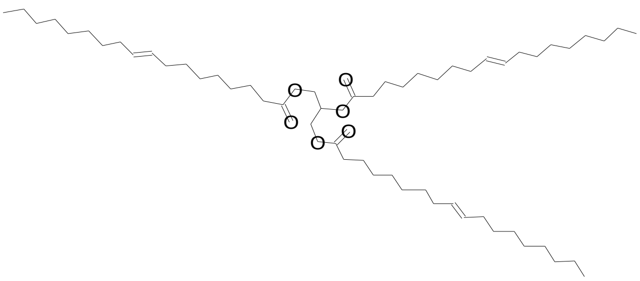


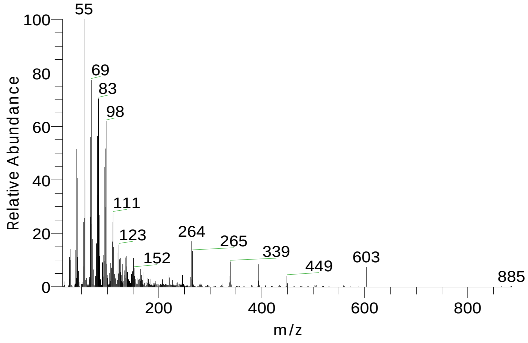


14008 #9843 RT: 37.01 AV: 1 NL: 4.55E6
T: + c EI Full ms [50.000-750.000]

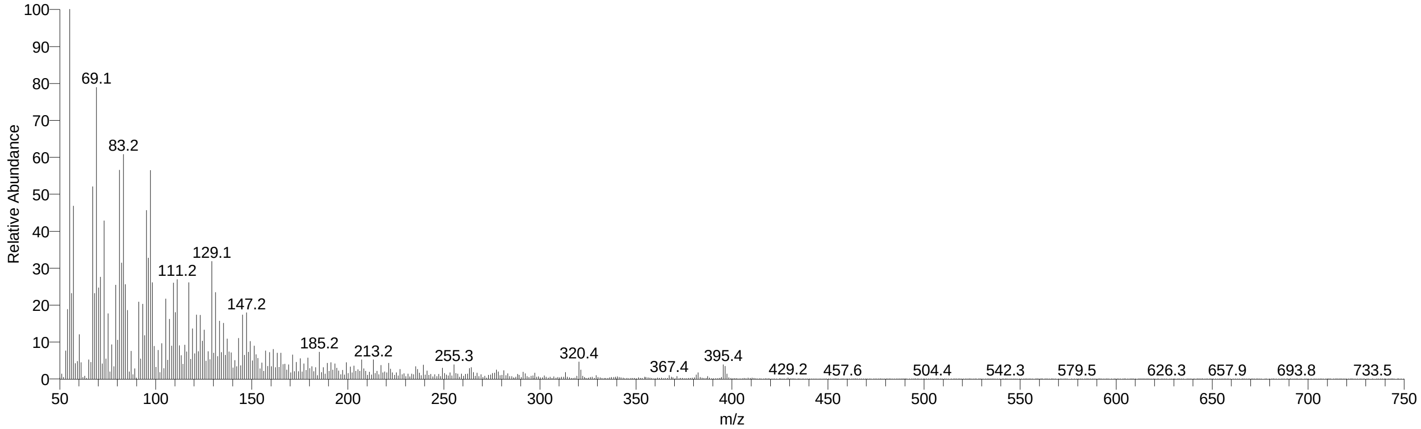


| RT | Compound Name | Area % | MF | Molecular Formula | Molecular Weight | Cas # | Library |
|---|---|---|---|---|---|---|---|
| 37.01 | 2-HYDROXY-3-[(9E)-9-OCTADECENOYLOXY]PROPYL (9E)-9-OCTADECENOATE # | 0.72 | 787 | C39H72O5 | 620 | 2465-32-9 | WileyRegistry8e |
| 37.01 | HAHNFETT | 0.72 | 773 | N/A | 0 | NA | WileyRegistry8e |

# *My GC-MS Report*

| RT | Compound Name | Area % | MF | Molecular Formula | Molecular Weight | Cas # | Library |
|---|---|---|---|---|---|---|---|
| 37.01 | HAHNFETT | 0.72 | 773 | N/A | 0 | NA | WileyRegistry8e |
| 37.01 | (E)-13-Docosenoic acid | 0.72 | 769 | C22H42O2 | 338 | 506-33-2 | mainlib |
| 37.01 | cis-13-Eicosenoic acid | 0.72 | 784 | C20H38O2 | 310 | 17735-94-3 | mainlib |

Compound Structure | Hit Spectrum

2-HYDROXY-3-[(9E)-9-OCTADECENOYLOXY]PROPYL (9E)-9-OCTADECENOATE #
Formula C39H72O5, MW 620, CAS# 2465-32-9, Entry# 298152
(Z,Z)-1,3-DIOCTADECENOYL GLYCEROL

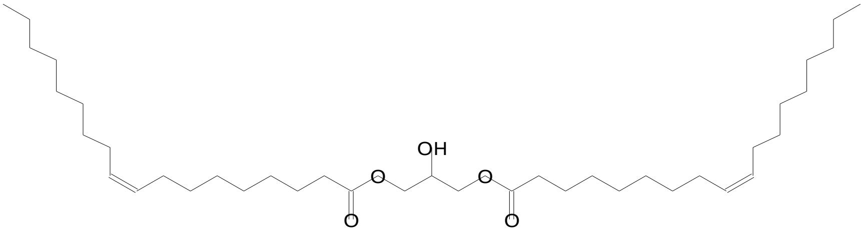


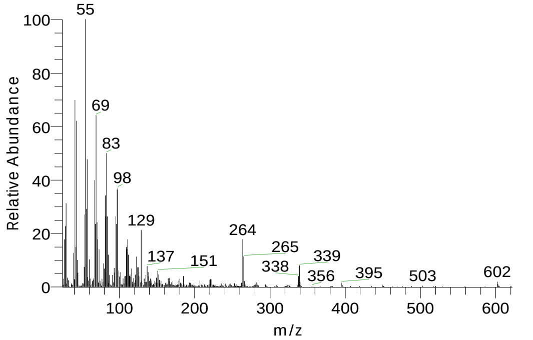


HAHNFETT
Formula , MW 0, CAS# NA, Entry# 305496

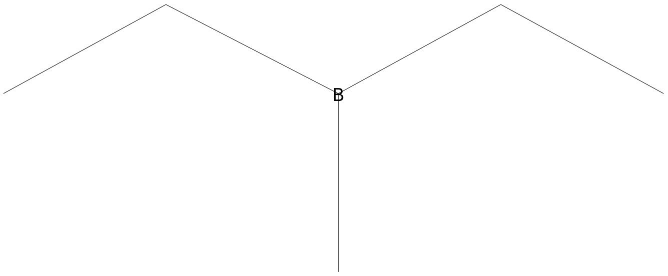


SI 757, RSI 773, WileyRegistry8e, Entry# 305496, CAS# NA, HAHNFETT

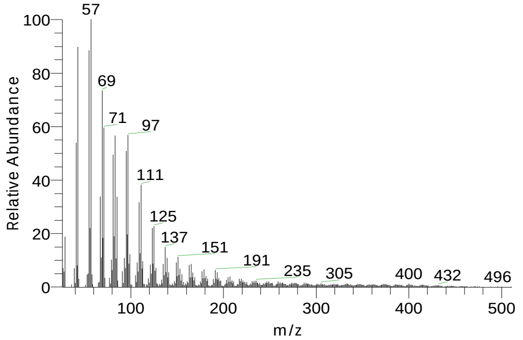


HAHNFETT
Formula , MW 0, CAS# NA, Entry# 391160

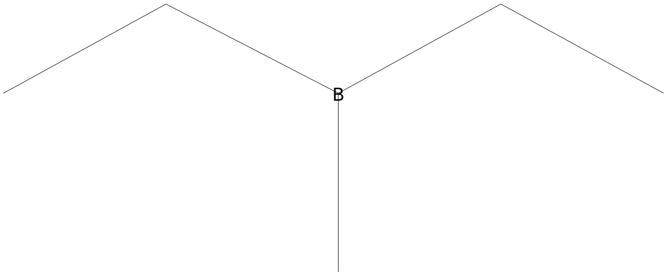


SI 757, RSI 773, WileyRegistry8e, Entry# 391160, CAS# NA, HAHNFETT

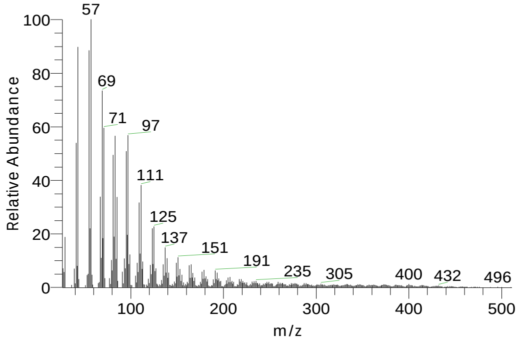


(E)-13-Docosenoic acid
Formula C22H42O2, MW 338, CAS# 506-33-2, Entry# 2721
13-Docosenoic acid, (E)-

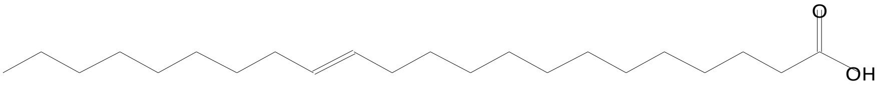


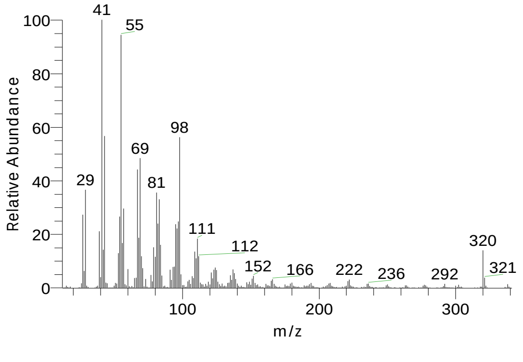

# My GC-MS Report

Compound Structure | Hit Spectrum

cis-13-Eicosenoic acid
Formula C20H38O2, MW 310, CAS# 17735-94-3, Entry# 20259
$:28URXZXNYJPAJJOQ-FPLPWBNLSA-N

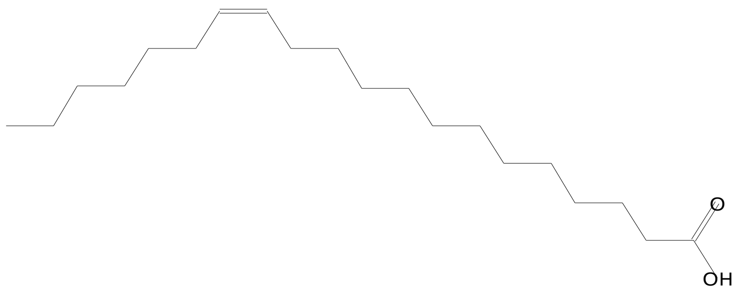


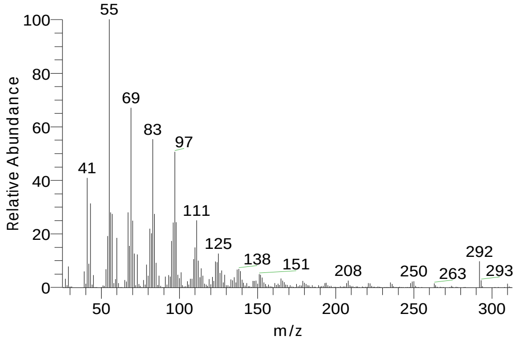


14008 #10116 RT: 37.92 AV: 1 NL: 3.77E6
T: + c EI Full ms [50.000-750.000]

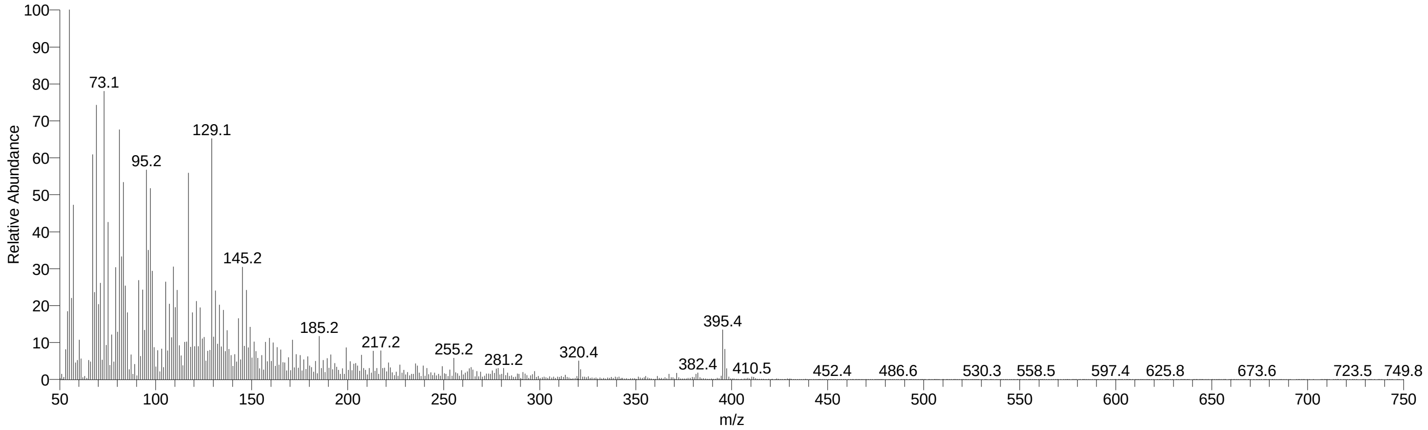


| RT | Compound Name | Area % | MF | Molecular Formula | Molecular Weight | Cas # | Library |
|---|---|---|---|---|---|---|---|
| 37.92 | 13-Docosenoic acid, (Z)-, TMS derivative | 1.06 | 757 | C25H50O2Si | 410 | NA | mainlib |
| 37.92 | .psi.,.psi.-Carotene, 1,1',2,2'-tetrahydro-1,1'-dimethoxy- | 1.06 | 730 | C42H64O2 | 600 | 13833-01-7 | mainlib |
| 37.92 | .PSI.,.PSI.-CAROTENE, 1,1',2,2'-TETRAHYDRO-1,1'-DIMETHOXY- | 1.06 | 729 | C42H64O2 | 600 | 13833-01-7 | WileyRegistry8e |
| 37.92 | Trilinolein | 1.06 | 739 | C57H98O6 | 878 | 537-40-6 | mainlib |
| 37.92 | Tricyclo[20.8.0.0(7,16)]triacontane, 1(22),7(16)-diepoxy- | 1.06 | 798 | C30H52O2 | 444 | NA | mainlib |

Compound Structure | Hit Spectrum

13-Docosenoic acid, (Z)-, TMS derivative
Formula C25H50O2Si, MW 410, CAS# NA, Entry# 41395
cis-13-Docosenoic acid, trimethylsilyl ester

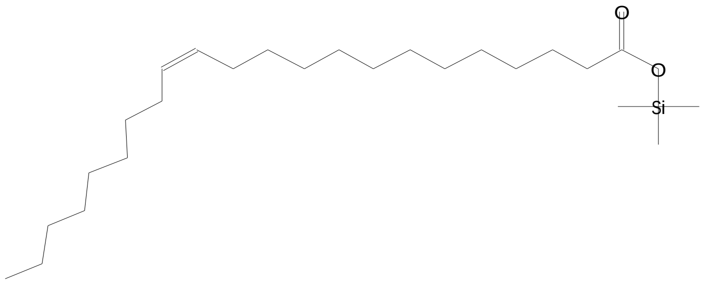


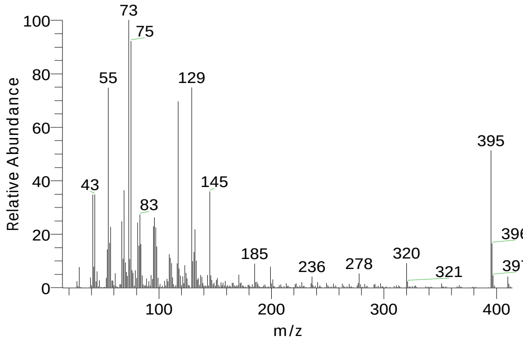

# My GC-MS Report

Compound Structure | Hit Spectrum

.psi.,.psi.-Carotene, 1,1',2,2'-tetrahydro-1,1'-dimethoxy-
Formula C42H64O2, MW 600, CAS# 13833-01-7, Entry# 41205
Lycopene, 1,1',2,2'-tetrahydro-1,1'-dimethoxy-, all-trans-

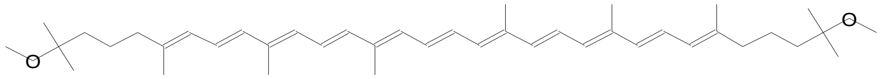


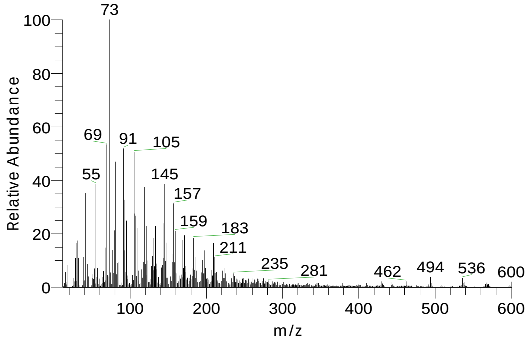


.PSI.,.PSI.-CAROTENE, 1,1',2,2'-TETRAHYDRO-1,1'-DIMETHOXY-
Formula C42H64O2, MW 600, CAS# 13833-01-7, Entry# 296796
1,1',2,2'-TETRAHYDRO-PSI,PSI-CAROTENE #

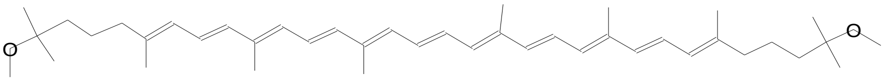


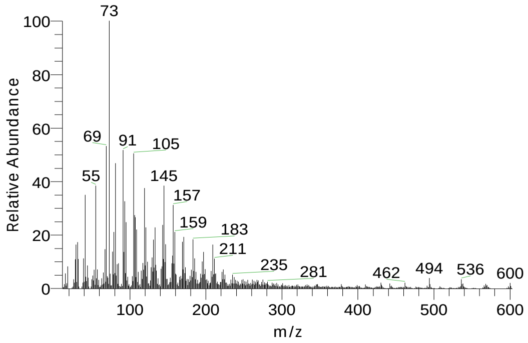


Trilinolein
Formula C57H98O6, MW 878, CAS# 537-40-6, Entry# 32618
9,12-Octadecadienoic acid (Z,Z)-, 1,2,3-propanetriyl ester

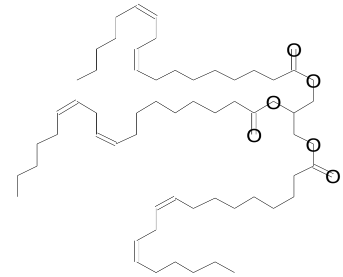


SI 725, RSI 739, mainlib, Entry# 32618, CAS# 537-40-6, Trilinolein

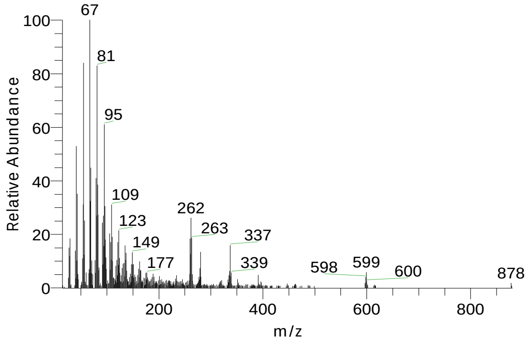


Tricyclo[20.8.0.0(7,16)]triacontane, 1(22),7(16)-diepoxy-
Formula C30H52O2, MW 444, CAS# NA, Entry# 20028
$:28XVGPDAFFXRGERF-UHFFFAOYSA-N

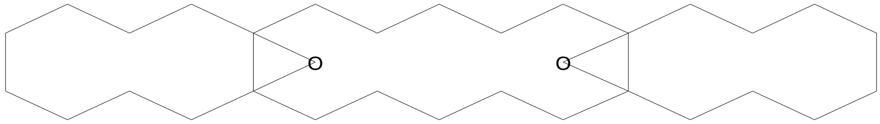


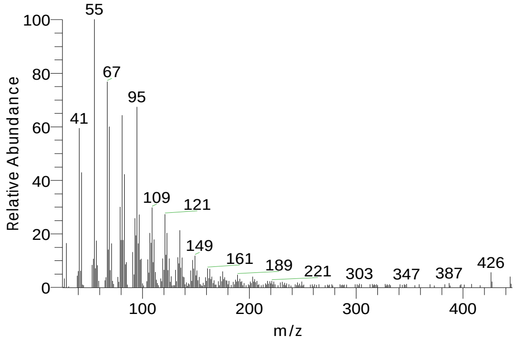

# *My GC-MS Report*

14008 #10606 RT: 39.57 AV: 1 NL: 3.55E6
T: + c EI Full ms [50.000-750.000]

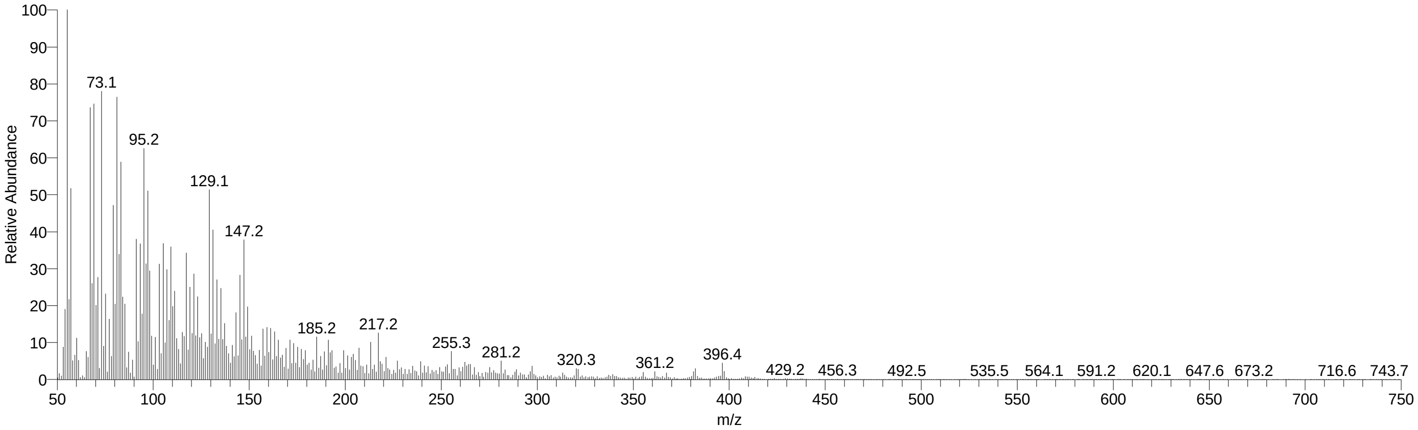


| RT | Compound Name | Area % | MF | Molecular Formula | Molecular Weight | Cas # | Library |
|---|---|---|---|---|---|---|---|
| 39.57 | STIGMAST-5-EN-3-OL, (3á,24S)- | 1.05 | 822 | C29H50O | 414 | 83-47-6 | WileyRegistry8e |
| 39.57 | 1-Heptatriacotanol | 1.05 | 839 | C37H76O | 536 | 105794-58-9 | mainlib |
| 39.57 | 03027205002 FLAVONE 4'-OH,5-OH,7-DI-O-GLUCOSIDE | 1.05 | 776 | C27H30O15 | 594 | NA | WileyRegistry8e |
| 39.57 | Rhodopin | 1.05 | 764 | C40H58O | 554 | 105-92-0 | mainlib |
| 39.57 | .PSI.,.PSI.-CAROTENE, 1,2-DIHYDRO-1-HYDROXY- | 1.05 | 761 | C40H58O | 554 | 105-92-0 | WileyRegistry8e |

| Compound Structure | Hit Spectrum |
|---|---|

STIGMAST-5-EN-3-OL, (3á,24S)-
Formula C29H50O, MW 414, CAS# 83-47-6, Entry# 262359
STIGMAST-5-EN-3-OL #

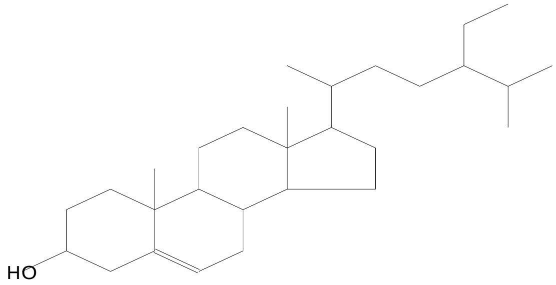


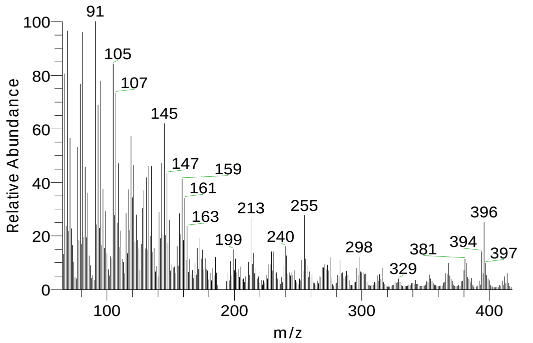


1-Heptatriacotanol
Formula C37H76O, MW 536, CAS# 105794-58-9, Entry# 7279
1-Heptatriacontanol #

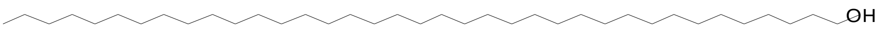


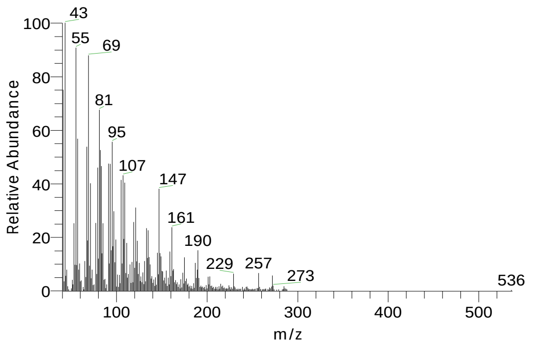

# *My GC-MS Report*

Compound Structure | Hit Spectrum

03027205002 FLAVONE 4'-OH,5-OH,7-DI-O-GLUCOSIDE
Formula C27H30O15, MW 594, CAS# NA, Entry# 296184

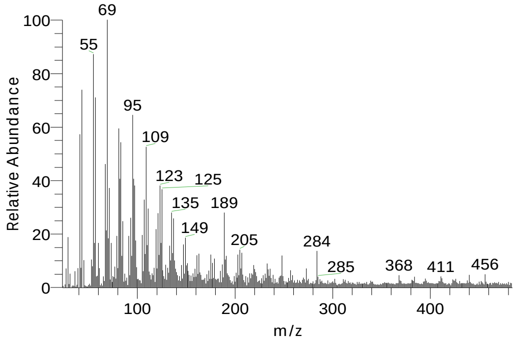


SI 762, RSI 764, mainlib, Entry# 34358, CAS# 105-92-0, Rhodopin

Rhodopin
Formula C40H58O, MW 554, CAS# 105-92-0, Entry# 34358
.psi.,.psi.-Carotene, 1,2-dihydro-1-hydroxy-

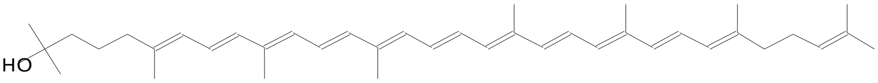


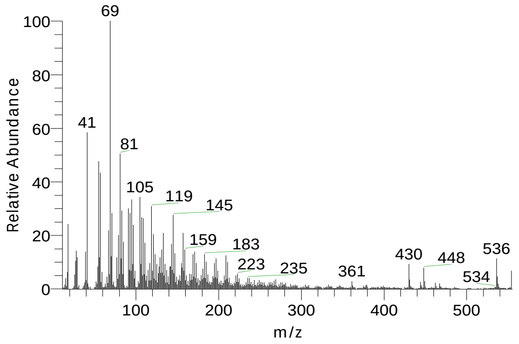


.PSI.,.PSI.-CAROTENE, 1,2-DIHYDRO-1-HYDROXY-
Formula C40H58O, MW 554, CAS# 105-92-0, Entry# 292854
1,2-DIHYDRO-PSI,PSI-CAROTENE #

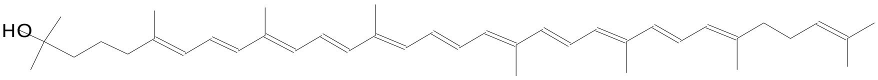


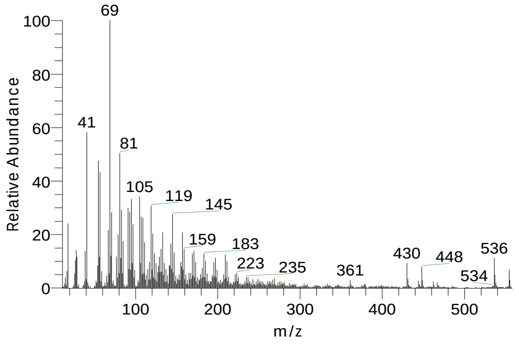


14008 #10643 RT: 39.69 AV: 1 NL: 3.90E6
T: + c EI Full ms [50.000-750.000]

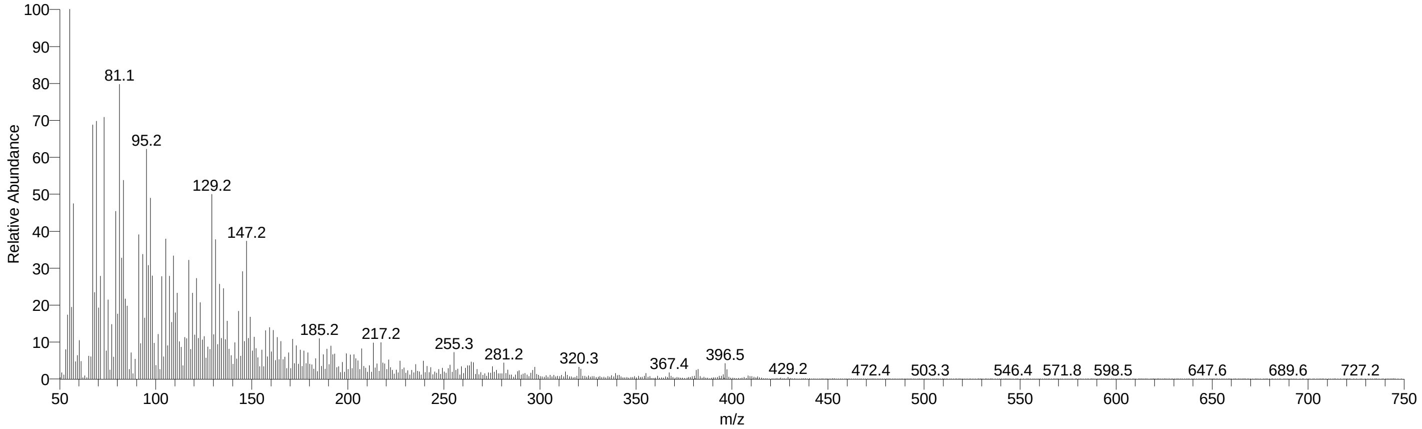


| RT | Compound Name | Area % | MF | Molecular Formula | Molecular Weight | Cas # | Library |
|---|---|---|---|---|---|---|---|
| 39.69 | STIGMAST-5-EN-3-OL, (3á,24S)- | 0.68 | 828 | C29H50O | 414 | 83-47-6 | WileyRegistry8e |
| 39.69 | 1-Heptatriacotanol | 0.68 | 842 | C37H76O | 536 | 105794-58-9 | mainlib |

# My GC-MS Report

| RT | Compound Name | Area % | MF | Molecular Formula | Molecular Weight | Cas # | Library |
|---|---|---|---|---|---|---|---|
| 39.69 | 03027205002 FLAVONE 4'-OH,5-OH,7-DI-O-GLUCOSIDE | 0.68 | 779 | C27H30O15 | 594 | NA | WileyRegistry8e |
| 39.69 | Rhodopin | 0.68 | 759 | C40H58O | 554 | 105-92-0 | mainlib |
| 39.69 | .psi.,.psi.-Carotene, 1,1',2,2'-tetrahydro-1,1'-dimethoxy- | 0.68 | 757 | C42H64O2 | 600 | 13833-01-7 | mainlib |

Compound Structure | Hit Spectrum

STIGMAST-5-EN-3-OL, (3á,24S)-
Formula C29H50O, MW 414, CAS# 83-47-6, Entry# 262359
STIGMAST-5-EN-3-OL #

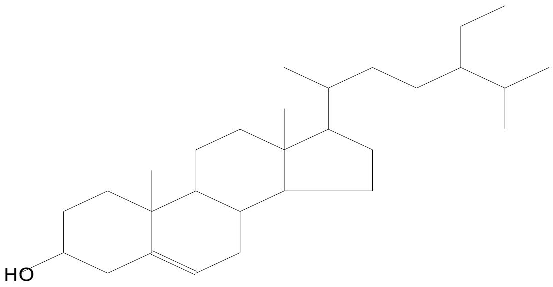


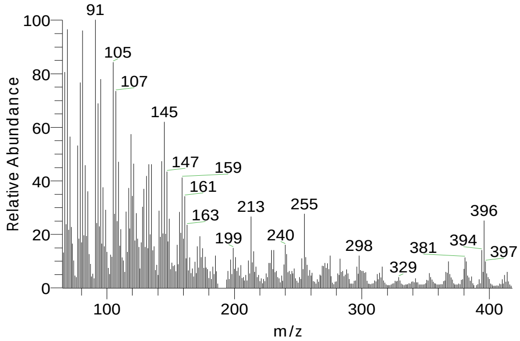


1-Heptatriacotanol
Formula C37H76O, MW 536, CAS# 105794-58-9, Entry# 7279
1-Heptatriacontanol #

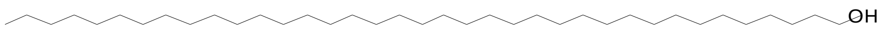


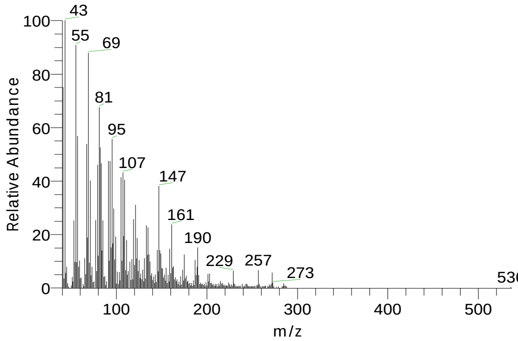


03027205002 FLAVONE 4'-OH,5-OH,7-DI-O-GLUCOSIDE
Formula C27H30O15, MW 594, CAS# NA, Entry# 296184

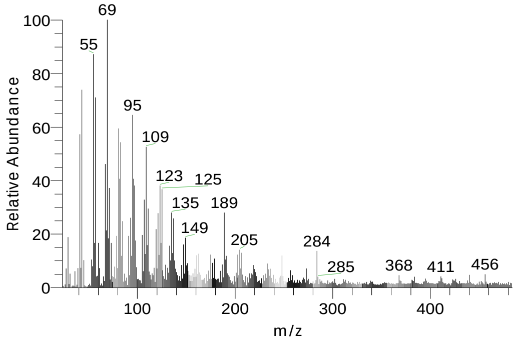


Rhodopin
Formula C40H58O, MW 554, CAS# 105-92-0, Entry# 34358
.psi.,.psi.-Carotene, 1,2-dihydro-1-hydroxy-

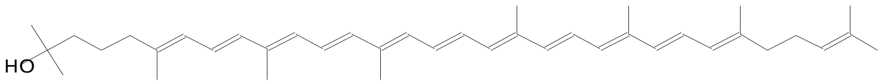


SI 757, RSI 759, mainlib, Entry# 34358, CAS# 105-92-0, Rhodopin

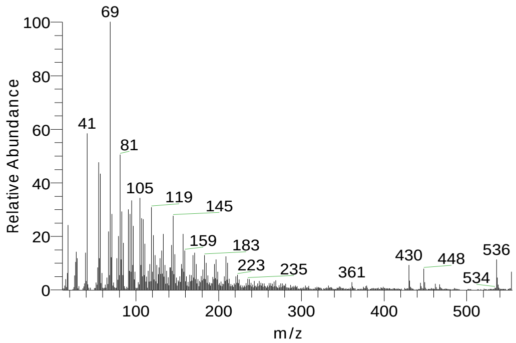

# My GC-MS Report

Compound Structure | Hit Spectrum

.psi.,.psi.-Carotene, 1,1',2,2'-tetrahydro-1,1'-dimethoxy-
Formula C42H64O2, MW 600, CAS# 13833-01-7, Entry# 41205
Lycopene, 1,1',2,2'-tetrahydro-1,1'-dimethoxy-, all-trans-

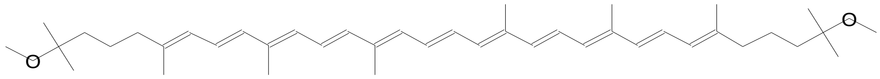

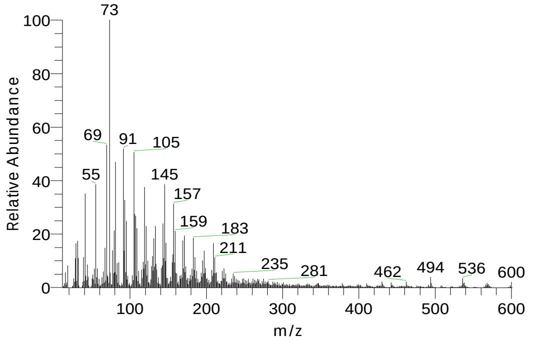


14008 #10758 RT: 40.08 AV: 1 NL: 3.96E6
T: + c EI Full ms [50.000-750.000]

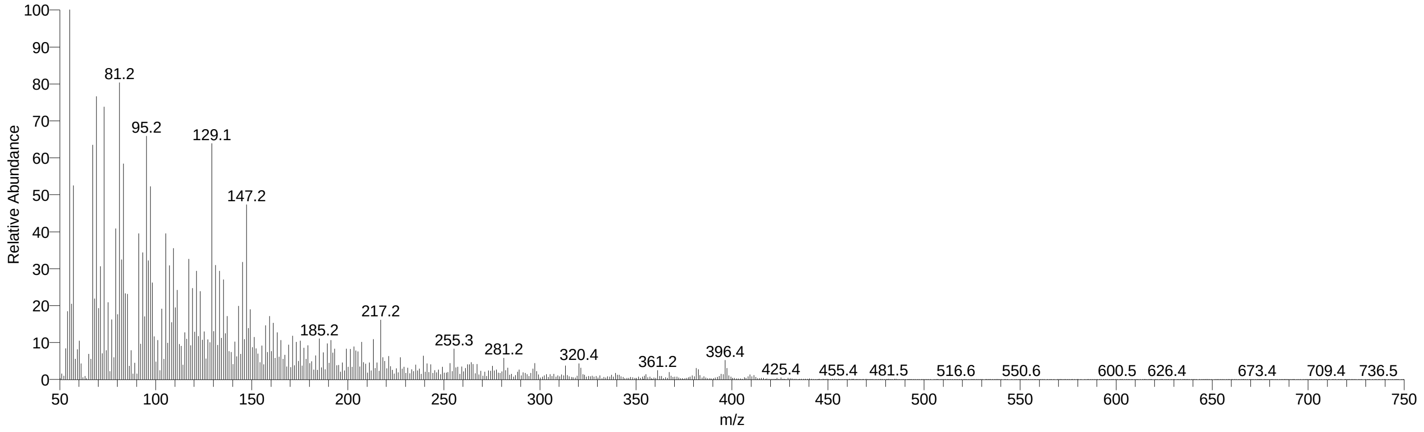


| RT | Compound Name | Area % | MF | Molecular Formula | Molecular Weight | Cas # | Library |
|---|---|---|---|---|---|---|---|
| 40.08 | 03027205002 FLAVONE 4'-OH,5-OH,7-DI-O-GLUCOSIDE | 0.52 | 785 | C27H30O15 | 594 | NA | WileyRegistry8e |
| 40.08 | 1-Heptatriacotanol | 0.52 | 842 | C37H76O | 536 | 105794-58-9 | mainlib |
| 40.08 | Rhodopin | 0.52 | 772 | C40H58O | 554 | 105-92-0 | mainlib |
| 40.08 | .psi.,.psi.-Carotene, 1,1',2,2'-tetrahydro-1,1'-dimethoxy- | 0.52 | 768 | C42H64O2 | 600 | 13833-01-7 | mainlib |
| 40.08 | .PSI.,.PSI.-CAROTENE, 1,2-DIHYDRO-1-HYDROXY- | 0.52 | 769 | C40H58O | 554 | 105-92-0 | WileyRegistry8e |

Compound Structure | Hit Spectrum

03027205002 FLAVONE 4'-OH,5-OH,7-DI-O-GLUCOSIDE
Formula C27H30O15, MW 594, CAS# NA, Entry# 296184

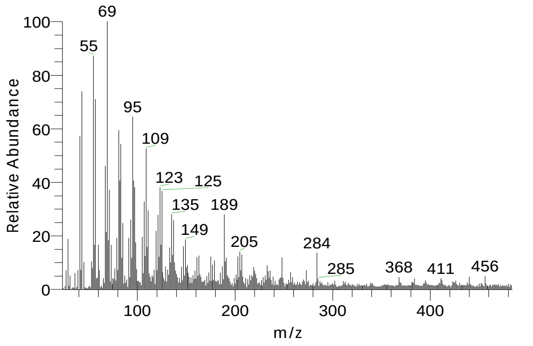

# *My GC-MS Report*

Compound Structure | Hit Spectrum

1-Heptatriacotanol
Formula C37H76O, MW 536, CAS# 105794-58-9, Entry# 7279
1-Heptatriacontanol #

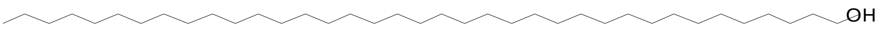


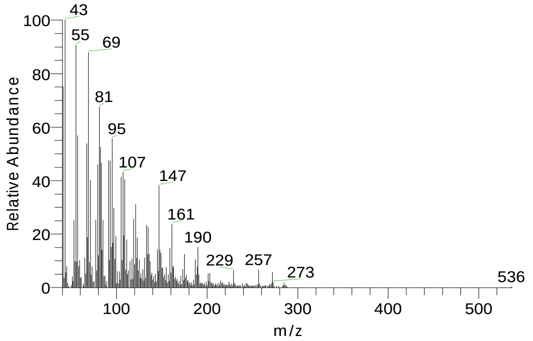


SI 770, RSI 772, mainlib, Entry# 34358, CAS# 105-92-0, Rhodopin

Rhodopin
Formula C40H58O, MW 554, CAS# 105-92-0, Entry# 34358
.psi.,.psi.-Carotene, 1,2-dihydro-1-hydroxy-

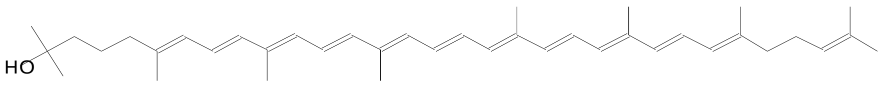


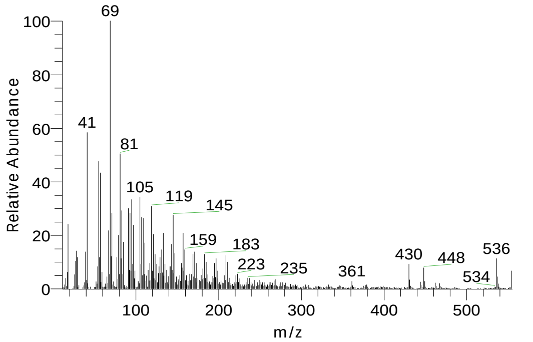


.psi.,.psi.-Carotene, 1,1',2,2'-tetrahydro-1,1'-dimethoxy-
Formula C42H64O2, MW 600, CAS# 13833-01-7, Entry# 41205
Lycopene, 1,1',2,2'-tetrahydro-1,1'-dimethoxy-, all-trans-

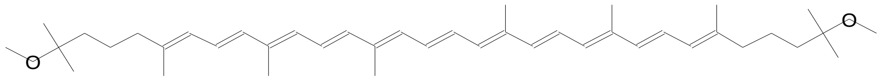


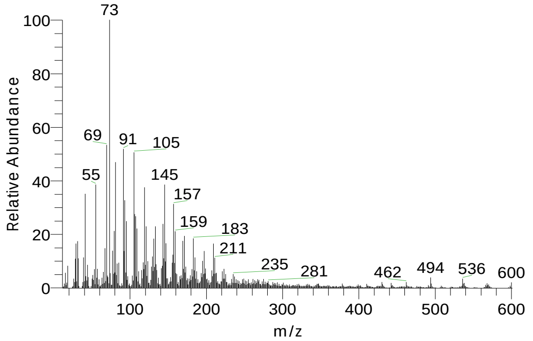


.PSI.,.PSI.-CAROTENE, 1,2-DIHYDRO-1-HYDROXY-
Formula C40H58O, MW 554, CAS# 105-92-0, Entry# 292854
1,2-DIHYDRO-PSI,PSI-CAROTENE #

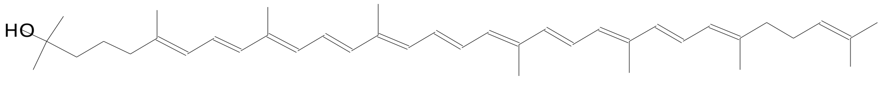


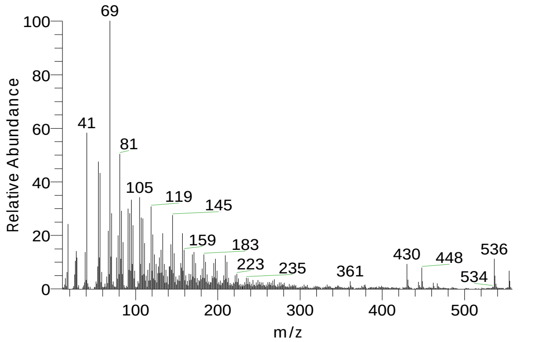

# *My GC-MS Report*

14008 #10874 RT: 40.46 AV: 1 NL: 5.67E6
T: + c EI Full ms [50.000-750.000]

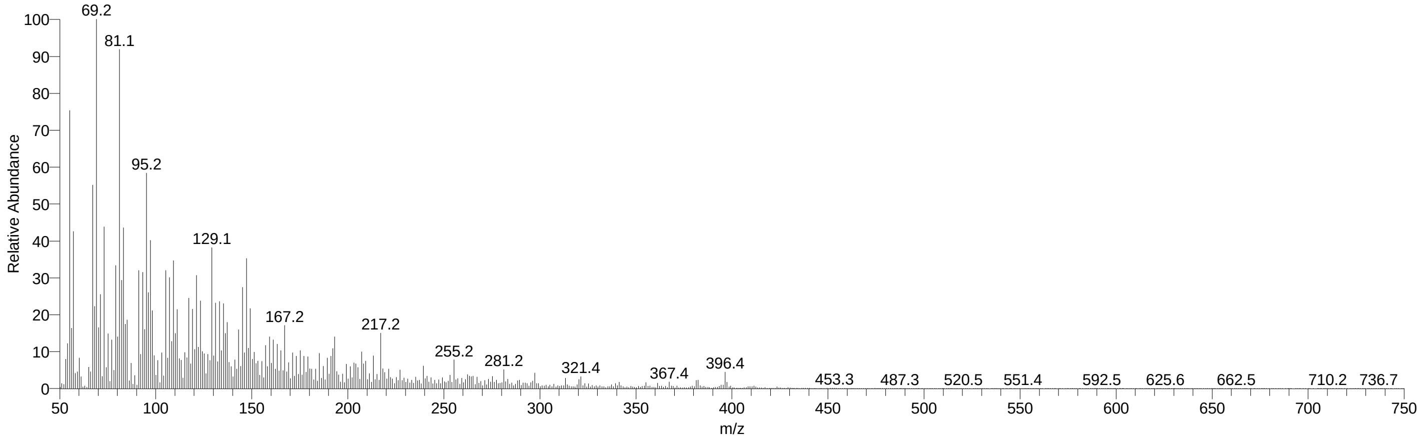


| RT | Compound Name | Area % | MF | Molecular Formula | Molecular Weight | Cas # | Library |
|---|---|---|---|---|---|---|---|
| 40.46 | STIGMAST-5-EN-3-OL, (3á,24S)- | 0.63 | 824 | C29H50O | 414 | 83-47-6 | WileyRegistry8e |
| 40.46 | 1-Heptatriacotanol | 0.63 | 842 | C37H76O | 536 | 105794-58-9 | mainlib |
| 40.46 | 03027205002 FLAVONE 4'-OH,5-OH,7-DI-O-GLUCOSIDE | 0.63 | 781 | C27H30O15 | 594 | NA | WileyRegistry8e |
| 40.46 | Rhodopin | 0.63 | 775 | C40H58O | 554 | 105-92-0 | mainlib |
| 40.46 | .PSI.,.PSI.-CAROTENE, 1,2-DIHYDRO-1-HYDROXY- | 0.63 | 772 | C40H58O | 554 | 105-92-0 | WileyRegistry8e |

| Compound Structure | Hit Spectrum |
|---|---|

STIGMAST-5-EN-3-OL, (3á,24S)-
Formula C29H50O, MW 414, CAS# 83-47-6, Entry# 262359
STIGMAST-5-EN-3-OL #

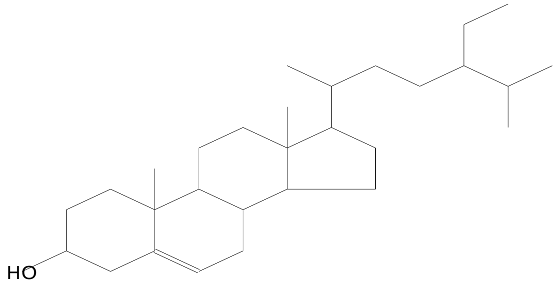


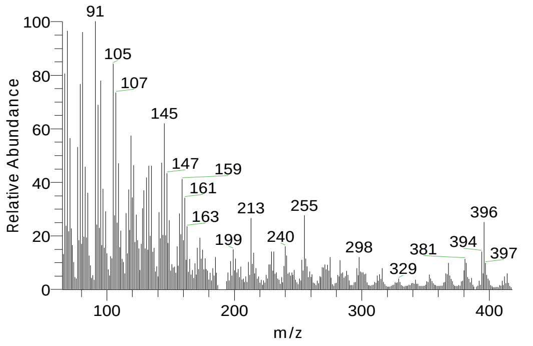


1-Heptatriacotanol
Formula C37H76O, MW 536, CAS# 105794-58-9, Entry# 7279
1-Heptatriacontanol #

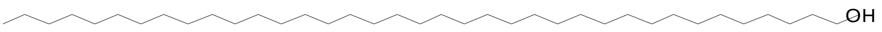


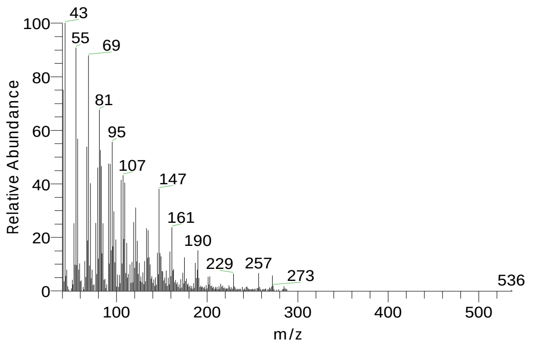

# *My GC-MS Report*

Compound Structure | Hit Spectrum

03027205002 FLAVONE 4'-OH,5-OH,7-DI-O-GLUCOSIDE
Formula C27H30O15, MW 594, CAS# NA, Entry# 296184

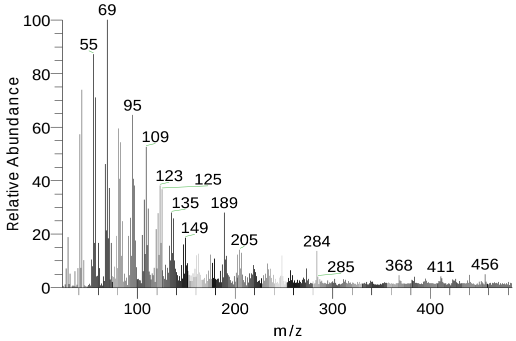


SI 773, RSI 775, mainlib, Entry# 34358, CAS# 105-92-0, Rhodopin

Rhodopin
Formula C40H58O, MW 554, CAS# 105-92-0, Entry# 34358
.psi.,.psi.-Carotene, 1,2-dihydro-1-hydroxy-

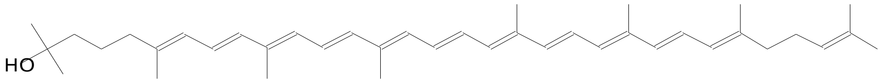


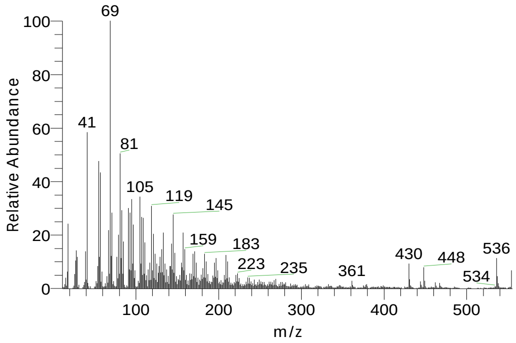


.PSI.,.PSI.-CAROTENE, 1,2-DIHYDRO-1-HYDROXY-
Formula C40H58O, MW 554, CAS# 105-92-0, Entry# 292854
1,2-DIHYDRO-PSI,PSI-CAROTENE #

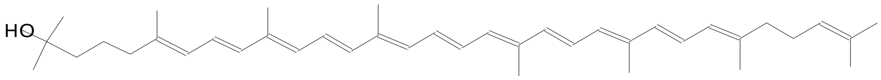


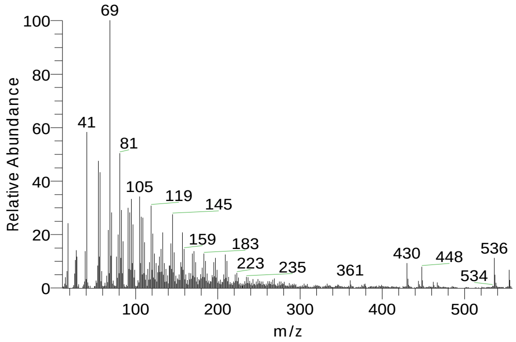


14008 #10898 RT: 40.54 AV: 1 NL: 5.47E6
T: + c EI Full ms [50.000-750.000]

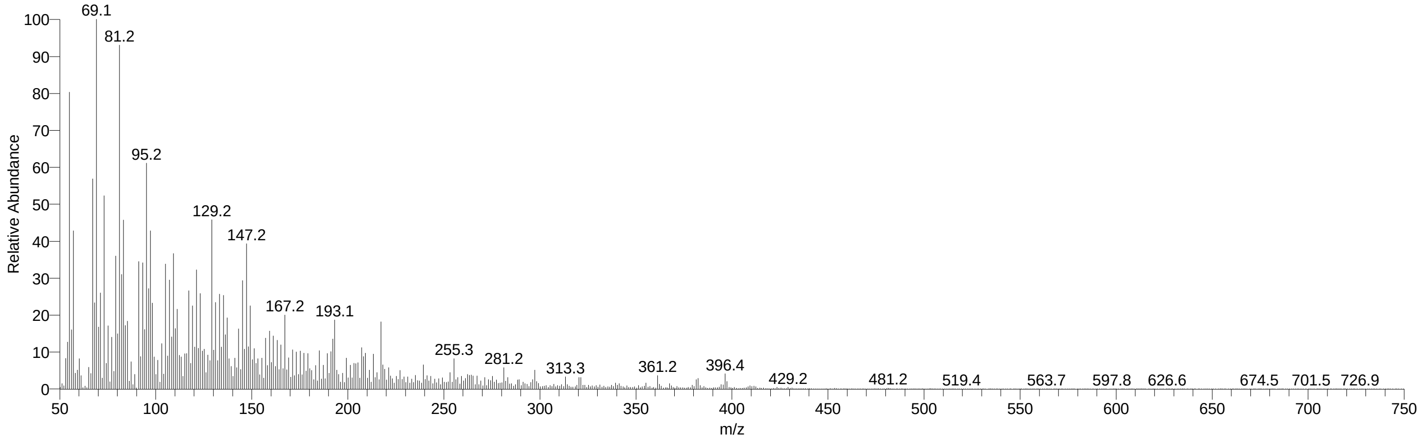


| RT | Compound Name | Area % | MF | Molecular Formula | Molecular Weight | Cas # | Library |
|---|---|---|---|---|---|---|---|
| 40.54 | STIGMAST-5-EN-3-OL, (3á,24S)- | 0.87 | 822 | C29H50O | 414 | 83-47-6 | WileyRegistry8e |
| 40.54 | 03027205002 FLAVONE 4'-OH,5-OH,7-DI-O-GLUCOSIDE | 0.87 | 777 | C27H30O15 | 594 | NA | WileyRegistry8e |

# My GC-MS Report

| RT | Compound Name | Area % | MF | Molecular Formula | Molecular Weight | Cas # | Library |
|---|---|---|---|---|---|---|---|
| 40.54 | 1-Heptatriacotanol | 0.87 | 832 | C37H76O | 536 | 105794-58-9 | mainlib |
| 40.54 | .psi.,.psi.-Carotene, 1,1',2,2'-tetrahydro-1,1'-dimethoxy- | 0.87 | 765 | C42H64O2 | 600 | 13833-01-7 | mainlib |
| 40.54 | Rhodopin | 0.87 | 767 | C40H58O | 554 | 105-92-0 | mainlib |

Compound Structure | Hit Spectrum

STIGMAST-5-EN-3-OL, (3á,24S)-
Formula C29H50O, MW 414, CAS# 83-47-6, Entry# 262359
STIGMAST-5-EN-3-OL #

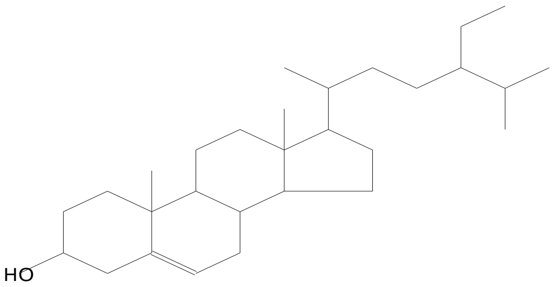


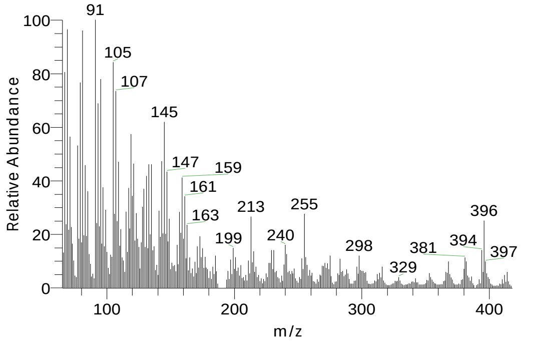


03027205002 FLAVONE 4'-OH,5-OH,7-DI-O-GLUCOSIDE
Formula C27H30O15, MW 594, CAS# NA, Entry# 296184

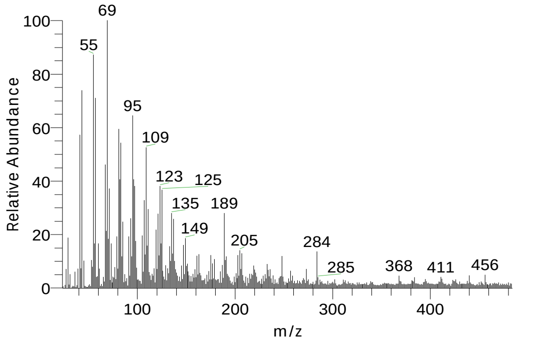


1-Heptatriacotanol
Formula C37H76O, MW 536, CAS# 105794-58-9, Entry# 7279
1-Heptatriacontanol #

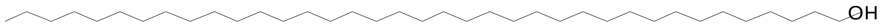


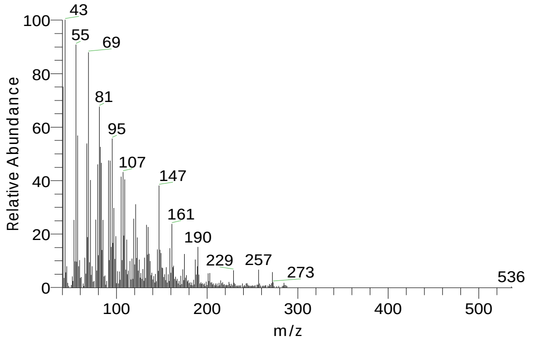


.psi.,.psi.-Carotene, 1,1',2,2'-tetrahydro-1,1'-dimethoxy-
Formula C42H64O2, MW 600, CAS# 13833-01-7, Entry# 41205
Lycopene, 1,1',2,2'-tetrahydro-1,1'-dimethoxy-, all-trans-

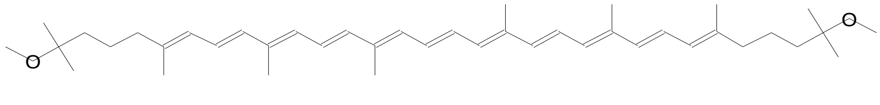


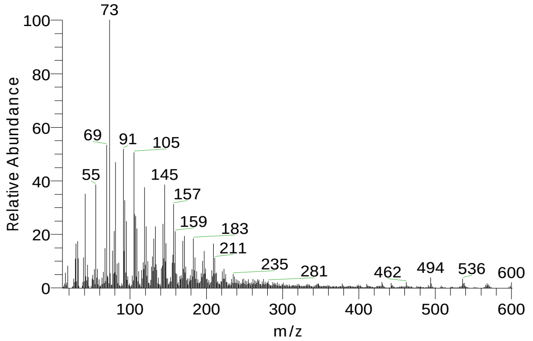

# My GC-MS Report

Compound Structure | Hit Spectrum

Rhodopin
Formula C40H58O, MW 554, CAS# 105-92-0, Entry# 34358
.psi.,.psi.-Carotene, 1,2-dihydro-1-hydroxy-

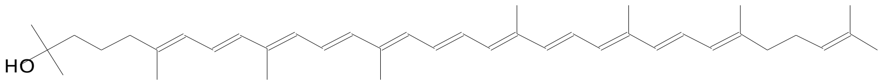


SI 764, RSI 767, mainlib, Entry# 34358, CAS# 105-92-0, Rhodopin

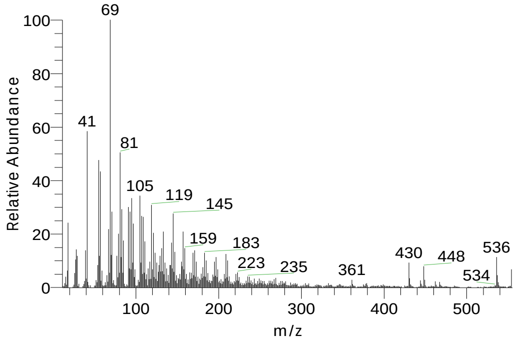


14008 #11062 RT: 41.09 AV: 1 NL: 4.08E6
T: + c EI Full ms [50.000-750.000]

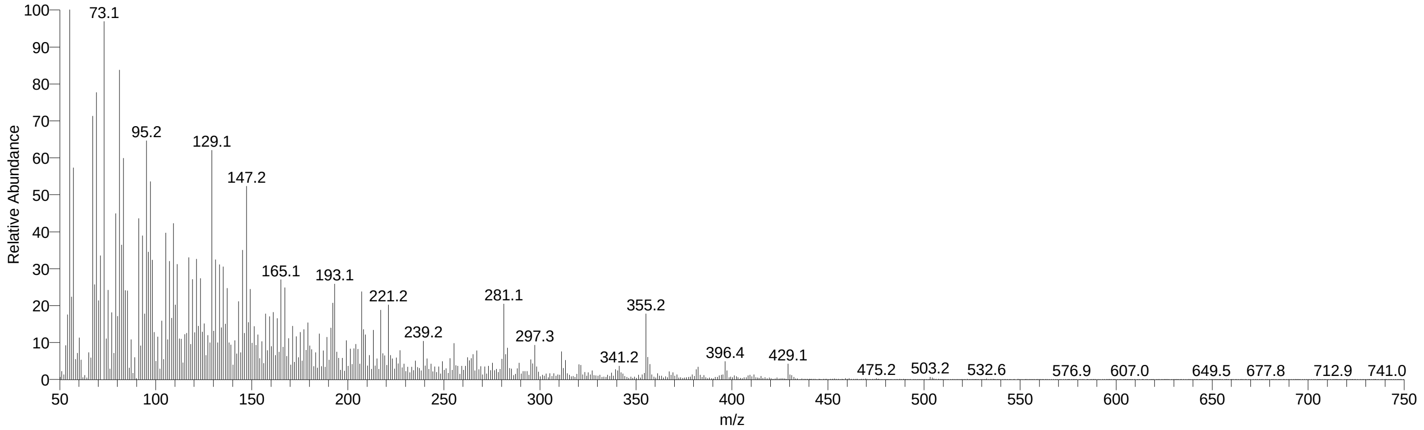


| RT | Compound Name | Area % | MF | Molecular Formula | Molecular Weight | Cas # | Library |
|---|---|---|---|---|---|---|---|
| 41.09 | STIGMAST-5-EN-3-OL, (3á,24S)- | 1.18 | 810 | C29H50O | 414 | 83-47-6 | WileyRegistry8e |
| 41.09 | 03027205002 FLAVONE 4'-OH,5-OH,7-DI-O-GLUCOSIDE | 1.18 | 779 | C27H30O15 | 594 | NA | WileyRegistry8e |
| 41.09 | 4H-1-BENZOPYRAN-4-ONE, 2-(3,4-DIHYDROXYPHENYL)-6,8-DI-á-D-GLUCOPYRANOSYL-5,7-DIHYDROXY- | 1.18 | 776 | C27H30O16 | 610 | 29428-58-8 | WileyRegistry8e |
| 41.09 | Rhodopin | 1.18 | 762 | C40H58O | 554 | 105-92-0 | mainlib |
| 41.09 | .psi.,.psi.-Carotene, 1,1',2,2'-tetrahydro-1,1'-dimethoxy- | 1.18 | 759 | C42H64O2 | 600 | 13833-01-7 | mainlib |

Compound Structure | Hit Spectrum

STIGMAST-5-EN-3-OL, (3á,24S)-
Formula C29H50O, MW 414, CAS# 83-47-6, Entry# 262359
STIGMAST-5-EN-3-OL #

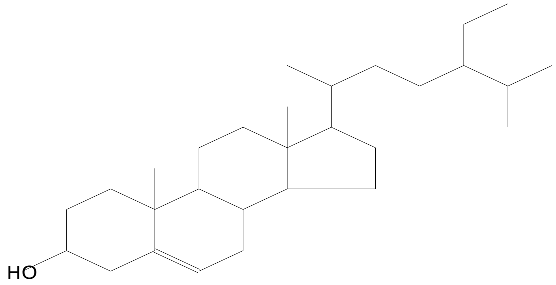


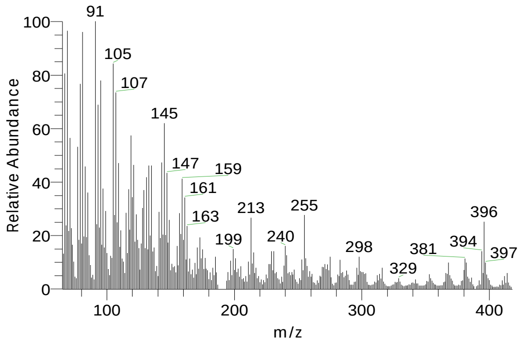

# My GC-MS Report

Compound Structure | Hit Spectrum

03027205002 FLAVONE 4'-OH,5-OH,7-DI-O-GLUCOSIDE
Formula C27H30O15, MW 594, CAS# NA, Entry# 296184

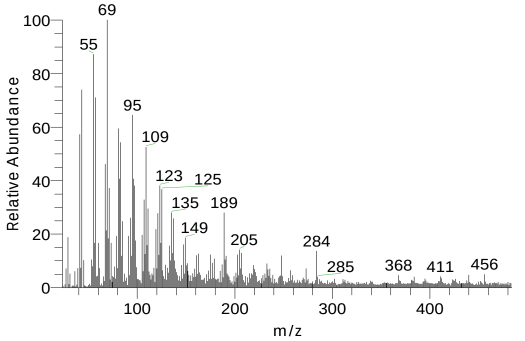


Formula C27H30O16, MW 610, CAS# 29428-58-8, Entry# 297453
6,8-DI-C-á-GLUCOSYLLUTEOLIN

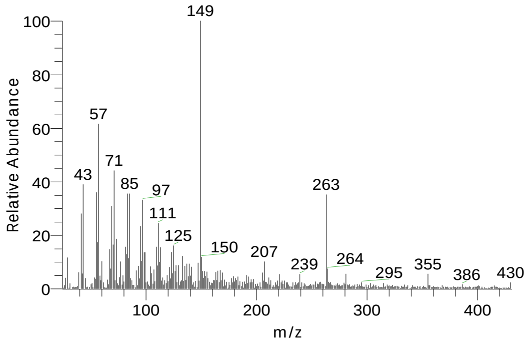


Rhodopin
Formula C40H58O, MW 554, CAS# 105-92-0, Entry# 34358
.psi.,.psi.-Carotene, 1,2-dihydro-1-hydroxy-

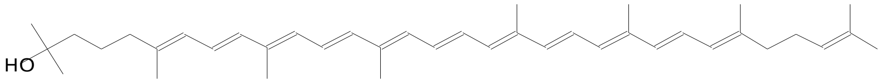


SI 760, RSI 762, mainlib, Entry# 34358, CAS# 105-92-0, Rhodopin

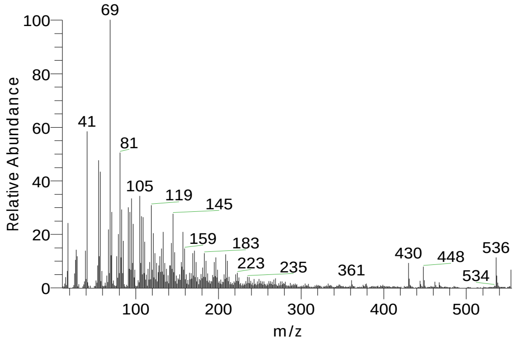


.psi.,.psi.-Carotene, 1,1',2,2'-tetrahydro-1,1'-dimethoxy-
Formula C42H64O2, MW 600, CAS# 13833-01-7, Entry# 41205
Lycopene, 1,1',2,2'-tetrahydro-1,1'-dimethoxy-, all-trans-

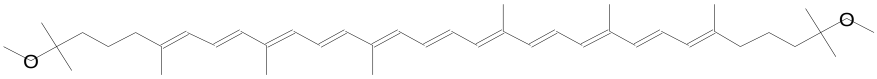


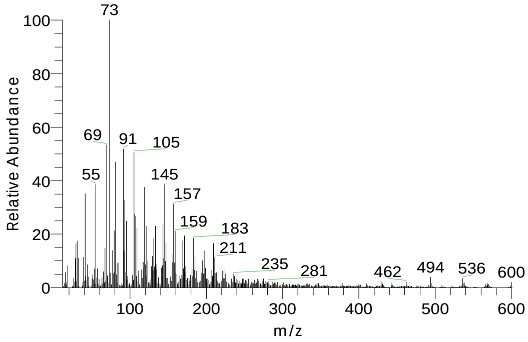

# My GC-MS Report

14008 #11114 RT: 41.27 AV: 1 NL: 5.22E6
T: + c EI Full ms [50.000-750.000]

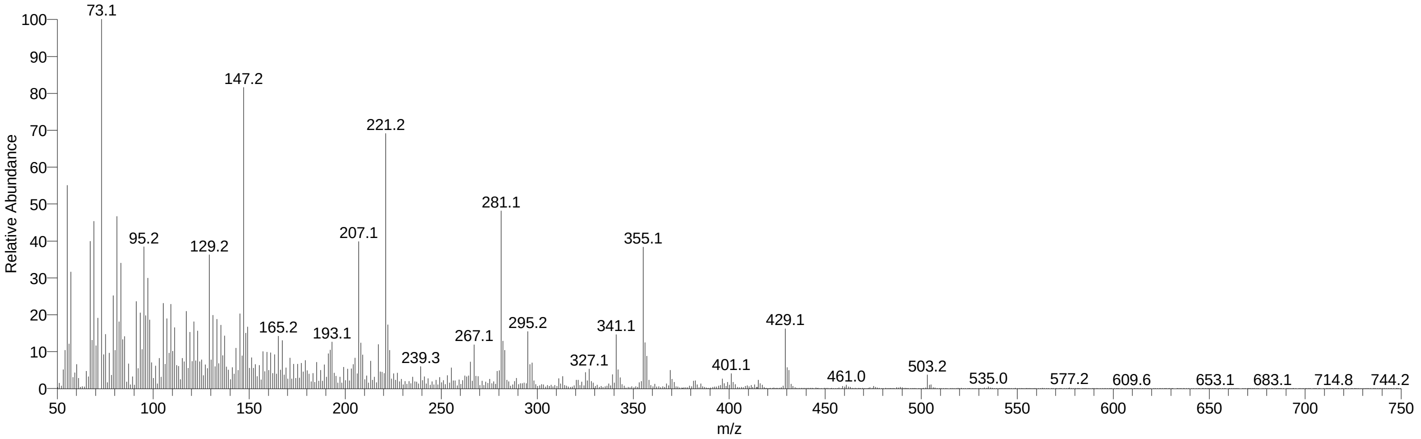


| RT | Compound Name | Area % | MF | Molecular Formula | Molecular Weight | Cas # | Library |
|---|---|---|---|---|---|---|---|
| 41.27 | 9,12-OCTADECADIENOIC ACID (Z,Z)-, 2,3-BIS[(TRIMETHYLSILYL)OXY]PROPYL ESTER | 0.44 | 809 | C27H54O4Si2 | 498 | 54284-45-6 | WileyRegistry8e |
| 41.27 | 9-OCTADECENOIC ACID (Z)-, 2-[(TRIMETHYLSILYL)OXY]-1-[[(TRIMETHYLSILYL)OXY]METHYL]ETHYL ESTER | 0.44 | 780 | C27H56O4Si2 | 500 | 54284-48-9 | WileyRegistry8e |
| 41.27 | .psi.,.psi.-Carotene, 1,1',2,2'-tetrahydro-1,1'-dimethoxy- | 0.44 | 708 | C42H64O2 | 600 | 13833-01-7 | mainlib |
| 41.27 | .PSI.,.PSI.-CAROTENE, 1,1',2,2'-TETRAHYDRO-1,1'-DIMETHOXY- | 0.44 | 708 | C42H64O2 | 600 | 13833-01-7 | WileyRegistry8e |
| 41.27 | 9,12,15-OCTADECATRIENOIC ACID, 2,3-BIS[(TRIMETHYLSILYL)OXY]PROPYL ESTER, (Z,Z,Z)- | 0.44 | 747 | C27H52O4Si2 | 496 | 55521-22-7 | WileyRegistry8e |

| Compound Structure | Hit Spectrum |
|---|---|

9,12-OCTADECADIENOIC ACID (Z,Z)-, 2,3-BIS[(TRIMETHYLSILYL)OXY]PROPYL ESTER
Formula C27H54O4Si2, MW 498, CAS# 54284-45-6, Entry# 285148
2,3-BIS[(TRIMETHYLSILYL)OXY]PROPYL (9Z,12Z)-9,12-OCTADECADIENOATE #

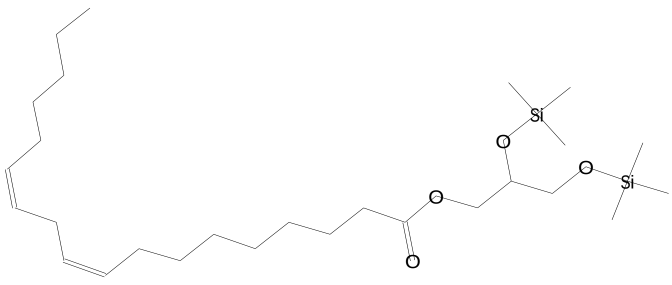

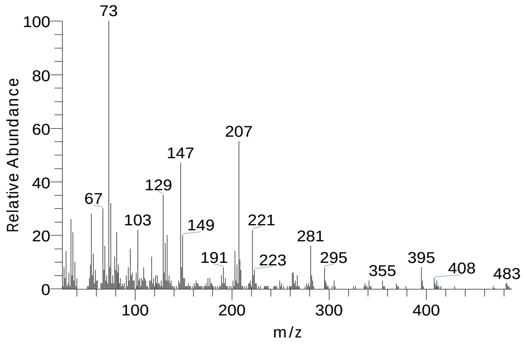


Formula C27H56O4Si2, MW 500, CAS# 54284-48-9, Entry# 285456
2-MONOOLEOYLGLYCEROL TRIMETHYLSILYL ETHER

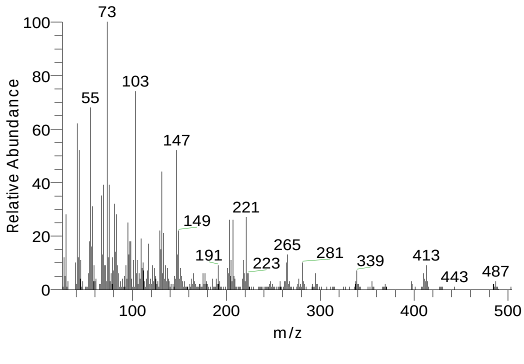

# *My GC-MS Report*

Compound Structure | Hit Spectrum

.psi.,.psi.-Carotene, 1,1',2,2'-tetrahydro-1,1'-dimethoxy-
Formula C42H64O2, MW 600, CAS# 13833-01-7, Entry# 41205
Lycopene, 1,1',2,2'-tetrahydro-1,1'-dimethoxy-, all-trans-

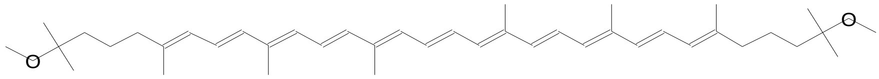


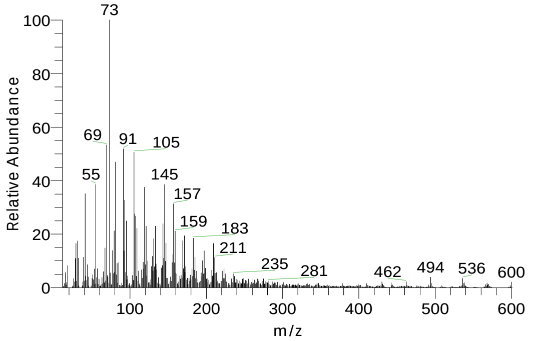


.PSI.,.PSI.-CAROTENE, 1,1',2,2'-TETRAHYDRO-1,1'-DIMETHOXY-
Formula C42H64O2, MW 600, CAS# 13833-01-7, Entry# 296796
1,1',2,2'-TETRAHYDRO-PSI,PSI-CAROTENE #

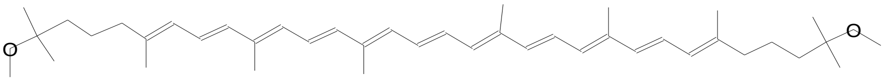


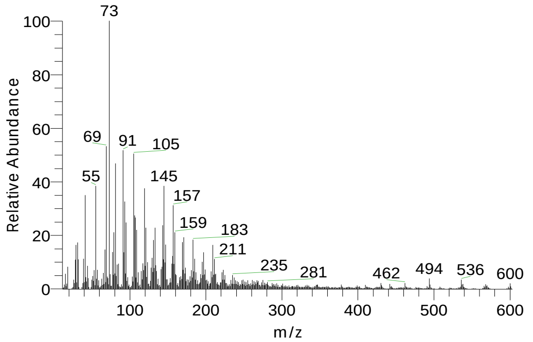


9,12,15-OCTADECATRIENOIC ACID, 2,3-BIS[(TRIMETHYLSILYL)OXY]PROPYL ESTER, (Z,Z,Z)-
Formula C27H52O4Si2, MW 496, CAS# 55521-22-7, Entry# 284833
2,3-BIS[(TRIMETHYLSILYL)OXY]PROPYL (9E,12E,15E)-9,12,15-OCTADECATRIENOATE #

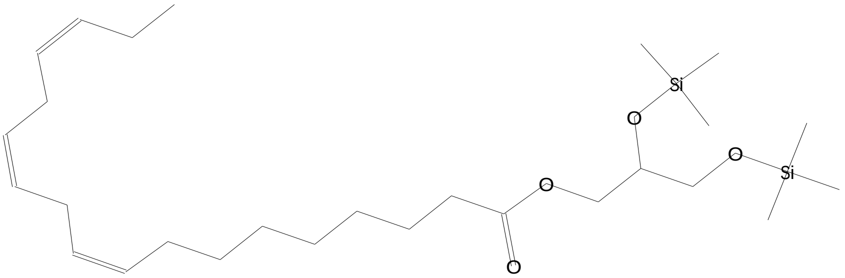


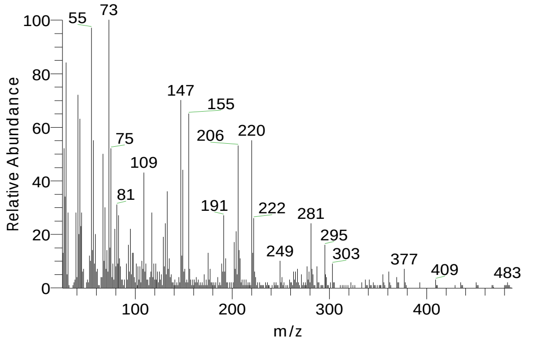


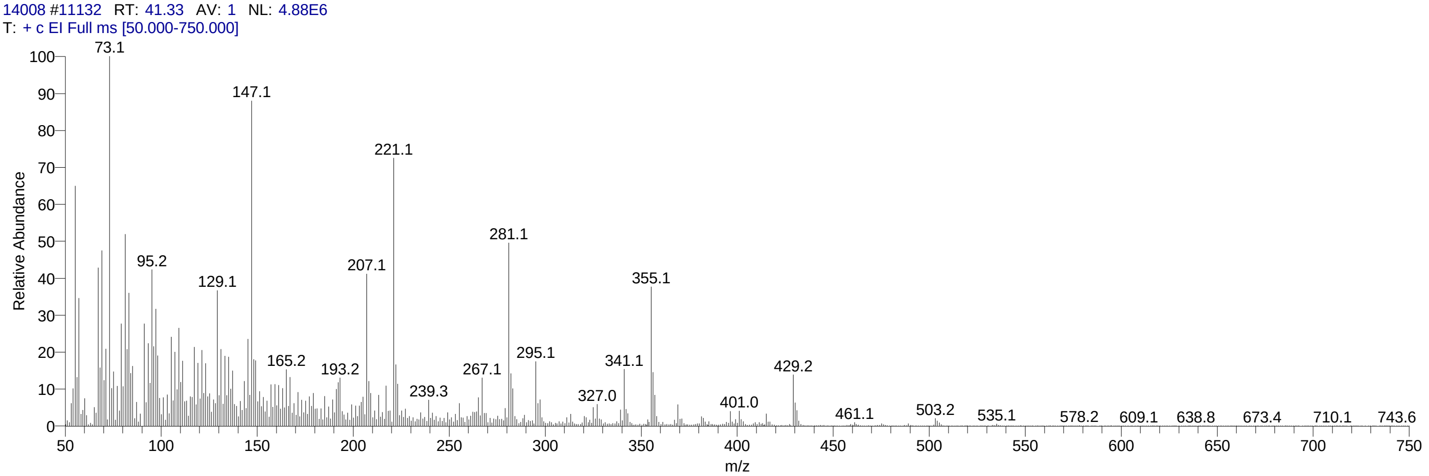


| RT | Compound Name | Area % | MF | Molecular Formula | Molecular Weight | Cas # | Library |
|---|---|---|---|---|---|---|---|
| 41.33 | 4H-1-BENZOPYRAN-4-ONE, 2-(3,4-DIMETHOXYPHENYL)-3,5-DIHYDROXY-7-METHOXY- | 0.43 | 810 | C18H16O7 | 344 | 6068-80-0 | WileyRegistry8e |

# My GC-MS Report

| RT | Compound Name | Area % | MF | Molecular Formula | Molecular Weight | Cas # | Library |
|---|---|---|---|---|---|---|---|
| 41.33 | 9,12-OCTADECADIENOIC ACID (Z,Z)-, 2,3-BIS[(TRIMETHYLSILYL)OXY]PROPYL ESTER | 0.43 | 812 | C27H54O4Si2 | 498 | 54284-45-6 | WileyRegistry8e |
| 41.33 | 9-OCTADECENOIC ACID (Z)-, 2-[(TRIMETHYLSILYL)OXY]-1-[[(TRIMETHYLSILYL)OXY]METHYL]ETHYL ESTER | 0.43 | 771 | C27H56O4Si2 | 500 | 54284-48-9 | WileyRegistry8e |
| 41.33 | .psi.,.psi.-Carotene, 1,1',2,2'-tetrahydro-1,1'-dimethoxy- | 0.43 | 705 | C42H64O2 | 600 | 13833-01-7 | mainlib |
| 41.33 | .PSI.,.PSI.-CAROTENE, 1,1',2,2'-TETRAHYDRO-1,1'-DIMETHOXY- | 0.43 | 704 | C42H64O2 | 600 | 13833-01-7 | WileyRegistry8e |

Compound Structure | Hit Spectrum

4H-1-BENZOPYRAN-4-ONE, 2-(3,4-DIMETHOXYPHENYL)-3,5-DIHYDROXY-7-METHOXY-
Formula C18H16O7, MW 344, CAS# 6068-80-0, Entry# 224392
3',4',7-TRIMETHYLQUERCETIN

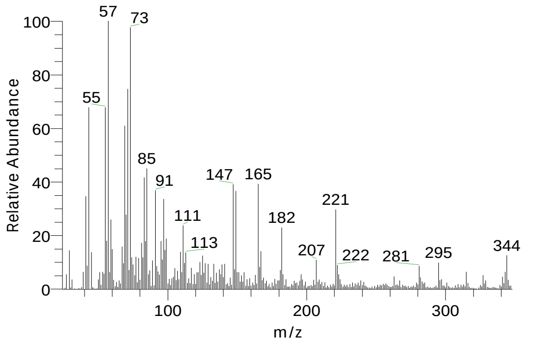


9,12-OCTADECADIENOIC ACID (Z,Z)-, 2,3-BIS[(TRIMETHYLSILYL)OXY]PROPYL ESTER
Formula C27H54O4Si2, MW 498, CAS# 54284-45-6, Entry# 285148
2,3-BIS[(TRIMETHYLSILYL)OXY]PROPYL (9Z,12Z)-9,12-OCTADECADIENOATE #

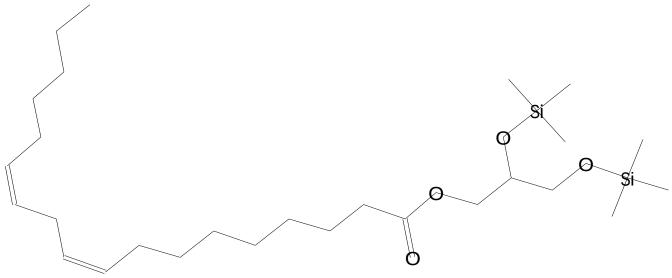


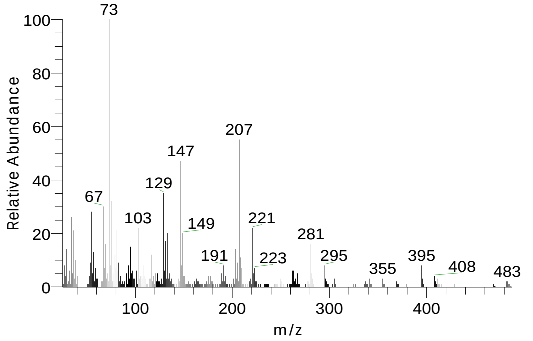


Formula C27H56O4Si2, MW 500, CAS# 54284-48-9, Entry# 285456
2-MONOOLEOYLGLYCEROL TRIMETHYLSILYL ETHER

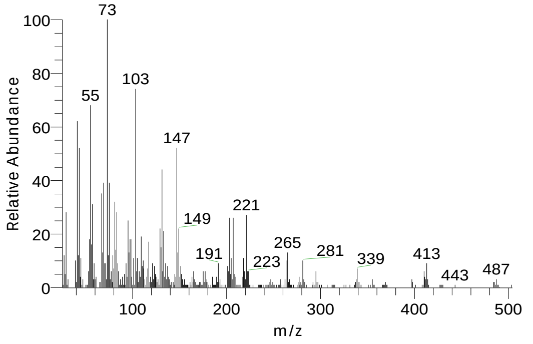

# *My GC-MS Report*

Compound Structure | Hit Spectrum

.psi.,.psi.-Carotene, 1,1',2,2'-tetrahydro-1,1'-dimethoxy-
Formula C42H64O2, MW 600, CAS# 13833-01-7, Entry# 41205
Lycopene, 1,1',2,2'-tetrahydro-1,1'-dimethoxy-, all-trans-

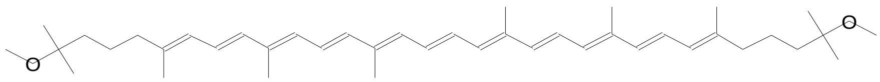

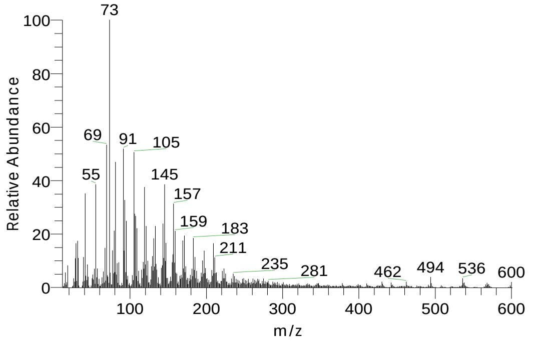


.PSI.,.PSI.-CAROTENE, 1,1',2,2'-TETRAHYDRO-1,1'-DIMETHOXY-
Formula C42H64O2, MW 600, CAS# 13833-01-7, Entry# 296796
1,1',2,2'-TETRAHYDRO-PSI,PSI-CAROTENE #

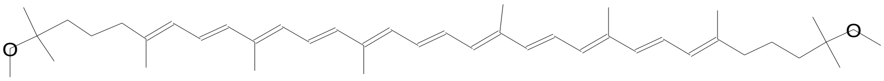

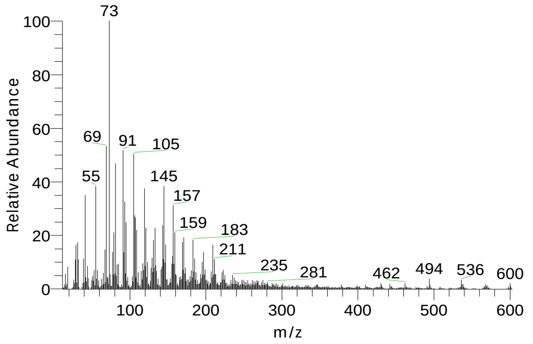


14008 #11388 RT: 42.19 AV: 1 NL: 4.62E6
T: + c EI Full ms [50.000-750.000]

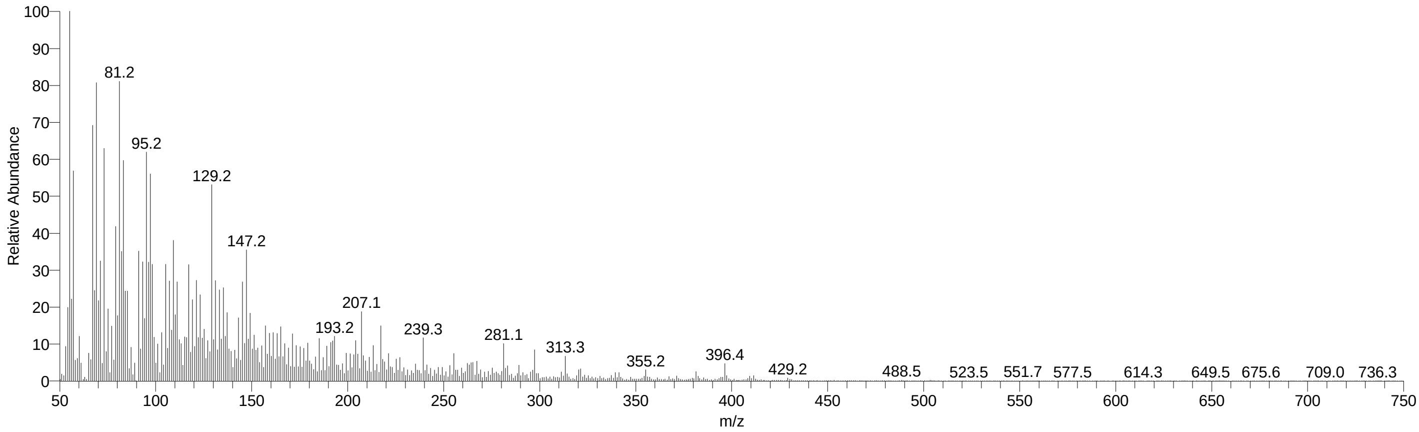


| RT | Compound Name | Area % | MF | Molecular Formula | Molecular Weight | Cas # | Library |
|---|---|---|---|---|---|---|---|
| 42.19 | STIGMAST-5-EN-3-OL, (3á,24S)- | 1.49 | 810 | C29H50O | 414 | 83-47-6 | WileyRegistry8e |
| 42.19 | 03027205002 FLAVONE 4'-OH,5-OH,7-DI-O-GLUCOSIDE | 1.49 | 782 | C27H30O15 | 594 | NA | WileyRegistry8e |
| 42.19 | Rhodopin | 1.49 | 766 | C40H58O | 554 | 105-92-0 | mainlib |
| 42.19 | 1-Heptatriacotanol | 1.49 | 827 | C37H76O | 536 | 105794-58-9 | mainlib |
| 42.19 | .PSI.,.PSI.-CAROTENE, 1,2-DIHYDRO-1-HYDROXY- | 1.49 | 763 | C40H58O | 554 | 105-92-0 | WileyRegistry8e |

# *My GC-MS Report*

Compound Structure | Hit Spectrum

STIGMAST-5-EN-3-OL, (3á,24S)-
Formula C29H50O, MW 414, CAS# 83-47-6, Entry# 262359
STIGMAST-5-EN-3-OL #

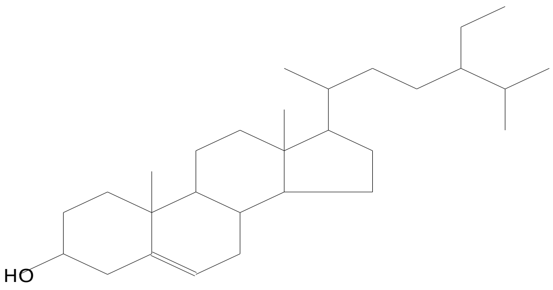


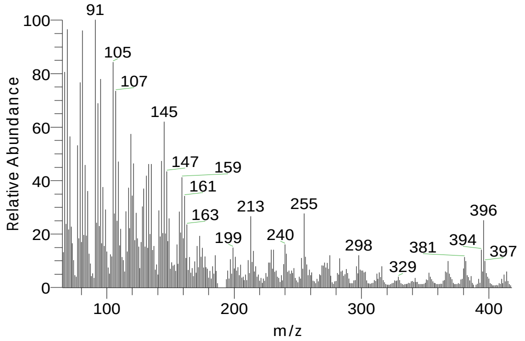


03027205002 FLAVONE 4'-OH,5-OH,7-DI-O-GLUCOSIDE
Formula C27H30O15, MW 594, CAS# NA, Entry# 296184

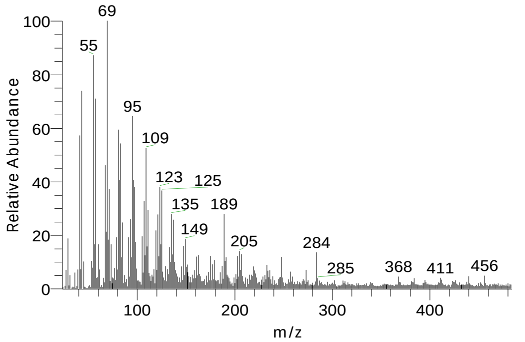


Rhodopin
Formula C40H58O, MW 554, CAS# 105-92-0, Entry# 34358
.psi.,.psi.-Carotene, 1,2-dihydro-1-hydroxy-

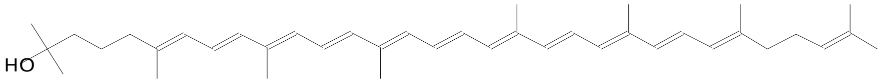


SI 765, RSI 766, mainlib, Entry# 34358, CAS# 105-92-0, Rhodopin

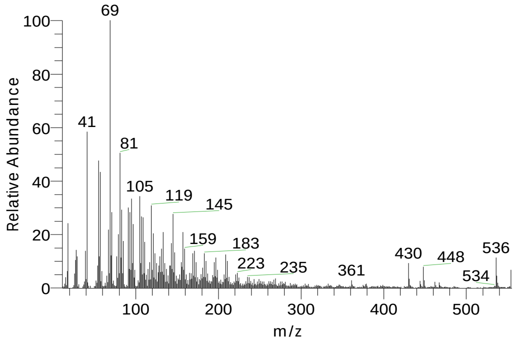


1-Heptatriacotanol
Formula C37H76O, MW 536, CAS# 105794-58-9, Entry# 7279
1-Heptatriacontanol #

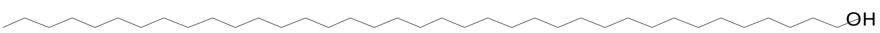


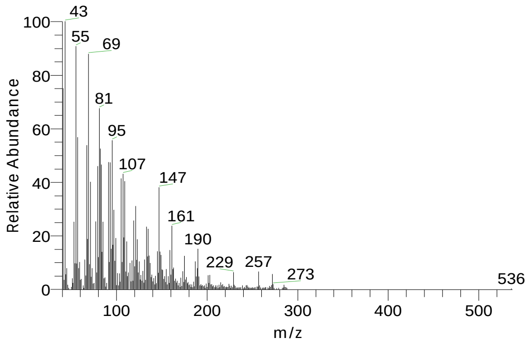

# My GC-MS Report

Compound Structure | Hit Spectrum

.PSI.,.PSI.-CAROTENE, 1,2-DIHYDRO-1-HYDROXY-
Formula C40H58O, MW 554, CAS# 105-92-0, Entry# 292854
1,2-DIHYDRO-PSI,PSI-CAROTENE #

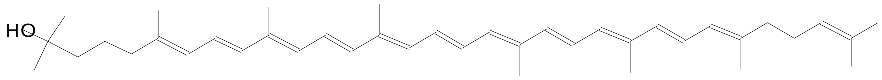


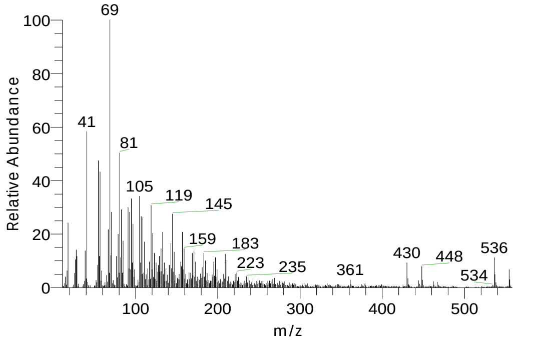


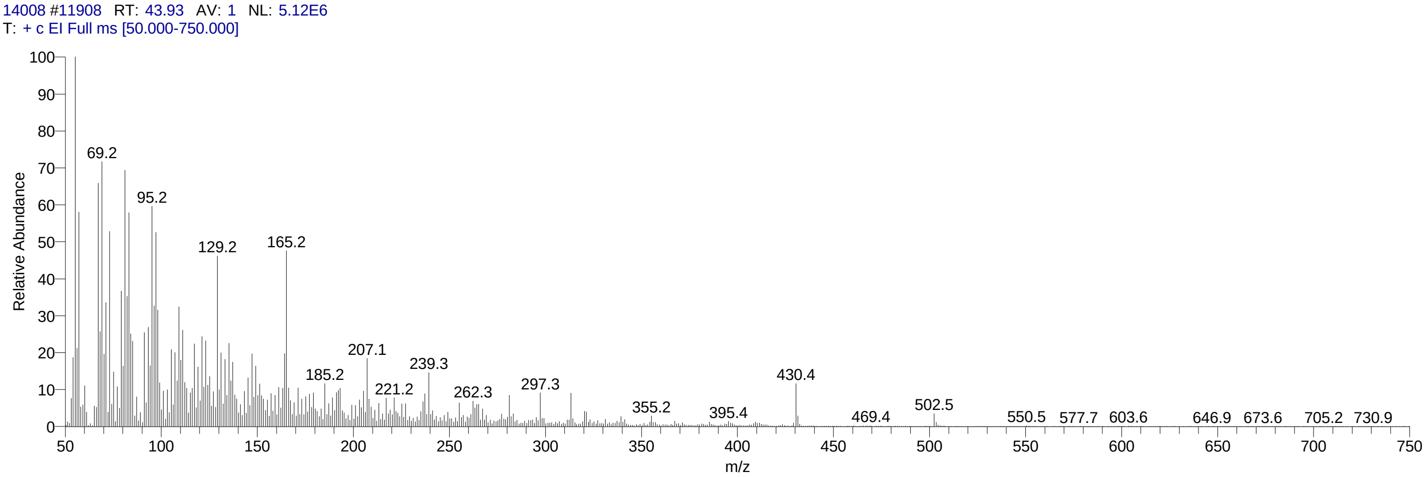


| RT | Compound Name | Area % | MF | Molecular Formula | Molecular Weight | Cas # | Library |
|---|---|---|---|---|---|---|---|
| 43.93 | 03027205002 FLAVONE 4'-OH,5-OH,7-DI-O-GLUCOSIDE | 2.17 | 791 | C27H30O15 | 594 | NA | WileyRegistry8e |
| 43.93 | 4H-1-BENZOPYRAN-4-ONE, 2-(3,4-DIHYDROXYPHENYL)-6,8-DI-á-D-GLUCOPYRANOSYL-5,7-DIHYDROXY- | 2.17 | 784 | C27H30O16 | 610 | 29428-58-8 | WileyRegistry8e |
| 43.93 | Rhodopin | 2.17 | 764 | C40H58O | 554 | 105-92-0 | mainlib |
| 43.93 | Trilinolein | 2.17 | 780 | C57H98O6 | 878 | 537-40-6 | mainlib |
| 43.93 | .PSI.,.PSI.-CAROTENE, 1,2-DIHYDRO-1-HYDROXY- | 2.17 | 761 | C40H58O | 554 | 105-92-0 | WileyRegistry8e |

Compound Structure | Hit Spectrum

03027205002 FLAVONE 4'-OH,5-OH,7-DI-O-GLUCOSIDE
Formula C27H30O15, MW 594, CAS# NA, Entry# 296184

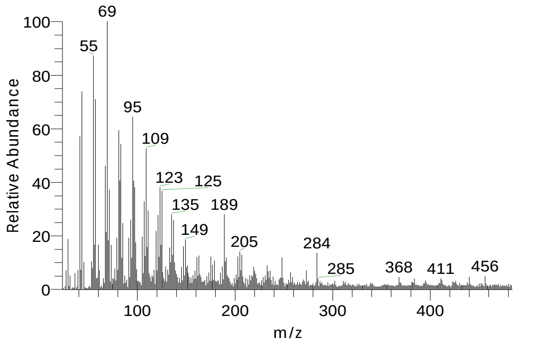

# *My GC-MS Report*

Compound Structure | Hit Spectrum

Formula C27H30O16, MW 610, CAS# 29428-58-8, Entry# 297453
6,8-DI-C-á-GLUCOSYLLUTEOLIN

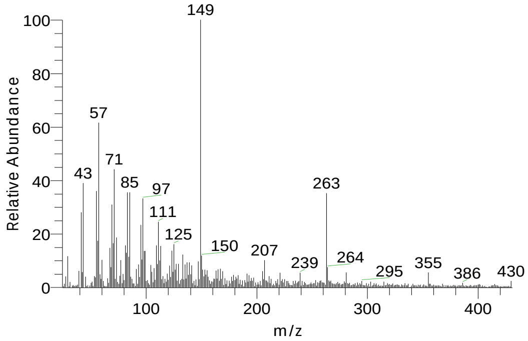


Rhodopin
Formula C40H58O, MW 554, CAS# 105-92-0, Entry# 34358
.psi.,.psi.-Carotene, 1,2-dihydro-1-hydroxy-

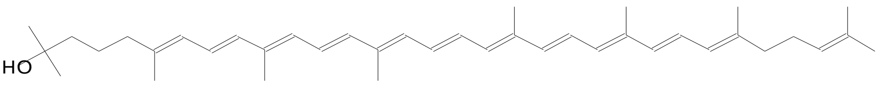


SI 762, RSI 764, mainlib, Entry# 34358, CAS# 105-92-0, Rhodopin

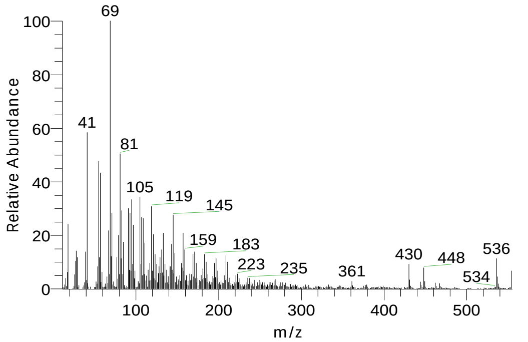


Trilinolein
Formula C57H98O6, MW 878, CAS# 537-40-6, Entry# 32618
9,12-Octadecadienoic acid (Z,Z)-, 1,2,3-propanetriyl ester

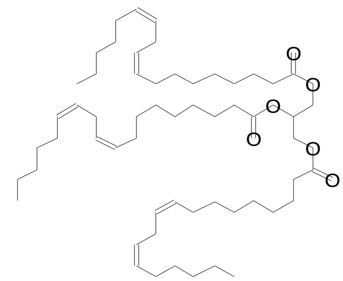

SI 760, RSI 780, mainlib, Entry# 32618, CAS# 537-40-6, Trilinolein

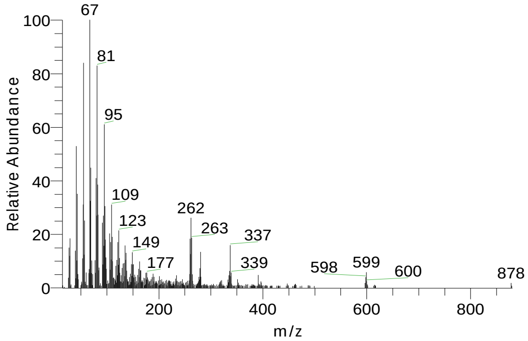


.PSI.,.PSI.-CAROTENE, 1,2-DIHYDRO-1-HYDROXY-
Formula C40H58O, MW 554, CAS# 105-92-0, Entry# 292854
1,2-DIHYDRO-PSI,PSI-CAROTENE #

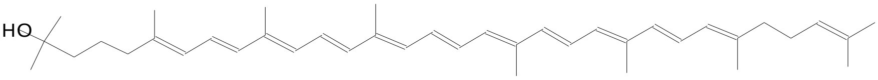


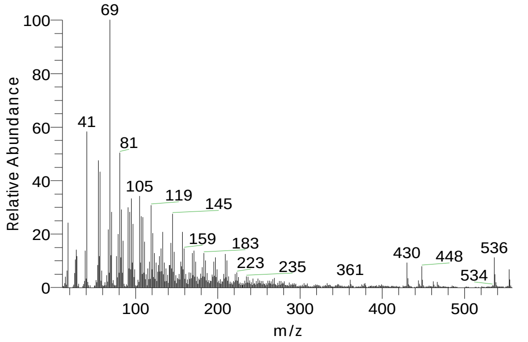

Supplement: Supplementary file 1 [file molecules-28-05543-s001.zip › molecules-2446527-supplementary.pdf]
